# Morphofunctional Analysis of the Quadrate of Spinosauridae (Dinosauria: Theropoda) and the Presence of *Spinosaurus* and a Second Spinosaurine Taxon in the Cenomanian of North Africa.

**DOI:** 10.1371/journal.pone.0144695

**Published:** 2016-01-06

**Authors:** Christophe Hendrickx, Octávio Mateus, Eric Buffetaut

**Affiliations:** 1 GeoBioTec, Faculdade de Ciências e Tecnologia, Universidade Nova de Lisboa, Caparica, Portugal; 2 Museu da Lourinhã, Lourinhã, Portugal; 3 CNRS (UMR 8538), Laboratoire de Géologie de l’École Normale Supérieure, PSL Research University, Paris, France; Monash University, AUSTRALIA

## Abstract

Six quadrate bones, of which two almost certainly come from the Kem Kem beds (Cenomanian, Upper Cretaceous) of south-eastern Morocco, are determined to be from juvenile and adult individuals of Spinosaurinae based on phylogenetic, geometric morphometric, and phylogenetic morphometric analyses. Their morphology indicates two morphotypes evidencing the presence of two spinosaurine taxa ascribed to *Spinosaurus aegyptiacus* and? *Sigilmassasaurus brevicollis* in the Cenomanian of North Africa, casting doubt on the accuracy of some recent skeletal reconstructions which may be based on elements from several distinct species. Morphofunctional analysis of the mandibular articulation of the quadrate has shown that the jaw mechanics was peculiar in Spinosauridae. In mature spinosaurids, the posterior parts of the two mandibular rami displaced laterally when the jaw was depressed due to a lateromedially oriented intercondylar sulcus of the quadrate. Such lateral movement of the mandibular ramus was possible due to a movable mandibular symphysis in spinosaurids, allowing the pharynx to be widened. Similar jaw mechanics also occur in some pterosaurs and living pelecanids which are both adapted to capture and swallow large prey items. Spinosauridae, which were engaged, at least partially, in a piscivorous lifestyle, were able to consume large fish and may have occasionally fed on other prey such as pterosaurs and juvenile dinosaurs.

## Introduction

The Kem Kem region of south-eastern Morocco is very well known for its rich vertebrate assemblage of Cenomanian age, which is characterized by a particularly high diversity of predatory dinosaurs [1–9]. The presence of at least five non-avian theropod clades has been documented in the Kem Kem beds, hitherto including non-abelisaurid Ceratosauria (Noasauridae?), Abelisauridae, Spinosauridae, Carcharodontosauridae, and Dromaeosauridae.

Ceratosaurs are represented by abelisaurids [2,9–12] and *Deltadromeus agilis* [3] interpreted either as a basal form [13,14] or a noasaurid [10,15]. Material resembling the primitive ceratosaur *Elaphrosaurus* was already reported by Lavocat [16], and additional remains of noasaurids have been recently described and may belong to a juvenile individual of *Deltadromeus* [1]. Among tetanurans, spinosaurids are documented by material assigned to two species of *Spinosaurus*, namely *Spinosaurus aegyptiacus* [17–22] and *Spinosaurus maroccanus* [2]. Likewise, carcharodontosaurid allosauroids are represented by at least two taxa: the very large form *Carcharodontosaurus saharicus* [3,16,23], and the thick-skulled *Sauroniops pachytholus* [6,24]. *Sigilmassasaurus brevicollis*, coined by Russell [2] and initially classified to the new clade Sigilmassasauridae, was interpreted as belonging to *Carcharodontosaurus saharicus* [5,23,25] (an hypothesis rejected by Novas et al. [26]), *Spinosaurus maroccanus* [11], and recently to *Spinosaurus aegyptiacus* [22]. Yet, recent investigations on the anatomy of *Sigilmassasaurus brevicollis* based on the holotype material [8] and unpublished specimens housed in the Bayerische Staatssammlung für Paläontologie und Geologie in Munich [27], retained it as a valid taxon of Spinosauridae, and a synonym of *Spinosaurus maroccanus* [8,27]. Finally, Dromaeosauridae, the only known non-avian coelurosaurs from the Kem Kem beds, have so far been documented by isolated teeth [9,28]. *Kemkemia*, an additional theropod of uncertain affinities known from a single caudal vertebra [29], was reinterpreted as belonging to a Crocodyliformes *incertea sedis* [30]. Likewise, an isolated vertebra of avian origin was reported by Riff et al. [31] but does not seem to preserve any avian synapomorphies [5]. Birds seem, however, to be present in the Kem Kem beds alongside non-avian dinosaurs [5].

Various scenarios have been suggested to explain such a large diversity of theropods while herbivorous dinosaurs seem to be rare. The latter are indeed documented by a few ornithopod tracks [32] and sauropods [4,5], known from two clades only, i.e., Rebbachisauridae and Lithostrothia (e.g., [2,3,5,16,33–39]). The dominant theropod assemblage in the Kem Kem was first interpreted by Russell [2] as resulting from an attraction of the predators to the margin of streams which were a major source of prey, or from a food chain linked to large bodies of water. Yet, the apparent scarcity of herbivorous taxa may indicate biased collecting in the Kem Kem area [40]. This overabundance of carnivorous dinosaurs may also be caused by the effect of ‘time-averaging’ in which fossils of different ages are mixed into a single rock layer, therefore altering the interpretation of the ecosystems based on fossil collections [41]. Nonetheless, an unbalanced ratio between herbivorous and carnivorous dinosaurs was clearly observed by Läng et al. [7] based on field data. These authors suggest that such an abundance of predators is linked to a widespread deltaic paleoenvironment with unstable climatic and hydrological features. Such an heterogeneous environment would have indeed favored the existence of many ecological niches, and the very abundant aquatic life could have formed the base of an aquatic or semi-aquatic food chain which could have directly fed top predators [7].

Spinosaurid material appears to be particularly abundant in the Kem Kem beds (spinosaurid teeth represent 60% of the dinosaurian fauna in all considered samples collected in the Ifezouane Formation; [7]), and isolated teeth and cranial and postcranial bones of Spinosauridae have been regularly reported in the literature over the past 30 years [5]. Although a probable spinosaurid tooth from the Kem Kem beds was illustrated and misinterpreted as belonging to *Crocodilus* sp. by Choubert et al. in 1952 [5], spinosaurid material from Morocco was first reported by Taquet [42]. This author was the first to mention the presence of this family in the Kem Kem region, and Buffetaut [17,19] was the first to describe an incomplete maxilla from the continental red beds and refer it to *Spinosaurus* cf. *aegyptiacus*. An International expedition in the Sahara desert in 1995 led to the discovery of additional remains of *Spinosaurus* from the Kem Kem region, including isolated teeth and fused nasals [3,21]. Dentary fragments, a cervical vertebra and a dorsal neural arch collected in the Tafilalt plain (northern part of the Kem Kem region) by locals, allowed Russell [2] to erect a second species of *Spinosaurus*, *S*. *maroccanus*. Isolated teeth from the Kem Kem beds were also reported by Kellner [43] and Sadleir [44] who identified the material as belonging to a spinosaurid and to the genus *Spinosaurus*, respectively. At the beginning of the 21^st^ century, more complete and better preserved skull remains were assigned to the species *S*. *aegyptiacus*. In 2003, Milner [20] briefly described an incomplete snout and a left dentary kept at the Natural History Museum of London. Two years later, Dal Sasso et al. [21] reported a well-preserved snout of very large size collected by locals in 1975, and ascribed the material to *Spinosaurus aegyptiacus*. More recently, the enamel texture of *Spinosaurus* teeth from the Kem Kem was investigated by Hasegawa et al. [45] whereas three morphotypes of isolated teeth assigned to *Spinosaurus* were described by Richter et al. [9] and may attest to the presence of more than one species of *Spinosaurus* in the Kem Kem beds. In 2014, the discovery of new cranial and postcranial material referred to *Spinosaurus aegyptiacus* by an international team of paleontologists in the region of Erfoud (northernmost part of the Kem Kem) provided additional information of this animal’s anatomy and ecology [22]. *Spinosaurus aegyptiacus* was revealed to be a semi-aquatic quadrupedal theropod with short hind limbs and dense postcranial bones [22]. Based on the newly discovered material of *Spinosaurus*, Ibrahim et al. [22] erected a neotype for *Spinosaurus aegyptiacus* and considered that both *Spinosaurus maroccanus* and *Sigilmassasaurus brevicollis* are junior synonyms of *S*. *aegyptiacus*. The recent description of additional material of the spinosaurid *Sigilmassasaurus brevicollis* from the Kem Kem beds by Evers et al.[27] has, however, casted doubt on the synonymy of *Sigilmassasaurus brevicollis* and *Spinosaurus maroccanus* with *Spinosaurus aegyptiacus*, and Evers et al.[27] have argued for the presence of more than one spinosaurid taxon in the Kem Kem compound assemblage.

The functional morphology of the spinosaurid skull was investigated by Therrien et al. [46], Rayfield et al. [47], Rayfield [48] and Cuff and Rayfield [49]. Therrien et al.’s [46] study on the biomechanical properties of the jaws of *Suchomimus* based on beam theory indicates that spinosaurid theropods were specialized in capturing small prey (n.b., given the paleogeographic and stratigraphic distribution of *Cristatusaurus lapparenti* and *Suchomimus tenerensis*, and because the material referred to the two taxa is almost identical, *Suchomimus tenerensis* [25] is most likely a junior synonym of *Cristatusaurus lapparenti*. Yet, no definitive autapomorphy could be found in the *Cristatusaurus* holotype and this taxon is here considered as a *nomen dubium*; Figs A and B in S1 File). The capture of small prey items by *Suchomimus* was possible thanks to its upturned chin with the terminal rosette, large mandibular symphysis allowing to resist the important stresses induced by struggling prey, and conical teeth designed to impale and hold prey and withstand bending loads applied in all directions [46]. Rayfield et al.’s [47] study based on finite element analysis (FEA) on the rostrum of *Baryonyx* reveals that the snout of *Baryonyx* and *Gavialis* are morphologically and functionally homologous in terms of resistance to bending and torsional feeding loads, thereby supporting the hypothesis of a partially piscivorous lifestyle in this theropod as well. Using FEA on 2D models of skulls, Rayfield [48] found that *Suchomimus* and *Spinosaurus* skulls experience cranial stresses in different ways. Whereas *Suchomimus* is scaling in a similar manner to most non-spinosaurid tetanurans, *Spinosaurus* experiences a much higher magnitude of cranial stress than what would be predicted, suggesting it may have fed on smaller prey. This hypothesis was later supported by Cuff and Rayfield [49] whose results of FEA on 3D models of the *Baryonyx* and *Spinosaurus* snout suggest that the crania of both taxa resist well to ventrodorsal bending but are poorly equipped to resist lateromedial and torsional loads.

Here we report additional cranial material of spinosaurids consisting of six isolated quadrates most likely coming from the Kem Kem beds. The quadrate is a cranial bone of endochondral origin that articulates with the mandible in all gnathostomes other than mammals [50–52]. In theropods, the quadrate had many important functions such as a structural support for the basicranium, an articulatory element with the lower jaw, an insertion area for several muscles, and in hosting important nerves, pneumatic sinuses, and vascular passages (e.g., [53–61]). This work aims to investigate the phylogenetic position of the isolated quadrates and the morphofunctional aspects of their mandibular articulations based on cladistic, geometric morphometric, and phylogenetic morphometric analyses.

## Material and Methods

### Material and geological settings

**Fig 1:**
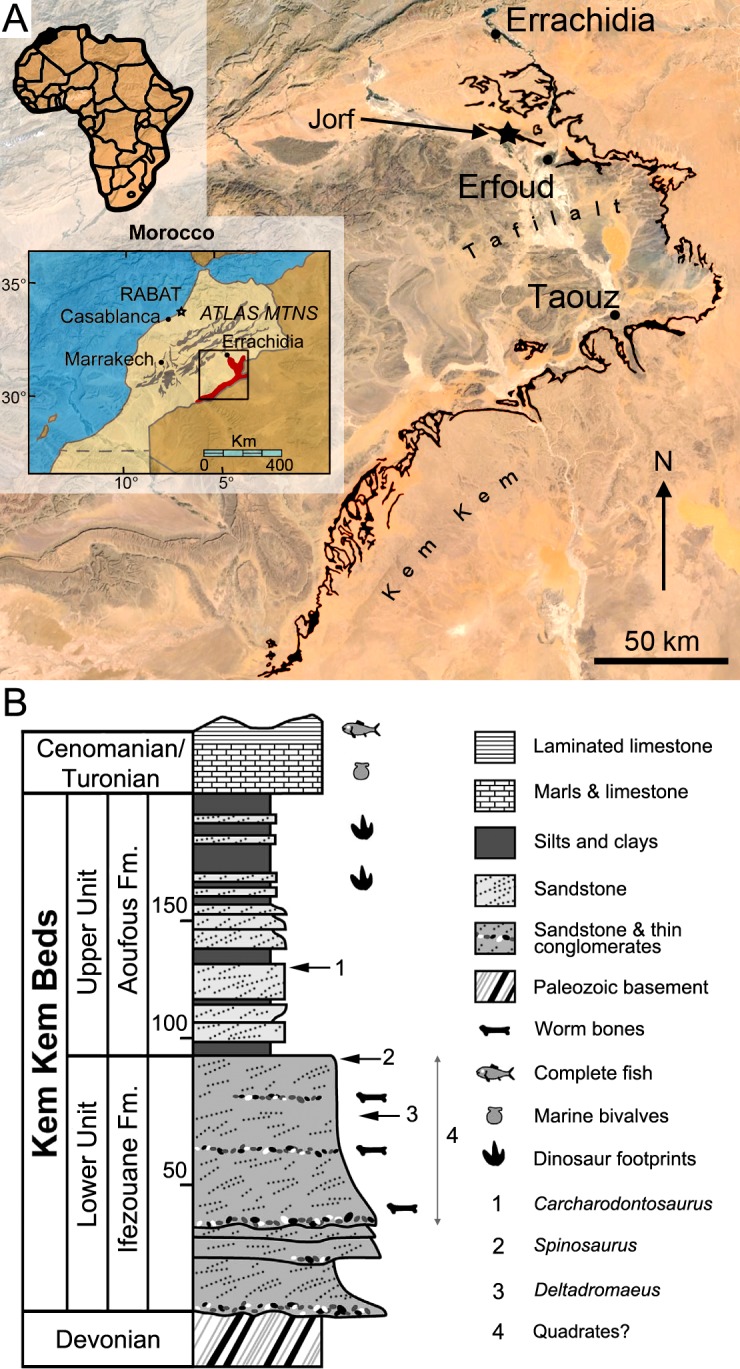
Geographical location and stratigraphy of the Kem Kem beds. **A**, Location of Morocco (in black) in Africa (left corner), the Kem Kem and Tafilalt regions (in red) in Morocco (middle left), and the Kem Kem beds (in black) in the Kem Kem plateau (right). The dark blue star indicates the site of Jorf from which two quadrates were found; **B**, Stratigraphic column of the Kem Kem beds of South-Eastern Morocco. Stratigraphic position of the type remains of **1**, *Carcharodontosaurus saharicus* (neotype; [3,23]); **2**, *Spinosaurus aegyptiacus* (neotype; [22]); and **3**, *Deltadromeus agilis* (holotype; [3]); and **4**, probable stratigraphic position of two spinosaurid quadrates. Modified from Sereno et al. [3] and Ibrahim et al. [32].

**Fig 2:**
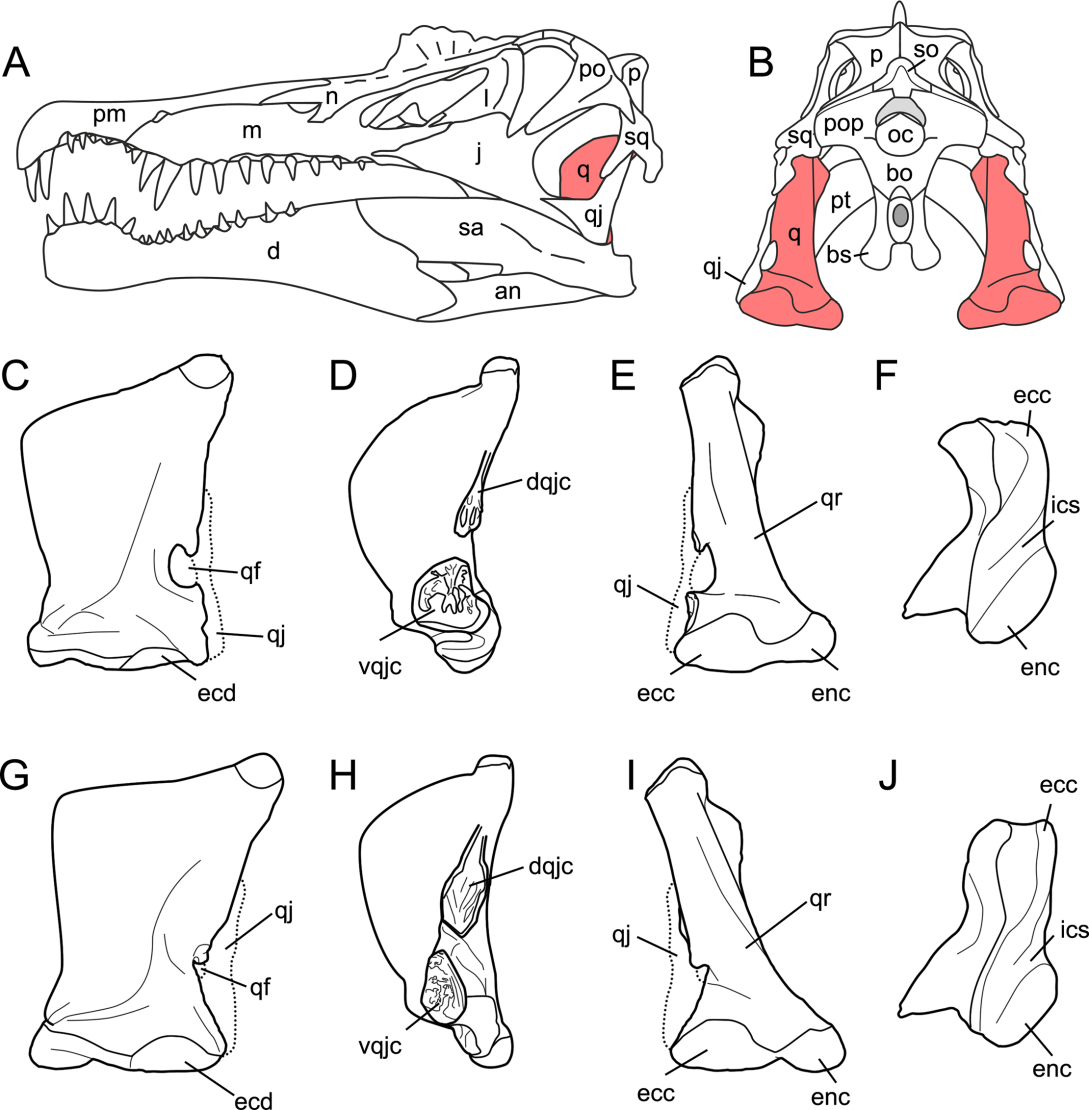
Quadrate position and quadrate morphotypes in Spinosaurinae from the Kem Kem beds. **A**–**B**, Position of the quadrate bone in the *Spinosaurus aegyptiacus* skull in **A**, left lateral; and **B**, occipital views; **C**–**F**, Morphotype 1; and **G**–**J**, reconstructed Morphotype 2 of an idealized left quadrate in **C, E, G, I**, articulation with the quadratojugal (dotted line) of **C**–**F**, *Spinosaurus aegyptiacus* (Morphotype 1); and **G**–**J**,? *Sigilmassasaurus brevicollis* (Morphotype 2), in **C, G**, anterior; **D, H**, lateral; **E, I**, posterior; and **F, J**, ventral views. **Abbreviations**: **an**, angular; **bo**, basioccipital; **bs**, basisphenoid; **d**, dentary; **dqjc**, dorsal quadratojugal contact; **ecc**, ectocondyle; **ecd**, ectocondyle depression; **enc**, entocondyle; **ics**, intercondylar sulcus; **j**, jugal; **l**, lacrimal; **m**, maxilla; **n**, nasal; **oc**, occipital condyle; **p**, parietal; **pm**, premaxilla; **pop**, paroccipital process; **pt**, pterygoid; **q**, quadrate; **qf**, quadrate foramen; **qj**, quadratojugal; **qr**, quadrate ridge; **sa**, surangular; **so**, supraoccipital; **sq**, squamosal; **vqjc**, ventral quadratojugal contact.

**Fig 3:**
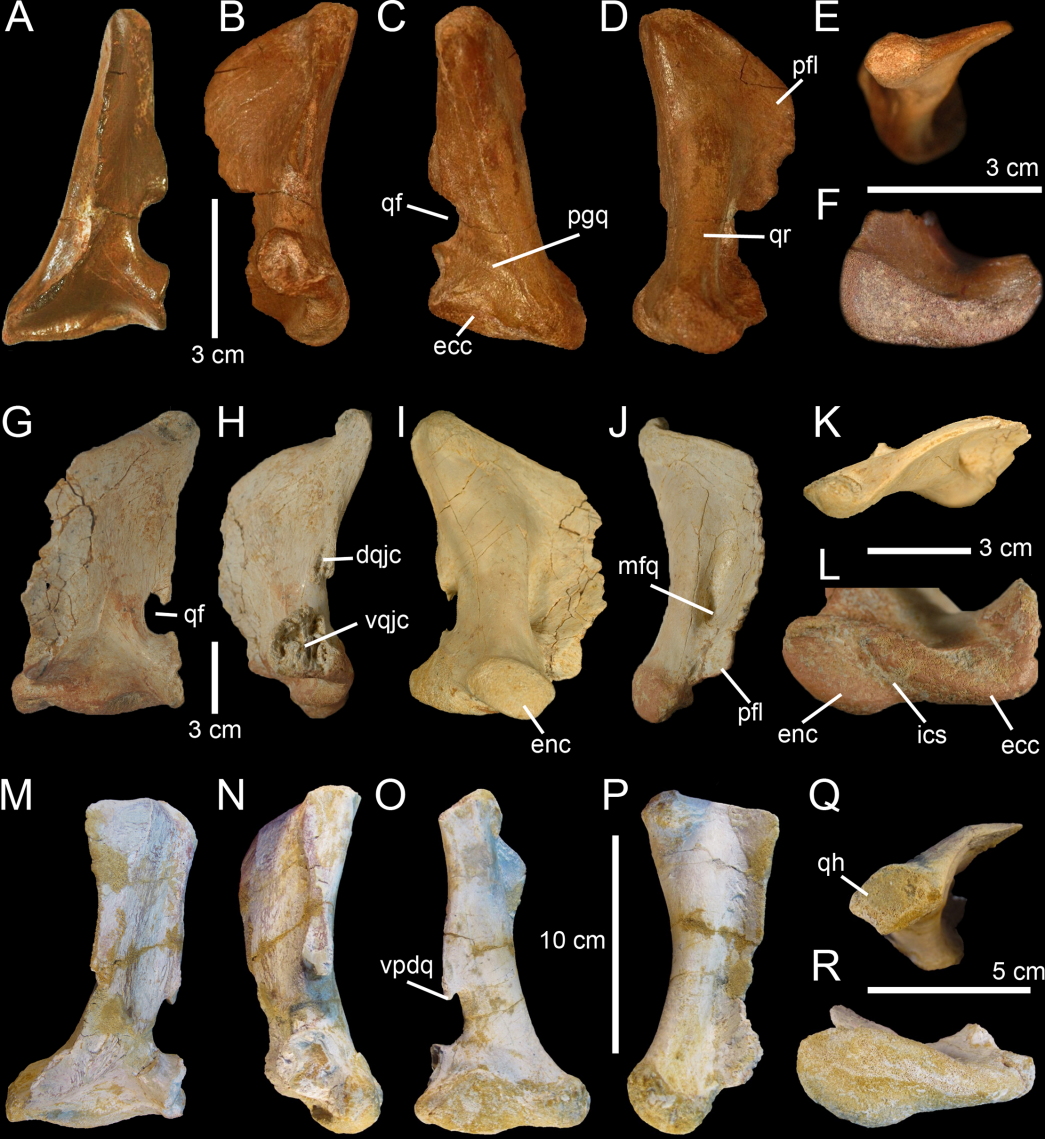
Quadrates of Morphotype 1 referred to *Spinosaurus aegyptiacus*. **A**–**N**, Left quadrates of specimens **A**–**F**, MHNM.KK374; **G**–**L**, MHNM.KK375; and **M**–**N**, MSNM V6896, in **A, G, M**, anterior; **B, H, N**, lateral; **C, O**, posterior; **I**, posteromedial; **D**, posterolateral; **J, P**, lateral; **E, K, P**, dorsal; and **F, L, R**, ventral views. **Abbreviations**: **dqjc**, dorsal quadratojugal contact; **ecc**, ectocondyle; **enc**, entocondyle, **ics**, intercondylar sulcus; **mfq**, medial fossa; **pfl**, pterygoid flange; **pgq**, posterior groove; **qf**, quadrate foramen; **qh**, quadrate head; **qr**, quadrate ridge; **vpdq**, ventral projection of the dorsal quadratojugal contact; **vqjc**, ventral quadratojugal contact.

**Fig 4:**
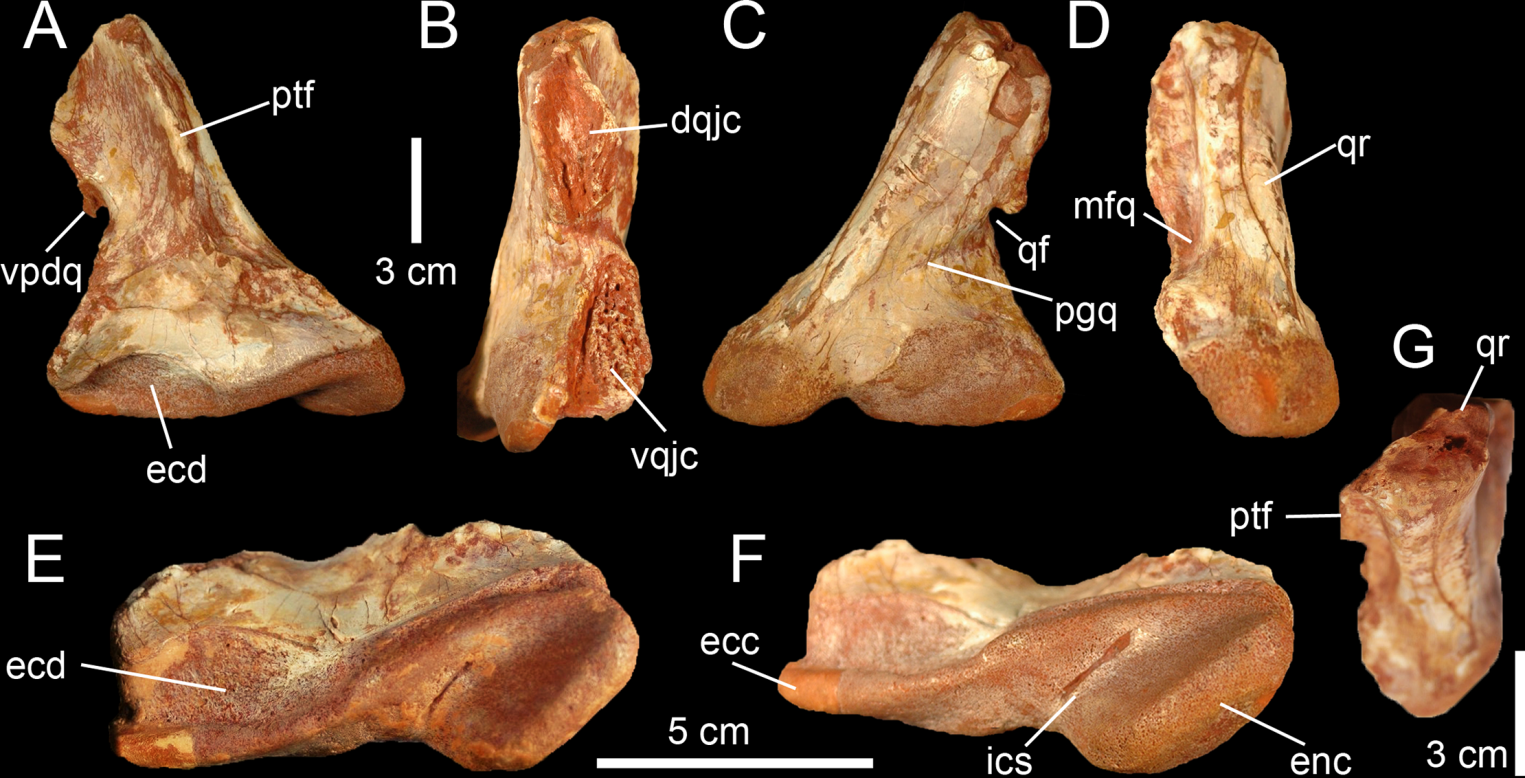
Quadrate of Morphotype 2 referred to *Sigilmassasaurus brevicollis*. **A–F**, Right quadrate MHNM.KK376 in **A**, anterior; **B**, lateral; **C**, posterior; **D**, medial; **E**, ventral; **F**, ventromedial; and **G**, dorsal views. **Abbreviations: dqjc**, dorsal quadratojugal contact; **ecc**, ectocondyle; **ecd**, depression of the ectocondyle; **enc**, entocondyle, ics, intercondylar sulcus; **mfq**, medial fossa; **pfl**, pterygoid flange; **qf**, quadrate foramen; **qr**, quadrate ridge; **vpdq**, ventral projection of the dorsal quadratojugal contact; **vqjc**, ventral quadratojugal contact.

Six isolated quadrates of different sizes were collected by locals and acquired commercially in the Kem Kem region of southeastern Morocco (Fig 1). Five bones (MHNM.KK374 to.KK378; Figs 2–4; Fig C in S1 File) were provided by François Escuillié and are deposited in the collections of the Muséum d’Histoire Naturelle of Marrakech (MHNM; see S1 File for a list of institutional abbreviations). A sixth quadrate (MSNM V6896; Fig 3) was donated to the Museo di Storia Naturale di Milano by an Italian fossil dealer who purchased it from locals (Dal Sasso pers. comm.). Two specimens, MHNM.KK376 and.KK378, were uncovered in reddish to violet sandstones with pebbles near the town of Jorf (Tafilalt) northwest of Erfoud (Fig 1A; Escuillié pers. comm.). As for the remaining specimens, given the general color of the bone and small patches of sediment associated with them, MHNM.KK374 was obviously found in reddish iron-rich sandstones, whereas MHNM.KK375,.KK377 and MSNM V6896 come from ironless layers of white to yellow sandstones.

As most other dinosaur material collected by non-paleontologists in the Kem Kem and reported in the literature (e.g., [1,2,6,9,11,12,21,24,26]), the exact horizon and precise locality of the six quadrates are unfortunately unknown. Nevertheless, all dinosaur material collected near Jorf is extracted from galleries dug in the Kem Kem beds and the specimens MHNM.KK376 and.KK378 most probably come from that unit (Escuillié pers. comm.). Because MHNM.KK374,.KK375 and.KK377 were bought in local markets in Erfoud (Escuillié pers. comm.) and given the fact that the sediment adhering to them is consistent with the lithology of the Kem Kem beds in color, composition, and texture, it is also unlikely that these specimens come from another lithostratigraphic unit of North Africa. Based on the most complete study of the Kem Kem stratigraphy (i.e., Cavin et al. [5]), the Kem Kem beds are dated to the Cenomanian (and likely to the Early Cenomanian [5,62,63]) and divided into the Ifezouane and Aoufous formations, which possibly correspond to the lower and upper units of Sereno et al. [3], respectively (Fig 1B; for an overview of the geological and stratigraphical settings of the Ifezouane and Aoufous formations, see S1 File). Because the majority of fossil vertebrates come from the upper part of the Ifezouane Formation (even in the northern Kem Kem area, see S1 File), all specimens probably come from this unit ([5,7,22]; Cavin pers. comm.; Fig 1B). The Aoufous Formation has indeed yielded a very small amount of vertebrate fossils [5,22], and large vertebrate remains are much rarer than in the Ifezouane Formation (Cavin pers. comm.). In addition, the Aoufous Formation essentially includes marls and mudstones ([5,22]), and the sandstone matrix visible on the quadrates supports the fact that they were uncovered in the Ifezouane Formation. Nonetheless, given the fact that theropod remains were already found in the Upper Unit (e.g., *Carcharodontosaurus*; see [3,23]), which may correspond to the Aoufous Formation, we remain cautious about the stratigraphic distribution of the material described here and only ascribe with confidence the two quadrates found near Jorf to the Kem Kem beds. The other quadrates are, therefore, tentatively assigned to this unit.

### Ethic statement

Although Moroccan collectors and their activities are protected by Moroccan law and the Ministère des Mines, de l’Eau et de l’Environnement [64], permits from the Ministère du Commerce Extérieur (with approval from the Division du Patrimoine Géologique, the Direction du Développement Minier, and/or the Ministère de l’Energie, des Mines, de l’Eau et de l’Environnement) are required when collecting and exporting Moroccan fossils abroad [65,66]. The specimens, of which five of them (i.e., MHNM.KK374 to.KK378) were bought from a licensed seller in Erfoud (Escuillié pers. comm.), were, however, exported before any of these steps could be taken by the authors so that the material was studied in Europe after being exported. The authors were not involved in the exporting or purchasing of fossils, despite accessing them before they were permanently deposited. Agreements with the Direction de la Géologie of the Ministère des Mines, de l’Eau et de l’Environnement and the Muséum d’Histoire Naturelle of Marrakech were later acquired to repatriate most of the specimens (i.e., MHNM.KK374 to.KK378) to Morocco, which complied with all relevant regulations. All specimens are, therefore, permanently deposited in appropriate repositories (i.e., the Muséum d’Histoire Naturelle of Marrakech and the Museo di Storia Naturale di Milano of Milan) and accessible to other researchers, which confirms that this study adheres to the PLoS ONE guidelines for paleontology/archaeology research: http://journals.plos.org/plosone/s/submission-guidelines#loc-paleontology-and-archaeology-resea(rch.

### Anatomical Nomenclature and Phylogenetic Definitions

The description of the quadrates follows the anatomical terminology proposed by Hendrickx et al. [60] which can be summarized as follow: The quadrate is comprised of two main parts: the quadrate body posteriorly, and the pterygoid flange anteriorly. The latter projects anteriorly from the quadrate body to contact the pterygoid. The quadrate body includes the quadrate shaft, which links the quadrate head dorsally to the mandibular articulation ventrally. The quadrate foramen, which typically lies at mid-height of the quadrate body, separates the ventral quadratojugal contact from the dorsal quadratojugal contact, which faces laterally, and sometimes anteriorly or posteriorly. Two processes project laterally or anterolaterally from the lateral margin of the quadrate body, namely the lateral process and the quadratojugal process. The lateral process either extends from the laterodorsal part of the quadrate body, dorsal to the quadrate foramen, or from the whole lateral margin of the quadrate shaft, whereas the quadratojugal process always projects anteriorly from the anterior margin of the ventral quadratojugal contact. The quadrate shaft corresponds to the part of the quadrate body excluding the quadrate head, mandibular articulation, quadratojugal contacts, lateral process, and quadratojugal process. The quadrate shaft typically includes a ventrodorsally oriented ridge, or quadrate ridge, on its posteromedial side. In some cases, the quadrate shaft also encompasses a ventrodorsally elongated depression, or fossa, on the posterior side of the quadrate and known as the posterior fossa. A second depression, the medial fossa, is located on the ventromedial surface of the pterygoid flange and is bounded by the quadrate shaft posteriorly. The quadrate head can be monostylic or bistylic and divided by an intercapitular sulcus into the squamosal and otic capitula. The mandibular articulation includes, in the large majority of theropods, two condyles. The lateral condyle of the mandibular articulation, called ectocondyle, is separated from the medial condyle, or entocondyle, by the intercondylar sulcus. An anterior or posterior intercondylar notch can sometimes be seen either on the anterior or posterior surface of the intercondylar sulcus, respectively, between the two mandibular condyles. When pneumatic, the quadrate includes one or several pneumatic openings, i.e., the anterior, posterior, medial, ventral and dorsal pneumatic foramina, depending on their position on the quadrate.

The theropod phylogeny adopted here follows the classification summarized by Hendrickx et al. [67] for nonavian theropods. Likewise, we follow the phylogenetic definitions compiled by Hendrickx et al. ([67]: Table 1) for nonavian theropod clades.

**Table 1:** Measurements of five quadrates of Spinosaurinae from the Kem Kem beds of Morocco. Values are given in millimeters.

	MHNM.KK374	MHNM.KK375	MHNM.KK376	MHNM.KK377	MHNM.KK378	MSNM V6896
**1. Ventrodorsal height of the quadrate**^**a**^	78	145	113^d^	130	220	145
**2. Anteroposterior length of the quadrate**^**b**^	30	75	?	45	?	>50
**3. Anteroposterior length of the dorsal margin of the pterygoid flange**^**c**^	27	56	?	?	115	55
**4. Ventrodorsal height of the anterior margin of the pterygoid flange**	57	93	?	60	164	104
**5. Anteroposterior length of the pterygoid flange at the level of the medial fossa**^**d**^	?	30	?	?	?	?
**6. Anteroposterior length of the quadrate shaft at the level of the quadrate foramen**	10	25	25	20	30	19
**7. Lateromedial width of the mandibular articulation**	36	77	108	70	?	76
**8. Lateromedial width of ectocondyle**	28	40	57	?	?	60
**9. Ventrodorsal height of ectocondyle**	8	24	30	17	?	14
**10. Lateromedial width of entocondyle**	26	50	50	47	80	31
**11. Ventrodorsal height of entocondyle**	10	21	24	17	33	22
**12. Anteroposterior length of squamosal capitulum**	13	25	?	25	33	28
**13. Ventrodorsal height of quadrate head**	9	16	?	18	22	20
**14. Ventrodorsal height of quadrate foramen**	14	18	15	25	30	20
**15. Lateromedial width of quadrate foramen**	7	11	10	10	13	11
**16. Ventrodorsal height of the quadratojugal contacts**	46	75	90	?	?	73
**17. Ventrodorsal height of the dorsal quadratojugal contact**	18	33	43	23	40	33
**18. Anteroposterior length of the dorsal quadratojugal contact**	6	11	21	11	15	11
**19. Ventrodorsal height of the ventral quadratojugal contact**	14	33	43	?	?	22
**20. Anteroposterior length of the ventral quadratojugal contact**	12	28	24	?	?	18
**21. Lateromedial width of the ectocondyle fossa**	/	/	48	/	/	/
**22. Ventrodorsal height of the ectocondyle fossa**	/	/	27	/	/	/

^a^Distance taken from the posterior margin of the squamosal capitulum to the ventral margin of the entocondyle.

^b^Distance taken from the dorsalmost point of the dorsal quadratojugal contact to the anterior surface of the pterygoid flange.

^c^Distance taken from the apex of the anterodorsal curvature of the pterygoid flange to the apex of the anteroventral curvature.

^d^ Distance taken from the base of the mandibular condyles to the dorsal extremity of the broken shaft.

### Cladistic Analysis

A phylogenetic analysis was performed to assess the phylogenetic relationships of the quadrate bones from the Kem Kem beds, and the bones were coded in an updated version of the supermatrix of Hendrickx et al. [61]. The supermatrix encompasses 98 quadrate-related characters (S1 File) originally associated with six recent datasets (i.e., [14,68–71]) on the whole theropod skeleton coded in one outgroup and 55 non-avian theropod taxa. All quadrate-based characters were removed from the six datasets. The main changes are the inclusion, in the supermatrix, of the data matrix of Novas et al. [72] as well as four additional taxa (i.e., *Guanlong*, *Sinosauropteryx*, *Ornithomimus* as well as *Spinosaurus* coded from the newly discovered specimen FSAC-KK 11888), and the replacement of the dataset of Choiniere et al. [68] by an updated version of Choiniere et al. [73]. The final supermatrix includes 2377 characters and 59 taxa for one outgroup (*Eoraptor*; Table A in S1 File). TNT v1.1 [74] was employed to search for most-parsimonious trees (MPTs). The supermatrix was analyzed under the ‘New Technology Search’ with the ‘driven search’ option (TreeDrift, Tree Fusing, Ratchet, and Sectorial Searches selected with default parameters), and stabilizing the consensus twice with a factor of 75. The consistency and retention indices as well as the Bremer supports [75] were calculated using the ‘stats’ and ‘aquickie’ commands, respectively, and a bootstrap analysis was performed with the standard options.

### Geometric Morphometric and Phylogenetic Morphometric Analyses

**Fig 5:**
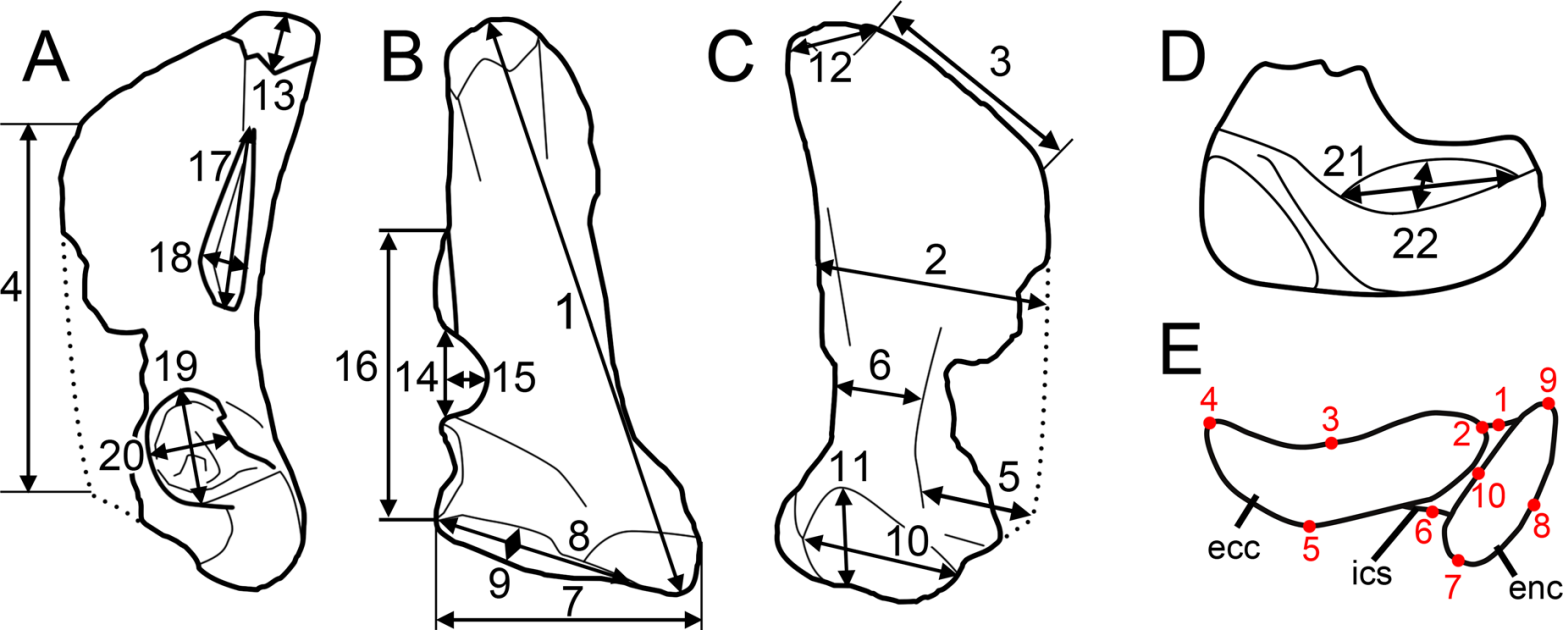
Measurements. **A**–**D**, Measurements taken on the six spinosaurine quadrates from the Kem Kem beds of Morocco in **A**, lateral; **B**, posterior, **C**, medial; and **D**, ventral views; **E**, location of the ten landmarks used in the morphometric analyses in an idealized mandibular articulation of a non-avian theropod in ventral view. **Abbreviations**: **ecc**, ectocondyle; **enc**, entocondyle, **ics**, intercondylar sulcus.

The morphological diversity of the mandibular articulation was investigated through geometric morphometric and phylogenetic morphometric analyses based on landmark configuration defined by Hendrickx et al. [61] for the quadrate in ventral view (character 2). Both morpho- and phylo-morpho analyses comprise a sample of 37 theropod taxa selected for their completeness and preservation (S1 File). Two additional landmarks were added to the eight initial landmarks proposed by Hendrickx et al. [61] to account for the orientation of the intercondylar sulcus. As a result, ten landmarks defining the outline of the mandibular articulation and the ecto- and entocondyles provide a comprehensive coverage of the ventral view of the quadrate (Fig 5E). Pictures from each taxon were sorted alphabetically and compiled using tpsUtil (Tps geometric morphometrics software is available for free download at http://life.bio.sunysb.edu/morph/soft-utility.html) and the digitization of the landmarks on the pictures was done with tpsDig2. The geometric morphometric analysis was performed with MorphoJ [76] in which the landmarks were first aligned by a Procrustes fit. A principal component analysis (PCA) was then conducted after generating a covariance matrix, and the morphospace occupation for each taxon was mapped onto phylogeny and along the two principal axes of the PCA. The resulting MorphoJ file is available in S1 File.

In order to reconstruct a phylogeny separate from landmark data alone, we performed a phylogenetic morphometric analysis using the same landmark position of the 37 theropod taxa (S1 File). The file created from the digitization of the landmarks using tpsUtil was first taken to tpsRelw where the alignment was saved by using the ‘Save aligned specimens’ option, after computing Consensus, Partial warps and Relative warps. In order to run in TNT v1.1 [74], the *.tps file was transformed into a *.tnt file using the tps2tnt software. A phylogenetic morphometric analysis was then performed on the newly created file by using the TNT script Landsch.run. To reconstruct a phylogeny using a combination of landmark data and the 2377 discrete characters of the supermatrix, we used the Landcombsch.run TNT script. This method allows to constrain all major theropod clades and see the ancestral landmark configuration of the mandibular articulation for each node. The phylogenetic searches were run considering three different levels of search thoroughness (the scripts pre-defined levels 0, 1 and 2; see [61] for more explanation). The scores of each configuration were rescaled in all analyses is such a way that the contribution of one landmark configuration character is similar to a traditional character (S1 File).

## Results

### Systematic Paleontology

Dinosauria Owen, 1842 [77]

Saurischia Seeley, 1887 [78]

Theropoda Marsh, 1881 [79]

Tetanurae Gauthier, 1986 [80]

Megalosauroidea (Fitzinger, 1843 [81]) Walker 1964 [82]

Spinosauridae Stromer, 1915 [83]

Spinosaurinae (Stromer, 1915 [83]) Sereno et al., 1998 [25]

### Description

The six isolated quadrates from the Kem Kem beds of Morocco clearly belong to two morphotypes (Figs 2–4) based on the size and outline of the quadrate foramen, shape of the mandibular articulation, and outline, surface, and orientation of the quadratojugal contacts. Measurements taken on each quadrate (Fig 5A–5D) are provided in Table 1.

*Spinosaurus* Stromer, 1915 [83]

*Spinosaurus aegyptiacus* Stromer, 1915 [83]

#### Morphotype 1

Five quadrates (MHNM.KK374 to.KK375 and.KK377 to.KK378; MSNM V6896) belonging to individuals of different ontogenetic stages are referred to a first morphotype (Fig 2C–2F). MHNM.KK374 (Fig 3A–3F) is a left quadrate of small size displaying ontogenetic features typical of immature theropods so that the bone can confidently be ascribed to a juvenile individual (a justification for the ontogenetic stages is given below). MHNM.KK375 (Fig 3G–3L), MHNM.KK377, and MSNM V6896 (Fig 3M–3R) are mid-size left quadrates of roughly similar dimensions (Table 1). Based on the excavation of the quadratojugal contacts and the morphology of the quadrate ridge, MHNM.KK375 likely belongs to a subadult individual whereas MHNM.KK377 and MSNM V6896 belong to relatively immature specimens (see below). The largest bone is MHNM.KK378, a right quadrate referred to a fully mature individual due to its particularly large size and the morphology of its mandibular condyles, quadrate head and quadrate ridge. Three of these quadrates (MHNM.KK374,.KK375; MSNM V6896) are relatively well-preserved as the anterior margin of the pterygoid flange is only missing some pieces of bones in these specimens (Fig 3). Among the two poorly preserved quadrates, MHNM.KK377 shows several anteroposteriorly oriented fractures and the ventral and dorsal halves of the bone were inaccurately glued, as the dorsal part should be rotated around 10 degrees clockwise (Fig C:A–F in S1 File). This quadrate is particularly damaged as part of the quadrate shaft, the ectocondyle and the pterygoid flange are missing. MHNM.KK378 is not deformed, yet the anterior surface is strongly damaged and the whole ectocondyle, the ventral quadratojugal contact and most of the pterygoid flange are missing (Fig C:G–L in S1 File). The quadrate shaft, the entocondyle and the dorsal quadratojugal contact are, however, well-preserved in this specimen.

In posterior view, the quadrate body of this first quadrate morphotype has a rough ‘Eiffel tower’ outline as the quadrate tapers dorsally, from a lateromedially wide mandibular articulation ventrally to a lateromedially narrow quadrate head dorsally (Fig 3C and 3O). The medial margin of the quadrate body is concave in posterior view, and straight to weakly convex at mid-height of the quadrate shaft. The lateral margin of the quadrate is straight to slightly concave along the ventral quadratojugal contact, and straight to sigmoid from the quadrate foramen to the quadrate head. The quadrate body displays a prominent and lateromedially wide, yet poorly delimited, quadrate ridge extending from the dorsal end of the entocondyle to two thirds of the bone, well beneath the quadrate head. The main axis of the quadrate ridge is inclined laterally at an angle of 110–120° from the main axis passing through the mandibular articulation. In the largest specimen (MHNM.KK378), a ventrodorsally long prominence can be seen on the dorsal third of the quadrate, ventral to the quadrate head and strongly deflected laterally (Fig C:I-J in S1 File). This prominence, which is here interpreted as a second quadrate ridge, reaches the quadrate head dorsally and may have contacted the medial surface of the squamosal laterally. This anteroposteriorly narrow convexity is also present in the smaller quadrates but not so well-delimited. The squamosal capitulum is convex and sometimes semi-circular in posterior view. A small concavity is visible directly ventral to the quadrate and was most likely in contact with the squamosal. A well-defined quadrate foramen, delimited by the ventral quadratojugal contact ventrally and the dorsal quadratojugal contact dorsally, appears on the lateral side of the quadrate, at one third of the bone height (Fig 3C, 3I and 3O). The foramen is parabolic in outline in MHNM.KK374,.KK377 and.KK378 and reniform in MHNM.KK375 and MSNM V6896 due to the presence of a well-developed ventral projection of the dorsal quadratojugal contact in these two specimens (Fig 3O). This ventral projection is absent in MHNM.KK374 (Fig 3C) and missing in MHNM.KK377 and.KK378. A shallow and lateromedially oriented groove runs from the ventral margin of the quadrate foramen to the laterodorsal margin of the ectocondyle in the smallest quadrate (MHNM.KK374; Fig 3C). This groove is poorly visible in the largest quadrate and absent in the others. The articulating surface of the two mandibular condyles is well-delimited, and delimited from the rest of the quadrate surface by a small step in mature specimens. The surface outline of the mandibular condyles is roughly oval to subtriangular. Both mandibular condyles are separated by a diagonally oriented groove so that the ventral margin of the mandibular articulation is biconvex in posterior view. The ecto- and entocondyle extend at the same level dorsally, yet the posterior surface of the ectocondyle is always more important than the entocondyle. The posterior surface of MHNM.KK375 is well-preserved and shows some pits where tendons of muscles were attached: one ventral to the quadrate foramen and medial to the ventral quadratojugal contact, a second beneath the ventral margin of the quadrate head and a third one on the dorsal surface of the pterygoid, directly medial to the quadrate head.

In medial view, the pterygoid flange expands from the dorsal margin of the quadrate head dorsally to the anterior extremity of the entocondyle ventrally (Fig 3D, 3J and 3P). The flange is subtrapezoidal in outline, with an anteroposteriorly long and anteroventrally inclined dorsal margin and an anteroposteriorly short and anterodorsally inclined ventral margin. The dorsal margin is inclined ventrally at an angle of 10° to 50° from the main axis of the quadrate shaft. The anterior margin is ventrodorsally biconvex in MHNM.KK375, which preserves most of the pterygoid flange (Fig 3J), as the flange makes an angle to extend only ventrally at one fifth of its height. A deep medial fossa lays at two fifth of the bone height between the quadrate shaft and the pterygoid flange. This fossa is not pneumatic as it does not lead to any internal pneumatic chamber within the quadrate (Fig 3J). The depression formed by the medial fossa extends adjacently to the quadrate ridge along two thirds of the flange. The posterior margin of the shaft is strongly concave and almost straight in the largest specimen. The entocondyle is globular, D-shaped and posteroventrally oriented.

In anterior view, the pterygoid flange covers five sixths of the bone and its anterior surface curves medially (Fig 3A, 3G and 3M). The flange terminates dorsally by a small subtriangular concavity anterior to the quadrate head in MHNM.KK375 (Fig 3G). The dorsal two-thirds of the flange are ventrodorsally oriented, whereas the ventral third curves postero-medially to reach the entocondyle. The medial margin of the pterygoid flange was most likely biconvex in anterior view, with a short subtriangular convexity at one-third of the bone height. The ventral margin of the mandibular articulation is biconcave and the ectocondyle covers three fourths of the mandibular articulation in anterior view. This lateral condyle is strongly lateromedially elongated and its ventral margin is sigmoid. A deep yet poorly delimited concavity is seen on the anterior surface of the ectocondyle, medial to the ventral quadratojugal contact (Fig 3A, 3G and 3M). The articulating surface of the entocondyle only forms a small subtriangular surface in anterior view. A lateromedially oriented groove is visible dorsal to the entocondyle and ventral to the pterygoid flange in MHNM.KK374 (Fig 3A). This groove, which is not present in other specimens, extends to the anterior depression of the ectocondyle in this specimen.

In lateral view, the two quadratojugal contacts are well-delimited and separated by the quadrate foramen (Fig 3B, 3H and 3N). The ventral quadratojugal contact is always anteroposteriorly longer than the dorsal contact in this quadrate morphotype. It has an oval and a reversed D-shaped outline in MHNM.KK374 and MHNM.KK375, respectively (Fig 3B and 3H). In mature specimens, the ventral quadratojugal contact is deeply excavated by several grooves and deep depressions, suggesting a strong and immovable contact between the quadrate and quadratojugal (Fig 3H). The ventral quadratojugal contact is incomplete in MHNM.KK377 and totally missing in MHNM.KK378. The dorsal quadratojugal contact of the quadrate is ventrodorsally elongated and has a lanceolate outline in lateral view. The lateral surface of the dorsal quadratojugal contact is flattened and faces posteriorly in the smallest specimen MHNM.KK374 (Fig 3B). There are two longitudinal grooves on this surface in MHNM.KK375 and MHNM.KK377, which appears to be the condition in the quadrate of mature specimens belonging to Morphotype 1 (Fig 3H). A flattened surface with a reverse tear-drop outline extends from the dorsal extremity of the dorsal quadratojugal contact ventrally, to the quadrate head dorsally. This surface is bounded by the dorsal quadrate ridge in MHNM.KK378, and most likely received the squamosal. Both the anterior and posterior surfaces of the ectocondyle are convex in lateral view, and the lateral mandibular condyle bows anteriorly from the ventral quadratojugal contact to the ventral extremity of the pterygoid flange. The quadrate head is prominent in mature specimens MHNM.KK375 and MHNM.KK378 (Fig 3H). The anteroposterior length of the quadrate head varies in quadrates of Morphotype 1, being short in MHNM.KK375 and long in MHNM.KK378 and MSNM V6896. This is also the case with the quadrate head outline, which is weakly convex in the immature specimens MHNM.KK374,.KK376, and MSNM V6896, and subconical in the largest quadrate MHNM.KK378.

In dorsal view, the quadrate head is diamond-shaped in MSNM V6896 and oval to subcircular in all other specimens (Fig 3E, 3K and 3Q). In MHNM.KK375 and MSNM V6896, the pterygoid flange extends anteriorly and bends anteromedially in its anteriormost part (Fig 3Q). In MHNM.KK374,.KK377, and.KK378, the pterygoid flange remains straight and only projects anteriorly in its dorsal part (Fig 3E). The pterygoid flange tapers anteriorly so that it has the same thickness than the quadrate head posteriorly and gets thinner to form a sheet-like structure more anteriorly (Fig 3Q). The pterygoid flange is, however, relatively thick anteriorly in the largest specimen. The quadrate ridge is an anteroposteriorly compressed cylinder at the level of the medial fossa.

In ventral view, the mandibular condyles are strongly asymmetrical (Fig 3L and 3R). The entocondyle is oval to oblong in outline and its main axis is anteromedially oriented. The ectocondyle, on the other hand, is helicoidal and strongly lateromedially elongated, so that the lateral condyle covers most of the anterior surface of the mandibular articulation, from the ventral quadratojugal contact to the anterior extremity of the entocondyle (Fig 3L). The thickness of the ectocondyle diminishes laterally, and a weak concavity is visible on the anterior surface of the condyle. The intercondylar sulcus separating the two mandibular condyles is straight and poorly delimited in immature specimens, and well-visible and sigmoid in more mature individuals. It is particularly deep in the subadult specimens MHNM.KK375 and.KK378 where the entocondyle is well-demarcated. The main axis of the intercondylar sulcus is lateromedially oriented in all specimens, and forms an angle of 130–140° with the main axis of the mandibular articulation. In MHNM.KK374, the two condyles are not easily distinguishable as the intercondylar sulcus separating them is almost absent (Fig 3F). In this juvenile specimen, the mandibular condyles are not prominent and the posterior margin of the mandibular articulation is roughly convex. On the other hand, the posterior margin of the mandibular articulation is biconvex in more mature specimens. The ventral quadratojugal contact projects anteriorly in the best preserved specimen (MHNM.KK375), and this anterior projection is absent in MHNM.KK374 and most likely missing in MHNM.KK377 and MSNM V6896.

?*Sigilmassasaurus* Russel, 1996 [2]

?*Sigilmassasaurus brevicollis* Russel, 1996 [2]

#### Morphotype 2

The ventral portion of a right quadrate (MHNM.KK376; Fig 3) shows some important morphological variations in comparison to the five other quadrates, namely a minute quadrate foramen, both ventral and dorsal quadratojugal contacts of similar anteroposterior length, a dorsal quadratojugal contact excavated by a deep depression, a trapezoidal ventral quadratojugal contact with a flat lateral margin strongly inclined medially, a deep and well-defined depression on the anterior surface of the ectocondyle, and a lateromedially wider and anteroposteriorly shorter ectocondyle (Fig 2G–2J). The dorsal part of MHNM.KK376 is missing above the dorsal end of the dorsal quadratojugal contact, and the preserved portion corresponds to half of the bone in the quadrates of Morphotype 1. The pterygoid flange is also almost entirely missing, yet its posteriormost part is visible (Fig 4A). Both quadratojugal contacts and mandibular condyles are well-preserved although a small portion of the ectocondyle, on the latero-ventral margin of the condyle, was restored.

In posterior view, the quadrate shaft is inclined laterally at an angle of around 30° with the main axis passing through the mandibular articulation (Fig 4C). The ridge is massive and its medial margin is concave ventrally and weakly convex at mid-height of the quadrate. The quadrate ridge is slightly constricted at the level of the quadrate foramen, and its thickness gently increases more dorsally. Unlike quadrates of Morphotype 1, the lateral margins of the quadratojugal contacts are not aligned on the same vertical plane. The lateral surface of the ventral quadratojugal contact is dorsomedially inclined whereas the dorsal quadratojugal contact is weakly laterodorsally inclined (Fig 4C). The surface of the ventral and dorsal quadratojugal contacts is roughly straight and the dorsal quadratojugal contact shows a short ventral projection as in Morphotype 1. The quadrate foramen is significantly ventrodorsally shorter and lateromedially narrower than that of Morphotype 1. When the quadrate was in articulation with the quadratojugal, the outline of the quadrate foramen was most likely a reversed tear-drop shape. The ento- and the ectocondyle are separated ventrally by an intercondylar sulcus formed by a lateromedially narrow and ventrodorsally tall concavity. The articulating surface of the ectocondyle is elliptical in outline, lateromedially wider than the entocondyle and extends slightly more dorsally than the medial condyle (Fig 4C). The articulating surface of the latter is oval to D-shaped in outline in posterior view. There is no step delimiting the articulating surface of the mandibular condyles from the rest of the quadrate body. A diagonally oriented groove extends from the ventral margin of the quadrate foramen laterally to the level of the intercondylar sulcus (Fig 4C). This groove is homologous with that seen in MHNM.KK374.

In medial view, the anteroposterior length of the quadrate ridge remains relatively constant along its ventrodorsal height (Fig 4D). The preserved portion of the pterygoid flange projects anteriorly and its ventralmost part reaches the entocondyle ventrally. There is a medial fossa situated between the quadrate shaft and the pterygoid flange. This depression is deep, yet it does not lead to a pneumatic chamber. The entocondyle protrudes ventrally and the articulating surface of the entocondyle is roughly D-shaped in outline in medial view.

In lateral view, the two quadratojugal contacts of MHNM.KK376 are well-delimited and their morphology strongly differs from that of Morphotype 1. Both ventral and dorsal quadratojugal contacts share the same anteroposterior length in their longest part (Fig 4B). The dorsal quadratojugal contact is incomplete and its remaining portion is excavated by a deep depression dorsally and two ventrodorsally oriented grooves converging ventrally in its ventral part (Fig 4B). The ventral quadratojugal contact, on the other hand, is fully preserved and its general outline is subtrapezoidal instead of D-shaped as in Morphotype 1 (Fig 2). It is slightly anteriorly deflected from the dorsal quadratojugal contact. The ventral quadratojugal contact gently tapers dorsally and its lateral surface is irregular and excavated by several foramina and irregular furrows. A deeper groove is also visible adjacent to the posterior margin of the ventral quadratojugal contact. The ectocondyle is anteroposteriorly short and weakly oriented posteroventrally. The ventral quadratojugal contact does not extend on the whole surface of the ectocondyle.

In anterior view, the preserved portion of the pterygoid flange is centrally positioned on the quadrate body and follows the orientation of the quadrate ridge dorsal to the ventral quadratojugal contact (Fig 4A). The pterygoid flange curves ventromedially at the level of the dorsalmost part of the ventral quadratojugal contact to reach the entocondyle ventrally. The anteromedial orientation of the posteriormost part of the pterygoid flange suggests that the latter mostly extended anteromedially. The ectocondyle is much wider lateromedially than the entocondyle as it occupies more than three fourths of the mandibular articulation. A deep and well-delimited depression is seen on the anterolateral surface of the ectocondyle (Fig 4A). The dorsal margin of this depression, which marks the dorsal limit of the ectocondyle, is convex and extends laterally directly ventral to the ventral quadratojugal contact. The medial part of the ectocondyle corresponds to a lateromedially elongated surface with parallel dorsal and ventral margins. The entocondyle, which is separated from the ectocondyle by the intercondylar sulcus in its ventral part, is roughly D-shaped. A shallow furrow parallel and adjacent to the dorsal margin of the ectocondyle runs along the dorsomedial part of the ectocondyle.

In dorsal view, the cross-section outline of the quadrate shaft is D-shaped, with the convexity oriented posteromedially (Fig 4G). This transverse section reveals the presence of a small hole within the quadrate, suggesting that at least a portion of the quadrate was hollow and may have included a small pneumatic chamber. The pterygoid flange projects anteromedially from the anterior surface of the quadrate body, which faces anterolaterally.

In ventral view, the mandibular condyles are strongly asymmetrical, with a much wider ectocondyle (Fig 4E). The entocondyle is oblong in outline and its main axis is lateroposteriorly oriented. The anterior surface of the entocondyle is flat whereas its posterior margin is convex. The ectocondyle is antero-posteriorly narrow, strongly lateromedially elongated, and less prominent than the entocondyle. It is helicoidal in shape and covers the whole surface of the mandibular articulation, from the ventral quadratojugal contact to the anteromedial extremity of the entocondyle. The main axis of the ectocondyle is lateromedially oriented and the ectocondyle corresponds to a low ridge in the medial half of the mandibular articulation. The anterior margin of the ectocondyle is biconvex, with the lateral convexity marking the limit of the anterior depression of the ectocondyle (Fig 4E). This deep fossa excavates the anterolateral surface of the ectocondyle so that the posterolateral part of the ectocondyle corresponds to a prominent ridge. The intercondylar sulcus is lateromedially wide in its posterior part, shallow, and tapers anteromedially. Its main axis is lateromedially oriented and inclined at an angle of 148° from the main axis of the mandibular articulation (Fig 4F).

### Cladistic Analysis

**Fig 6:**
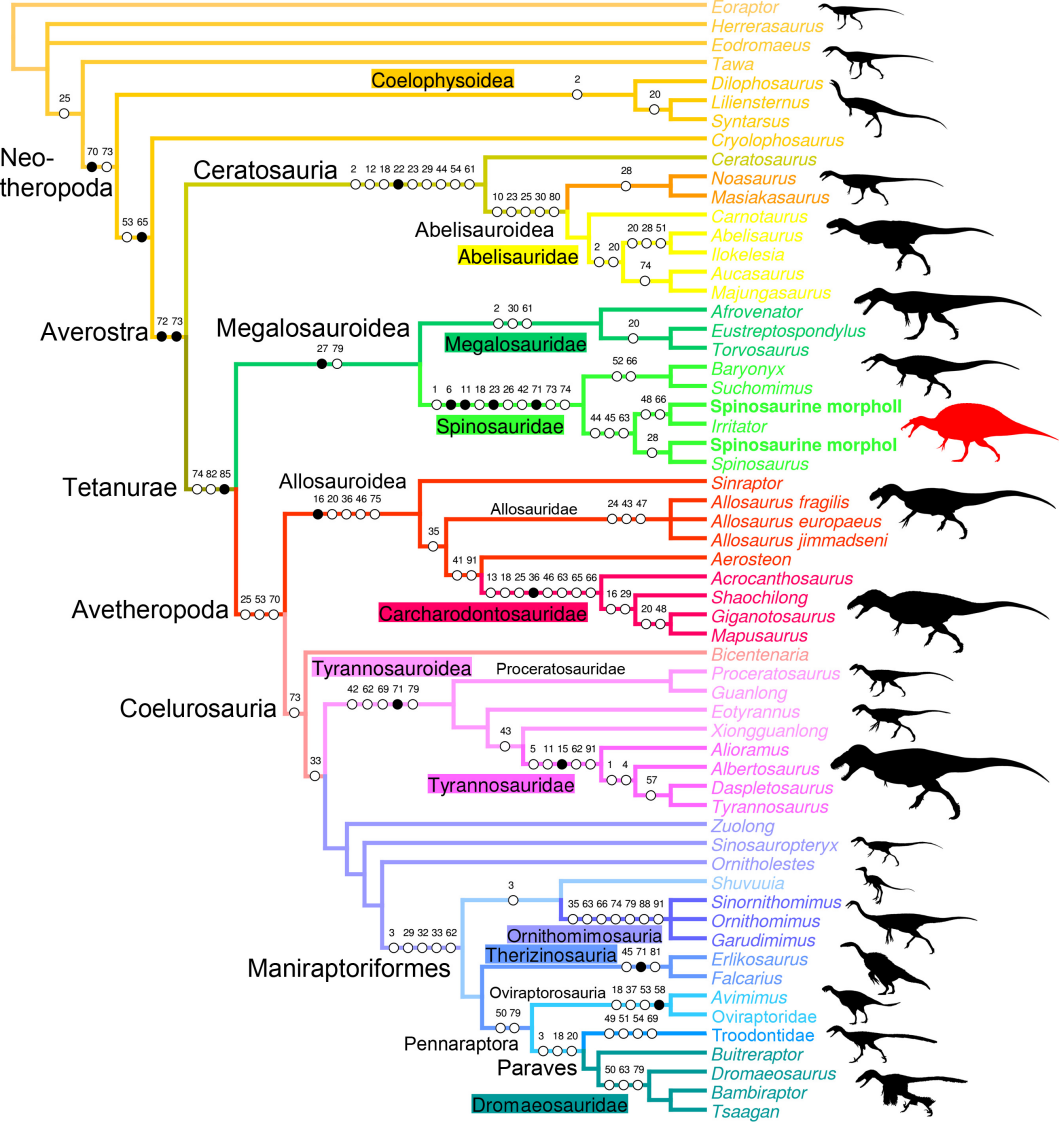
Quadrate-based phylogeny of non-avian theropods. Strict consensus cladogram from most parsimonious trees after the a posteriori deletion of *Monolophosaurus*. Initial analysis was a New Technology Search using TNT v.1.1 of a supermatrix comprising 98 quadrate-based characters combined with seven recent datasets (i.e., [14,69–71,73,132]) based on the whole skeleton, for one outgroup (*Eoraptor lunensis*) and 18 non-avian theropod taxa. Tree length = 4994; CI = 0.522, RI = 0.611. Dinosaur silhouettes by Scott Hartman (all but Coelophysoidea, *Shuvuuia* and Therizinosauria) and Funkmonk (Coelophysoidea, *Shuvuuia*, Therizinosauria) from Phylopic, used with permission.

The full phylogenetic analysis produced 16 most parsimonious trees (MPTs) of length 5049, consistency index (CI) 0.485 and retention index (RI) 0.55. The strict consensus tree is relatively unresolved as an important polytomy occurs among Neotheropoda. Yet, Ceratosauria, Megalosauroidea, and Coelurosauria are resolved clades, and the two quadrate morphotypes are recovered among spinosaurine Spinosauridae. This lack of resolution is due to the instability of *Monolophosaurus*, and a reduced consensus approach [84] was used to calculate a consensus tree excluding this taxon. The new cladistic analysis yielded 17 MPTs (length 4994, CI of 0.522 and RI of 0.611) and produced a much better resolved consensus tree mirroring to a large degree the current consensus classification of non-avian theropods (Fig 6). Both morphotypes are still recovered among Spinosaurinae which is supported by three ambiguous synapomorphies: a smooth lateral surface of the dorsal quadratojugal contact (char. 44:0), the presence of anterior and posterior margins delimiting this dorsal quadratojugal contact (char. 45:2), and the ventral position of the quadrate foramen, beneath the mid-height of the quadrate body, on the lateral surface of the quadrate (char. 63:0). While the first morphotype is recovered with *Spinosaurus aegyptiacus* among Spinosaurinae based on a single ambiguous synapomorphy (i.e., the important extension of the articular surface of the ectocondyle on the posterior surface of the quadrate body; char. 28:2), the second morphotype is closely related to *Irritator challengeri*. Among non-avian theropods, the clade Spinosauridae is the best resolved in terms of quadrate-related characters, with four unambiguous and six ambiguous synapomorphies constraining it (Fig 6). With eight quadrate-related synapomorphies, Carcharodontosauridae is the second best resolved theropod clade. Ceratosauria and Ornithomimosauria (6 synapomorphies), and Tyrannosauroidea, Tyrannosauridae, Maniraptoriformes and Troodontidae (5 synapomorphies) are also well-diagnosed clades in term of quadrate-related features.

### Geometric Morphometric Analysis

**Fig 7:**
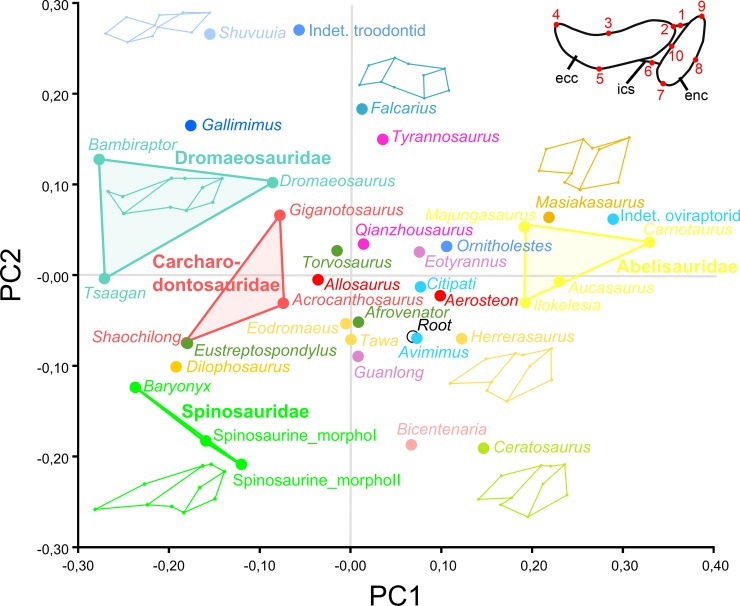
Results of the geometric morphometric analysis performed on the mandibular articulation of non-avian theropods. PCA plot of the principal component analysis performed on 37 theropod taxa and 10 landmarks along the first two principal axes explaining 35.8% and 20.04% of the variation in the sample. Colors refer to theropod clades and correspond to those in Fig 6. Major groupings at family level are delimited and outline images are associated with taxa of hypothetical extremes.

The first two main axes of the principal component analysis (PCA) performed on 37 theropod taxa and 10 landmarks, explained 35.8% and 20.04% of the variation in the sample, respectively (Fig 7). The first principal axis PC1 accounts for the lateromedial elongation of the mandibular articulation whereas the second one PC2 captures the anteroposterior thickness of this articulation. With their typical and relatively similar mandibular articulations, abelisaurid, carcharodontosaurid, and dromaeosaurid taxa are relatively closely distributed and Abelisauridae, Carcharodontosauridae and Dromaeosauridae each occupy a unique region of the morphospace (Fig 7). On the other hand, the morphospace occupation of tyrannosauroid and oviraptorid taxa is particularly important as the morphology of the mandibular articulation of the most basal taxon significantly differs from that of the derived members, in both clades. With their strongly elongated, yet anteroposteriorly broad ectocondyle associated with their oblong entocondyle, the two morphotypes are closely distributed and cluster away from other theropods, with *Baryonyx* as the closest taxon in the morphospace. Likewise, with a lateromedially short mandibular articulation including two subcircular ecto- and entocondyles, *Masiakasaurus*, abelisaurids, and an indeterminate oviraptorid cluster together. The most primitive theropods *Herrerasaurus*, *Eodromaeus* and *Tawa* also occupy very close positions, near the root of the morphospace (Fig 7). Other distantly related taxa, such as the megalosaurid *Afrovenator*, the tyrannosauroid *Guanlong*, and the basal oviraptorid *Avimimus*, also cluster with basalmost theropods.

### Phylogenetic Morphometric Analysis

**Fig 8:**
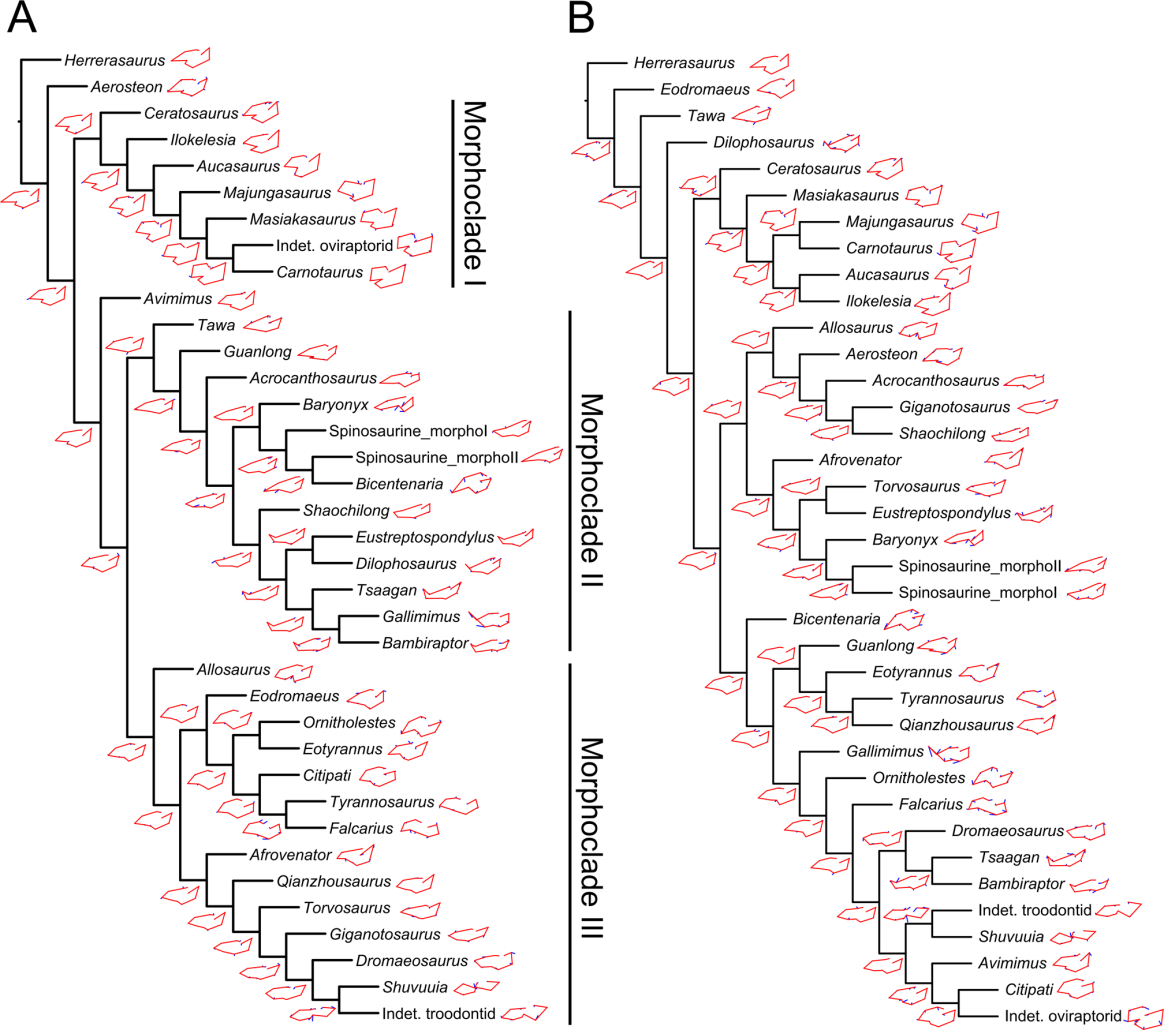
Results of the phylogenetic morphometric analysis. **A**–**B**, Phylogenetic morphometric analysis of the mandibular articulation of 36 non-avian taxa performed with a degree of thoroughness of one, and using **A**, 10 landmarks on the quadrate in ventral view (Tree score = 5.18); and **B**, combination of the phylogenetic morphometric character based on 10 landmarks of the mandibular articulation in ventral view and 2377 discrete characters from the supermatrix (Tree score = 6.61).

The phylogenetic morphometric analysis based on landmark data alone yielded a single tree which, for each different degree of thoroughness, poorly mirrors the current classification of non-avian theropods. Yet, several closely related taxa such as ceratosaurs were recovered in the same grouping (or morphoclade as the grouping results from a cladistic analysis solely based on landmark data) in the analysis performed with a degree of thoroughness of one and above (Fig 8A). The two morphotypes are closely related to *Baryonyx* in the analyses performed with a degree of thoroughness of zero and one (Fig 8A). In the trees obtained with a level of thoroughness of one and two, three morphoclades, associated with three morphotypes of the mandibular articulation, emerged. A first morphoclade consists essentially of ceratosaurs, and includes the indeterminate oviraptorid IGM A ([85]). This first morphotype is defined by a mandibular articulation with two anteroposteriorly wide condyles in which the entocondyle is larger than the ectocondyle. A second morphoclade encompasses theropods with a lateromedially elongated and parabolic to sigmoid ectocondyle, and a smaller and anteromedially oriented entocondyle. This morphoclade includes Spinosauridae, and a mixture of dilophosaurid, basal tyrannosauroid, carcharodontosaurid and dromaeosaurid taxa. Finally, a third morphoclade gathers some megalosaurid, tyrannosaurid, alvarezsaurid, therizinosaurid, and troodontid taxa. This grouping is characterized by two mandibular condyles of equal sizes and relatively similar orientation, and by ecto- and ento- condyles either anteromedially inclined, or extending parallel to the long axis of the mandibular articulation.

The phylogenetic analysis combining discrete characters and landmarks resulted in a single tree mirroring to a much better degree the current classification of theropods. Once again, the two morphotypes were placed among the Spinosauridae clade (n.b., we here refer to clade and not morphoclade as the cladistic analysis is now based on both discrete characters and landmark data), along with *Baryonyx* (Fig 8B). Most theropod clades were found resolved, yet a ‘carnosaur’ clade (sensu Rauhut [86]) including Megalosauroidea and Allosauroidea was recovered, and the alvarezsaurid *Shuvuuia* and troodontid *Saurornithoides* together form the sister clade of Oviraptorosauria. Likewise, *Afrovenator* is excluded from the clade of Megalosauridae formed by *Torvosaurus* and *Eustreptospondylus*. The most important landmark migrations from an ancestral landmark configuration of the mandibular articulation occur in the dilophosaurid *Dilophosaurus*, the spinosaurid *Baryonyx*, the ornithomimid *Gallimimus*, the basal coelurosaur *Bicentenaria*, the therizinosauroid *Falcarius*, and the indeterminate oviraptorid. This is, however, due to the absence, in our dataset, of closely related taxa for *Dilophosaurus* (no coelophysoids), *Baryonyx* (no basal spinosaurid), and *Gallimimus* (no basal ornithomimosaur), and the peculiar morphology of the mandibular articulation in the basal coelurosaur *Bicentenaria* and the basal therizinosaur *Falcarius*.

## Discussion

### Systematics

Based on the cladistic, geometric morphometric, and phylogenetic morphometric analyses, the six isolated quadrates can confidently be assigned to Spinosauridae. Both morphotypes clearly share a combination of features only seen in this clade. The quadrate is short (ambiguous synapomorphy of Spinosauridae; char. 1:2) and show a thick and cylindrical quadrate ridge which forms a prominent shaft (unambiguous syn.; char. 11:2). The quadrate ridge bounds a deep medial fossa on the ventromedial part of the pterygoid flange (char. 86:1). The dorsal quadratojugal contact is lanceolate in outline (ambiguous syn.; char. 42:1) and shows a ventral projection (char. 18:3), and the quadrate foramen is ventrodorsally elongated (char. 65:1) and mostly delimited by the quadrate (char. 62:1). The pterygoid flange is subrectangular in outline (unambiguous syn.; char. 71:1) and mostly projects anteriorly. Its ventral portion curves ventromedially, slightly above the mandibular articulation, and reaches the entocondyle ventrally (ambiguous syn.; char. 74:2). The mandibular articulation is lateromedially broad and anteroposteriorly narrow (ambiguous syn.; char. 18:3), and the ectocondyle is sigmoid, much longer than the entocondyle (unambiguous syn.; char. 23:3), and shows a concavity on its anterior surface (ambiguous syn.; char. 26:3). This combination of features is observed in the quadrate of Baryonychinae and absent in all other dinosaur clades ([61]; pers. obs.).

Similar to megalosauroids (other than *Irritator*), tyrannosaurids, some allosauroids, oviraptorids, and troodontids, the quadrate of both morphotypes lacks a lateral process. Such a process is present in non-neotheropod theropods, coelophysoids, ceratosaurs, basal Maniraptora, alvarezsauroids, therizinosauroids, and dromaeosaurids [61]. Likewise, a quadrate foramen is developed as a distinct opening between the quadrate and quadratojugal, and is mostly delimited by the quadrate. This condition contrasts with the absence of a quadrate foramen in megalosaurids and ceratosaurs (Ceratosauridae + Abelisauroidea), and with the quadrate foramen of carcharodontosaurids and dromaeosaurids, which is equally delimited by the quadrate and quadratojugal. It also differs from the very large quadrate fenestra of alvarezsauroids and deinonychosaurs. A mandibular articulation with a sigmoid and strongly elongated ectocondyle much longer than the entocondyle differs from that of ceratosaurids, tyrannosaurids, oviraptorids, alvarezsauroids, therizinosauroids, and troodontids in which the mandibular condyles are subequal in size, and that of abelisauroids in which the ectocondyle is ovoid [61]. Finally, given the absence of externally expressed pneumatic foramina, these six quadrates differ from the pneumatic quadrate of carcharodontosaurids, tyrannosaurids, ornithomimosaurs, therizinosauroids, and some compsognathids, oviraptorids, dromaeosaurids, and troodontids [60].

**Fig 9:**
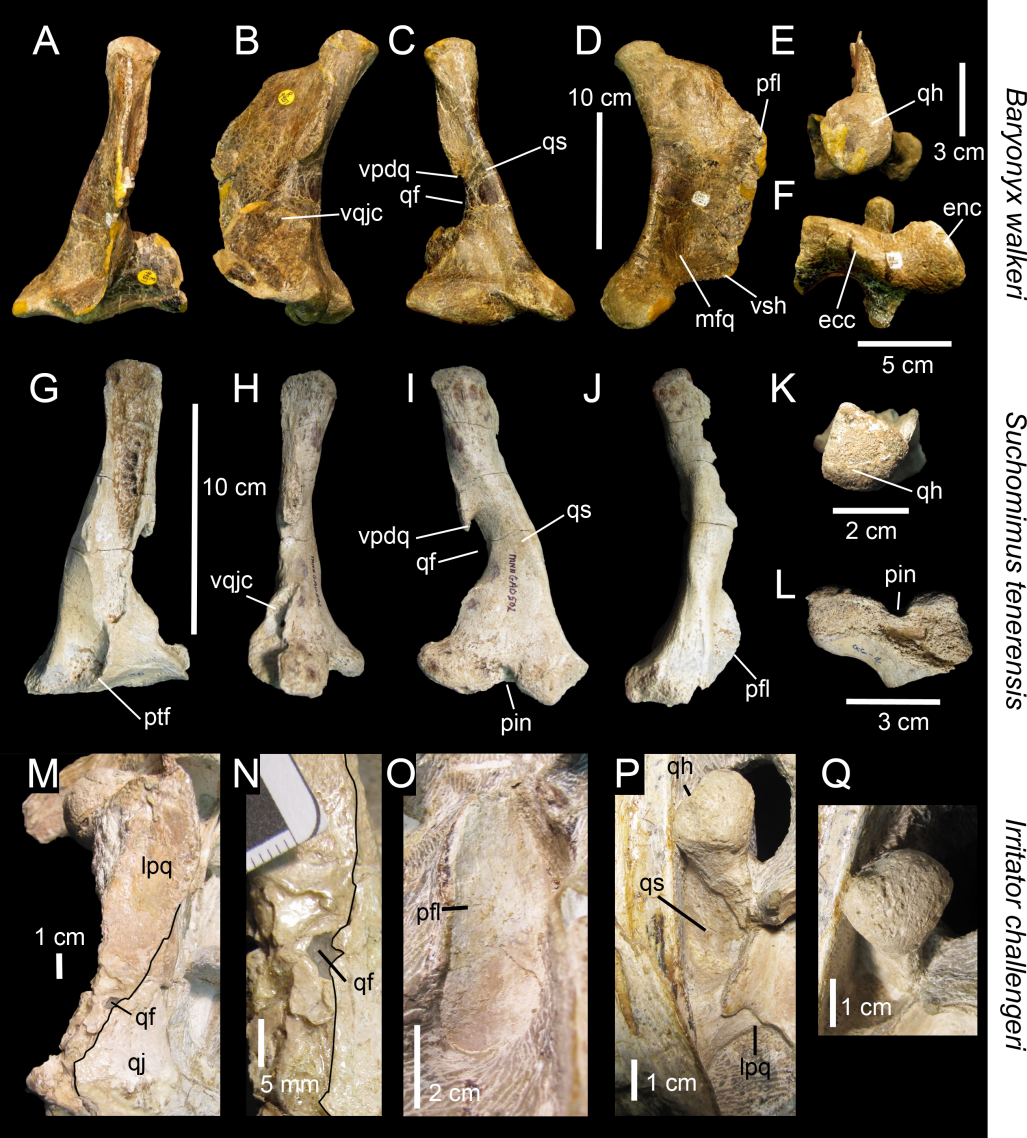
Quadrate morphology in Baryonychinae and *Irritator*. **A**–**L**, Left quadrates of **A–F**, *Baryonyx walkeri* (NHM R9951); and **G–L**, *Suchomimus tenerensis* (MNN GAD 502) in **A, G**, anterior; **B, H**, lateral; **C, I**, posterior; **D, J**, medial; **E, K**, dorsal; and **F, L**, ventral views. **M**–**N**, Right and **O**–**Q**, left quadrates of *Irritator challengeri* (SMNS 58022) with **M**, close up on the lateral portion of the quadrate body; **N**, quadrate foramen; **O**, anteromedial surface of the pterygoid flange; and **P**–**Q**, quadrate head in **M**–**N, P**, posterolateral, **O**, anterior; **Q**, and dorsal views. **Abbreviations**: **ecc**, ectocondyle; **enc**, entocondyle; **lpq**, lateral process; **mfq**, medial fossa; **pfl**, pterygoid flange; **pin**, posterior intercondylar notch; **qf**, quadrate foramen; **qh**, quadrate head; **qjp**, quadratojugal process; **qr**, quadrate ridge; **qs**, quadrate shaft; **vpdq**, ventral projection of the dorsal quadratojugal contact; **vsh**, ventral shelf of the pterygoid flange.

A subrectangular pterygoid flange with a ventral part curving medially and reaching the entocondyle, associated with a prominent and thick quadrate ridge has in fact only been identified in *Baryonyx* and *Suchomimus* [61]. Nevertheless, quadrates belonging to Morphotypes 1 and 2 differ from those of Baryonychinae by a relatively small quadrate foramen situated at one third of the quadrate height (ambiguous syn. of Spinosaurinae; char. 63:0), as well as a cylindrical quadrate ridge and an oblong entocondyle. Baryonychine quadrates possess a large and strongly ventrodorsally elongated quadrate foramen (ambiguous syn. of Baryonychinae; char. 66) at one half of the quadrate height. Likewise, the posteromedial surface of the quadrate ridge is slightly acute rather than rounded, and the entocondyle is subtriangular and shallowly delimited at least in *Baryonyx*. Quadrates of both morphotypes also differ from *Baryonyx* quadrates by a smaller ventral quadratojugal contact in which the posterodorsal part only faces laterally and not lateroposteriorly (Fig 9A–9F). They can also be distinguished from the *Suchomimus* quadrate by the absence of a subtriangular projection of the dorsal margin of the ventral quadratojugal contact (Fig 9G–9L), and an elevated rim along the dorsal and posterior margin of the ventral quadratojugal contact.

Quadrates of Spinosaurinae are known in *Irritator challengeri* [87] and *Spinosaurus aegyptiacus* [22], and although *Irritator* quadrates are incomplete or obscured by matrix, important information on quadrate anatomy can be extracted from this taxon (SMNS 58022; Fig 9M–9Q). The left quadrate is partially visible, with most of the quadrate body and pterygoid flange obscured by matrix [87], and only the quadrate head and the anterodorsal extremity of the pterygoid flange of this quadrate are visible (Fig 9O–9Q). The posterior part of the dorsal process of the right quadratojugal, which faces posterolaterally and is separated from the rest of the quadratojugal by an acute lateral ridge [87], is here interpreted as the lateral portion of the quadrate body of the right quadrate (Fig 9M). If this interpretation is correct, the right quadrate of *Irritator* shows a short laterally projected lateral process lateral to the quadrate ridge and quadrate head, a feature visible in the left quadrate as well in posterodorsal view (Fig 9P). This condition is, however, absent in both morphotypes in which the prominent and cylindrical quadrate shaft is adjacent to the quadratojugal contacts. A minute quadrate foramen seems also to be present in *Irritator* (Fig 9N) and contrasts with the much larger quadrate foramen of Morphotype 1. The quadrate head of *Irritator* has a rounded triangular to subrectangular outline in dorsal view ([87]; Fig 9Q) and differs from the subcircular squamosal capitulum of MHNM.KK374,.KK375,.KK377, and.KK378 (Fig 3E and 3K; Fig C:E, K in S1 File), and the diamond-shaped quadrate head of MSNM V6896 (Fig 3Q). Morphotype 2 also differs from *Irritator* by a medially inclined ventral quadratojugal contact in posterior view. Yet, due to the very small quadrate foramen and the straight surface of the ventral quadratojugal contact in posterior view, the second spinosaurine morphotype is morphologically closer to *Irritator*.

The holotype specimen of *S*. *aegyptiacus* did not preserve any quadrate [83], but the situation has changed with the erection of a neotype for this species (FSAC-KK 11888) based on newly discovered material from the Kem Kem beds which includes the left and right quadrates FSAC-KK 11888 [22]. The latter were only mentioned by Ibrahim et al. [22] in the supplementary material, along with the quadrate MSNM V6896 here described and referred to a subadult individual of *S*. *aegyptiacus* by Ibrahim et al. [22]. Recently, the taxonomic identification of the material reported by Ibrahim et al. [22] has been questioned by Evers et al. [27] who notice that osteological evidence showing unambiguously that the neotype material pertains to the same species as Stromer’s *Spinosaurus aegyptiacus* is lacking. For the purpose of this paper, we accept the taxonomic assignment of Ibrahim et al. [22], and view the quadrates of FSAC-KK 11888 as representing the morphology of *Spinosaurus aegyptiacus*. Photos of the two quadrate specimens FSAC-KK 11888 were kindly provided by Nizar Ibrahim shortly before the final submission of this study, allowing us to compare the isolated quadrates from the Kem Kem beds with those of *S*. *aegyptiacus*, and to include this taxon in our cladistic analysis. Given the fact that the quadrates of the *Spinosaurus* neotype will be illustrated and thoroughly described in a future publication (Ibrahim, pers. comm.), this study will only focus on the main anatomical similarities and difference observed between FSAC-KK 11888 and the quadrates in this study.

Although incomplete, the two quadrates of *Spinosaurus aegyptiacus* share many features with the six isolated quadrates from the Kem Kem beds, confirming their spinosaurine status. Indeed, similar to our six quadrates, the two quadrate specimens FSAC-KK 11888 display a large cylindrical quadrate ridge and a small quadrate foramen situated at one third of the quadrate body. This contrasts with the more lateromedially angular quadrate ridge and the large quadrate foramen located at mid-height of the quadrates of baryonychines. Unlike *Irritator*, the quadrates of *Spinosaurus* and morphotypes I and II share a subcircular squamosal capitulum in dorsal view, the absence of a lateral process, and the presence of a ventral projection of the dorsal quadratojugal contact. Quadrates of *Spinosaurus aegyptiacus* and Morphotype 1 are very similar and only differ by subtle morphological features likely due to ontogeny. Unlike Morphotype 2, these quadrates show a small yet not minute quadrate foramen as well as a D-shaped ventral quadratojugal contact in which the anteroposterior length is significantly longer than that of the dorsal quadratojugal contact in lateral view. In posterior view, the lateral surface of the ventral quadratojugal contact is concave and not strongly medially inclined as in Morphotype 2, and it also extends on the whole surface of the ectocondyle in lateral view. Contrary to Morphotype 2 and similar to Morphotype 1, the dorsal quadratojugal contact is drop-shaped in lateral view, with the longest anteroposterior length situated in the ventral most part of this contact. Likewise, the ectocondyle of FSAC-KK 11888 does not form a crest-like structure as in MHNM.KK376, and the concavity on the anterior surface of the ectocondyle is shallow and poorly delimited, contrasting with the deep and well-defined anterior concavity on the ectocondyle in Morphotype 2. The main differences between the quadrates of *Spinosaurus aegyptiacus* and those of Morphotype 1 mostly lie in the morphology of the dorsal quadratojugal contact. In *Spinosaurus aegyptiacus*, the dorsal quadratojugal contact protrudes laterally in anterior view and faces posterolaterally in posterior view (char. 43:2). This is due to the lateromedially wide rim-like anterior margin of the dorsal quadratojugal contact, a feature poorly developed in MHNM.KK374 (Fig 3A and 3C) and absent in all other isolated quadrates. The deep intercondylar sulcus of *Spinosaurus aegyptiacus* also extends far dorsally along the posterior surface of the quadrate, forming a posterior intercondylar notch absent in both quadrate morphotypes (but present in *Suchomimus*; char. 24:2). This feature, however, most likely results from ontogeny as an intercondylar notch was probably present on the posterior surface of the largest quadrate MHNM.KK378.

Morphotypes 1 and 2 were recovered in two separate spinosaurine clades in the phylogenetic analysis, the former being closely related to *Spinosaurus* whereas the latter forms a sister-taxon pair with *Irritator* (Fig 6). Nevertheless, the two ambiguous synapomorphies uniting Morphotype 2 and *Irritator* (i.e., a minute quadrate foramen and a straight lateral margin of the ventral quadratojugal contact in posterior view) result from our tentative interpretation of the morphology of the lateral part of the right *Irritator* quadrate, interpreted by Sues et al. [87] as being the posterior part of the quadratojugal. Consequently, based on the results of the phylogenetic analysis, and given the fact that fossils of Spinosauridae have so far been assigned to the spinosaurid taxa *Spinosaurus* and *Sigilmassasaurus*, Morphotype 1 is referred with confidence to *Spinosaurus*, and Morphotype 2 likely belongs to *Sigilmassasaurus*, a referral that will, however, be thoroughly discussed in another section below. Likewise, given the almost identical morphology of quadrates of Morphotype 1 and FSAC-KK 11888, Morphotype 1 is confidently assigned to the species *Spinosaurus aegyptiacus*, an opinion followed by Ibrahim et al. [22] for MSNM V6896.

### Ontogeny

Ontogenetic variation occurring in the spinosaurid quadrate was briefly investigated by Hendrickx and Mateus [88] based on the material examined here. Given the relative size of the specimens, the level of coossification between the quadrate and quadratojugal and the development of dorsal and mandibular condyles, it is assumed that Morphotype 1 includes quadrates belonging to juvenile, immature, sub-adult and adult individuals, so that a sequence list of ontogenetic character transformations (maturity-dependent characters) can be provided for the quadrate of *Spinosaurus aegyptiacus*. Due to the scarcity of spinosaurid cranial material, no destructive methods were employed to conduct histological analysis and to investigate growth series in the *Spinosaurus* quadrates.

#### State 1

**Fig 10:**
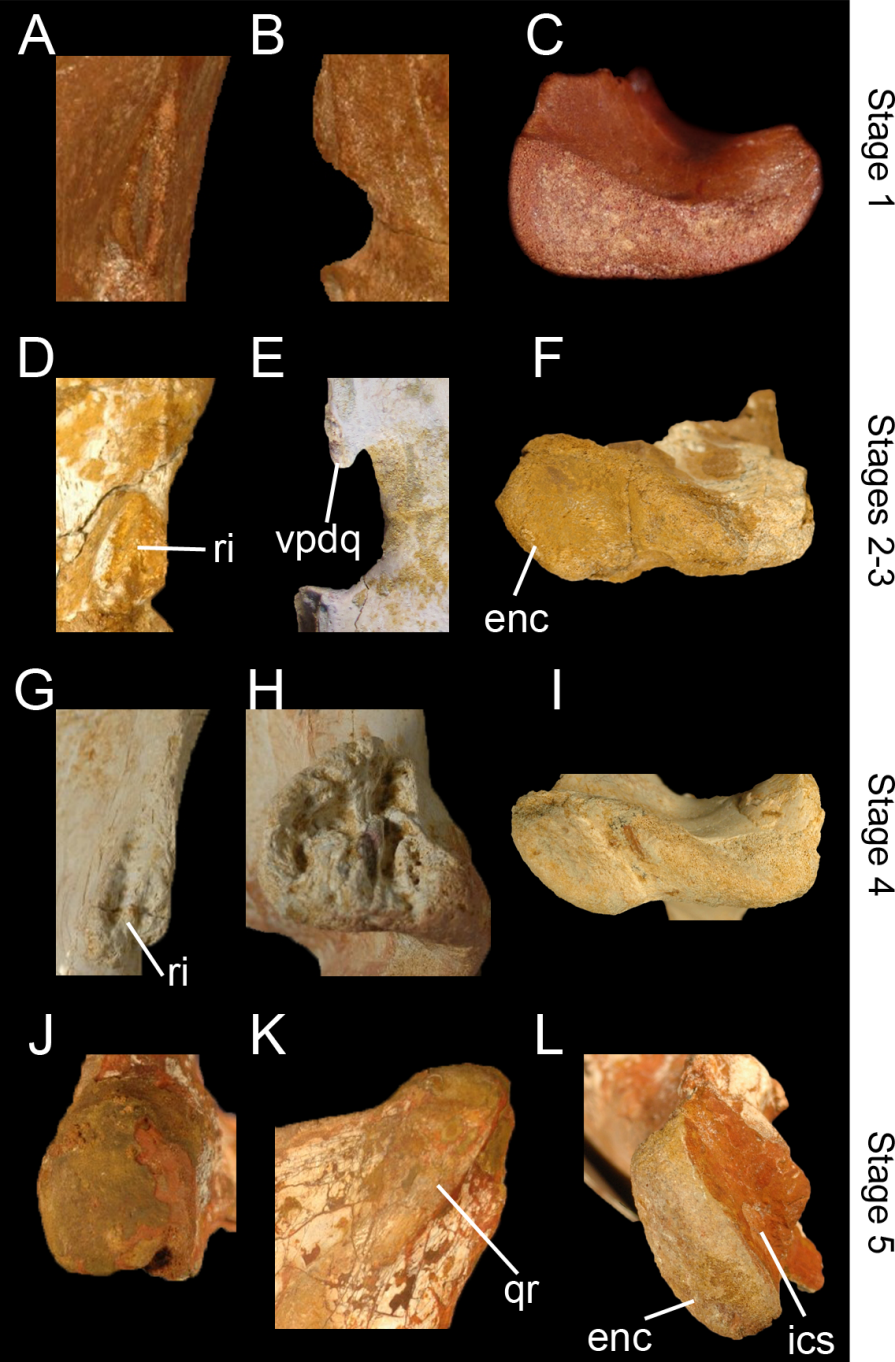
Ontogenetic changes in the quadrates of *Spinosaurus aegyptiacus* (Morphotype 1). **A**–**C**, Left quadrate MHNM.KK374 representing ontogenetic stage 1 (juvenile) with **A**, close up on the smooth lateral surface of the dorsal quadratojugal contact in lateral view; **B**, quadrate foramen and absence of a ventral projection of the dorsal quadratojugal contact in posterior view; **C**, and non-delimited mandibular condyles in ventral view. **D**–**E**, Left quadrates of specimens **D, F**, MHNM.KK377; and **E**, MSNM V6896 representing ontogenetic stage 2 and 3 (non-juvenile immature individuals) with **D**, close up on the ridged dorsal quadratojugal contact in lateral view; **E**, quadrate foramen and ventral projection of the dorsal quadratojugal contact in posterior view; and **F**, poorly delimited mandibular condyles in ventral view. **G**–**I**, Left quadrate MHNM.KK375 representing ontogenetic stage 4 (subadult) with **G**, close up on the irregular and ridged lateral surface of the dorsal quadratojugal contact in lateral view; **H**, deeply excavated ventral quadratojugal contact in lateral view; and **I**, well-delimited mandibular condyles in ventral view. **J**–**L**, Left quadrate MHNM.KK378 representing ontogenetic stage 4 (fully grown individual) with **J**, close up on the protuberant squamosal capitulum in ventral view; **K**, second dorsal quadrate ridge extending to the quadrate head in posterior view; and **L**, well-delimited entocondyle with deep intercondylar sulcus in ventral view. **Abbreviations**: **enc**, entocondyle; **ics**, intercondylar sulcus; **qr**, quadrate ridge; **ri**, ridge of the dorsal quadratojugal contact; **vpdq**, ventral projection of the dorsal quadratojugal contact. Quadrates not to scale.

At a juvenile stage represented by MHNM.KK374, the quadrate lacks several deep grooves on the lateral side of the ventral quadratojugal contact, and two grooves on the dorsal quadratojugal contact (Fig 10A–10C). This suggests a weak and loose articulation between the quadrate and quadratojugal. The ectocondyle is also poorly developed and the dorsal quadratojugal contact lacks a ventral projection.

#### State 2

Both quadrates MHNM.KK377 and MSNM V6896 show some signs of immaturity based on the fact that the mandibular condyles are not globular (Fig 10A–10C) and prominent and the squamosal capitulum is poorly delimited. The two condyles of the mandibular articulation are also weakly separated by a shallow intercondylar sulcus, indicating a loose articulation between the cranium and mandibles. Yet, the ventral projection of the dorsal quadratojugal contact is present in MSNM V6896, and was most likely lost in MHNM.KK377 due to taphonomic processes (Fig 10E). This indicates that mid-sized specimens MHNM.KK377 and MSNM V6896 belonged to immature yet not juvenile individuals and not to subadult animals, as suggested by Ibrahim et al. [22] for MSNM V6896.

#### State 3

At a slightly more advanced stage of maturity reached by the immature specimen MHNM.KK377, the mandibular condyles and intercondylar sulcus are still weakly delimited but the dorsal quadratojugal contact displays a low ridge separating two shallow grooves (Fig 10D). This indicates a stronger articulation between the quadrate and quadratojugal at that stage.

#### State 4

In a subadult, the quadratojugal contacts of MHNM.KK375 (and fully grown MHNM.KK378) are deeply excavated by ridges and fossae, evidencing a strong and immobile suture between the quadrate and quadratojugal (Fig 10G–10I). Both ento- and ectocondyles are also well-delimited and the intercondylar sulcus is deep. This indicates that the quadrate was tightly articulated with the lower jaw.

#### State 5

MHNM.KK378 is the largest quadrate and most likely belongs to a fully grown individual. The quadrate is much larger than the other ones, and the entocondyle is strongly prominent, suggesting that the intercondylar sulcus of the mandibular articulation was particularly deep (Fig 10J–10L). The squamosal capitulum is also globular and an additional quadrate ridge appears ventral to it (Fig 10K).

These ontogenetic transformations result from the fusion between the quadrate and quadratojugal, the reinforcement of the quadrate shaft, and the stabilization and tightening of the articulation of the squamosal capitulum and mandibular condyles with the squamosal and lower jaw, respectively. Given the fact that both quadratojugal contacts are deeply excavated or have an irregular surface, that the mandibular condyles are well-developed and well-delimited by an intercondylar sulcus, and that the ectocondyle is excavated by a deep depression, MHNM.KK376 (Morphotype 2) clearly belongs to a mature individual (Stage 4 to 5). Likewise, the poorly delimited mandibular condyles, associated with an irregular surface of the dorsal quadratojugal contacts, suggest that the quadrates of *Baryonyx* (and possibly *Suchomimus*) belong to an immature individual (Stage 3). Based on the deep intercondylar sulcus, the globular squamosal capitulum, the deeply excavated quadratojugal contacts and the absence of a second quadrate ridge ventral to the quadrate head, we interpret the quadrates of the *Spinosaurus aegyptiacus* neotype as belonging to a subadult individual, an opinion followed by Ibrahim et al. [22] based on histological data and the absence of coossification between vertebral centra and between the ilium and sacral vertebrae.

### Size

Spinosauridae encompasses large tetanurans and some of the largest known terrestrial predators. A complete snout from the Kem Kem beds assigned to *Spinosaurus aegyptiacus* belongs to an animal with an estimated skull length of 175 cm [21] (the skull length estimated by Ibrahim et al. [22] seems to be closer to 160 cm as it is 32% larger than the neotype subadult skull estimated as 112 cm; Ibrahim pers. comm.) and a body length estimated to reach 15 meters [22]. Based on comparison with the quadrates of *Baryonyx walkeri* and *Spinosaurus aegyptiacus* and the estimated length of their skulls, an estimation of the skull size for each quadrate can be proposed. The proportion of the quadrate relative to the skull length is significantly different in the baryonychines *Baryonyx* and *Suchomimus*, yet this difference can be explained by the fact that the isolated quadrate of *Suchomimus* likely belongs to a smaller individual than the holotype MNN GDF500 and the paratype MNN GDF501. Indeed, the premaxillae and humerus of *Baryonyx* are 17–20% smaller than those of *Suchomimus* ([25,89]; pers. obs.) whereas the best preserved quadrate is 20% larger than that of *Suchomimus* (Table 2; pers. obs.). With a quadrate height of 145 mm from entocondyle to squamosal capitulum, and an estimated skull length of 1190 mm [90], the quadrate to skull ratio is only 0.12 in *Suchomimus*, which seems to be particularly low (Table 2).

**Table 2:** Quadrate size and estimated skull length in Spinosauridae.

Taxa	Specimen	Ontogenetic stage	Quadrate subunit	Size (mm)	Width-Height Ratio	quadrate-skull percentage	Skull (mm)
*Baryonyx walkeri*	NHM R.9951 (left quadrate)	Immature	Quadrate height	181	0.55	19–20%	910–950*
			Mandibular articulation width	100		10.5–11%	
*Suchomimus tenerensis*	MNN GAD 502	Immature?	Quadrate height	145	0.48	12.1%**	1190*
			Mandibular articulation width	70		5.9%**	
*Spinosaurus aegyptiacus* (Morphotype 1)	FSAC-KK 11888	Subadult	Quadrate height	240	?	21.4%	1120*
	MHNM.KK374	Juvenile	Quadrate height	78	0.46	?	327–409*
			Mandibular articulation width	36		?	
	MHNM.KK375	Subadult	Quadrate height	145	0.53	?	677–761*
			Mandibular articulation width	77		?	
	MHNM.KK377	Immature	Quadrate height	130	0.54	?	607–682*
			Mandibular articulation width	70		?	
	MHNM.KK378	Adult	Quadrate height	220	?	?	1028–1154*
	MSNM V6896	Immature	Quadrate height	145	0.52	?	677–761*
			Mandibular articulation width	76		?	
?*Sigilmassasaurus brevicollis* (Morphotype 2)	MHNM.KK376	Mature (subadult to adult)	Quadrate height	204*	?	?	953–1070*
			Mandibular articulation width	108		?	

*Estimations.

**Considering that MNN GAD 502 (quadrate) and MNN GAD 501 (articulated premaxillae and maxillae) belong to the same individual.

Based on the reconstruction of the *Suchomimus* skull, and given the fact that *Baryonyx* cranial material is 20% smaller than that of *Suchomimus*, the skull length of *Baryonyx* can be estimated to reach around 950 mm, which is close to the value obtained by Therrien and Henderson [90] (i.e., 910 mm for the *Baryonyx* skull length). With a quadrate length of 181 mm, the quadrate-skull ratio of *Baryonyx* is 0.19 (and 0.2 based on the estimated skull of Therrien and Henderson [90]), a very close value to that calculated in the *Spinosaurus* neotype (FSAC-KK 11888) given a quadrate height of 240 mm and an estimated skull length of 1120 mm (ratio of 0.21). With a height of 220 mm, and based on the quadrate-skull ratio obtained in *Baryonyx* and *Spinosaurus* (from 0.19 to 0.21), the largest quadrate MHNM.KK378 belongs to an animal with an estimated skull length varying from 1028 to 1154 mm. This estimate is much lower than the estimated length of the skull of the largest specimen of *Spinosaurus* (i.e., 160–175 cm in MSNM V4047; [21,22]). Likewise, MHNM.KK378 is 48% and 37% shorter than the quadrates of the largest carcharodontosaurids *Giganotosaurus* (430 mm for the quadrate height; pers. obs.), and *Acrocanthosaurus* (350 mm; [91]) and *Mapusaurus* (350 mm; [92]), respectively. This can be explained by the fact that the spinosaurid skull is particularly ventrodorsally low compared to that of other basal tetanurans, and the cranium, along with the quadrate height, was subject to a ventrodorsal compression throughout the evolution of Megalosauroidea leading to Spinosauridae.

Interestingly, all spinosaurine quadrates so far collected from the Kem Kem beds and belonging to non-juvenile individuals (MHNM.KK375 to.KK378; MSNM V6896; FSAC-KK 11888; Alain Cabot private collection) are 28% smaller to 32% larger than that of *Baryonyx* and belong to animals with estimated skull lengths varying from 607 to 1154 mm, which is shorter than that of *Suchomimus* (Table 2). This either suggests that very large forms of *Spinosaurus* with skulls of more than 150 cm in length and/or fully grown individuals may have been rare in the Kem Kem assemblage, or that the quadrate height is proportionally shorter relative to the skull length in Spinosaurinae than in Baryonychinae.

### Diversity

Based on our investigation on the ontogenetic variations in the quadrates of Morphotype 1, and given the fact that Morphotype 2 and four quadrates of Morphotype 1 (MHNM.KK375, MHNM.KK378, and the two *Spinosaurus aegyptiacus* quadrates FSAC-KK 11888) belong to mature individuals, the morphological difference observed between Morphotypes 1 and 2 cannot be explained by ontogeny. Likewise, all isolated quadrates from the Kem Kem beds do not show any sign of taphonomic distortion, and it is clear that the morphological variations seen in the mandibular articulation of Morphotypes 1 and 2 do not result from postmortem deformation. There is also no evidence supporting the contention that the morphological differences observed in MHNM.KK376 are pathological and we, therefore, exclude that Morphotype 2 may belong to a pathological animal. It is finally highly unlikely that these differences are due to sexual dimorphism or interindividual variation among a single species. Indeed, the amount of difference observed in the quadrates of the two baryonychine taxa *Baryonyx walkeri* (NHM R.9951) and *Suchomimus tenerensis* (MNN GAD 502) are as large as those displayed by the two spinosaurine morphotypes. *Baryonyx* and *Suchomimus* quadrates only differ in the morphology of the ventral quadratojugal contact and the quadrate head, the degree of curvature of the medial margin of the quadrate shaft and the presence of a posterior intercondylar notch (Fig 9A–9L). Unlike *Baryonyx*, the quadrate of *Suchomimus* shows a dorsal projection of the ventral quadratojugal contact (Fig 9H), a longer ventral projection of the dorsal quadratojugal contact, and a posterior intercondylar notch (Fig 9I and 9K). The quadrate head is also subtriangular rather than subcircular and the convexity of the medial margin of the quadrate shaft is more pronounced in anterior view (Fig 9G). Contrary to the two spinosaurine morphotypes, the quadrate foramen and the dorsal quadratojugal contact of the two baryonychine taxa are almost identical in shape and outline (Fig 9C and 9I), and the anterior surface of the ectocondyle shows the same concavity. The ventral quadratojugal contact of the two Baryonychinae and the two spinosaurine morphotypes also display the same level of difference. Each morphotype can, therefore, be confidently referred to different spinosaurine taxa, evidencing the presence of two species of Spinosaurinae in the Cenomanian of North Africa.

As previously mentioned, two species of *Spinosaurus* have already been recorded from the Kem Kem beds namely, *S*. *aegyptiacus* [17–21] and *S*. *maroccanus* [2]. The latter was erected by Russell [2] in 1996 based on dentary fragments, cervical vertebrae and a dorsal neural arch uncovered in the Kem Kem beds of the Tafilalt, north of the Kem Kem region (Fig 1). An incomplete snout and additional vertebral material from the Tademaït of Algeria (Adrar Province, central Algeria; Albian?) as well as an isolated vertebra from the ‘Grès rouges infracénomanien[s]’ of Morocco were later ascribed to this species by Taquet and Russell [93] and D’Anastasio and Capasso [94], respectively (n.b., the isolated vertebra described by D’Anastasio and Capasso [94] likely comes from the Kem Kem beds of Cenomanian age at Taouz, and not from the Albian of ‘Taout’ *sensu* D’Anastasio and Capasso [94]; this vertebra was latter assigned to *Sigilmassasaurus* by McFeeters et al. [8]). The validity of *S*. *maroccanus* was, however, questioned by Sereno et al. [25], Buffetaut and Ouaja [95], Rauhut [86], Dal Sasso et al. [21], Carrano et al. [71] and Ibrahim et al. [22] who regard this species as a *nomen dubium* and/or a junior synonym of *S*. *aegyptiacus*. Russell [2] distinguished *S*. *maroccanus* from *S*. *aegyptiacus* by the proportion of the mid-cervical vertebrae based on one isolated cervical vertebra. According to Russell [2], the “ratio between length of centrum (excluding anterior articular condyle) and height of posterior articular facet of centrum [is] approximately 1.5 in mid-cervical vertebrae” ([2], p. 356) versus 1.1 in the Egyptian species [93]. Rauhut [96] interpreted this difference to a more posterior position of the cervical vertebra, noticing that “the posterior cervicals are relatively shorter than the mid-cervicals” in theropods, therefore “the difference in ratio is thus insufficient to diagnose a separate species” ([96], p. 100). Similarly, Buffetaut and Ouaja [95] doubt of the exact position of the isolated vertebra described by Russell [2], arguing that Stromer’s original material of *Spinosaurus aegyptiacus* is no longer available for direct comparison and that the material illustrated by Stromer was damaged. In addition, Mortimer [97] notes that ratios in *Baryonyx walkeri* cervical series range from 1.25 to 1.81, which is the same amount of variation supposedly separating the *Spinosaurus* species. Recently, Ibrahim et al. [22] regarded the difference in proportion “as an artifact of differing ways to measure opisthocoelous vertebrae” ([22]: supplemental information p.10).

**Fig 11:**
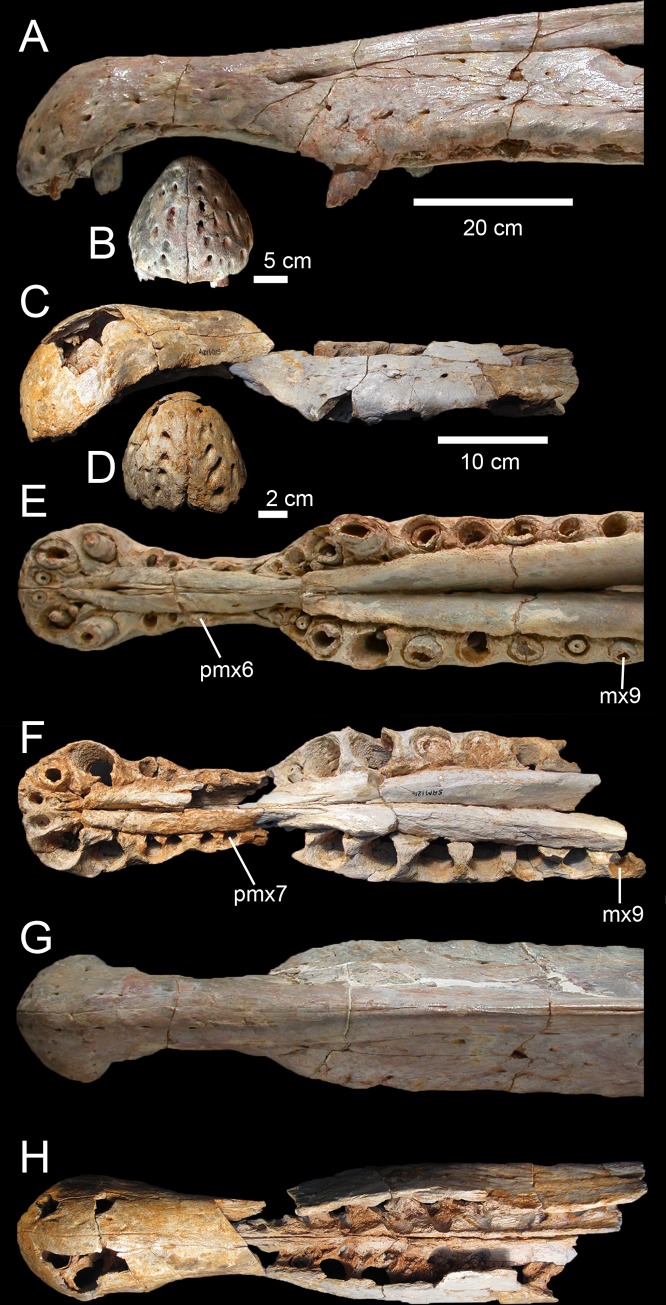
Comparison of the snout of two specimens of *Spinosaurus* from the ‘Continental intercalaire’ of Northwestern Africa. **A**–**H**, Fused maxillae and premaxillae of **A**–**B, E, G**, MSNM V4047 referred to *Spinosaurus aegyptiacus* by Dal Sasso et al. [21] (courtesy of Simone Maganuco); and **C**–**D, F, H**, MNHM SAM 124 referred to *Spinosaurus maroccanus* (*nomen dubium*) by Taquet and Russell [93] in **A, C**, lateral; **B, D**, anterior; **E, F**, ventral; and **G, H**, dorsal views. Abbreviation: **mx9**, ninth maxillary alveolus; **pmx6**, sixth premaxillary alveolus, **pmx7**, seventh premaxillary alveolus. Scale = 20 cm (A, E, G), 10 cm (C, F, H), 5 cm (B), 2 cm (D).

As to the cranial material assigned to the Moroccan species of *Spinosaurus*, Russell [2] mentioned the fact that the dentary fragments referred to the holotype of *S*. *maroccanus* are “essentially indistinguishable” from the type of *S*. *aegyptiacus* (Russell [2], p. 356). A similar observation can be made for the premaxillae, maxillae and dentary later ascribed to *S*. *maroccanus* by Taquet and Russell [93]. Indeed, the differential diagnosis proposed by these authors for this species corresponds exactly to the description of the material identified as belonging to *S*. *aegyptiacus* by Milner [20] and Dal Sasso et al. [21]. The morphology of the fused premaxillae and maxillae referred to *S*. *maroccanus* are extremely similar to those ascribed to *S*. *aegyptiacus*, and the main difference lies in the premaxillary tooth count (Fig 11). In *S*. *maroccanus*, each premaxilla bears seven alveoli whereas the premaxilla of the specimen MSNM V4047 referred to *S*. *aegyptiacus*, only has 6 teeth. This difference is, however, negligible given the fact that tooth count can vary during ontogeny in some basal tetanurans (e.g., [98,99]), between individuals of the same species (e.g., [100–103]), and even between left and right premaxillae of a same specimen (e.g., [89,104]). In addition, a second specimen with fused premaxillae referred to *Spinosaurus* cf. *aegyptiacus* (NHM R.16420; [20]) also possesses seven premaxillary alveoli. Consequently, such a difference in premaxillary tooth count between the two species of *Spinosaurus* is here considered as ontogenetic or intraspecific, and not taxonomically significant, and cranial material ascribed to *Spinosaurus maroccanus* are here tentatively considered to belong to *Spinosaurus aegyptiacus*, pending a comprehensive description of this material.

More recently, Richter et al. [9] reported three types of crown ornamentation and enamel texture in isolated teeth assigned to *Spinosaurus*. In a first morphotype, flutes are distinct, numerous, and strongly developed on the lingual surface and only weakly developed on the labial surface. A second morphotype is defined by well-developed flutes on both lingual and labial sides, yet this fluting is more distinct, numerous and narrower lingually. Finally, the absence of flutes and a smooth enamel surface texture on the crown characterize a third morphotype of *Spinosaurus* teeth. According to Richter et al. [9], there is no gradational transition between each crown ornamentation which may suggest that more than one species of *Spinosaurus* were present in the Cenomanian of Morocco. However, Richter et al. [9] suggested that different ornamentations may also be related to a strong variation in the dentition of *Spinosaurus*, although they noted that such heterodonty has never been observed in any articulated specimen of *Spinosaurus*. Nonetheless, no *Spinosaurus* tooth-bearing bone reported in the literature so far preserves complete in-situ teeth, and the morphological variation of *Spinosaurus* teeth along the dentition remains unknown (pers. obs.). Furthermore, due to the straight and centrally positioned carinae as well as the absence of lingual and/or labial depressions mediobasally situated on the crown, the labial and lingual sides are excessively difficult to distinguish in isolated teeth of Spinosauridae (pers. obs.). In addition, variations in crown ornamentation may be due to ontogenetic factors, as in the case with denticle size and density, and crown thickness in the dentition of basal tetanurans (e.g., [99,105]). Finally, teeth of *Baryonyx* and *Suchomimus* display a similar variation in the number and development of flutes (pers. obs.). In *Baryonyx*, in which all isolated teeth with the specimen number NHM R.9951 belong to same individual [89], some crowns do not possess any flutes whereas others show more than eight distinctly developed flutes on the lingual side (pers. obs.). Variation in the development of the veined enamel texture of the crowns have also been noted. It is, therefore, likely that variation in crown ornamentation in Spinosauridae is positional and possibly ontogenetic rather than taxonomic, and the hypothesis that more than one species of Spinosauridae were present in the Kem Kem beds based on different tooth morphotypes is, therefore, poorly supported.

As recently formulated by Ibrahim and Sereno [106], we agreed that there was “no basis to distinguish spinosaurid remains at generic or specific levels from eastern and western localities in coeval Cenomanian-age rocks on Africa” ([106], p. 130) prior to 2015. Following the opinion of Sereno et al. [25], Buffetaut and Ouaja [95], Rauhut [86], Dal Sasso et al. [21], Carrano et al. [71] and Ibrahim et al. [22], we initially considered that the material hitherto reported in the literature did not convincingly support the existence of a spinosaurid taxon distinct from *S*. *aegyptiacus* in the Kem Kem beds. The situation has, however, changed with the recent description of additional *Sigilmassasaurus* remains from the Kem Kem beds by Evers et al. [27], whose publication came out at the final stage of the correction of this manuscript. Evers et al. [27] brought compelling evidence supporting the fact that *Sigilmassasaurus brevicollis* and *Spinosaurus maroccanus* are conspecific, and that *Sigilmassasaurus brevicollis* and *Spinosaurus aegyptiacus* are distinct spinosaurid taxa. They additionally made a strong case regarding the presence of more than one spinosaurid taxon in the Kem Kem assemblage compounds [27]. The occurrence of two morphotypes of spinosaurid quadrates in the Kem Kem beds, therefore, corroborates Evers et al.’s [27] hypothesis on the presence of a second spinosaurid taxon, here represented by the isolated quadrate MHNM.KK376, in the Early Late Cretaceous of what is now Morocco, increasing the already high diversity of predatory dinosaurs in the Kem Kem beds. Due to the paucity of material known for this second spinosaurid species (i.e., a single incomplete bone) and given the absence of precise geographic and stratigraphic information for the type specimen, we initially refrained from erecting a new taxon of Spinosauridae (despite the fact that MHNM.KK376 possesses two unambiguous autapomorphies and a unique combination of characters, see S1 File). Yet, with the presence of the *Sigilmassasaurus* and a second spinosaurid taxon in the Kem Kem assemblage compound, and given the fact that the quadrates belonging to Morphotype 1 are confidently ascribed to the neotype of *Spinosaurus aegyptiacus* [27], we tentatively refer MHNM.KK376 to *Sigilmassasaurus brevicollis*, pending the discovery of cranial material for this taxon. Evers et al. ([27], p.62) wrote that “it is unclear if the second spinosaurid from the ‘Kem Kem beds’ can be referred to *Spinosaurus aegyptiacus*, or another, currently unrecognized taxon”. Because we consider the neotype of *Spinosaurus aegyptiacus* FASC-KK 11888 as valid, and given the similarities between the holotype material of *Spinosaurus aegyptiacus*, FASC-KK 11888, and the material assigned to a second spinosaurid taxon from the Kem Kem beds by Evers et al., [27] (i.e., NHM PV R 16429, BSPG 2006 I 57, BSPG 2013 I 97 and NHM PV R 36637; see Evers et al., [27] for more information), *Spinosaurus aegyptiacus* is here considered as the second spinosaurid taxon from the Kem Kem assemblage compound. Evers et al. [27] also note that it is speculative to comment on the phylogenetic position of *Sigilmassasaurus* among Spinosauridae based on the material currently known for this taxon. Nevertheless, based on the morphology of the quadrate ascribed to this taxon, and given the total absence of serrated spinosaurid teeth in the Kem Kem beds, *Sigilmassasaurus brevicollis* is here considered as a Spinosaurinae.

The presence of two quadrate morphotypes (MHNM.KK378 of Morphotype 1 and MHNM.KK376 of Morphotype 2) from the Kem Kem beds, and probably from the Ifezouane Formation, also suggests that *Spinosaurus aegyptiacus* and the second spinosaurine taxon tentatively referred to *Sigilmassasaurus brevicollis* may have been coeval species. Because the large majority of theropod material are from the upper part of the Ifezouane Formation, and that the assemblage of the latter is qualitatively relatively homogeneous [7], it is indeed likely that all theropod taxa recovered from the Kem Kem beds were in fact coeval. Caution should, therefore, be exercised when referring spinosaurid material from the Kem Kem beds to the single species *Spinosaurus aegyptiacus* based on paleogeographical and stratigraphical data [27]. Ibrahim et al.’s [22] recent reconstruction of *Spinosaurus aegyptiacus*, which is based on the association of cranial and postcranial elements belonging to different individuals possibly separated by a considerable time span, should, therefore, be regarded as tentative. It is, indeed, more than likely that the reconstructed morphology illustrating a quadrupedal *Spinosaurus*, the only quadrupedal theropod known so far, is based on artificially associated bones from two different species of Spinosaurinae [27]. Likewise, Evers et al. [27] note that the material defining the neotype of *Spinosaurus aegyptiacus* may not represent the same individual, so that the proportion of the pelvis and hind limb remains with the rest of the skeleton in Ibrahim et al.’s [22] model should also be considered as hypothetical.

Several scenarios have been proposed to explain the high diversity of theropod dinosaurs in the Kem Kem beds, and the unbalanced ratio between predatory and herbivorous dinosaurs. Up to seven theropod clades have been recorded in the Kem Kem beds (i.e., Basal Ceratosauria, Noasauridae, Abelisauridae, Spinosauridae, Carcharodontosauridae, Sigilmassasauridae, and Dromaeosauridae). The overabundance of theropod material, explained by collecting bias [40] and a ‘time-averaging’ effect [41], but supported by field data [7], strongly suggests some niche partitioning in a very widespread heterogeneous deltaic paleoenvironment [7,107]. This taxonomic abundance of theropods might, however, be due to an overestimation of the number of clades in the Kem Kem compound assemblage. A recently reported femur of a juvenile noasaurid may belong to the basal ceratosaur *Deltadromeus*, classified as a Noasauridae by Sereno et al. [10] and Tortosa et al. [15], and isolated teeth referred to dromaeosaurids may in fact belong to a noasaurid [1]. Given the important morphological similarities noted by Fanti and Therrien [108], Hendrickx and Mateus [109], and Evans et al. [1] between the dentition of Noasauridae and Dromaeosauridae, it is indeed likely that the dromaeosaurid teeth reported by Amiot et al. [28] and Richter et al. [9] from the Kem Kem beds belong to *Deltadromeus*. Consequently, only four non-avian theropod clades (i.e., ‘Elaphrosaurids’/Noasauridae, Abelisauridae, Spinosauridae, and Carcharodontosauridae) and six theropod taxa (*Deltadromeus*, an indeterminate abelisaurid, *Spinosaurus*, *Sigilmassasaurus*, *Carcharodontosaurus*, and *Sauroniops*) may have been present in the Cenomanian of Northern Africa, a diversity equal to that of the Kimmeridgian/Tithonian Lourinhã Formation of Portugal, which yielded remains of at least five definitive non-avian theropod clades and possibly nine taxa (i.e., the ceratosaurid *Ceratosaurus*, an indeterminate abelisaurid, the megalosaurid *Torvosaurus*, the allosaurid *Allosaurus*, the tyrannosauroid *Aviatyrannis*, the basal coelurosaur *Lourinhanosaurus*, the compsognathid cf. *Compsognathus*, and the paravians *Richardoestesia* and cf. *Paronychodon*; see [109] and references therein). A main difference with the Lourinhã Formation is, however, the low diversity of herbivorous dinosaurs and the scarcity of their remains in the Kem Kem beds [32], which seems to support an unbalanced food chain given such a diversity of carnivorous dinosaurs. Yet, with two sauropod taxa, at least one ornithopod taxon, and a large taxonomic diversity of fish, amphibians, crocodiles, turtles, and pterosaurs (see [5,32,37,39,110–116] and references therein), prey was not rare in the Kem Kem ecosystems and seems to have been in a sufficient quantity to feed six predatory dinosaurs of various size. As for Spinosauridae, the presence of two taxa is not surprising given the opportunistic nature of their feeding strategies ([89,117]; see below). The abundance of spinosaurid remains in the Ifezouane Formation [7] and ‘Grès rouges’ Formation of the Guir basin [62] (which is contemporaneous with the Kem Kem beds; [62]), in Western Algeria, may also be explained by the presence of two species of Spinosaurinae in the fluvial system of the Continental intercalaire of North Africa, as well as the particularly high replacement and low formation rates of teeth in this clade [118].

### Morphofunctional analysis

Spinosauridae form a highly specialized clade of tetanurans characterized by an elongated and narrow snout with spatulate jaws (or ‘terminal rosette’ *sensu* Charig and Milner [89]), sigmoid alveolar margin of the rostrum, posteriorly retracted external nares, a secondary bony palate, and subconical fluted teeth bearing minute or no denticles [21,22,25,87,89,119,120]. Such combination of cranial features, associated with the development of robust anterior limbs bearing a huge claw in digit I, was interpreted as indicating piscivorous [18,20–22,25,42,47,87,89,121,122] or scavenging lifestyles [89,123]. Postcranial bones of a juvenile *Iguanodon* and *Lepidotes* scales found in the ribcage of the holotype of *Baryonyx* [89], as well as a tooth of a spinosaurid embedded within a pterosaur cervical vertebra [117] and the association of *Baryonyx* material with isolated *Iguanodon* teeth from Portugal (O.M. pers. obs.), support the fact that these derived tetanurans were opportunistic animals feeding on fish, ornithopods, and pterosaurs. Likewise, on the basis on the isotopic ratios of oxygen in their remains, spinosaurids have been interpreted as semi-aquatic [124], a conclusion subsequently supported by the peculiar morphology of their hind limbs and feet, and the high density of their bones in *Spinosaurus* [22].

**Fig 12:**
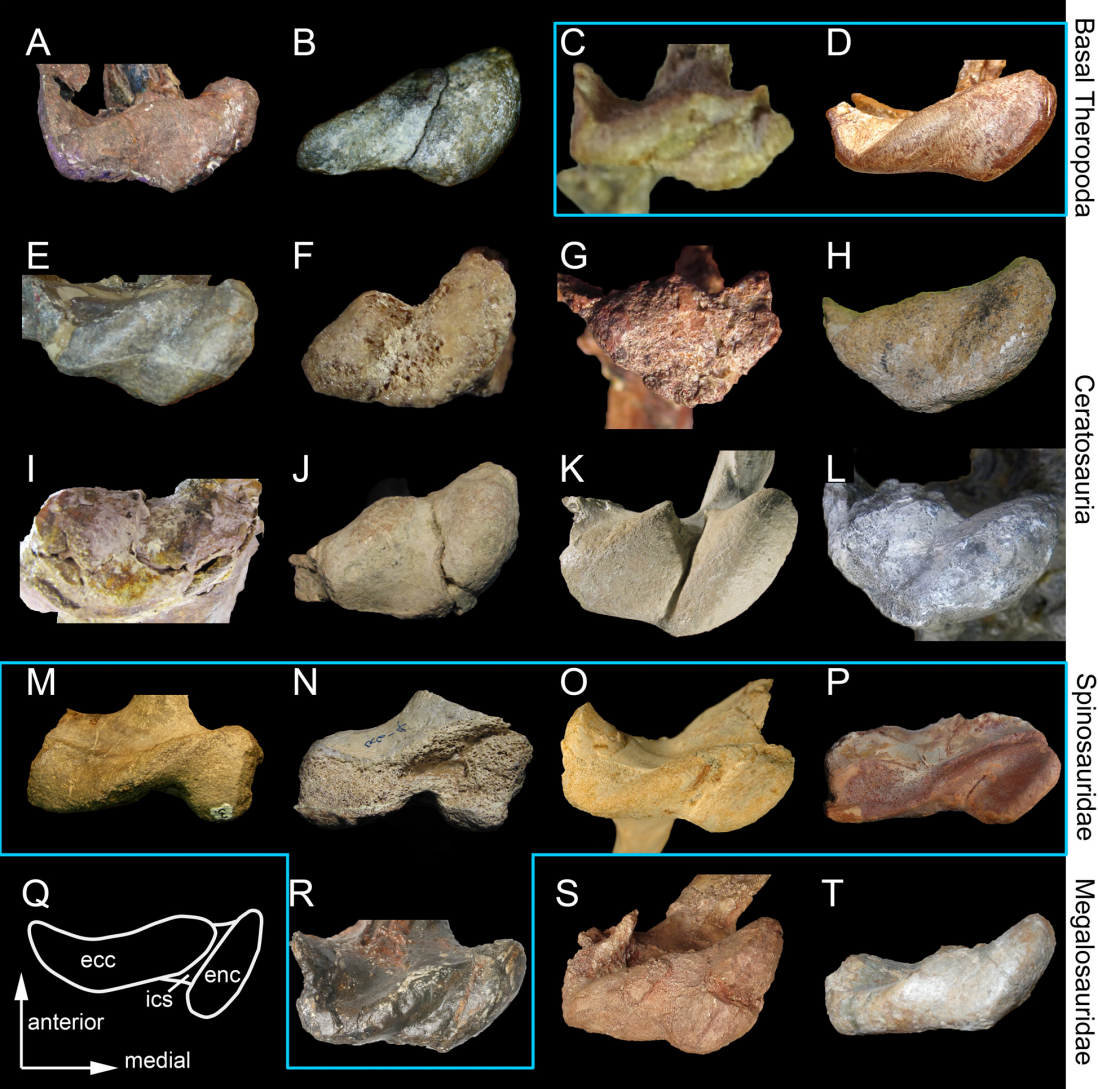
Morphological diversity of the mandibular articulation in non-avetheropod theropods. **A–P**, **R–T**, right quadrate (unless indicated) in ventral view in; **A**, *Herrerasaurus ischigualastensis* (formerly ‘*Frenguellisaurus*’ *ischigualastensis*; PVSJ 053; left reversed); **B**, *Eodromaeus murphi* (PVSJ 562); **C**, *Tawa hallae* (GR 241; courtesy of Sterling Nesbitt); **D**, *Dilophosaurus wetherilli* (UCMP 37302; left reversed; courtesy of Juan Canale); **E**, *Ceratosaurus nasicornis* (MWC 1; left reversed); **F**, *Masiakasaurus knopfleri* (FMNH PR 2496); **G**, *Noasaurus leali* (PVL 4061); **H**, *Ilokelesia aguadagrandensis* (MCF-PVPH 35); **I**, *Abelisaurus comahuensis* (MPCA 11098; left reversed; in posteroventral view); **J**, *Aucasaurus garridoi* (MCF-PVPH 236); **K**, *Majungasaurus crenatissimus* (FMNH PR 2100; left reversed); **L**, *Carnotaurus sastrei* (MACN-CH 894; left reversed; courtesy of Pablo Asaroff); **M**, *Baryonyx walkeri* (NHM R.9951; left reversed); **N**, *Suchomimus tenerensis* (MNN GAD502; left reversed); **O**, *Spinosaurus aegyptiacus* (MHNM.KK375; left reversed); **P**,? *Sigilmassasaurus brevicollis* (Morphotype 2; MHNM.KK376; left reversed); **Q**, anatomy and orientation of an idealized right quadrate in ventral view; **R**, *Eustreptospondylus oxoniensis* (OUMNH J.13558; courtesy of Paul Barrett); **S**, *Afrovenator abakensis* (MNN UBA1; left reversed; courtesy of Roger Benson); **T**, *Torvosaurus tanneri* (BYU-VP 5110). **Abbreviations**: **ecc**, ectocondyle; **enc**, entocondyle; **ics**, intercondylar sulcus. Taxa framed in blue are those belonging to morphoclade II retrieved in the phylogenetic morphometric analysis (Fig 8A). Quadrates not to scale.

The peculiar morphology of the mandibular articulation of *Baryonyx* and Morphotypes 1 and 2 of Spinosaurinae provides additional information on the jaw mechanics of Spinosauridae. In mature spinosaurid individuals, the shape of the articulation significantly differs from that of other theropods (Fig 12P). Whereas the ectocondyle typically forms a broad subcircular, elliptical or parabolic protuberance in many theropods, the ectocondyle of Spinosauridae is particularly elongated, much longer than the entocondyle, and corresponds to a narrow and sigmoid ridge that extends behind the entocondyle anteromedially. Although the ectocondyle is anteroposteriorly large in its lateral part, a concavity is present on the anterolateral surface of the condyle so that the apical ridge of the ectocondyle is posteriorly displaced in its lateral part in Spinosauridae. In addition, the intercondylar sulcus is narrow and strongly diagonally oriented so that its posterior orientation follows the posterior surface of the ectocondyle, which becomes entirely lateromedially oriented along its lateral part. This condition is exacerbated in MHNM.KK376 in which the ectocondyle forms a particularly narrow and sigmoid ridge extending well behind the entocondyle. The ectocondyle is, therefore, much longer than the entocondyle, which is oblong in outline and much more protuberant than the ectocondyle. In this second spinosaurine morphotype, the anterior surface of the ectocondyle is deeply excavated by a large depression whereas the diagonal intercondylar sulcus is narrow and very well-defined.

**Fig 13:**
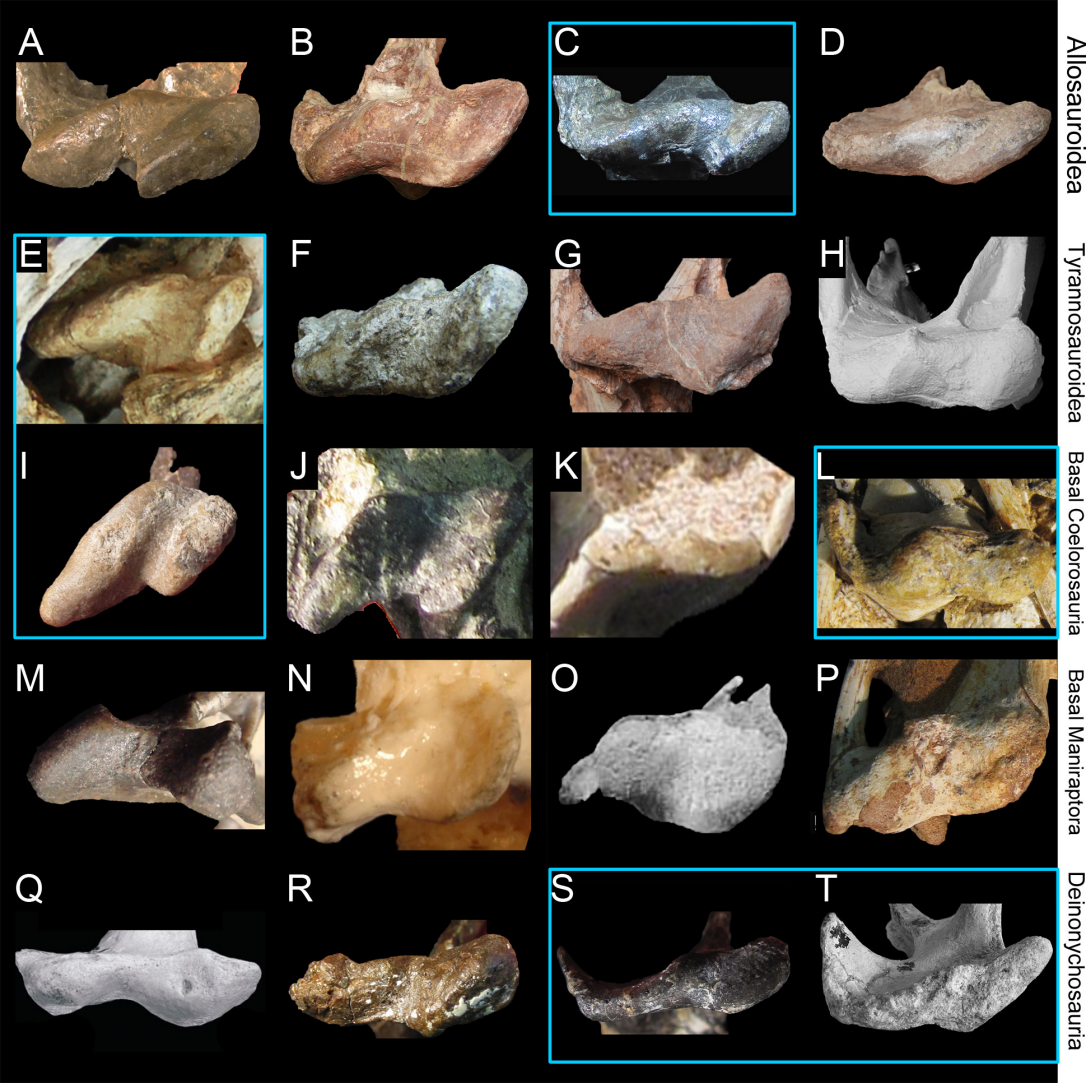
Morphological diversity of the mandibular articulation in non-avian Avetheropoda. **A–T**, Right quadrate (unless indicated) in ventral view in; **A**, *Allosaurus* ‘*jimmadseni*’ (SMA 05/002); **B**, *Aerosteon riocoloradensis* (MCNA-PV-3137; left reversed; courtesy of Martin Ezcurra); **C**, *Acrocanthosaurus atokensis* (NCSM 14345); **D**, *Giganotosaurus carolinii* (MUCPv-CH-1; left reversed); **E**, *Guanlong wucaii* (IVPP V14531; left reversed; courtesy of Oliver Rauhut); **F**, *Eotyrannus lengi* (MIWG 1997.550); **G**, *Qianzhousaurus sinensis* (GM F10004-1; left reversed; courtesy of Stephen Brusatte); **H**, *Tyrannosaurus rex* (BHI l013; modified from Larson [133]); **I**, *Bicentenaria argentina* (MPCA 865; left reversed); **J**, *Ornitholestes hermanni* (AMNH FARB 619); **K**, *Shuvuuia deserti* (IGM 100–1001; left reversed); **L**, *Gallimimus bullatus* (IGM 100–1133; left reversed); **M**, *Falcarius utahensis* (UMNH VP 14559; left reversed; courtesy of Lindsay Zanno); **N**, *Avimimus portentosus* (cast of PIN 3907–3; left reversed; courtesy of Lawrence Witmer); **O**, Indeterminate Oviraptoridae (?*Ajancingenia yanshini* or? *Conchoraptor gracilis*; IGM A; left reversed; [85]); **P**, *Citipati osmolskae* (IGM 100–978); **Q**, Indeterminate Oviraptoridae (?*Saurornithoides mongoliensis*; IGM 100–1083; [134], modified); **R**, *Dromaeosaurus albertensis* (AMNH FARB 5356); **S**, *Bambiraptor feinbergi* (AMNH FARB 30556; left reversed); **T**, *Tsaagan mangas* (IGM 100–1015; [135]; courtesy of Mick Ellison). Taxa framed in blue are those belonging to morphoclade II retrieved in the phylogenetic morphometric analysis (Fig 8A). Quadrates not to scale.

The morphology of the mandibular articulation strongly differs from that of the first and third morphoclades obtained in the phylogenetic morphometric analysis (Fig 8A). Both morphotypes of the mandibular articulation are characterized by a weakly lateromedially elongated mandibular articulation showing a wide and poorly lateromedially oriented mandibular sulcus and an ectocondyle subequal to or smaller than the entocondyle. Theropods recovered in these two morphoclades encompass ceratosaurs, allosaurids, non-proceratosaurid tyrannosauroids, therizinosaurs, alvarezsauroids, oviraptorosaurs, and troodontids (Figs 12 and 13). As already suggested by Hendrickx et al. [61], these clades encompass two types of theropods: the large predators with relatively short and broad skulls resisting torsional bending such as ceratosaurs, some megalosaurids and allosauroids, and tyrannosaurids, and the herbivorous theropods with beaks, edentulous jaws or leaf-shaped crowns such as alvarezsauroids, therizinosauroids, oviraptorosaurs, and troodontids. In these two types of theropods, a broad and/or latero-medially short articulation of the quadrate was advantageous for either feeding on large prey or on hard plants thanks to a powerful and highly efficient bite that could resist high degrees of stresses and torsional bending [46,125,126].

On the other hand, spinosaurid taxa were recovered among the second morphoclade obtained in the phylogenetic morphometric analysis (Fig 6A). This morphotype of the mandibular articulation is characterized by a diagonally oriented intercondylar sulcus combined with an elongated and lateromedially oriented ectocondyle much longer than the entocondyle. Such a morphology of the mandibular articulation is also present in the primitive theropod *Tawa*, the dilophosaurid *Dilophosaurus*, the megalosaurid *Eustreptospondylus*, the basal carcharodontosaurids *Acrocanthosaurus* and *Shaochilong*, the proceratosaurid *Guanlong*, and the dromaeosaurids *Bambiraptor* and *Tsaagan* (Figs 12 and 13; blue frames). These taxa are roughly distributed in the same morphospace in the geometric morphometric analysis (Fig 7). They have also been recovered in a same morphoclade in the phylogenetic morphometric analysis performed by Hendrickx et al. [61]. Based on similar results, Hendrickx et al. [61] have suggested that these distantly related theropods shared the same jaw mechanics where the two mandibular rami were laterally displaced when the mandible was depressed. Two types of theropods show this morphology of the mandibular articulation, namely the weakly and fast biting carnivores with an elongated skull, and sometimes a sigmoid alveolar margin of the upper jaw (i.e., *Tawa*, *Dilophosaurus*, *Eustreptospondylus*, *Guanlong*, *Tsaagan*, *Bambiraptor*), and the massive predators with powerful and robust skulls that were able to swallow large chunks of meat (*Acrocanthosaurus*, *Shaochilong*). Spinosauridae were recovered in the first type of theropods, yet they differ from dilophosaurids, *Eustreptospondylus*, proceratosaurids and dromaeosaurids by having a much larger body size and a strongly elongated crocodile-like skull. Such a transformation of the skull also affected the mandibular articulation which shows a derived morphology among theropods.

**Fig 14:**
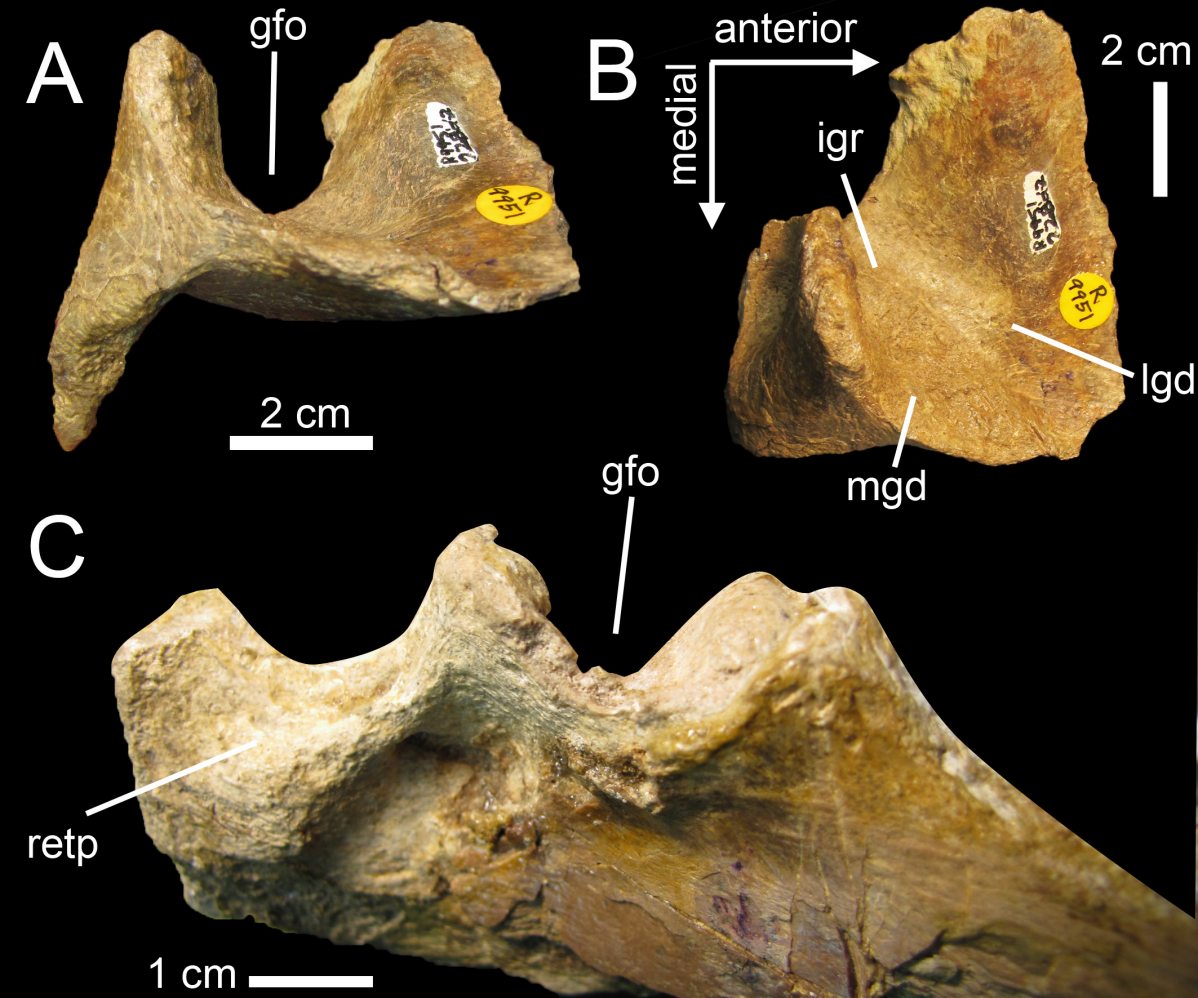
Morphology of the left articular in Spinosauridae. **A–B**, *Baryonyx walkeri* (NHM R.9951); and **C**, *Irritator challengeri* (SMNS 58022) in **A, C**, medial; and **B**, dorsal views. **Abbreviations**: **igr**, interglenoid ridge; **gfo**, glenoid fossa; **lgd**, lateral glenoid depression; **mgd**, medial glenoid depression; **retp**, retroarticular process.

**Fig 15:**
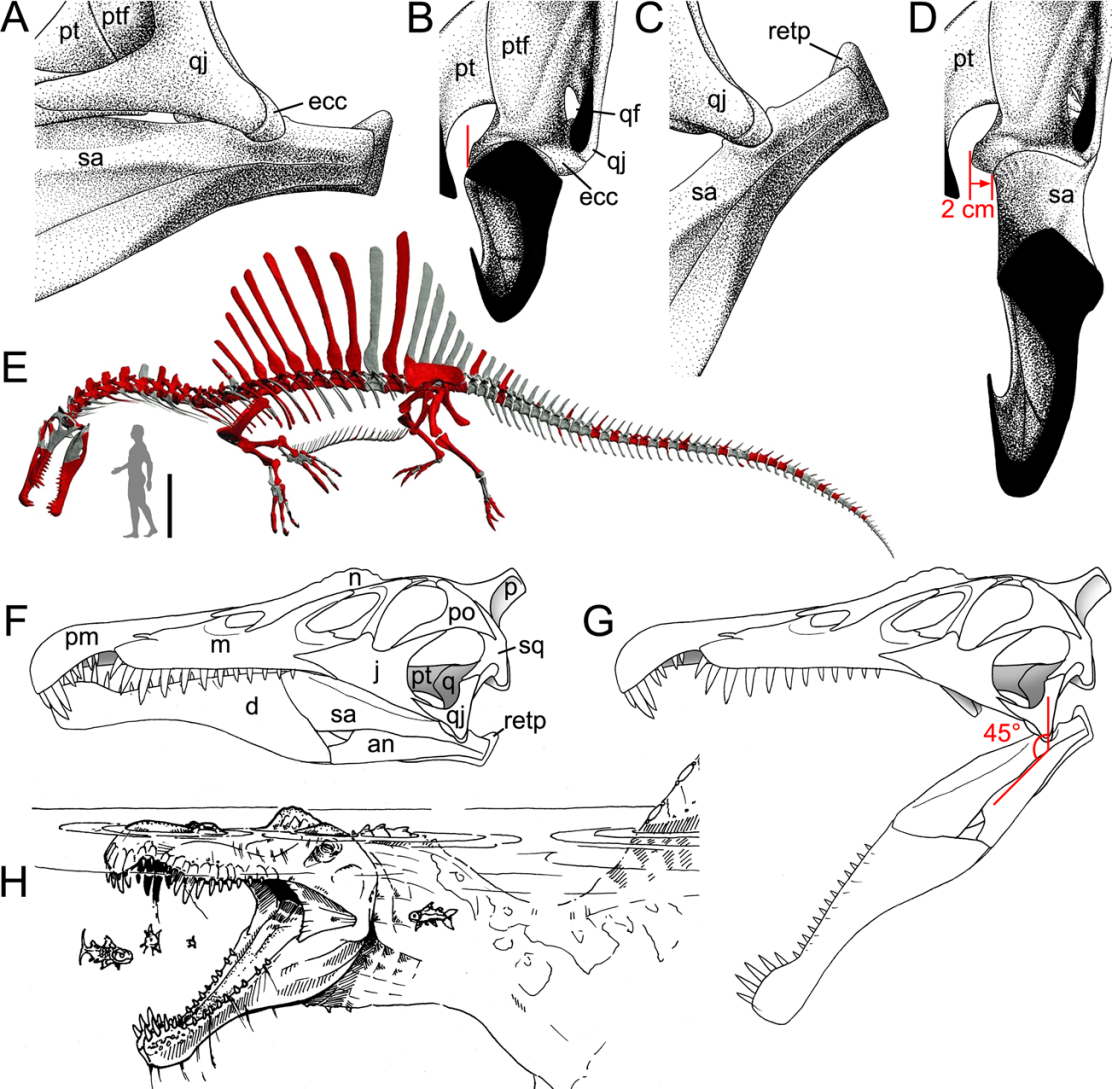
Jaw mechanics in the spinosaurid *Spinosaurus*. **A–D**, Mandibular articulation; and **F, G**, skull in **A, C, F–G**, lateral; and **B, D**, anterior views; when **A–B, F**, the mouth is closed; and **C–D, G**, fully open, illustrating the lateral movement (in red) of the mandibular ramus for a 45° rotation of the lower jaw (courtesy of Jaime A. Headden); **E**, skeletal reconstruction of *Spinosaurus aegyptiacus* by Ibrahim et al. [22]) in swimming position in lateral view with a human (1.8 m) as a scale (modified from Ibrahim et al. [22]). This model is based on spinosaurid cranial and postcranial remains (colored in red) from the Albian-Cenomanian of Northern Africa which possibly belong to two spinosaurine taxa (see also Evers et al. [27]); **H**, reconstruction of a semi-aquatic *Spinosaurus* in fishing position (i.e., jaws wide open) in anterolateral view (courtesy of Jason Poole). **Abbreviations: an**, angular; **ar**, articular; **d**, dentary; **ecc**, ectocondyle; **enc**, entocondyle; **j**, jugal; **m**, maxilla; **n**, nasal; **p**, parietal; **pm**, premaxilla; **po**, postorbital; **pt**, pterygoid; **ptf**, pterygoid flange; **q**, quadrate; **qf**, quadrate foramen; **qj**, quadratojugal; **retp**, retroarticular process of the articular; **sa**, surangular; **sq**, squamosal.

The presence of a narrow and posteriorly displaced ectocondyle displaying a large concave surface on the anterior face first implies a very strong and particularly stable articulation between the mandibular condyles of the quadrate and the glenoid fossa of the articular. In *Baryonyx* and *Irritator*, the dorsal margin of the articular bone shows a deep glenoid fossa formed by two depressions separated by a faint interglenoid ridge (Fig 14B; Sues et al. [87]; n.b., the left articular of *Baryonyx* was identified as the right atlantal neural arch by Charig and Milner [89] and the central body of the left pterygoid by Sereno et al. [25], whereas the right articular was interpreted as the left postorbital by Charig and Milner [89] and the posterior portion of the right surangular by Sereno et al. [25]; Matt Carrano pers. comm.). A similar morphology most likely existed in *Spinosaurus*, and Morphotype 2 probably had one of the most stable mandibular articulations among all theropods. Indeed, based on the morphology of the mandibular articulation, the articular of MHNM.KK376 was deeply excavated by a narrow glenoid fossa. It also had two well-defined lateral and medial glenoid depressions divided by an acute interglenoid ridge. The articulation must also have been stabilized by a smooth and prominent convexity delimiting the anterior surface of the lateral glenoid depression. Evolution towards a particularly stable mandibular articulation in Spinosauridae was probably the result of two independent factors: an important lateral displacement of the two rami of the mandible, and the swift movement of the jaw opening. As soon as the mandible was depressed, the interglenoid ridge of the articular slid along the lateromedially oriented intercondylar sulcus of the quadrate, forcing the articular, and consequently the two mandibular rami, to be displaced laterally (Fig 15A–15D, 15F and 15G). This lateral displacement was increased by the fact that the interglenoid fossa could carry on its movement along the ventrodorsally high posterior articular surface of the ectocondyle, especially in Morphotype 2. A strong lateral displacement of the two rami allowed the pharynx of Spinosauridae to be significantly enlarged, therefore favoring the deglutition of whole prey or large chunks of food.

A similar lateral displacement of the lower jaw was also observed in pterosaurs and living pelecanids, which share an elongated and narrow skull and a piscivorous diet with spinosaurids [127–130]. Eaton [127] was one of the first to describe a spiral groove in the quadrate of *Pteranodon*. This obliquely oriented intercondylar sulcus was interpreted as forming “an effective screw that thrust apart the mandibular rami when the mouth is opened” and as being “directly concerned in the widening of the mouth” (Eaton [127], p. 5). Eaton [127] and Wellnhofer [129] compared this peculiar jaw mechanism with that of the pelican. The lateral displacement of the rami when the jaw was depressed was illustrated and exhaustively described by Wellnhofer [129] in *Ornithocheirus* and *Pteranodon*. Wellnhofer [129] and Bennett [128] measured a lateral displacement of one centimeter for a 90° depression of the mandible in the pelican and *Pteranodon*, respectively. According to Wellnhofer [122], this corresponds to a widening of 25% of the mouth in *Pelecanus*. By using a cast in clay, we could measure, in MHNM.KK376, a displacement of two cm of the rami for a 45° depression (Fig 15D and 15G). This corresponds to 18% of the lateromedial width of the mandibular articulation of this specimen. As observed by Bennett [128] for *Pteranodon*, the depression of the mandible only spreads the rami slightly, but may have helped in swallowing large items.

Wellnhofer [129] noted that the lateral displacement of the ramus prevents the retroarticular process of the articular from contacting the quadrate, allowing extreme depression of the mandible (Wellnhofer [129]: Fig 7). Spinosaurids did not have such extreme movement of the lower jaw as the glenoid fossa is deep, and the retroarticular process posterior to this fossa and visible in the *Baryonyx* and *Irritator* articular (Fig 14), was most likely abutting against the quadrate body before the mandible was depressed at an angle of 90°. Yet, based on a reconstruction of the *Irritator* skull by Sues et al. [87], in which the long axis of the mandibular articulation of the quadrate is ventrodorsally inclined, a 70° rotation of the mandible from its horizontal position may have been possible. Similar to what has been noted for *Pteranodon*, the helical joint of spinosaurids also provided better resistance to medial displacement of the mandibular rami, and was important in maintaining accurate alignment between the jaws [128].

**Fig 16:**
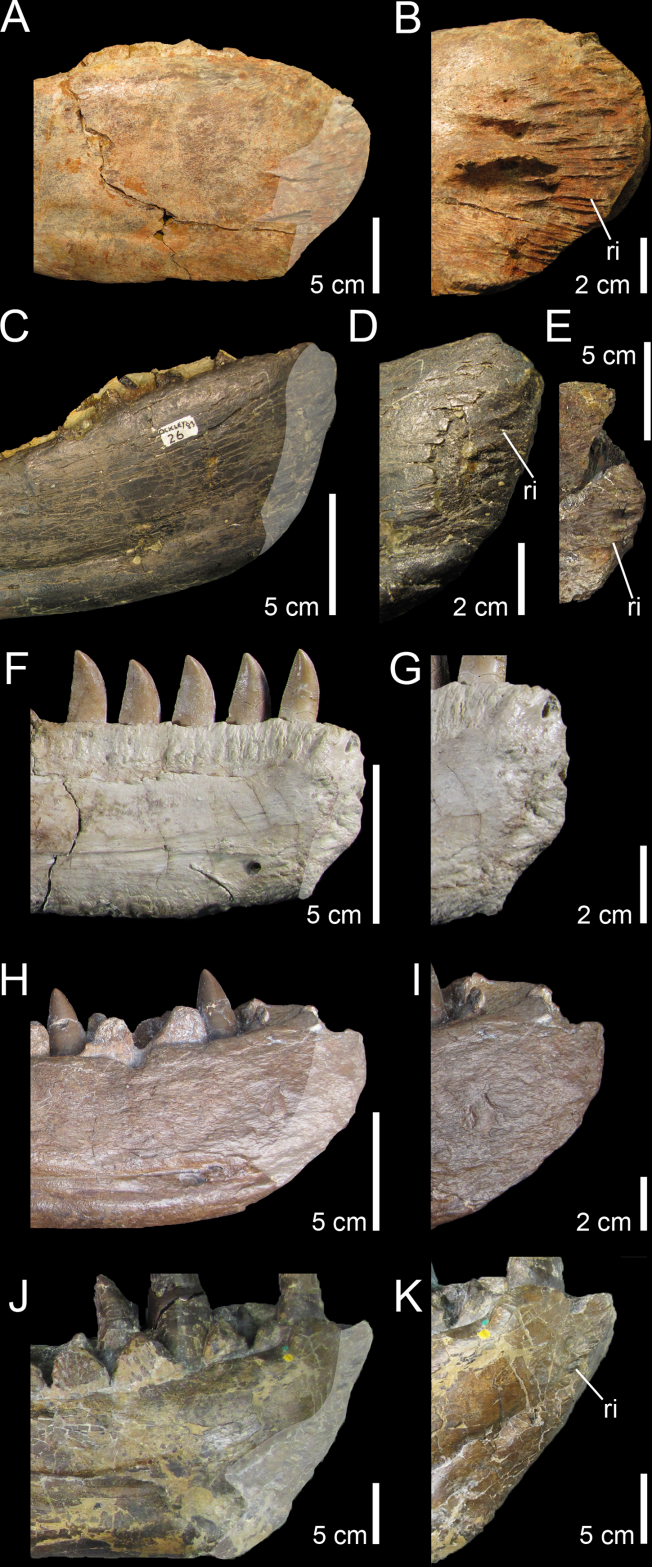
Morphological diversity of the mandibular symphysis in non-avian theropods in medial view. **A**–**B**, left dentary of *Spinosaurus* cf. *aegyptiacus* (NHM R.16421); **A**, Anterior portion; and **B**, close up on the well-developed anterior ridges of the mandibular symphysis. **C**–**E**, left dentary of *Baryonyx walkeri* (NHM R.9951 and ML 1190); **C**, anterior portion; and **D**–**E**, close up on the weakly developed anterior ridges of the mandibular symphysis in **D**, NHM R.9951; and **E**, ML 1190. **F**–**G**, right dentary of *Majungasaurus crenatissimus* (FMNH PR 2100; reversed); **F**, anterior portion; and **G**, close up on the irregular surface of the mandibular symphysis. **H**–**I**, right dentary of *Megalosaurus bucklandii* (OUMNH J13505; reversed); **H**, anterior portion; and **I**, close up on the smooth surface of the mandibular symphysis. **J**–**K**, right dentary of *Tyrannosaurus rex* (NHM R.7994); **J**, anterior portion; and **K**, close up on the poorly developed anterodorsal ridges of the mandibular symphysis. The symphyseal surface is colored in light grey. **Abbreviation**: **ri**, anteroposterior ridges of the mandibular symphysis.

Unlike pterosaurs and similar to pelecanids, lateral displacement of the mandibular rami was possible due to a movable mandibular symphysis of the dentaries in Spinosauridae [89,95,130,131]. A short mandibular symphysis has been noticed in *Baryonyx walkeri* ([89]; Fig 16C–16E) and *Spinosaurus aegyptiacus* [83,95], yet the anteroposterior length of the mandibular symphysis of *Spinosaurus* is not shorter, but actually longer than in other theropods (Fig 16A), as observed in *Suchomimus tenerensis* by Therrien et al. [46] (n.b., none of the *Suchomimus* dentaries that we examined (i.e., MNN G2-2, G5-1, G34-5, and G74-1) preserved the mandibular symphysis. Given the fact that the mandibular symphysis of the closely related taxon *Baryonyx* is particularly short (Fig 16C–16E), we cast doubt on the length of the mandibular symphysis measured by Therrien et al. [46] on a cast of *Suchomimus* dentary). Likewise, the symphyseal surface of the spinosaurid dentaries bears anteroposteriorly oriented striations suggesting that the mandibular rami were articulated by connective tissue (Fig 16A–16E; [89,95]). These ridges are short and only restricted to the anteriormost part of the dentary in *Baryonyx walkeri* (NHM R.9951; ML1190; Fig 16C–16E). Nevertheless, a dentary ascribed to *Spinosaurus* cf. *aegyptiacus* (NHM R.16421) shows strongly developed ridges covering the whole symphyseal surface of the dentary (Fig 16B). Symphyseal ridges in spinosaurids suggest the presence of connective tissues linking the two dentaries, allowing some lateromedial mobility of the mandibular rami [89,95]. This condition is once again exacerbated in *Spinosaurus aegyptiacus* in which these ridges are particularly numerous and prominent (Fig 16B). Such a peculiar morphology of the symphyseal surface is, to our knowledge, unique among theropods as non-spinosaurid theropods typically have a smooth or irregular symphyseal surface (Fig 16F–16K).

## Conclusion

The description and identification of six isolated quadrates, among which two most probably come from the Kem Kem beds of Morocco, provide additional information on the Cenomanian dinosaur fauna of North Africa. Based on cladistic, geometric morphometric, and phylogenetic morphometric analyses, two morphotypes have been successfully identified as belonging to two species of Spinosaurinae, and ascribed to *Spinosaurus aegyptiacus* and? *Sigilmassasaurus brevicollis*. This study provides the first convincing evidence of two spinosaurine taxa in the Cenomanian of North Africa based on cranial material, casting doubt on the recent reconstruction of a quadrupedal *Spinosaurus* which may be based on individuals belonging to two different species of Spinosaurinae.

Ontogenetic changes occurring in the spinosaurid quadrates include the suture of the quadrate and quadratojugal, delimitation of the mandibular condyles and squamosal capitulum, and development of a ventral projection of the dorsal quadratojugal contact and a second quadrate ridge ventral to the quadrate head. Based on the quadrate proportions and estimated skull length of *Baryonyx* and *Spinosaurus*, quadrates of mature individuals from Morocco belong to animals with a skull length of no more than 120 cm. This suggests that very large forms of *Spinosaurus* may have been rare in the Kem Kem assemblages.

Morphofunctional analysis of the spinosaurid quadrates has revealed peculiar jaw mechanics in these specialized theropods. An helicoidal and strongly lateromedially oriented joint of the jaw articulation allowed the lateral displacement of the mandibular ramus when the lower jaw was depressed. This lateral movement of the ramus was possible due to a movable mandibular symphysis as the dentaries were joined by connective tissues, and allowed the pharynx to be widened. A similar jaw articulation was convergently present in pterosaurs and particularly pelecanids which also have a mandibular symphysis restricted to the anterior extremity of the mandible. Spinosauridae, which are considered to be semi-aquatic and partially piscivorous animals, were able to swallow large prey such as fish in the same way as pelecanids.

## Supporting Information

S1 File Institutional abbreviations, comments on *Cristatusaurus lapparenti* (Figs A and B in S1 File), illustration of MHNM.KK377 and.KK378 (Fig C in S1 File), geological settings of the Kem Kem beds, quadrate-based diagnosis of a new species of Spinosaurinae tentatively referred to *Sigilmassasaurus brevicollis*, quadrate-based characters, list of taxa included in the cladistic analysis (Table A in S1 File), quadrate related datamatrix and supermatrix, and files of the cladistic, geometric morphometric, and phylogenetic morphometric analyses (TNT and MorphoJ files). (DOCX)
