# Supplementary material for: Morphofunctional Analysis of the Quadrate of Spinosauridae (Dinosauria: Theropoda) and the Presence of Spinosaurus and a Second Spinosaurine Taxon in the Cenomanian of North Africa
Source: PLoS One. 2016 Jan 6;11(1):e0144695. doi: 10.1371/journal.pone.0144695 (PMC4703214; doi:10.1371/journal.pone.0144695)
Supplement: S1 File — (DOCX) [file pone.0144695.s001.docx]

**Supplementary Information (S1 File)**

**Morphofunctional analysis of the quadrate of Spinosauridae (Dinosauria: Theropoda) and the presence of *Spinosaurus* and a second spinosaurine taxon in the Cenomanian of North Africa.**

**Christophe Hendrickx, Octávio Mateus, and Eric Buffetaut**

## Institutional abbreviations

**AMNH FARB**, American Museum of Natural History, collection of fossil reptiles, amphibians and birds, New York, New York, USA; **BHI**, Black Hills Institute, Hill City, South Dakota, USA; **BSPG**, Bayerische Staatssammlung für Pälontologie und Geologie, Munich ,Germany; **BYU-VP**, Brigham Young University Vertebrate Paleontology, Provo, Utah, USA; **FMNH PR**, Field Museum of Natural History, Chicago, Illinois, USA; **FSAC**, Faculté des Sciences Ain Chock, University of Hassan II Casablanca, Casablanca, Morocco; **GM**, Ganzhou Museum, Ganzhou City, Jiangxi Province, China; **GR**, Ghost Ranch Ruth Hall Museum of Paleontology, Ghost Ranch, New Mexico, USA; **IGM**, Institute of Geology, Ulaan Baatar, Mongolia; **IVPP**, Institute for Vertebrate Paleontology and Paleoanthropology, Beijing, China; **MACN-CH**, Museo Argentino de Ciencias Naturales “Bernardino Rivadavia”, Buenos Aires, Argentina; **MCNA-PV**, Museo de Ciencias Naturales y Antropológicas (J. C. Moyano) de Mendoza, Mendoza, Argentina; **MCF-PVPH**, Museo Municipal Carmen Funes, Paleontologia de Vertebrados, Plaza Huincul, Argentina; **MHNM**, Muséum d’Histoire Naturelle de Marrakech, Marrakech, Morocco; **MIWG**, Dinosaur Isle, Isle of Wight Museum Services, Sandown, United Kingdom; **ML**, Museu da Lourinhã, Lourinhã, Portugal; **MNHN**, Muséum national d’Histoire Naturelle, Paris, France; **MNN**, Musée National du Niger, Niamey, Niger; **MPCA**, Museo Provincial Carlos Ameghino, Cipolletti, Río Negro, Argentina; **MSNM**, Museo di Storia Naturale di Milano, Milan, Italy; **MUCPv-CH**, Museo de Ciencias Naturales de la Universidad Nacional de Comahue, El Chocón collection, Villa El Chocón, Argentina; **MWC**, Museum of Western Colorado, Fruita (or Grand Junction), Colorado, USA; **NCSM**, North Carolina Museum of Natural Sciences, Raleigh, North Carolina, USA; **NHM**, The Natural History Museum, London, United Kingdom; **OUMNH**, Oxford University Museum, Oxford, UK; **PIN**, Paleontological Institute of the Russian Academy of Sciences, Moscow, Russia; **PVSJ**, Museo de Ciencias Naturales, Universidad Nacional de San Juan, San Juan, Argentina; **SMA**, Sauriermuseum Aathal, Aathal, Switzerland; **SMNS**, Staatliches Museum für Naturkunde, Stuttgart, Germany; **UCMP**, University of California Museum of Paleontology, Berkeley, California, USA; **UMNH VP**, Utah Museum of Natural History, University of Utah, Salt Lake City, Utah, USA.

## Comments on *Cristatusaurus lapparenti* from the Lower Cretaceous of Niger

*Cristatusaurus lapparenti* [1] and *Suchomimus tenerensis* [2] are two baryonychine taxa from the Elrhaz Formation (Aptian‒Albian?, Lower Cretaceous; [3]) of Gadoufaoua, Niger, that were erected in 1998. *Cristatusaurus lapparenti* is currently considered to be a valid species and a senior synonym of *Suchomimus tenerensis* by Allain [4], a junior synonym of *Baryonyx* by Charig and Milner [5], Buffetaut and Ouaja [6] and Sues et al. [7], an indeterminate Baryonychinae by Bertin [8], and a *nomen dubium* by Sereno et al. [2], Rauhut [9], and Carrano et al. [10]. Because *Cristatusaurus* and *Suchomimus* come from the same sites and deposits, Kellner et al. [11] and Carrano et al. [10] also consider that these two taxa are possibly congeneric/conspecific and almost certainly represent the same animal, respectively. In the latter case, if the diagnosis given to define *Cristatusaurus lapparenti* is accepted as valid by the scientific community, *Cristatusaurus lapparenti* would have priority over *Suchomimus tenerensis* as the article describing the former was published in September 1998, two months before that of *Suchomimus*, published on 13^th^ November 1998.

The diagnosis given by Taquet and Russell [1] to define *Cristatusaurus* is nonetheless considered by many paleontologists as uninformative and/or insufficient to distinguish it from *Baryonyx*. Taquet and Russell [1], indeed, differentiate *Cristatusaurus* from *Baryonyx* on the basis of its ‘brevirostrine condition’ (i.e., a short rostrum). The authors consider that the *Cristatusaurus* premaxilla is short (which would indeed differ from the anteroposteriorly long premaxilla of *Baryonyx*), possibly due to the confusion between the deep maxillary notch of the *Cristatusaurus* premaxilla (Figs A:A, B:C) and the external naris, which is clearly visible in *Baryonyx* and *Suchomimus* (Fig A:G, J; Fig B:A‒B, D). Given that both premaxillae of *Cristatusaurus* are incomplete and only preserved the anterior portion of the bone, it is very likely that the premaxilla was as long in *Cristatusaurus* as in *Baryonyx*. We, therefore, agree with most paleontologists and consider that the differential diagnosis provided by Taquet and Russell [1] to distinguish *Cristatusaurus* from *Baryonyx* is uninformative.

A deeper look at the baryonychine cranial material, however, allows us to note some similarities between *Cristatusaurus* and *Suchomimus* premaxillae, and some differences between the premaxilla of these two taxa and *Baryonyx*. In palatal view, the second alveolus is significantly larger than the first one in *Cristatusaurus* and *Suchomimus* whereas it is smaller than the first alveolus in *Baryonyx* (Fig A:B, H, K). Likewise, the three posteriormost alveoli (alveoli 5, 6 and 7) share the same size in the two African baryonychines, while the fourth/fifth alveolus is significantly larger than the last two in *Baryonyx* (Fig A:B, H, K). In lateral and dorsal views, the portion of the premaxilla dorsal to the the maxillary notch is strongly lateromedially narrows dorsally in *Cristatusaurus* and *Suchomimus*, forming what has been interpreted as a posterior crest by Taquet & Russell [1] in *Cristatusaurus*. This dorsal narrowing of the premaxillary body is due to the deep, ventrodorsally tall and anterodorsally long narial fossa, which extends anteriorly from the external nares all along the posterior half of the premaxillary body in *Suchomimus* and *Cristatusaurus*. Yet, the *Suchomimus* snout indeed shows a low and poorly developed premaxillary crest with a rugose lateral surface and an ondulated posterior margin. This premaxillary crest is restricted to the posterior portion of the premaxillary body and the anterior part of the nasal process of the premaxilla (Fig B:A). A premaxillary crest was already described in the spinosaurine *Angaturama limai* [12] and most likely results from a deep and extensive narial fossa on the premaxillary body as well. As noted by Carrano et al. [10], the premaxillary crest (the ‘midline ridge on dorsal surface of conjoined premaxillae’ *sensu* [10]) extends further anteriorly, and almost up to the anterior extremity of the premaxilla in *Angaturama*. Both narial fossa and premaxillary crest are absent in *Baryonyx*, so that the lateromedial narrowing of the dorsal part of the premaxillary body is not as important as in *Suchomimus* and *Cristatusaurus*. Mortimer [13] also adds to the similarities between *Cristatusaurus* and *Suchomimus* the curvature of the anterodorsal margin of the premaxilla, which is less rounded than that of *Baryonyx* in lateral view. Due to the premaxillary crest, the curvature of the anterodorsal margin of the premaxilla body is, indeed, wider in *Suchomimus* than in *Baryonyx* (Fig B:B), as it forms a large convexity from the anteriormost point of the premaxilla to the anterior part of the nasal process of the premaxilla in *Suchomimus*. In *Baryonyx*, this convexity is restricted to the anterior part of the premaxillary body so that the dorsal margin of the nasal process of the premaxilla is straight and not sigmoid as in *Suchomimus* (Fig B:E). Yet, given the incompleteness of both specimens of premaxillae, it is unknown whether *Cristatusaurus* had a curvature of the anterodorsal margin of the premaxilla similar to that of *Suchomimus*. The curvature of the anterior margin of the premaxilla is at least almost exactly the same in *Cristatusaurus* (MNHN GDF365; Fig B:D), *Suchomimus* (MNN GAD501; Fig B:B) and *Baryonyx* (NHM R.9951; Fig B:E). The anterodorsal margin of the premaxilla is nonetheless strongly posteriorly inclined in the immature specimen of *Cristatusaurus* (MNHN GDF366; Figs A:A, B:C) and in one specimen of *Suchomimus* (MNN GADG-6; Fig A:D), but this feature obviously varies ontogenetically and/or intraspecifically.

Despite the numerous similarities between the *Cristatusaurus* and *Suchomimus* premaxillae, we could not find a definitive apomorphic character diagnosing *Cristatusaurus lapparenti*. Indeed, the size (as the number) of the premaxillary alveoli, the depth and extension of the nasal fossa, and the development of a premaxillary crest may all vary ontogenetically and intraspecifically, or may result from sexual dimorphism. We, therefore, tentatively consider *Cristatusaurus* *lapparenti* as a Baryonychinae *nomen dubium* pending a deeper examination of the postcranial material of this taxon. Given the apomorphic characters in the cranial and postcranial skeleton (see Carrano et al. [10]), we, however, consider *Suchomimus tenerensis* as a valid taxon.


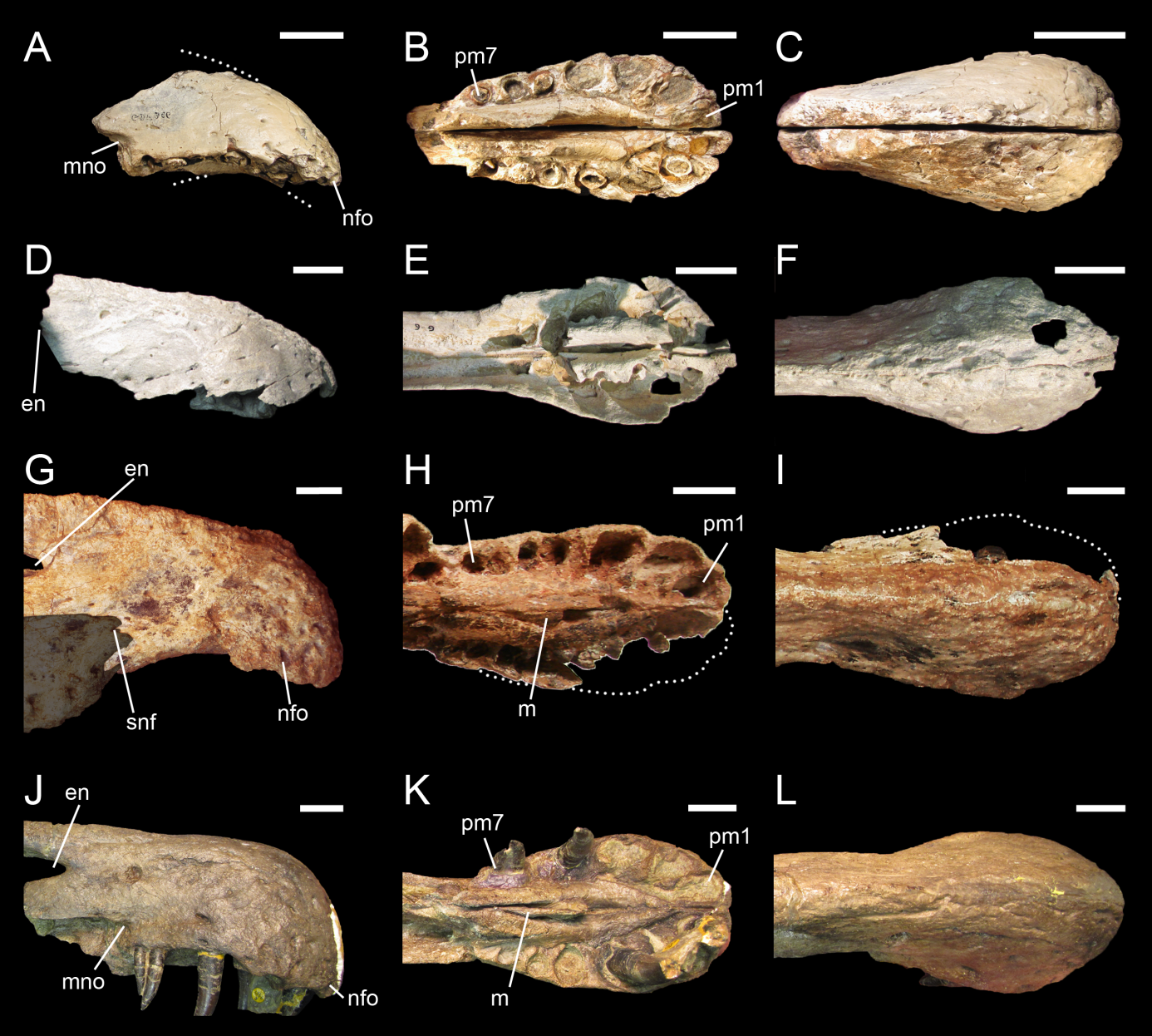


**Figure A. Articulated premaxillae of Baryonychinae in A, D, G, J, lateral, B, E, H, K, palatal, and C, F, I, L, dorsal views.** **A‒C**, *Cristatusaurus lapparenti* (MNHN GDF366; **A** left reversed); **D‒I**, *Suchomimus tenerensis* (**D‒F**, MNN GADG-6; **G‒I**, MNN GAD501; photos courtesy shared by Roger Benson for **G**, and Juan Canale for **H** and **I**); and **J‒L**, *Baryonyx walkeri* (NHM R.9951). **Abbreviations**: **en**, external naris; **m**, maxilla; **mno**, maxillary notch of the premaxilla; **nfo**, neurovascular foramina; **pm1**, first premaxillary alveolus; **pm7**, seventh premaxillary alveolus; **snf**, subnarial foramen. Scale bar = 3 cm.


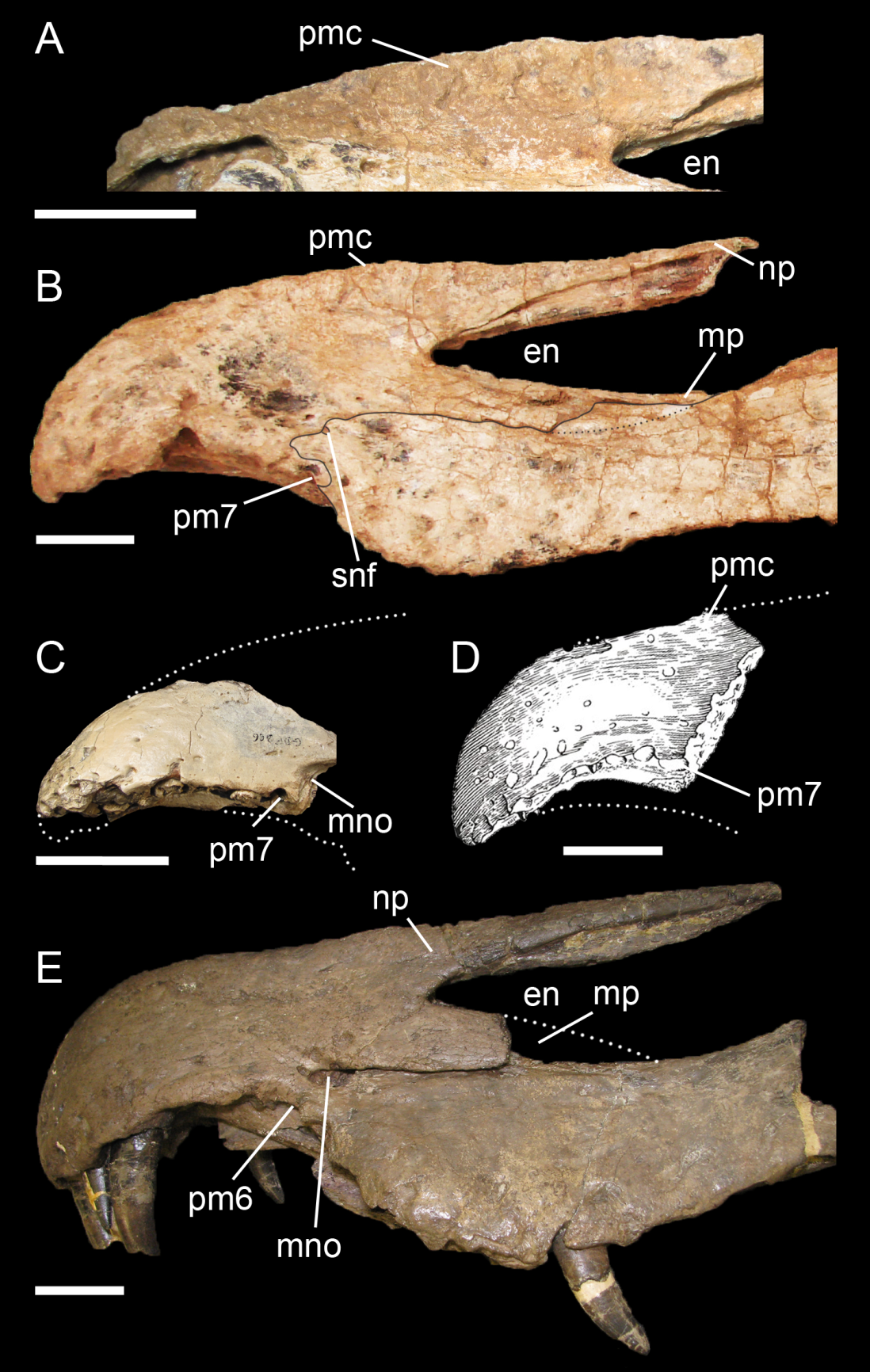


**Figure B. Baryonychine rostrum in lateral view.** **A‒B**, *Suchomimus tenerensis* (MNN GAD501); **A**, close up on the premaxillary crest (left side); and **B**, snout (right reversed, photo courtesy shared by Juan Canale); **C‒D**, *Cristatusaurus lapparenti* (**C**, MNHN GDF566; **D**, MNHN GDF565, modified from Taquet [14]); **E**, *Baryonyx walkeri* (NHM R.9951). **Abbreviations**: **en**, external naris; **mno**, maxillary notch of the premaxilla; **mp**, maxillary process of the premaxilla; **nfo**, neurovascular foramina; **np**, nasal process of the premaxilla; **pm1**, first premaxillary alveolus; **pm6**, sixth premaxillary alveolus; **pm7**, seventh premaxillary alveolus; **pmc**, premaxillary crest; **snf**, subnarial foramen. Scale bar = 5 cm.

## Illustration of MHNM.KK377 and .KK378


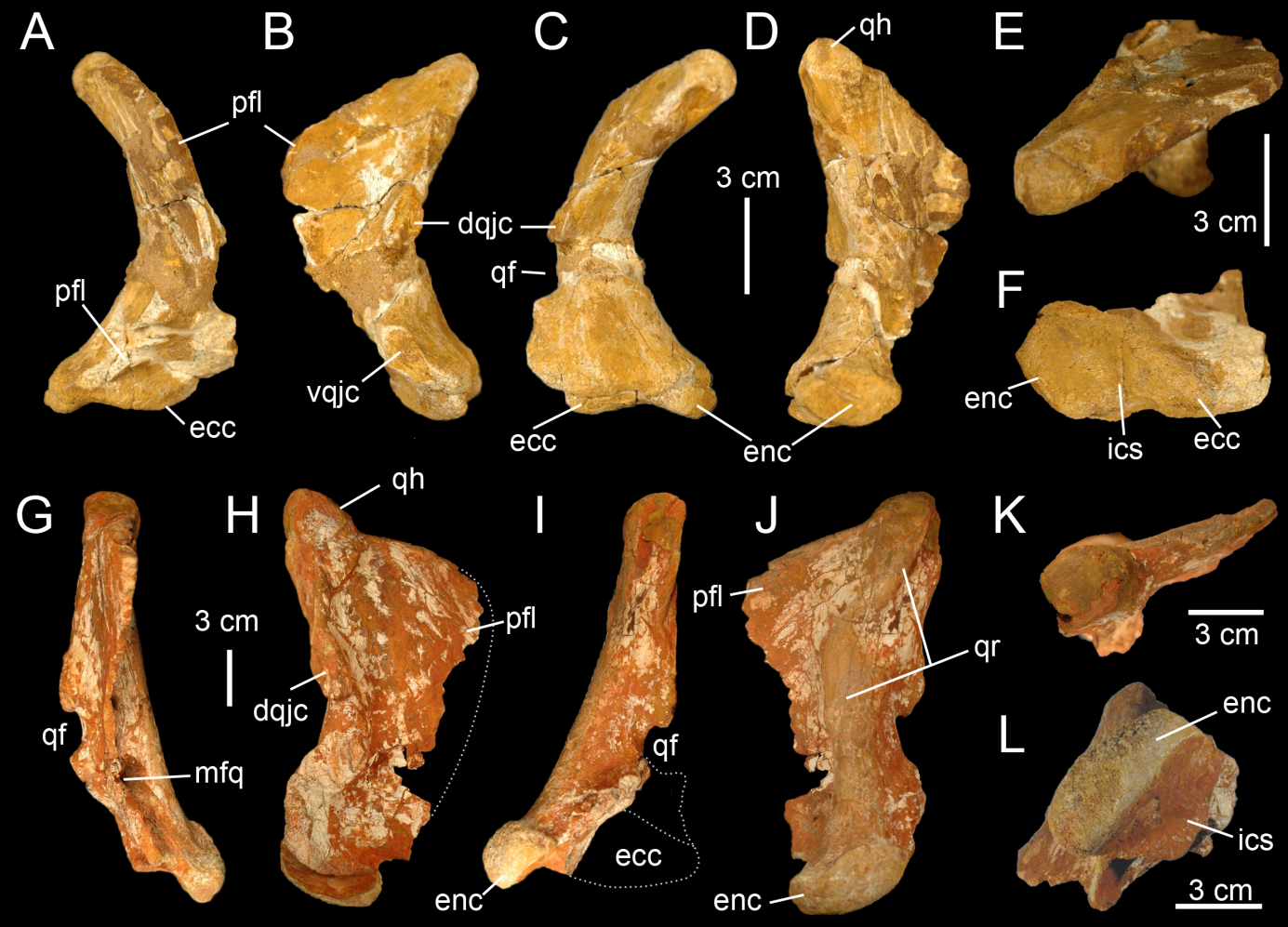


**Figure C. Quadrates of Morphotype 1 referred to *Spinosaurus aegyptiacus*.** **A‒N**, Left quadrates of specimen MHNM.KK377 and right quadrate of specimen MHNM.KK378 in **A, G**, anterior; **B, H**, lateral; **C, I**, posterior; **D**, medial; **J**, posteromedial; **E, K**, dorsal; and **F, L**, ventral views, with reconstructed parts in dotted line. **Abbreviations**: **dqjc**, dorsal quadratojugal contact; **ecc**, ectocondyle; **enc**, entocondyle, **ics**, intercondylar sulcus; **mfq**, medial fossa; **pfl**, pterygoid flange; **qf**, quadrate foramen; **qh**, quadrate head; **qr**, quadrate ridge; **vqjc**, ventral quadratojugal contact.

## Geological settings of the Kem Kem beds

The Kem Kem is a vast tabular rocky plateau (or hamada) bounding the Algerian boundary in south-eastern Morocco. This region has yielded a large number of vertebrate remains, all originating from a continental unit referred as the Kem Kem beds [15] (Fig 1A). Formerly known as the ‘Continental Intercalaire’ (sensu Kilian [16]; [17,18]), the Kem Kem beds form a 150 to 200 meters thick continental succession exposed along the face of a limestone-capped escarpment, which extends some 250 km in length along, or close by, the Algerian border of Morocco (e.g., [17,19–22]; Fig 1A). The Kem Kem beds overlie Paleozoic (Cambrian to Silurian) sediments unconformably, and are currently dated to the Cenomanian [15,20,23]. They comprise fluvial to coastal deposits typically divided into two main units, the Ifezouane and Aoufous formations [20,24] (Fig 1B).

The Ifezouane Formation, which corresponds to the ‘Grès Infracénomaniens’ of Choubert [25], the ‘Grès rouges infracénomaniens’ of Joly [26] and possibly the ‘lower unit’ of Sereno et al. [15], consists of reddish detritic sandstones with cross-stratified structures alternating with pinkish sands, occasional conglomeratic layers of quartz pebbles, and some calcareous layers containing bivalves and gastropods [20,24,26,27]. The thickness of this unit is extremely variable (from 0 to 250 meters) and typically decreases from the south to the north [20,25]. The base of the Ifezouane Formation is made of conglomerates and breccia intercalated with poorly cemented reddish sandstones, whereas the upper part comprises finer-grained, better calibrated, and uniform sandstones with lighter colors that can even go towards yellow [26]. According to Belvedere et al. [21], the reddish fluvial sandstones are organized in meter-thick beds, and show high-angled cross-bedding, representative of channel belt successions, with subordinate intercalations of pedogenized overbank mudstones. This facies was interpreted by the authors "as a braided fluvial system [and] a fully continental depositional environment characterized by high-energy deposits" ([21], p. 53). The Ifezouane Formation is the richest unit in terms of skeletal elements and has yielded articulated specimens of *Spinosaurus*, *Deltadromeus* and *Rebbachisaurus* [20,28] (Fig 1B).

The Aoufous Formation, also known as the ‘Marnes à gypses cénomaniennes’ [25] and the ‘Marnes versicolores à gypse’ [26], and possibly corresponding to the ‘upper unit’ [15], varies in thickness from 100 to 200 meters [20]. This unit is mostly comprised of multi-colored mudstones and marls, with intercalations of thinly-bedded detritic sandstones, microconglomerates, calcitic palaeosols, thin intercalations of evaporites (fibrous and saccharoïd gypsum), and yellowish dolomitic layers [20–22,24,26]. According to Belvedere et al. [21] and Cavin et al. [20], this facies association represents a coastal lagoon/mudflat deposited in a medio- to supralittoral paleoenvironment, and a low-energy environment close to the shoreline with local development of sabkhas and marginal ponds of water. The sandstone layers of the Aoufous Formation have yielded a large diversity of theropod footprints [15,21,22] (Fig 1B), and skeletal remains from the Aoufous Formation are mostly comprised of *Onchopristis* teeth (Cavin pers. comm.). According to Cavin et al. [20], vertebrate remains are only abundant in the Aoufous Formation in the northern part of the Kem Kem, such as in the Douira locality, north of Erfoud. Yet fossiliferous levels from Douira essentially include microfossils, and classical vertebrate remains found in this locality most likely come from the upper part of the Ifezouane Formation (Cavin pers. comm.). Nevertheless, given the presence of some dinosaur remains, such as the *Carcharodontosaurus* cranial material described by Sereno et al. [15] and Brusatte and Sereno [29], in the upper unit defined by Sereno et al. [15], the Aoufous Formation seems also to yield large vertebrate remains. It is, however, possible that the upper and lower units defined by Sereno et al. [15] do not exactly correspond to the Aoufous and Ifezouane formations (Cavin pers. comm.), and that the thick layer of sandstone which yielded *Carcharodontosaurus* remains (Sereno et al. [15]: figure 1C) may in fact be considered as belonging to the Ifezouane Formation by Cavin et al. [20].

**References**

1. Taquet P, Russell DA. New data on spinosaurid dinosaurs from the Early Cretaceous of the Sahara. Comptes Rendus de l’Académie des Sciences-Series IIA-Earth and Planetary Science. 1998;327: 347–353.

2. Sereno PC, Beck AL, Dutheil DB, Gado B, Larsson HCE, Lyon GH, et al. A long-snouted predatory dinosaur from Africa and the evolution of spinosaurids. Science. 1998;282: 1298–1302. doi:10.1126/science.282.5392.1298

3. Sereno PC, Brusatte SL. Basal abelisaurid and carcharodontosaurid theropods from the Lower Cretaceous Elrhaz Formation of Niger. Acta Palaeontologica Polonica. 2008;53: 15–46.

4. Allain R. Les Megalosauridae (Dinosauria, Theropoda). Nouvelle découverte et révision systématique: implications phylogénétiques et paléobiogéographiques. Ph.D. dissertation, Muséum National d’Histoire Naturelle, Paris. 2002.

5. Charig AJ, Milner AC. *Baryonyx walkeri*, a fish-eating dinosaur from the Wealden of Surrey. Bulletin of the Natural History Museum. 1997;53: 11–70.

6. Buffetaut E, Ouaja M. A new specimen of *Spinosaurus* (Dinosauria, Theropoda) from the Lower Cretaceous of Tunisia, with remarks on the evolutionary history of the Spinosauridae. Bulletin de la Societe Geologique de France. 2002;173: 415–421. doi:10.2113/173.5.415

7. Sues H-D, Frey E, Martill DM, Scott DM. *Irritator challengeri*, a spinosaurid (Dinosauria: Theropoda) from the Lower Cretaceous of Brazil. Journal of Vertebrate Paleontology. 2002;22: 535–547. doi:10.1671/0272-4634(2002)022[0535:ICASDT]2.0.CO;2

8. Bertin T. A catalogue of material and review of the Spinosauridae. PalArch’s Journal of Vertebrate Palaeontology. 2010;7: 1–39.

9. Rauhut OWM. The interrelationships and evolution of basal theropod dinosaurs. Special Papers in Palaeontology. 2003;69: 1–213.

10. Carrano MT, Benson RBJ, Sampson SD. The phylogeny of Tetanurae (Dinosauria: Theropoda). Journal of Systematic Palaeontology. 2012;10: 211–300.

11. Kellner AW, Azevedo SA, Machado EB, Carvalho LB de, Henriques DD. A new dinosaur (Theropoda, Spinosauridae) from the Cretaceous (Cenomanian) Alcântara Formation, Cajual Island, Brazil. Anais da Academia Brasileira de Ciências. 2011;83: 99–108.

12. Kellner AWA, Campos DA. First Early Cretaceous theropod dinosaur from Brazil with comments on Spinosauridae. Neues Jahrbuch für Geologie und Paläontologie - Abhandlungen. 1996;199: 151–166.

13. Mortimer M. *Cristatusaurus* Taquet and Russell, 1998. In: The Theropod Database [Internet]. 2015 [cited 13 May 2015]. Available: http://theropoddatabase.com/Megalosauroidea.htm#Cristatusauruslapparenti

14. Taquet P. Une curieuse spécialisation du crâne de certains dinosaures carnivores du Crétacé: le museau long et étroit des spinosauridés. Comptes-rendus des séances de l’Académie des sciences Série 2, Mécanique-physique, chimie, sciences de l’univers, sciences de la terre. 1984;299: 217–222.

15. Sereno PC, Dutheil DB, Larochene M, Larsson HCE, Lyon GH, Magwene PM, et al. Predatory dinosaurs from the Sahara and Late Cretaceous faunal differentiation. Science. 1996;272: 986–991. doi:10.1126/science.272.5264.986

16. Kilian C. Des principaux complexes continentaux du Sahara. Comptes Rendus sommaires de la Société géologique de France. 1931;9: 109–111.

17. Lavocat R. Reconnaissance géologique dans les Hammadas des confins algéro-marocains du Sud. Notes et Mémoires du Service Géologique du Maroc. 1954;116: 1–147.

18. De Lapparent AF. Les dinosauriens du “Continental Intercalaire” du Sahara Central. Mémoire de la Société Géologique de France. 1960;88A: l–57.

19. Russell DA. Isolated dinosaur bones from the Middle Cretaceous of the Tafilalt, Morocco. Bulletin du Muséum National d’Histoire Naturelle. 1996;4: 349–402.

20. Cavin L, Tong H, Boudad L, Meister C, Piuz A, Tabouelle J, et al. Vertebrate assemblages from the early Late Cretaceous of southeastern Morocco: an overview. Journal of African Earth Sciences. 2010;57: 391–412.

21. Belvedere M, Jalil N-E, Breda A, Gattolin G, Bourget H, Khaldoune F, et al. Vertebrate footprints from the Kem Kem beds (Morocco): A novel ichnological approach to faunal reconstruction. Palaeogeography, Palaeoclimatology, Palaeoecology. 2013;383–384: 52–58. doi:10.1016/j.palaeo.2013.04.026

22. Ibrahim N, Varricchio DJ, Sereno PC, Wilson JA, Dutheil DB, Martill DM, et al. Dinosaur footprints and other ichnofauna from the Cretaceous Kem Kem Beds of Morocco. PLoS ONE. 2014;9: e90751. doi:10.1371/journal.pone.0090751

23. Dutheil DB. An overview of the freshwater fish fauna from the Kem Kem beds (Late Cretaceous: Cenomanian) of southeastern Morocco. Mesozoic fishes. 1999;2: 553–563.

24. Ettachfini EM, Andreu B. Le Cénomanien et le Turonien de la Plate-forme Préafricaine du Maroc. Cretaceous Research. 2004;25: 277–302. doi:10.1016/j.cretres.2004.01.001

25. Choubert G. Essai sur la paléogéographie du Mésocrétacé marocain. Volume Jubilaire de la Société des Sciences Naturelles du Maroc. 1948; 307–329.

26. Joly F. Études sur le relief du Sud-Est marocain. Travaux de l’Institut Scientifique Chérien Série géologie et géographie physique. 1962;10: 1–578.

27. Abramovich S, Keller G, Adatte T, Stinnesbeck W, Hottinger L, Stueben D, et al. Age and paleoenvironment of the Maastrichtian to Paleocene of the Mahajanga Basin, Madagascar: a multidisciplinary approach. Marine Micropaleontology. 2003;47: 17–70. doi:10.1016/S0377-8398(02)00094-4

28. Ibrahim N, Sereno PC, Sasso CD, Maganuco S, Fabbri M, Martill DM, et al. Semiaquatic adaptations in a giant predatory dinosaur. Science. 2014;345: 1613–1616. doi:10.1126/science.1258750

29. Brusatte SL, Sereno PC. A new species of *Carcharodontosaurus* (Dinosauria: Theropoda) from the Cenomanian of Niger and a revision of the genus. Journal of Vertebrate Paleontology. 2007;27: 902–916. doi:10.1671/0272-4634(2007)27[902:ANSOCD]2.0.CO;2

## Quadrate-based diagnosis of a new species of Spinosaurinae tentatively referred to *Sigilmassasaurus brevicollis*

?*Sigilmassasaurus brevicollis* Russel, 1996

**Referred material**—MHNM.KK376, the ventral portion of a right quadrate (Fig 3).

**Occurrence**—Near the town of Jorf, northwest of Erfoud, Tafilalt, Morocco. Ifezouane Formation?, Kem Kem beds, Lower? Cenomanian, Upper Cretaceous.

**Quadrate-based diagnosis**—Megalosauroid theropod possessing the following quadrate-related unambiguous autapomorphies: deep and well-delimited depression on the anterior surface of the ectocondyle; sigmoid, strongly lateromedially elongated and anteroposteriorly short crest-like ectocondyle. This species is also diagnosed by the following unique combination of characters: minute quadrate foramen; subtrapezoidal ventral quadratojugal contact of the quadrate showing a punctuated surface; dorsal and ventral quadratojugal contacts of the quadrate with similar anteroposterior length in lateral view; straight and diagonally oriented ventral quadratojugal contact of the quadrate not extending on the whole surface of the ectocondyle.

## Quadrate based characters

I. QUADRATE

1. Quadrate, ventrodorsal elongation (ratio between the lateromedial width of the mandibular articulation and the ventrodorsal height of the quadrate body): (0) strongly elongated, <0.35; (1) moderately elongated, 0.35-0.5; (2) short, >0.5 (**Ordered**; Based on Currie & Carpenter, 2000 #20)
2. Quadrate, position of the quadrate head relative to the orbit height: (0) positioned at 80% or less of the orbit height; (1) positioned at more than 80% of the orbit height (Modified from Sereno et al. 1994)
3. Quadrate, position of the mandibular articulation relative to the quadrate head, when the quadrate is articulated within the cranium: (0) entirely posterior; (1) approximately aligned; (2) entirely anterior (**Ordered**; Modified from Gauthier, 1986)
4. Quadrate, ventral extension relative to the alveolar margin of the maxilla in lateral view: (0) projects well-ventral to the alveolar margin of the maxilla; (1) level with the alveolar margin of the maxilla; (2) well-dorsal to the alveolar margin of the maxilla (**Ordered**; Holtz, 1994)

II. QUADRATE BODY

*Margins*

1. Quadrate body, outline of the posterior margin in lateral view (mandibular articulation and quadrate head excluded): (0) strongly concave; (1) roughly straight; (2) convex; (3) sigmoid, convex dorsally and straight or concave ventrally; (4) sigmoid, concave dorsally and convex ventrally (Unordered; New)
2. Quadrate body, posterior surface at one half of the quadrate height in posterior view: (0) lateromedially concave; (1) lateromedially convex (New)
3. Quadrate body, outline of the ventromedial extremity in posterior view: (0) rounded; (1) angular (New)
4. Quadrate body, shallow groove on the posterior surface, medial to the quadrate foramen and extending on the first third of the quadrate height: (0) absent; (1) present (New)
5. Quadrate body, protuberant ridge dorsal to the ectocondyle, at one fourth of the quadrate height: (0) absent; (1) present (New)

*Quadrate ridge*

1. Quadrate ridge, shape at mid-height of the quadrate in posterior view: (0) prominent and well-delimited; (1) shallow and poorly delimited; (2) ridge absent (Unordered; New)
2. Quadrate ridge at mid-height of the quadrate, shape in posterior view (ratio: lateromedial width of ridge/lateromedial width of quadrate body, pterygoid excluded): (0) narrow crest (< 0.1); (1) rod-shaped (0.1-0.7); (2) very broad shaft (>0.7). (**Ordered**; New)
3. Quadrate ridge, lateroposterior inclination in posterior view: (0) present; (1) absent, subvertical ridge (New)
4. Quadrate ridge, ventral extension in posterior view: (0) extending well-dorsal to the entocondyle; (1) extending directly dorsal to the entocondyle; (2) reaching the entocondyle (Unordered; New)
5. Quadrate ridge, dorsal extension in posterior view: (0) extending to the quadrate head or directly ventral to it; (1) extending at two third of the quadrate height; (2) extending at mid-height of the quadrate (Unordered; New)
6. Quadrate ridge, bifurcation of the ventral extremity: (0) absent; (1) present (New)
7. Quadrate ridge, separation of the ridge at two-third of the quadrate height in dorsal view: (0) absent, ridge unique; (1) present, groove separating the ridge; (2) present, ridge fading away at two-third of the quadrate height and reappearing more dorsally (Unordered; New)
8. Quadrate ridge, protuberance at two third of the quadrate height in lateral view: (0) absent; (1) present (**New**)

III. MANDIBULAR ARTICULATION

*General shape*

1. Mandibular articulation, ratio between the lateromedial width and the anteroposterior length (perpendicular and at midlength): (0) <2; (1) 2-3; (2) 3-4; (3) >4 (**Ordered**; New)
2. Mandibular articulation, number of condyles: (0) two; (1) three (Chiappe, 2001 #21)
3. Mandibular articulation, step between the mandibular condyles and the quadrate body in lateral view: (0) absent; (1) present and weak, limit between mandibular condyles and quadrate body shallowly concave; (2) present and important, limit between mandibular condyles and quadrate body deeply concave (**Ordered**; New)
4. Mandibular condyles, ventral margin in posterior view: (0) biconvex, limit between the two condyles angular or slightly concave; (1) biconvex, very large concavity separating the two condyles; (2) W-shaped, ventral margin of condyles roughly flattened and angular roughly convex (Unordered; New)
5. Mandibular condyles, posterior margin in ventral view: (0) strongly biconvex; (1) very slightly biconvex, almost uniquely convex (New)
6. Mandibular condyles, size in ventral view (ratio: longest length ectocondyle/longest length entocondyle): (0) longer entocondyle (<0.9); (1) subequal in size (0.9-1.1); (2) longer ectocondyle (1.1-1.9); (3) much longer ectocondyle (>1.9). (**Ordered**; New)
7. Mandibular condyles, intercondylar notch in between the ecto- and entocondyles: (0) absent; (1) present on the anterior margin of the mandibular articulation; (2) present on the posterior margin of the mandibular articulation (Unordered; New)

*Ectocondyle*

1. Ectocondyle, ratio: width/length in ventral view: (0) >0.55, oval to subcircular; (1) 0.3-0.55, elliptical; (2) 0.3-0.55, parabolic; (3) 0.3-0.1, parabolic to weakly sigmoid; (4) <0.1 strongly sigmoid and almost forming a crest (**Ordered**; New)
2. Ectocondyle, concavity on the anterior side in anterior view: (0) absent; (1) present, shallow and poorly delimited; (2) present, deep and well-delimited (Unordered; New)
3. Ectocondyle, ventral margin in anterior view: (0) convex; (1) sigmoid (New)
4. Ectocondyle, extension of the articular surface on the posterior surface of the quadrate body (ratio: width/length of articular surface in posterior view): (0) limited, <0.3; (1) moderately extended, 0.3-0.5; (2) important, >0.5 (Unordered; New)

*Entocondyle*

1. Entocondyle, ratio: width/length in ventral view: (0) >0.4, oval to subcircular; (1) 0.3-0.4, elliptical and moderately elongated; (2) <0.3, elliptical and strongly elongated (**Ordered**; New)
2. Entocondyle, shape in ventral view: (0) not protruding anteriorly, or very slightly; (1) strongly protruding anteriorly (New)
3. Entocondyle, extension of the articular surface on the quadrate body (ratio: width/length of articular surface in posterior view): (0) <0.25, limited; (1) 0.25-0.6, moderately extent; (2) >0.6, important (Unordered; New)

*Intercondylar sulcus*

1. Intercondylar sulcus in ventral view: (0) well-delimited by the mandibular condyles; (1) shallow (New)
2. Intercondylar sulcus in ventral view: (0) narrow, narrower than the entocondyle width; (1) wide, same width or larger than the entocondyle width (New)
3. Intercondylar sulcus, angle between main axis of sulcus and long axis of mandibular articulation in ventral view: (0) >135°; (1) <135° (New)

IV. QUADRATE HEAD

1. Quadrate head in articulation within the skull, exposure in lateral view: (0) quadrate head entirely or almost entirely exposed; (1) quadrate head partially exposed; (2) quadrate head completely obscured (Unordered; Sereno and Novas 1994)
2. Quadrate head size relative to mandibular articulation (ratio: mediolateral width of quadrate head/mediolateral width of mandibular articulation in posterior view): (0) >0.31; (1) 0.29-0.31; (2) 0.28-0.24; (3) < 0.24 (Unordered; New)
3. Quadrate head, shape in dorsal view: (0) one single condyle, the squamosal capitulum; (1) two slightly differentiated condyles on the top of the columnar body of the quadrate; (2) two very distinct condyles, one large and laterally positioned, the squamosal capitulum, and one smaller and ventromedially positioned, the otic capitulum (Unordered; Modified from Gauthier, 1986 and Chiappe, 1995)
4. Quadrate head (otic and squamosal capitula included), shape in dorsal view: (0) subtriangular; (1) oval or subcircular; (2) subquadrangular to subrectangular (Unordered; Modified from Sereno et al. 1998 #27)
5. Quadrate head, outline in posterior view: (0) convex or roughly flattened quadrate head; (1) strongly convex, conical and pointed quadrate head; (2) concave (Unordered; New)

V. CONTACTS

*Lateral contact, general shape*

1. Laterodorsal contact in lateral view: (0) only or mostly contacting quadratojugal; (1) mostly contacting squamosal; (2) contacting postorbital and squamosal (Unordered; New)
2. Lateral contacts, ratio: anteroposterior width of dorsal contact/anteroposterior width of ventral contact in lateral view: (0) <0.2; (1) 0.2-0.5; (2) >0.5 (Unordered; New)

*Dorsal quadratojugal/squamosal/postorbital contact*

1. Dorsal contact, shape in lateral view: (0) elongated line; (1) drop-shaped; (2) drop-shaped reversed; (3) elliptical; (4) subrectangular (Unordered; New)
2. Dorsal contact: (0) facing anteriorly; (1) facing laterally; (2) facing posterolaterally or completely posteriorly (Unordered; New)
3. Dorsal contact, surface: (0) roughly smooth; (1) irregular; (2) with two longitudinal furrows separated by a ridge; (3) with one longitudinal furrow (Unordered; New)
4. Dorsal contact, delimitation: (0) not delimited by any margin; (1) delimited posteriorly by a longitudinal ridge; (2) delimited by anterior and posterior margins (Unordered; New)
5. Dorsal contact, dorsal extension: (0) well-beneath the quadrate head; (1) almost reaching or reaching the quadrate head (New)
6. Dorsal contact, ventral projection bounding the quadrate foramen: (0) absent; (1) present, ventrodorsally short projection; (2) present, ventrodorsally tall process (**Ordered**; New)

*Ventral quadratojugal contact*

1. Ventral quadratojugal contact, shape in posterior view: (0) concave; (1) straight; (2) convex (Unordered; New)
2. Ventral quadratojugal contact: (0) facing posterolaterally, contact overlapping the posteroventral part of the quadrate body; (1) facing laterally; (2) facing anterolaterally (Unordered; New)
3. Ventral quadratojugal contact, shape in lateral view: (0) ovoid to D-shaped; (1) drop-shape to d-shaped; (2) semi-circular; (3) subrectangular to subtrapezoidal; (4) elongated ellipse (Unordered; New)
4. Ventral quadratojugal contact, surface: (0) with radiating ridges; (1) roughly smooth; (2) irregular and weakly grooved; (3) heavily and deeply grooved (Unordered; New)
5. Ventral quadratojugal contact, surface: (0) not delimited by any upper margin; (1) delimited by upper margins (New)
6. Ventral quadratojugal contact, extension on lateral surface of ectocondyle: (0) limited, occupies only part of the surface; (1) extensive, covers entire lateral surface of the ectocondyle Brusatte et al. 2010 #108)
7. Ventral quadratojugal contact, anterior projection in ventral view: (0) absent; (1) present, short; (2) present, elongated (Unordered; New)
8. Ventral quadratojugal contact, ventrolateral projection in ventral view: (0) absent; (1) present (New)
9. Ventral quadratojugal contact, small perforation: (0) absent; (1) present (New)
10. Ventral quadratojugal contact, dorsal projection bounding the quadrate foramen: (0) absent; (1) present (New)

*Pterygoid contact*

1. Pterygoid contact, in posterior view: (0) contact on the pterygoid flange; (1) contact on the ventromedial or anteroventral side of the quadrate body (New)
2. Contact of the epipterygoid and the pterygoid flange, in medial view: (0) present; (1) absent, quadrate and epipterygoid remains separated (New)

*Braincase contact*

1. Braincase (opisthotic/exoccipital/paroccipital process) contact on the dorsal and/or medial part of the quadrate: (0) absent; (1) present (New)

VI. FORAMINA

*Quadrate foramen*

1. Quadrate foramen: (0) present; (1) absent (Modified from Novas, 1989 and Sereno et al. 1996 #36)
2. Quadrate foramen, position: (0) completely enclosed within the quadrate; (1) mostly delimited by the quadrate, only lateral margin of foramen bordered by quadratojugal; (2) developed as a distinct opening between the quadrate and quadratojugal. Lateral margin of the foramen formed by the quadratojugal and ventral and dorsal margins formed by both quadrate and quadratojugal; (3) developed as a distinct opening between the quadrate and postorbital. (**Ordered**; Modified from Novas, 1989)
3. Quadrate foramen, position in posterior view: (0) situated more ventrally than the mid-height of the quadrate, or covering most of the ventral part of the quadrate; (1) situated at mid-height of the quadrate (Modified from Holtz, 2000)
4. Quadrate foramen, position in lateral view: (0) facing posterolaterally and visible in lateral view; (1) facing posteriorly and not visible in lateral view (New)
5. Quadrate foramen, shape in posterior view: (0) subcircular; (1) strongly ventrodorsally elongated and elliptical or bean-shaped; (2) strongly ventrodorsally elongated and lenticular or tear drop shaped; (3) strongly lateromedially elongated (Unordered; New)
6. Quadrate foramen, size in posterior view: (0) minute, long axis less than 7% of the ventrodorsal height of the quadrate; (1) small, long axis between 7 to 15% of the ventrodorsal height of the quadrate; (2) large quadrate fenestra, long axis greater than 15% of the ventrodorsal height of the quadrate (**Ordered**; Holtz,1998 #67; Carr and Williamson 2010 #123)
7. Inclination of the main axis of the quadrate foramen: (0) absent, main axis parallel to quadrate ridge; (1) present, foramen strongly medially inclined; (2) present, foramen perpendicular to quadrate ridge (Unordered; New)

*Medial foramen*

1. Medial foramen, at the ventralmost part of the pterygoid flange: (0) absent; (1) present (Benson, 2009 #57)

VII. FLANGE & PROCESS

*Pterygoid flange*

1. Pterygoid flange, anterior extension in medial view (ratio between the anteroposterior length of the pterygoid flange/ventrodorsal height of the quadrate body): (0) >0.65; (1) 0.57-0.65; (2) 0.4-0.57; (3) <0.4 (**Ordered**; New)
2. Pterygoid flange, position of the anteriormost point: (0) at two-third of the quadrate height or more dorsally; (1) at mid-height of the quadrate; (2) at one-third of the quadrate height or more ventrally (Unordered; New)
3. Pterygoid flange, outline in medial view: (0) subtrapezoidal, formed by 3 distinct sides, the anteriormost one being ventrodorsally short; (1) subrectangular, formed by 3 distinct sides, the anteriormost one being ventrodorsally long; (2) roughly parabolic, formed by 3 poorly-defined sides, the anterior one being convex; (3) Semi-oval; (4) roughly M-shaped; (5) subtriangular, formed by two distinct sides (Unordered; Modified from Chiappe, 2001 #18)
4. Pterygoid flange, shape and orientation of the anteriormost side in medial view: (0) straight and inclined posteriorly from the long axis of the quadrate body; (1) straight and subparalell to long axis of quadrate body, or inclined anteriorly; (2) rounded or sigmoid (Unordered; New)
5. Pterygoid flange, angle between the main axis of the ventral margin and the main axis of the quadrate body in medial view: (0) < 55°; (1) 55° - 75°; (2) > 75° (**Ordered**; New)
6. Pterygoid flange, position of the ventralmost point in medial view (ratio between the distance separating the dorsal margin of entocondyle and ventral end of flange and the ventrodorsal height of the quadrate body): (0) well dorsal to the mandibular articulation (>0.1); (1) directly dorsal to the mandibular articulation (0.02-0.1); (2) reaching the mandibular articulation (<0.02). (**Ordered**; New)
7. Pterygoid flange, medial curvature in ventral view: (0) absent or weak, flange projecting mostly anteriorly; (1) present and important, flange curving anteromedially (New)
8. Pterygoid flange, curvature of the ventroposterior part at the level of the quadrate body in anterior view: (0) present, important; (1) present, short; (2) absent (Unordered; New)
9. Pterygoid flange, ventral shelf on the anteroventral margin in medial view: (0) absent; (1) present (New)
10. Pterygoid flange, posteromedial projection of the ventral part in posterior view: (0) absent; (1) present (New)

*Lateral process*

1. Lateral process: (0) present; (1) absent (Currie, 1995 and Sereno et al. 1996 #58)
2. Lateral process, ventral extension: (0) process extending to the quadrate foramen or at mid-height of the bone; (1) process extending below the mid-height of the bone, just above the ectocondyle or reaching it. (New)
3. Lateral process, maximum width: (0) large, >40% the lateromedial length of the mandibular articulation; (1) short, <40% the lateromedial length of the mandibular articulation (Modified from Forster 1999)
4. Lateral process, outline of lateral margin: (0) angular; (1) parabolic (New)
5. Lateral process, main orientation: (0) lateral; (1) anterolateral; (2) anterior (Unordered; New)
6. Lateral process, dorsal extension: (0) reaching the quadrate head; (1) not reaching the quadrate head (New)
7. Lateral process, extension of the dorsal contact: (0) contact extending entirely along the lateral process; (1) contact restricted to the ventral part of the lateral process; (2) contact restricted to the dorsal part of the lateral process (Unordered; New)

VIII. QUADRATE FOSSAE

*Medial fossae*

1. Medial fossa between pterygoid flange and quadrate body (pneumatic fossa excluded), in medial view: (0) shallow fossa; (1) deep depression (New)
2. Small fossa on the ventralmost part to the pterygoid flange, dorsal to the entocondyle, in medial view: (0) absent; (1) present (New)

*Posterior fossa*

1. Posterior fossa, in posterior view: (0) absent; (1) present and separated from the quadrate foramen; (2) present, and leading to or surrounding the quadrate foramen (Unordered; New)
2. Posterior fossa, shape in posterior view: (0) small oval and poorly delimited depression; (1) ventrodorsally tall, diagonally oriented, and poorly delimited depression; (2) ventrodorsally oriented, tall, and well-delimited depression (Unordered; New)

*Anterior fossa*

1. Anterior fossa, at one third of the quadrate height, lateral to the ventral extremity of the pterygoid flange in anterior view: (0) absent or shallow concavity; (1) present, deep depression (New)

IX. PNEUMACITY

1. Quadrate, pneumaticity: (0) absent; (1) present (Gauthier, 1986; Molnar, 1991)
2. Pneumatic foramen on the posterior surface of the quadrate body, within the posterior fossa, in posterior view: (0) absent; (1) present and ventral to the quadrate foramen; (2) present and at the same level or dorsal to the quadrate foramen; (3) present and at the same level than the quadrate foramen (Unordered; New)
3. Posterior pneumatic foramen, size (ratio between the maximum length of the pneumatic foramen and the lateromedial width of the mandibular articulation): (0) large, >30%; (1) small, <30% (New)
4. Pneumatic depression on the medial side, in medial view: (0) absent; (1) present, with no septum; (2) present and divided by a septum (Unordered; New)
5. Medial pneumatic foramen, size (ratio between the maximum length of the pneumatic foramen and the lateromedial width of the mandibular articulation): (0) small, <20%; (1) large, >20% (New)
6. Pneumatic foramen on the anteroventral margin of the quadrate body, in ventral view: (0) absent; (1) present, small circular pneumatic foramen; (2) present, large pneumatic recess (Unordered; New)
7. Pneumatic foramen on the anterodorsal surface of the quadrate body, ventral to the quadrate head, in anterior view: (0) absent; (1) present (New)
8. Pneumatic foramen on the lateral surface of the quadrate body, dorsal to the ectocondyle: (0) absent; (1) present (New)

## List of taxa included in the cladistic analysis

**Table A.** Quadrates of non-avian theropod taxa included in the cladistic analysis.

| **Taxon - author** | **Specimens** | **Examined** | **Photo credits** | **Source** |
| --- | --- | --- | --- | --- |
| *Eoraptor lunensis* Sereno et al., 1993 | PVSJ 512 | Y | Martín Ezcurra | Sereno et al., 2013 |
| *Herrerasaurus* *ischigualastensis* Reig, 1963 | PVSJ 53, 407 | Y | Martín Ezcurra |  |
| *Eodromaeus murphi* Martinez et al., 2011 | PVSJ 562 | Y |  |  |
| *Tawa hallae* Nesbitt et al., 2009 | GR 241 | N/photos | Sterling Nesbitt | Nesbitt et al., 2009 |
| ‘*Syntarsus*’ *kayentakatae* Rowe, 1989 | MNA V2623 | N/photos | Ronald Tykoski; Randall Irmis | Rowe, 1989 |
| *Liliensternus* *liliensterni* von Huene, 1934 | MB R.2175 | N/photos | Martín Ezcurra | von Huene, 1934 |
| *Dilophosaurus* *wetherilli* Welles, 1954 | UCMP 37302 | N/photos | Randall Irmis; Martín Ezcurra; Mathew Carrano | Welles, 1984 |
| *Ceratosaurus* *nasicornis* Marsh, 1884 | USNM 4735; MWC 1 | Y | Mathew Carrano |  |
| *Noasaurus leali* Bonaparte and Powell, 1980 | PVL 4061 | Y |  |  |
| *Masiakasaurus* *knopfleri* Sampson et al. 2001 | FMNH PR 2496 | Y | Mathew Carrano |  |
| *Abelisaurus comahuensis* Bonaparte and Novas, 1985 | MPCA 11098 | Y |  |  |
| *Ilokelesia* *aguadagrandensis* Coria and Salgado, 1998 | MCF PVPH 35 | Y | Matthew Lamanna |  |
| *Carnotaurus* *sastrei* Bonaparte, 1985 | MACN CH 894 | Y | Pablo Asaroff |  |
| *Aucasaurus garridoi* Coria et al., 2002 | MCF-PVPH-236 | Y |  |  |
| *Majungasaurus* *crenatissimus* Lavocat, 1955 | FMNH PR 2100 | Y | Lawrence Witmer |  |
| *Cryolophosaurus* *ellioti* Hammer and Hickerson, 1994 | FMNH PR1821 | N/photos | Nathan Smith | Smith et al., 2007 |
| *Monolophosaurus* *jiangi* Zhao and Currie, 1993 | IVPP 84019 | N/publi |  | Zhao and Currie 1993; Brusatte, et al. 2010a |
| *Eustreptospondylus* *oxoniensis* Walker, 1964 | OUMNH J.13558 | N/photos | Paul Barrett |  |
| *Afrovenator* *abakensis* Sereno et al., 1994 | UC OBA1 | N/photos | Roger Benson; Juan Canale; Mathew Carrano | |
| *Torvosaurus tanneri* Galton and Jensen, 1979 | BYUVP 9246 | Y | Matthew Lamanna |  |
| *Baryonyx walkeri* Charig and Milner, 1986 | NHM R9951 | Y | Eric Buffetaut |  |
| *Suchomimus* *tenerensis* Sereno et al., 1998 | MNN GAD 502 | Y | Steve Brusatte |  |
| *Irritator* *challengeri* Martill et al., 1996 | SMNS 58022 | Y |  |  |
| *Spinosaurus aegyptiacus* Stromer, 1915 | FSAC-KK 11888 | N/photos | Nizar Ibrahim |  |
| Spinosaurinae morphotype I (*Spinosaurus aegyptiacus*) | MHNM.KK374 to.KK375 and .KK377 to.KK378; MSNM V6896 | Y |  |  |
| Spinosaurinae morphotype II (?*Sigilmassasaurus brevicolis*) | MHNM.KK376 | Y |  |  |
| *Allosaurus* *fragilis* Marsh, 1877 | BYU VP8901 | Y |  | Osborn, 1912; Gilmore, 1920; Madsen, 1976b; Chure, 2000 |
| *Allosaurus* *europaeus* Mateus et al., 2006 | ML 415 | Y |  |  |
| *Allosaurus* ‘*jimmadseni*’ Chure, 2000 | SMA 005/02 | Y |  |  |
| *Aerosteon* *riocoloradensis* Sereno et al., 2008 | MCNA-PV-3137 | N/photos | Martin Ezcurra |  |
| *Sinraptor dongi* Currie and Zhao, 1993a | IVPP 10600 | N/photos | Philip Currie | Currie, 2006 |
| *Acrocanthosaurus* *atokensis* Stovall and Langston, 1950 | NCSM 14345 | Y | Drew Eddy; Vince Shneider |  |
| *Shaochilong* *maortuensis* Brusatte, Benson, et al., 2009 | IVPP V2885.3 | N/photos | Steve Brusatte | Brusatte, Benson, et al., 2009; Brusatte, Chure, et al., 2010 |
| *Mapusaurus roseae* Coria and Currie, 2006 | MCF PVPH 108.102 | Y | Matthew Lamanna |  |
| *Giganotosaurus* *carolinii* Coria and Salgado, 1995 | MUCPv-CH-1 | Y | Matthew Lamanna |  |
| *Ornitholestes* *hermanni* Osborn, 1903 | AMNH FARB 619 | Y | Mickey Mortimer |  |
| *Bicentenaria* *argentina* Novas et al., 2012 | MPCA 865 | Y |  |  |
| *Zuolong salleei* Choiniere, Clark, et al., 2010 | IVPP V15912 | N/publi |  | Choiniere, Clark, et al., 2010 |
| *Proceratosaurus* *bradleyi* von Huene, 1926 | NHM R 4860 | Y |  | Rauhut et al., 2010 |
| *Guanlong wucaii* Xu et al., 2006 | IVPP V14531 | N | Oliver Rauhut |  |
| *Eotyrannus lengi* Hutt et al., 2001 | MIWG 1997.550 | Y | Darren Naish |  |
| *Xionggualong* *baimoensis* Li et al., 2010 | FRDC-GS JB16-2-1 | N/publi |  | Li et al., 2010 |
| *Alioramus altai* Brusatte, Carr, et al., 2009 | IGM 100-1844 | N/photos | Mick Ellison | Brusatte et al., 2012 |
| *Albertosaurus* *sarcophagus* Osborn, 1905 | RTMP 81.10.1; FMNH PR308; CMN 12; CMN 2120 | N/publi |  | Carr, 1996; Currie, 2003 |
| *Daspletosaurus* sp. Russell, 1970 | RTMP 94.143.1 | N/publi |  | Currie, 2003 |
| *Tyrannosaurus* *rex* Osborn, 1905 | AMNH 5027; FMNH PR2081 | Y | Mickey Mortimer | Molnar, 1991; Brochu, 2003; Larson, 2008 |
| *Sinosauropteryx prima* Ji and Ji, 1996 | NIGP 127587 | N/publi |  | Currie and Chen, 2001 |
| *Garudimimus* *brevipes* Barsbold, 1981 | GIN 100/13 | N/photos | Yoshitsugu Kobayashi | Kobayashi and Barsbold, 2005 |
| *Sinornithomimus* *dongi* Kobayashi and Lü, 2003 | IVPP−V11797−10 | N/photos | Yoshitsugu Kobayashi | Kobayashi and Lü, 2003 |
| *Ornithomimus* *edmontonicus* Sternberg, 1933 | RTMP 95.110.1 | N/photos | Rui Tahara | Tahara and Larsson, 2011 |
| *Shuvuuia deserti* Chiappe et al., 1998 | IGM 100-977, 100-1001 | Y |  |  |
| *Falcarius utahensis* Kirkland et al., 2005 | UMNH VP 14559 | N/photos | Lindsay Zanno | Zanno, 2010 |
| *Erlikosaurus andrewsi* Barsbold and Perle, 1980 | PST 100/111 | N/photos | Stephan Lautenschlager | Clark et al., 1994 |
| *Avimimus* *portentosus* Kurzanov, 1981 | PIN 3907/1 | N/photos | Lawrence Witmer | Kurzanov, 1985; Vickers-Rich et al., 2002 |
| Oviraptoridae Barsbold, 1986 | AMNH FARB 6517; IGM 100-1127; IGM 100-978 | Y |  | Maryańska and Osmólska, 1997; Balanoff and Norell, 2012 |
| *Buitreraptor* *gonzalezorum* Makovicky et al., 2005 | MPCA 245 | Y | Martín Ezcurra |  |
| *Bambiraptor* *feinbergi* Burnham et al., 2000 | FIN 001 | Y | David Burnham |  |
| *Tsaagan mangas* Norell et al., 2006 | IGM 100-1015 | Y | Mick Ellison |  |
| *Dromaeosaurus* *albertensis* Matthew and Brown, 1922 | AMNH 5356 | Y |  |  |
| Troodontidae Gilmore, 1924 | IGM 100-1083, 100-1128; 100-1323 | Y | Rui Pei | Norell and Hwang, 2004 |

## Supermatrix on the non-avian theropod quadrate (Excel file)

The file is downloadable at

<https://drive.google.com/file/d/0B_-0b-kZatHiQ3NIOUdDNWdqVkE/view?usp=sharing>

and can be obtained by request to the corresponding author.

## Cladistic analysis on the non-avian theropod quadrate (TNT file)

The cladistic analysis was performed on a supermatrix combining the quadrate based datamatrix of Hendrickx et al. [71] with six recent datasets ([14,73–77]) on the whole theropod skeleton and from which all quadrate related characters were removed.

The file is downloadable at

<https://drive.google.com/file/d/0B_-0b-kZatHidXV5UEN6YWFfRkE/view?usp=sharing>

and can be obtained by request to the corresponding author.

nstates 6

xread

2377 60

*Eoraptor* ?02100?000?1?0?00??0??????????????0???00?????00?1???0???0???0101000???????????000100???0-?0-------??0000000000?000----0?00?01001000000100????0000000001?01-000000000-0--11100--001001?0?-000?0-00??0??0000000???0???0000???????????????????????????????????????????0101?000-00?0??00-0-0?010?0??0?0???????101000000010000000000???0011?0-0?????0?0?????????0?10?????0?00??0?00????????0??0????01???????0????0???0?00???0??0??????00?????????0?????00??00?0???0?10?0??????????0??0????????10?01???0??0110???0100???0????00000-0??0???????000?100?????????0-?????00000?010?????00-1???0-????0????2??0?0???0??????????0--???????00???????????????????0-?????0??????????0110001010000000001101?10000?0100000-10000[02][01]10??0001001001000?00000?21000101102010000012010100100000000000-000100000000001001?0?00000010000????????????????????????????????????????????????????????????????????????????????????????????????????????????????????????????????????????????????????????????????????????????????????????????????????????????00010000?00000??????0101000?0?0010000?0000100??0001?0000001000000?00000000000?001000?0000?0??????0??00?0?101000000?0?0000?00?0000?0?00?000100000000000-0?????????-00000000000000?0?00000000000000?0000001?0010000?000001000000101000000000?00100000100000000000010000000?00000000000??000?0000000?00000?0000?000?000000000???-0000000000000000000000000???????????????????????????????????????????????????????????????????????????????????????????????????????????????????????????????????????????????????????????????????????????????????????????????????????????????????????????????????????????????????????????????????????????????????????????????????????????????????????????????????????????????????????????????????????????????????????????????????????????????????????????????????????????????????????????????????????????????????????????????????????????????????????????????????????????????????????????????????????????????????????????????????????????????????????????????????????????????????????????????????????????????????????????????????????????????????????????????????????????????????????????????????????????????????????????????????????????????????????????????????????????????????????????????????????????????????????????????????????????????????????????????????????????????????????????????????????????????????????????????????????????????????????????????????????????????????????????????????????????????????????

*Herrerasaurus* 000110000010000001000010100[01]2011010?0000?????0001???[01]1??00010101000011001001000000100000-00-------??000000?00??100----???0010-00000000000????0?00000001?201000000000-0--01100--0010000??-?00?0-00?00??00?1?????000?0?0000?0????0??000??1?1?0?0?0-000????0??????????0??1?000-0?0011???????010?000??????????1010?000?010000000???0?000??00-0000100?0????0010?001000?0000000000000000100?0??1??0?00000???200000000?000?0000?????????00?????????0??0-00?1010?00110?1000010??0100?0?00001?0??010000?10000011000?11100000???0000?0-0??0????00-00001001????00??1010???00000?0101????00-1?1?0-??010000?2000000000000000?0000----0?0??100????001000000??00?0-0?00?000?000?01000100?10000000000101010010000?110000-100000001100001001001000010?0011200111011000010000100000000000001001100101001000000000000000000001000100000000000210000000000000000000100100[01]0000000000000000?00000000000000000010000000000000000010000000000000000[01]00100000010000000?000000000001100?0000000000100000000000000000000000000000000?000000000000000000001000000000????0?0001000?0?0?00000?0000100??001201000000000000?0000010000000010?00000?10??00000000?000000?10000?0?0000?000010010000?0?0100000000000-00?000010?-00000000000000?0?1000000?0000000000000??001??00?1?011?0??000101000000100000101000100000000000010000000?00000000000?0?001000000?00000??0000100000000000001??-0100001000000000000000000???????????????????????????????????????????????????????????????????????????????????????????????????????????????????????????????????????????????????????????????????????????????????????????????????????????????????????????????????????????????????????????????????????????????????????????????????????????????????????????????????????????????????????????????????????????????????????????????????????????????????????????????????????????????????????????????????????????????????????????????????????????????????????????????????????????????????????????????????????????????????????????????????????????????????????????????????????????????????????????????????????????????????????????????????????????????????????????????????????????????????????????????????????????????????????????????????????????????????????????????????????????????????????????????????????????????????????????????????????????????????????????????????????????????????????????????????????????????????????????????????????????????????????????????????????????????????????????????????????????????????????

*Eodromaeus* 0???0?00?0100000010000201002[12]0210[01]?1000????????000200000???????????0????100??0???????????00-------??????????????????????????????????????????????????????????????????????????????????????????????????????????????????????????????????????????????????????????????????????????????????????????????????????????????????????????????????????????????????????????????????????????????????????????????????????????????????????????????????????????????????????????????????????????????????????????????????????????????????????????????????????????????????????????????????????????????????????????????????????????????????????????????????????????????????????????????????00?00?1000?0?00001?????0???0?1???000-?0000000010101100100?000010?001120011101130001000120001000000100000110000100110000000000000000000?00????????????????????????????????????????????????????????????????????????????????????????????????????????????????????????????????????????????????????????????????????????????????????????????????????????????????????????????????????????????????????????????????????????????????????????????????????????????????????????????????????????????????????????????????????????????????????????????????????????????????????????????????????????????????????????????????????????????????????????????????????????????????????????????????????????????????????????????????????????????????????????????????????????????????????????????????????????????????????????????????????????????????????????????????????????????????????????????????????????????????????????????????????????????????????????????????????????????????????????????????????????????????????????????????????????????????????????????????????????????????????????????????????????????????????????????????????????????????????????????????????????????????????????????????????????????????????????????????????????????????????????????????????????????????????????????????????????????????????????????????????????????????????????????????????????????????????????????????????????????????????????????????????????????????????????????????????????????????????????????????????????????????????????????????????????????????????????????????????????????????????????????????????????????????????????????????????????????????????????????????????????????????????????????????????????????????????????????????????????????????????????????????????????????????????????????????????????????????????????????

*Tawa* 0??10?00???100000100002?3001??1?00000?0000?????010000100?0?????????02100100100???????00???0-------??0001201000??00----01001?11000?00000?1????0?000?0011?200?00100010-0--12100--?1100?????000?0-00?0?0-??11000??????????0?020?001??000????000??1??0??????1???????????10??000-00000-00-100?00-?0????0????????00??00002000?0000?0000?000100-0???????1100?00??00?1?????0?0????????????1????1???0???????0???????0??00????0?0?00????????????????????????1?10100?0100110000????????????0??1????000??10??00?01110001110?000000000000-0???0--?0?00?00000100?000?0110????????10?????????????????????001??2000000??100????????????????????????000100000000?010-?00?00??00?1????0000010111?11010110001?010???0??0000-?0010000???101??01????0?01???011201111011?0?0100010???1?0000010000011110010010000?0000001?11???00?0000??????????????????????????????????????????????????????????????????????????????????????????????????????????????????????????????????????????????????????????????????????????????????????????????????????????????????????????????????????????????????????????????????????????????????????????????????????????????????????????????????????????????????????????????????????????????????????????????????????????????????????????????????????????????????????????????????????????????????????????????????????????????????????????????????????????????????????????????????????????????????????????????????????????????????????????????????????????????????????????????????????????????????????????????????????????????????????????????????????????????????????????????????????????????????????????????????????????????????????????????????????????????????????????????????????????????????????????????????????????????????????????????????????????????????????????????????????????????????????????????????????????????????????????????????????????????????????????????????????????????????????????????????????????????????????????????????????????????????????????????????????????????????????????????????????????????????????????????????????????????????????????????????????????????????????????????????????????????????????????????????????????????????????????????????????????????????????????????????????????????????????????????????????????????????????????????????????????????????????????????????????????????????????????????????????????????????????????????????????????????????????????????????????????????????????????????????????????????????????????

*Liliensternus* ????3000?01011000??1????????001??????????????????????????????????????????0?1??0???1???01?00-------???????????????????????????????????????????????????????????????????????????????????????????????????????????????????????????????????????????????????????????????????????????????????????????????????????????????????????????????????????????????????????????????????????????????????????????????????????????????????????????????????????????????????????????????????????????????????????????????????????????????????????????????????????????????????????????????????????????????????????????????????????????????????????????????????????????????????????????????????????????????????????????????????????????????????????????????????????????????????????????????????????????????????????????????????????????????????????????????????????????????????????????????????????????????????????????????????????????????????????????????????????????????????????????????????????????????????????????????????????????????????????????????????????????????????????????????????????????????????????????????????????????????????????????????????????????????????????????????????????????????????????????????????????????????????????????????????????????????????????????????????????????????????????????????????????????????????????????????????????????????????????????????????????????????????????????????????????????????????????????????????????????????????????????????????????????????????????????????????????????????????????????????????????????????????????????????????????????????????????????????????????????????????????????????????????????????????????????????????????????????????????????????????????????????????????????????????????????????????????????????????????????????????????????????????????????????????????????????????????????????????????????????????????????????????????????????????????????????????????????????????????????????????????????????????????????????????????????????????????????????????????????????????????????????????????????????????????????????????????????????????????????????????????????????????????????????????????????????????????????????????????????????????????????????????????????????????????????????????????????????????????????????????????????????????????????????????????????????????????????????????????????????????????????????????????????????????????????????????????????????????????????????????????????????????????

*Syntarsus* 01010000000100000?010??0???1??1???100?10??1??0001???0???00??0111000??????????00000100??10?0-------???0?0211?0??000-??0???0000?0101001?1110??1010?001001?11-000000000-0--122?0--0012010??-?0000-00?00??0110?????00??1?0000?0???01??100?00?0?0?0?10000??0010-???0???????1?100-??0011?100-?10101000??????????1010?010?010??00000??1??00??00-0110??0?1100?00???001000?0000010002??100010?00??0?00?03010???1?0000020?000?00001?1?0-1??00?????????0??1000?1000?0?1?0?10001100??1???0??0?0??1??000001?001000110?0?11110000???011000-0000????10000001101???0001?0-00???0001101100010110010000-0?110000??00000[01]010000101?10110?000?10?1010??1001000000?1?0?100?0??00????1001010010?0111111010110111?010????100000-?0010000???10?11????111??????011?????????3100111113000??11??????????1110011011111110?0??-?1111?11?????000001000010000000000000000000001000000000000000000000000000000010000000010000000000000000000010000001000000010121101000000010000010000[01]00000011000000000000000?101101101000000100010011000000100010001?0000??????????????????????????????????????????????????????????????????????????????????????????????????????????????????????????????????????????????????????????????????????????????????????????????????????????????????????????????????????????????????????????????????????????????????????????????????????????????????????????????????????????????????????????????????????????????????????????????????????????????????????????????????????????????????????????????????????????????????????????????????????????????????????????????????????????????????????????????????????????????????????????????????????????????????????????????????????????????????????????????????????????????????????????????????????????????????????????????????????????????????????????????????????????????????????????????????????????????????????????????????????????????????????????????????????????????????????????????????????????????????????????????????????????????????????????????????????????????????????????????????????????????????????????????????????????????????????????????????????????????????????????????????????????????????????????????????????????????????????????????????????????????????????????????????????????????????????????????????????????????????????????????????????????????????????????????????????????????????????????????????????????????????????????????????????????????????????????????????????????????????????????????????????????????

*Dilophosaurus* 1101200000100000020000203101[12]01111?0000?10110?111120010000?1010130202000201100000010?002200-------??001020?00??0?0----1?????0-010?0010011[01]0[01]1??0?0?1001?201000100010?0--1111???0010?0????00010-01?0????111???????????0000?0???01??000?11?0?0?0?10000??101?????0?????1?1?1?0-???0???0-10?00101?01??????????1010?000?1000000000??1?10???00-0110110?1101?0011?0011?0??0?011000200?10?10000??1100?03000???0?0000020?000?01000?0???10?0??????????0??100010?00?0?100?10000100?0100?0??010?01?00?0001?00100011000?1?11000?????11000-???0????1?000001101????100?0-00???100?00?100????10-????0-??11000001000000000?0000??00110?000?0??100????001000000???0?10??0??000?0?20010?0000101111110101101110010?111100000-?0010000?1111011010011?10100001121111101131001110130001011000001000?1110011011110110001011111101111100????????????????????????????????????????????????????????????????????????????????????????????????????????????????????????????????????????????????????????????????????????????????????????????????????????????01?1121100001000000[01]001000100000000?100200100??00?200100001?0001001000?11011000?000000200101000001??0??0?????201100010000?1000000[01]1000000010000010101101??00011100100000101100000000000010101000010000201??10000100000010010011010000001010001?111011000100010010001100001100110[01]000?10000010001?00100110[01]000000000001000211002100001010110100011000?0???????????????????????????????????????????????????????????????????????????????????????????????????????????????????????????????????????????????????????????????????????????????????????????????????????????????????????????????????????????????????????????????????????????????????????????????????????????????000000000000100000?00000000??000000?011002?001001000000000?00?0???????00000000000000100000000????????00000000000001000000000000000000000000000000100100001300000000001100100100000000000000?000?0000000?0??????20000000000000000000100000100?1010000000000000001000000001000000001000000000000000000000000000000000020000000000000000000000001000000000010000100000000000000000000000000000000?00000?110000010010000001000000000000001?001001??????01000000??????000100000?0000000001010100100001310001000010?000100000000?????????00?0000000000?0?0001000000001000011000000001000001000100010010?0000000000000000000000000000000000001000100???0?0000??0000010000000110??02?1010011000001000?000??0000010000000000001001000000000?00?00000000000000000?0100000??00000000

*Cryolophosaurus* 10010000-0100?001?0?0???????????????0??0??1??0011???1???001?0111100?00?02000?01------10???0-------????????????????????????????????????????????????????????????????????????????????????????????????????????????????????????????????????????????????????????????????????????????????????????????????????????????????????????????????????????????????????????????????????????????????????????????????????????????????????????????????????????????????????????????????????????????????????????????????????????????????????????????????????????????????????????????????????????????????????????????????????????????????????????????????????????????????????????????????????????????????????????????????????????????????????????????????????????????????????????????????????????????????????????????????????????????????????????????????????????????????????????????????????????????????????????????????????????????????????????????????????????????????????????????????????????????????????????????????????????????????????????????????????????????00?????????1??00?1001?01010?000?0111??0?00[01]0?1?10000?10??10001000001??1?0010????0??????1??0????????????10?000000000?0?01??????0?01000????????00?0?000?0?1000??001000??010?0?????000200????0?01??????????????????????0?10001??????????????00???00???0?????011??0????0???0?0??10011001?0?00110100000000?2??????01001011??0???????????????????????????????????????????????????????????????????????????????????????????????????????????????????????????????????????????????????????????????????????????????????????????????????????????????????????????????????????????????????????????????????????????????????????????????????????????????????????????????????????????????????????????????????????????????????????????????????????????????????????????????????????????????????????????????????????????????????????????????????????????????????????????????????????????????????????????????????????????????????????????????????????????????????????????????????????????????????????????????????????????????????????????????????????????????????????????????????????????????????????????????????????????????????????????????????????????????????????????????????????????????????????????????????????????????????????????????????????????????????????????????????????????????????????????????????????????????????????????????????????????????????????????????????????????????????????????????????????????????????????????????????

*Ceratosaurus* 0101000000110000000101102001001011010100101000-11[01]101000-0111------020010001100000010100-00-------??10100010001100---00?00?0100000001000010001111010101101-100000001110?11100--001001000000010-011101010010000?00?0??1000?02??01??000?0??1?1???10?00???0???????????1????000-???0???0-10?001000?0??????100?100??000?11000?0000??0?0?0??00-0000111?11011000000011??0?00101?00100????10000011011004001?0?1?000?01000?0?01000010??10000?????????00010?01001000???????????????10001100101????0100?100010101110??1??????2????1100??0000????1?000001001000000000-000101000100100000010-00000-0?11000000000011011000111011110?0?0000?1000???001000011????????????000?????????01001000010000011111100101111100000-11000000111110100100111?0100?0112111110?131001000120001000000001000?10002100111101100000-?1111???111??000012001000000001000000000101002010000000000000000000000011?110000111011100111101011000011111211111121000000[01]1200001101110?1?00001002000001000001111[01]000010100?1111??01000?111111010[01]1101101101111100010???0210100010000000000000000010100010120?000000202001100000001000011111100010101010100000000110100001110000?100010000001??1000000100100010000010100100011021?0100010000000021100000010100001010211??10000210?0100001101011110100100110000????0??1??1?01?00010?010?11100?0000111011110110100001[01]0001001100110[01]001102100010100200211101001021??1?0001??10??????????????????????????????????????????????????????????????????????????????????????????????????????????????????????????????????????????????????????????????????????????????????????????????????????????????????????????????????????????????????????????????????????????????????????????????????????????????????????????????????????????????????????????????????????????????????????????????????????????????????????????????????????????????????????????????????????????????????????????????????????????????????????????????????????????????????????????????????????????????????????????????????????????????????????????????????????????????????????????????????????????????????????????????????????????????????????????????????????????????????????????????????????????????????????????????????????????????????????????????????????????????????????????????????????????????????????????????????????????????????????????????????????????????????????????????????????????????????????????????????????????????????????????????????????????????????????????????????????????????????????????

*Noasaurus* ????00?0?11100000?0001000000?1??0???010??????????????????0?????????0210100010001??01?00??00-------?????????????????????????????????????????????????????????????????????????????????????????????????????????????????????????????????????????????????????????????????????????????????????????????????????????????????????????????????????????????????????????????????????????????????????????????????????????????????????????????????????????????????????????????????????????????????????????????????????????????????????????????????????????????????????????????????????????????????????????????????????????????????????????????????????????????????????????????????????????????????????????????????????????????????????????????????????????????????????????????????????????????????????????????????????????????0??1?00111110?0??????????????????????????????????????????????????????????????????????10011??01[12]????????1?1?10111??00???????????????????000????????????????????00??????????????????0???????????????????1???????????????????????????????????????????????????????????????????????????????????????????????????????????????????????????????????????????????????????????????????????????????????????????????????????????????????????????????????????????????????????????????????????????????????????????????????????????????????????????????????????????????????????????????????????????????????????????????????????????????????????????????????????????????????????????????????????????????????????????????????????????????????????????????????????????????????????????????????????????????????????????????????????????????????????????????????????????????????????????????????????????????????????????????????????????????????????????????????????????????????????????????????????????????????????????????????????????????????????????????????????????????????????????????????????????????????????????????????????????????????????????????????????????????????????????????????????????????????????????????????????????????????????????????????????????????????????????????????????????????????????????????????????????????????????????????????????????????????????????????????????????????????????????????????????????????????????????????????????????????????????????????????????????????????????????????????????????????????????????????????????????????????????????????????????????????????????????????????????????????????????????????????????????????????

*Masiakasaurus* 01??1000011100000000010000?0011001?10?00??????-111101000-0??1------0????2001?0010?0???0??00-------???????????????0--??100???0-00???0100??10000???????????????????????????????????0-???????????????????????????????????????????????????????????????????????????????????1?110-01?11100-1000?????????????0-??????????0?00?0?00?000?00010100-0???????1101-10??00?0-1?2110?0???01??00001??00010?01?0???01?0?110?0020????0??0?0???????????????????00010???????????100?1001????????????????????????????????????????????????????????????????????????????????????110?01000000????????????????????0100100010000100100011101011000000?????????1??100111100?????000?0??00?????00???????????????????????????????????????????????????????????????????????????????????????????????????????????????????????????????????????????00?1?00?111?0?????00000000????00001000100?0?0010????????????11?1001???????10?1110000110011?0?12?01111211111001112100111010111100111000?0111?01011111010?????110?1111?11010??1110110111210?1111111111111111010?????0???00100100000100001000???0?????????10???????????0??20101?10?????????0????000?0001??00???0100?????????11101000011-0??00?000100000000???0020001102?00110010011000111?000000101000000002011110001011?0100001?01011111001110100001????????????????????001001?1000000011?111111120100001001001??10011010011021000101002102?1111112021111?0011??1101????????????????????????????????????????????????????????????????????????????????????????????????????????????????????????????????????????????????????????????????????????????????????????????????????????????????????????????????????????????????????????????????????????????????????????????????????????????????????????????????????????????????????????????????????????????????????????????????????????????????????????????????????????????????????????????????????????????????????????????????????????????????????????????????????????????????????????????????????????????????????????????????????????????????????????????????????????????????????????????????????????????????????????????????????????????????????????????????????????????????????????????????????????????????????????????????????????????????????????????????????????????????????????????????????????????????????????????????????????????????????????????????????????????????????????????????????????????????????????????????????????????????????????????????????????????????????????????????????????????????????????

*Abelisaurus* ?00100???11102000?010?00?0??0??0??0[01]0?0?????????1????????010???????000010101000???01??0??10-------??10??001?0010?????0??0???10000?00000001?????10?101???????00?????11????1?1????1??11?000011?1101110101100000????????1000???????????????????????????????????????????????????????????????????????????????????1???????[01]?0??0?????????????0-0??????????????????????????????????????????????????????????????????????????????????????????????????????????????????????????????????????????????????0??????????????????????????????????????????????????????????????????????????????????????????????????????????????????????????????????????????????????????????????????????????????????????????????????????????????????????????????????????????????????????????????????????????????????????????????????????????????????1111120?1?021??1011111121011111011101000101?11111?1001?11110???10011??1???1??1?????????101????????????????????????????????????????????????????????????????????????????????????????????????????????????????????????????????????????????????????????????????????????????????????????????????????????????????????????????????????????????????????????????????????????????????????????????????????????????????????????????????????????????????????????????????????????????????????????????????????????????????????????????????????????????????????????????????????????????????????????????????????????????????????????????????????????????????????????????????????????????????????????????????????????????????????????????????????????????????????????????????????????????????????????????????????????????????????????????????????????????????????????????????????????????????????????????????????????????????????????????????????????????????????????????????????????????????????????????????????????????????????????????????????????????????????????????????????????????????????????????????????????????????????????????????????????????????????????????????????????????????????????????????????????????????????????????????????????????????????????????????????????????????????????????????????????????????????????????????????????????????????????????????????????????????????????????????????????????????????????????????????????????????????????????????????????????????????????????????????????????????????????????????????????????????????????????????????????????????????????????????????????????????????????????????????????????????????????????????

*Ilokelesia* ????1?0011110000000101000001001101?[01]0000??1??0-122101000?0?????????0?????1????010?01??0??00-------??????????????????????????????????????????????????????????????????????????????????????????????????????????????????????????????????????????????????????????????????????????????????????????????????????????????????????????????????????????????????????????????????????????????????????????????????????????????????????????????????????????????????????????????????????????????????????????????????????????????????????????????????????????????????????????????????????????????????????????????????????????????????????????????????????????????????????????????????????????????????????????????????????????????????????????????????????????????????????????????????????????????????????????????????????????????1????????????????????????????11101111010?1??????????????????1???????????????????????????????1???????221001010120110?111????????1??112?011??????????????????????????????????????????????????????????????111???????????????????????????????????????????????????????????????????????????????????????????????????????????????????????????????????????????????????????????????????????????????????????????????????????????????????????????????????????????????????????????????????????????????????????????????????????????????????????????????????????????????????????????????????????????????????????????????????????????????????????????????????????????????????????????????????????????????????????????????????????????????????????????????????????????????????????????????????????????????????????????????????????????????????????????????????????????????????????????????????????????????????????????????????????????????????????????????????????????????????????????????????????????????????????????????????????????????????????????????????????????????????????????????????????????????????????????????????????????????????????????????????????????????????????????????????????????????????????????????????????????????????????????????????????????????????????????????????????????????????????????????????????????????????????????????????????????????????????????????????????????????????????????????????????????????????????????????????????????????????????????????????????????????????????????????????????????????????????????????????????????????????????????????????????????????????????????????????????????????????????????????????????????????

*Carnotaurus* 01020000011100000000010100010110010?0?00??0??1-?????100?-0?01------?20011010000100110100-10-------??10000000001000---0??00?010000100010001???1?1001110??200000000?00-10101000--?10-110000001?1101100101000000???0????1000??2??0????0????00?1001??0?0???????????????1101100??00?01000-100?01000??0?11101000101000??01000000?0?0000000???0-000010101101?10?00??11102?10100?00100?10?10000111?110040?11?0??????00????0?????0000???0??????00--0?0000-?01001010?221?0110?200000-000111000----???????????????????????????????11000-0000??????0000??00??0?00000100????1000000100?000?0-00000-??000?10000?0111011??011?00?110?0????????????????????????????????????????????????????????????????????????????????????????????????????????????????????????????????????????????????????????????????????????????????????????111112111?02111011011112101111110111001110111011111111111011???100111?111?111111111110?10100012?11111211101110120100111111111111111?1?1011110110111111111211111?1111111111111?11110?1111????11????1???????????????????????????????????????????????????????????????????????????????????????????????????????????????????????????????????????????????????????????????????????????????????????????????????????????????????????????????????????????????????????????????????????????????????????????????????????????????????????????????????????????????????????????????????????????????????????????????????????????????????????????????????????????????????????????????????????????????????????????????????????????????????????????????????????????????????????????????????????????????????????????????????????????????????????????????????????????????????????????????????????????????????????????????????????????????????????????????????????????????????????????????????????????????????????????????????????????????????????????????????????????????????????????????????????????????????????????????????????????????????????????????????????????????????????????????????????????????????????????????????????????????????????????????????????????????????????????????????????????????????????????????????????????????????????????????????????????????????????????????????????????????????????????????????????????????????????????????????????????????????????????????????????????????????????????????????????????????????????????????????????????????????????????????????????????????????????????????????????????????????????????????????????????????????????

*Aucasaurus* ????0000001100000002010000?2012101?0010????????111200000???????????0?????0????01??01???1000-------?????????????????????????????????????????????????????????????????????????????????????????????????????????????????????????????????????????????????????????????????????????????????????????????????????????????????????????????????????????????????????????????????????????????????????????????????????????????????????????????????????????????????????????????????????????????????????????????????????????????????????????????????????????????????????????????????????????????????????????????????????????????????????????????????????????????????????????????????????????????????????????????????????????????????????????????????????????????????????????????????????????????????????????????????????????????11?112??1?0?11???1??11?2??????0????????????110???1???????????????????????????????????0?????????????????1?????????1??????[01]?????1?11111?1?????010?1111111112111?11??11?11???111???1?0??1?1111??1?1111??0??1111??????????????????????????????????????????????????????????????????????????????????????????????????????????????????????????????????????????????????????????????????????????????????????????????????????????????????????????????????????????????????????????????????????????????????????????????????????????????????????????????????????????????????????????????????????????????????????????????????????????????????????????????????????????????????????????????????????????????????????????????????????????????????????????????????????????????????????????????????????????????????????????????????????????????????????????????????????????????????????????????????????????????????????????????????????????????????????????????????????????????????????????????????????????????????????????????????????????????????????????????????????????????????????????????????????????????????????????????????????????????????????????????????????????????????????????????????????????????????????????????????????????????????????????????????????????????????????????????????????????????????????????????????????????????????????????????????????????????????????????????????????????????????????????????????????????????????????????????????????????????????????????????????????????????????????????????????????????????????????????????????????????????????????????????????????????????????????????????????????????????????????????????????????????????

*Majungasaurus* 0001000001110000000201010002011001000000001000-111201000-0101------020012000100100010101010-------??10100010001000--??100000100001000100011100?101101011201000000010-101?1010--010-110000011?11011111011000000000001?1000012??01000001?1?????0???0?0?????0-????????010110???0?00?????10?0?1000100001001000101000000110000000000000000100-0000111010-1?10000?011?020001010001001101?00000010110030??1?001100002000000110000000?100100?????????000-00??0?0?002210010012000??????????0???????????????????11???????????????010010000010001000000100100000010????????????0???0??????-??0???????????000000??00100011101101010000111100010000100010??0?0???00000000??0?0000???????????????????????????????????????????????????????????????????????????????????????????????????????????????????????????????????????????111112011102110101111111101111110110001000111101111011111111111101111111121111111001101101000121111112111010101201001111[01]11111101100021011??010?111111??????1?????1111111011?11??11?11??0?1011?11111100111110210100010000001000000010010000011120?10020000?101200000020202012?111101101110100?00000010?01101?100000001010110010100110010001001010000000100001000110210011001001100012110000001010000001021?1111001201?01000011?1011?10?011102000010??????0????????????0??0011100000001111111??????0???1?????????0????100110?100010100210211111111021111?0001??1101????????????????????????????????????????????????????????????????????????????????????????????????????????????????????????????????????????????????????????????????????????????????????????????????????????????????????????????????????????????????????????????????????????????????????????????????????????????????????????????????????????????????????????????????????????????????????????????????????????????????????????????????????????????????????????????????????????????????????????????????????????????????????????????????????????????????????????????????????????????????????????????????????????????????????????????????????????????????????????????????????????????????????????????????????????????????????????????????????????????????????????????????????????????????????????????????????????????????????????????????????????????????????????????????????????????????????????????????????????????????????????????????????????????????????????????????????????????????????????????????????????????????????????????????????????????????????????????????????????????????????????

*Monolophosaurus* 10010?0000102?000?0?0?????????????0?0??0??1???001???????00?00101220?200110??100011011100-00-------??001000100101?10000?00?01110001000?0000??00111100010020110000001111??11100--001000000-000110011000-0011000????????0000?????01??00???1010100?100000-?????????????00011110-?0000-00-100?01001????0100211?1010000002000?00000000000?010100??1101?10-1?100?01011?0001000101010000011?0000?1001003000?0?????0?02????0?????0??????????????????????????????????????????????????????????????????????????????????????????????0100100000100100?00100001000000000-??0101000100100?00110-100?0-?????????????????????????????????????????????????????????????????????????????????????????????????????????????????????????????????????????????????????????????????????????????????????????????????????????????????????????????????????????????????????????????????????????????????????????????????????????????????????????????????????????????????????????????????????????????????????????????????????????????????????????????????????001?1000?00100??????000002000?100011101300100?-0012[01]11000110010?11110001?0211000100000010?????0????01?1??????001000010020100?0???00?0010?0??0000101110010?0010010010?0010110000000?10000101?10?0?1?000?00?010???????????????????????????????????????????????0011000101010111011?00?[12]0100???0001000?0??????????????????????????????????????????????????????????????????????????????????????????????????????????????????????????????????????????????????????????????????????????????????????????????????????????????????????????????????????????????????????????????????????????????????????????????????????????????????????????????????????????????????????????????????????????????????????????????????????????????????????????????????????????????????????????????????????????????????????????????????????????????????????????????????????????????????????????????????????????????????????????????????????????????????????????????????????????????????????????????????????????????????????????????????????????????????????????????????????????????????????????????????????????????????????????????????????????????????????????????????????????????????????????????????????????????????????????????????????????????????????????????????????????????????????????????????????????????????????????????????????????????????????????????????????????????????????????????????????????????????????????????????????????????????????????????????????????

*Eustreptospondylus* 11??10000110100001010020300?11?000?00100001100-010111100-0?01------020010100001------000-00-------??0??020100?1100----00000?1100??0001001000?0???????1??????????00?1-11011000--001101100000010-010?0????01000????????0??0?????01000100110001?0111?100-??10-00-1000????1?110-?0????0101001????????????0?????010000002000?0000000101000100-0110111010-11100101011?00?10001000200000011000001000?03000?0?0?[01]00002???00?00????????????????????????????1?00100?0200010001?000????????????????????????????????????????????????10???0??0???0100000000011000000010000001010000100010?0?01?000-010100100000001000100010000011100000001100000000100011??0??????0000010000?0010????????????????????????????????????????????????????????????????????????????????????????????????????????????????????????????????????????????????????????????????????????????????????????????????????????????????????????????????????????????????????????????????????????????????????????????????????????????????????????????????????????????????????????1???20000011?01100000010120?1???0???????100200001??????10?10001?1??00000???10??1001000100?11000012??????????00110111????????????00000?0?010000010011001???01101001000110110000010011000101?100??1?0000????1????????111?0???0121210?00???????????????????????00110101100011?011300?2?1?000001010100?1111110000110000100002111101?110102111??1001??000?????????????????????????????????????????????????????????????????????????????????????????????????????????????????????????????????????????????????????????????????????????????????????????????????????????????????????????????????????????????????????????????????????????????????????????????????????????????????????????????????????????????????????????????????????????????????????????????????????????????????????????????????????????????????????????????????????????????????????????????????????????????????????????????????????????????????????????????????????????????????????????????????????????????????????????????????????????????????????????????????????????????????????????????????????????????????????????????????????????????????????????????????????????????????????????????????????????????????????????????????????????????????????????????????????????????????????????????????????????????????????????????????????????????????????????????????????????????????????????????????????????????????????????????????????????????????????????????????????????????????????????

*Afrovenator* 1101100000101000110000001012112100?0010?101100-010200100-0??1------120010100001------010-00-------?????0???????1?101001??0??11010100110?00000?1???????0?201100??0?01010011100--00????????00010-01??0????01000????????0?0??????????????????????????????????????????????????????????????????????????????????????????02000100000???????0100-0110110?10-1?0001?1?11???0100?[01]?1010?????1?00?????0???????????0???0??0?0?0?100???????11?00??????????????????????????0??0?01??????00?????????1???011?1?0?????12100?2???010000000100100??0????00?0010000100?000000-0?0?01???000100010011010000-?0?0?1100010001000100????0?0??????001????00?0000100?111???0?110????01????2????????????????????????????????????????????????????????????????????????????????????????????????????????????????????????????????????????????????????????????????????????????????????????????????????????????????????????????????????????????????????????????????????????????????????????????????????????????????????????????????????????????????????????????????????0010101100000100120011000?????0?000201001?011001011000?????????00?0?????????????????????????????????????????????????????0000000001?????10?11001?0?011000010001101100001?001?000001???????100000???1?01010??????????0121?????00?0?????0?31001111?100001?10?01101011?011?2012?1?00?001011100011101100001100001000021?11010110???111011?011100??1???????????????????????????????????????????????????????????????????????????????????????????????????????????????????????????????????????????????????????????????????????????????????????????????????????????????????????????????????????????????????????????????????????????????????????????????????????????????????????????????????????????????????????????????????????????????????????????????????????????????????????????????????????????????????????????????????????????????????????????????????????????????????????????????????????????????????????????????????????????????????????????????????????????????????????????????????????????????????????????????????????????????????????????????????????????????????????????????????????????????????????????????????????????????????????????????????????????????????????????????????????????????????????????????????????????????????????????????????????????????????????????????????????????????????????????????????????????????????????????????????????????????????????????????????????????????????????????????????????????????????????

*Torvosaurus* 11?1000000101000010100203011011000?00100101100-021201000-0??1------1?????1?0??1------?10-00-------??001010???????0-?????????1100????00000101??????????1?2??10000??0101??1100???00?????????0010-00???????????????????????????????????????????????????????????????????????0?0-???????0-??????????????????????00??0??0?0001?00?0????1????010000???1?10-1000?1?1?11????10002?10100????11000??1?0??03??????????1?02??0?0??0?1??????11?0??????????0??0-0000??????2?0??0201?00?0200?1?01001?????01111?0?10?01?????0????10????00100110??0????00?0000000?????000?0-00???0000000100?10?00-1?0?0-???0??1????0?010?0??0??0??00111???0?0????00???011000111????????????010???????????????????????????????????????????????????????????????????????????????????????????????????????????????????????????????????????????????????????????????????????????????????????????????????????????????????????????????????????????????????????????????????????????????????????????????????????????????????????????????????????????????????????????????00???100?0010??110110001012000??00????00?101200001?011111011000?[01]??????????0???????????????????????????????????010?0?????????????00000100010010010011011010210010010001121100001100110001011100001110000??010?101001110000000121211000000100?0[12]???001101101000001010110101110113000[12]?1000002001010001110?1000011000010000212110101101021110?1001??0???????????????????????????????????????????????????????????????????????????????????????????????????????????????????????????????????????????????????????????????????????????????????????????????????????????????????????????????????????????????????????????????????????????????????????????????????????????????????????????????????????????????????????????????????????????????????????????????????????????????????????????????????????????????????????????????????????????????????????????????????????????????????????????????????????????????????????????????????????????????????????????????????????????????????????????????????????????????????????????????????????????????????????????????????????????????????????????????????????????????????????????????????????????????????????????????????????????????????????????????????????????????????????????????????????????????????????????????????????????????????????????????????????????????????????????????????????????????????????????????????????????????????????????????????????????????????????????????????????????????????????????

*Baryonyx* 2???010100201000030000303111002100?00100111200101021110000??0111120020111200001------100-00-------?????????????????????????????????????????????????????????????????????????????????????????????????????????????????????????????????????????????????????????????????????????????????????????????????????????????????????????????????????????????????????????????????????????????????????????????????????????????????????????????????????????????????????????????????????????????????????????????????????????????????????????????????????????????????????????????????????????????????????????????????????????????????????????????????????????????????????????????????????????????????????????????????????????????????????????????????????????????????????????????????????????????????????????????????????????????????????????????????????????????????????????????????????????????????????????????????????????????????????????????????????????????????????????????????????????????????????????????????????????????????????????111?0201011210110?0000???02???1??01????0?001202011?0??????0??0?1111100??????10001011?01??010210?011??????????001101010?[12]101???1??0100101100013001011100[12]?1001001001000110111110100011000101010?0???000?00??1??1?00??0?1?0100012111100010011010????????????10?0?11010?101?1110110[01]0?2?1?0????0011000????1????00110100????????110?0?????????????0???????????????????????????????????????????????????????????????????????????????????????????????????????????????????????????????????????????????????????????????????????????????????????????????????????????????????????????????????????????????????????????????????????????????????????????????????????????????????????????????????????????????????????????????????????????????????????????????????????????????????????????????????????????????????????????????????????????????????????????????????????????????????????????????????????????????????????????????????????????????????????????????????????????????????????????????????????????????????????????????????????????????????????????????????????????????????????????????????????????????????????????????????????????????????????????????????????????????????????????????????????????????????????????????????????????????????????????????????????????????????????????????????????????????????????????????????????????????????????????????????????????????????????????????????????????????????????????????????????????????????????????????

*Suchomimus* 2???01?000201000030000?2???1???????00000111100101011100100??01111200?????2?0??1------?00-00-------???101201????0?0?-???1?0?111???0?01?001000?01?????????????????00??0???????0--??100??????000?????0??1???????????????0??????????????????????????????????????????????????110-????0-???????????1??????????????3???00022000?01?1?2????0???0-1?01??1?10-??0??????11??0?1?0?1?1??10????11000001?01?0???????????0???????0??0???0????0100??????????0??0-?0?0????001010102?1??0??21001??10?????????????????100???0?????0100????0?0?10000?????1??001?0????0?0???00?????0?[01][01]00?0????00?11?10??0???01011?1001?010?0??0??0??10111???1?1??0?00????110001????????????????????????????????????????????????????????????????????????????????????????????????????????????????????????????????????????????????????????????????????????????????????????????????????????????????????????????????????????????????????????????????????????????????????????????????????????????????????????????????????????????????????????????????????????????????111102010112101?0?00000100200?1??0?????0??????????????????????????1????????????????????????????????????????????110??????1????????010010110?013001011100[12]???????100?0001121?1110100011000201?10?0???00000???1001?0001011?0100012111100010011010????????????100011?010?1000111011000?[12]?0?00?000011?00?11??110100110100100??41211010?102021??0?1?????????????????????????????????????????????????????????????????????????????????????????????????????????????????????????????????????????????????????????????????????????????????????????????????????????????????????????????????????????????????????????????????????????????????????????????????????????????????????????????????????????????????????????????????????????????????????????????????????????????????????????????????????????????????????????????????????????????????????????????????????????????????????????????????????????????????????????????????????????????????????????????????????????????????????????????????????????????????????????????????????????????????????????????????????????????????????????????????????????????????????????????????????????????????????????????????????????????????????????????????????????????????????????????????????????????????????????????????????????????????????????????????????????????????????????????????????????????????????????????????????????????????????????????????????????????????????????????????????????????????????????????????

*Irritator* ?020????????????????????????????????0200??1??0?11???????0??00101100???????????0011001?????0-------????????1??????0?-???????111?0?0?0??0?????1010?0?0??0?21-?????000101???0100--?01001????0?0?0-01?0???10?1???????????0??0??????100????0?0101???11?00????1?????0-???0??????????????????????1011???????0????????????02?????11?1??????????0-1?????????????????????????????????????????????????????????????0??????????????????????????????????????????????????????????????????????????????????????????????????????????0?????????????????????????????????????????????????????????????????????????????????????????????????????????????????????????????????????????????????????????????????????????????????????????????????????????????????????????????????????????????????????????????????????????????????????????????????????????????????????????????????????????????????????????????????????????????????????????????????????????????????????????????????????????????????????????????????????????????????????????????????????????????2????02?0???????01100200?100010??0000010?201110010000??00011?11000????010??10?10010001021000?101?10?????0????????????10?11?0???1101?1-??????1???????????????????????????????????????????????????????????????????????????????????????????????????????????????????????????????????????????????????????1????????????????????????????????????????????????????????????????????????????????????????????????????????????????????????????????????????????????????????????????????????????????????????????????????????????????????????????????????????????????????????????????????????????????????????????????????????????????????????????????????????????????????????????????????????????????????????????????????????????????????????????????????????????????????????????????????????????????????????????????????????????????????????????????????????????????????????????????????????????????????????????????????????????????????????????????????????????????????????????????????????????????????????????????????????????????????????????????????????????????????????????????????????????????????????????????????????????????????????????????????????????????????????????????????????????????????????????????????????????????????????????????????????????????????????????????????????????????????????????????????????????????????????????????????????????????????????????????????????????????????????????????????????????????????????????????????

*Spinosaurus* 2???01?1002010000300003231?2??2000??010011202010102011000???01011100????1200?01------100-00-------?????????????????????????????????????????????????????????????????????????????????????????????????????????????????????????????????????????????????????????????????????????????????????????????????????????????????????????????????????????????????????????????????????????????????????????????????????????????????????????????????????????????????????????????????????????????????????????????????????????????????????????????????????????????????????????????????????????????????????????????????????????????????????????????????????????????????????????????????????????????????????????????????????????????????????????????????????????????????????????????????????????????????????????????????????????????????????????????????????????????????????????????????????????????????????????????????????????????????????????????????????????????????????????????????????????????????????????????????????????????????????????110?1201011210????0??0000020??1??0?????0?????????????????????????????????????????????????????????????????????00110?0??011????????010110111001311?101100[12]??????????1000??21?0100?00???0?02010?00????000?0??0???????????????????????????????????????????????????????????????????????????????????????????????????????????????????????????????????????????????????????????????????????????????????????????????????????????????????????????????????????????????????????????????????????????????????????????????????????????????????????????????????????????????????????????????????????????????????????????????????????????????????????????????????????????????????????????????????????????????????????????????????????????????????????????????????????????????????????????????????????????????????????????????????????????????????????????????????????????????????????????????????????????????????????????????????????????????????????????????????????????????????????????????????????????????????????????????????????????????????????????????????????????????????????????????????????????????????????????????????????????????????????????????????????????????????????????????????????????????????????????????????????????????????????????????????????????????????????????????????????????????????????????????????????????????????????????????????????????????????????????????????????????????????????????????????????????????????????????????????

Spinosaurine_morphoI 2???010[01]00201[01]0[02]03000030311210[12][01]00?00[12]00111[02][02]01010[23]0110000??01011100301112[01]0001------100-00-------???????????????????????????????????????????????????????????????????????????????????????????????????????????????????????????????????????????????????????????????????????????????????????????????????????????????????????????????????????????????????????????????????????????????????????????????????????????????????????????????????????????????????????????????????????????????????????????????????????????????????????????????????????????????????????????????????????????????????????????????????????????????????????????????????????????????????????????????????????????????????????????????????????????????????????????????????????????????????????????????????????????????????????????????????????????????????????????????????????????????????????????????????????????????????????????????????????????????????????????????????????????????????????????????????????????????????????????????????????????????????????????????????????????????????????????????????????????????????????????????????????????????????????????????????????????????????????????????????????????????????????????????????????????????????????????????????????????????????????????????????????????????????????????????????????????????????????????????????????????????????????????????????????????????????????????????????????????????????????????????????????????????????????????????????????????????????????????????????????????????????????????????????????????????????????????????????????????????????????????????????????????????????????????????????????????????????????????????????????????????????????????????????????????????????????????????????????????????????????????????????????????????????????????????????????????????????????????????????????????????????????????????????????????????????????????????????????????????????????????????????????????????????????????????????????????????????????????????????????????????????????????????????????????????????????????????????????????????????????????????????????????????????????????????????????????????????????????????????????????????????????????????????????????????????????????????????????????????????????????????????????????????????????????????????????????????????????????????????????????????????????????????????????????????????????????????????????????????????????????????????????????????????????????????????????

Spinosaurine_morphoII ?????10100201?0??30000304201102000?????02?1[02]2?11132001000???01011000?????2?0??1------100-00-------???????????????????????????????????????????????????????????????????????????????????????????????????????????????????????????????????????????????????????????????????????????????????????????????????????????????????????????????????????????????????????????????????????????????????????????????????????????????????????????????????????????????????????????????????????????????????????????????????????????????????????????????????????????????????????????????????????????????????????????????????????????????????????????????????????????????????????????????????????????????????????????????????????????????????????????????????????????????????????????????????????????????????????????????????????????????????????????????????????????????????????????????????????????????????????????????????????????????????????????????????????????????????????????????????????????????????????????????????????????????????????????????????????????????????????????????????????????????????????????????????????????????????????????????????????????????????????????????????????????????????????????????????????????????????????????????????????????????????????????????????????????????????????????????????????????????????????????????????????????????????????????????????????????????????????????????????????????????????????????????????????????????????????????????????????????????????????????????????????????????????????????????????????????????????????????????????????????????????????????????????????????????????????????????????????????????????????????????????????????????????????????????????????????????????????????????????????????????????????????????????????????????????????????????????????????????????????????????????????????????????????????????????????????????????????????????????????????????????????????????????????????????????????????????????????????????????????????????????????????????????????????????????????????????????????????????????????????????????????????????????????????????????????????????????????????????????????????????????????????????????????????????????????????????????????????????????????????????????????????????????????????????????????????????????????????????????????????????????????????????????????????????????????????????????????????????????????????????????????????????????????????????????????????????????????

*Allosaurus*_*fragilis* 1000000000102001110100221001111000110100100111101000000010100111110031220011101------100-00-------??0010[01]01001010?0000?0000?1000010?110?0100100001?1?011?1-00000000101???[01]100--?11101000-01000-010?0???01?00020?00010??00010010100?001000101001100100-1?10-?0-0-00000010000-?0000-00-1000011111?000100101010200000021001000000000000010100000111010-10000001011000010011010001000010000001001003000000000000020000001001000???1?0100???100??000110101000000100000000100000000000000101000011110001010121000211100000000010010100010001000010000100000000101000?1010000100000011010000-00010110001100110010001000101110000?00010000000110001110??0011000000110?020010???????????????????????????????????????????????????????????????????????????????????????????????????????????????????????????????????????????00000100000000000000000010000000200001000000000000001000000111011000110101001100000010000000001011110020100000100000000100000000000000000000100000001000000?00000[01]00000100011001000001000010200121011000000100101[01]000001000101000000021111110001101100002[01]2[01]00101100000001011111000110201101100011010010010201201111111112000100110201111111110000100011020010011001100110010[01]100001011000100001000101111000011?0000000101?11011012001200121010110000100011131001111110000111010010211110110201201?1100200111000[12]1011101111000011001021221010111102111012101110010101000000000001010000001000000100011000000000021021000100000010001????0000000001?0??100?00000?000000000000000100000000?0?10000000000????000?0001001000000001000000000000000000000200000000000000000000100000010000002001000[01]0000100000000010000000000001001010000000000100?000101000100000010201100000000000110000000000000100001?000000010000000000110120001001000000000000002?0002000?000000000001000000000000100000000000000001000000000000000000000000000000100100001000000000001000000100000000000000?000?000000010????000000000000000000000000000010001010000000000000000000000100100000001000000000100000001000000000000001000000000000100000000000001000100000010000000000000000000000000000000000000000000?11000?0000000000010001000100000000101100201020000000001000000000000001000000000101010010000000100100000000001?010000000011?00000000000010001010001010000001000001000000001000000001100000010000000000000000000000000000000000000000000000000000000000000100000000000000000000000000000000000100000000001000000000000000000000000000000000000000000000000000000000000

*Allosaurus*_*europaeus* ?00000?00010???1??????????????????0?0??0??0??0101???0?0000100111110??1220001100011011102200-------???????????????????????????????????????????????????????????????????????????????????????????????????????????????????????????????????????????????????????????????????????????????????????????????????????????????????????????????????????????????????????????????????????????????????????????????????????????????????????????????????????????????????????????????????????????????????????????????????????????????????????????????????????????????????????????????????????????????????????????????????????????????????????????????????????????????????????????????????????????????????????????????????????????????????????????????????????????????????????????????????????????????????????????????????????????????????????????????????????????????????????????????????????????????????????????????????????????????????????????????????????????????????????????????????????????????????????????????????????????????????????????????????????????????????????????????????????????????????????????????????????????????????????????????????????????????????????????????????????????????????????????????????????????????????????????????????????????????????????????????????????????????????????????????????????????????????????????????????????????????????????????????????????????????????????????????????????????????????????????????????????????????????????????????????????????????????????????????????????????????????????????????????????????????????????????????????????????????????????????????????????????????????????????????????????????????????????????????????????????????????????????????????????????????????????????????????????????????????????????????????????????????????????????????????????????????????????????????????????????????????????????????????????????????????????????????????????????????????????????????????????????????????????????????????????????????????????????????????????????????????????????????????????????????????????????????????????????????????????????????????????????????????????????????????????????????????????????????????????????????????????????????????????????????????????????????????????????????????????????????????????????????????????????????????????????????????????????????????????????????????????????????????????????????????????????????????????????????????????????????????????????????????????????????????????

*Allosaurus*_*'jimmadseni'* 10?0100000101001110100221001111000?10100?00??1101???000010?001011100212?1010100011011102200-------???????????????????????????????????????????????????????????????????????????????????????????????????????????????????????????????????????????????????????????????????????????????????????????????????????????????????????????????????????????????????????????????????????????????????????????????????????????????????????????????????????????????????????????????????????????????????????????????????????????????????????????????????????????????????????????????????????????????????????????????????????????????????????????????????????????????????????????????????????????????????????????????????????????????????????????????????????????????????????????????????????????????????????????????????????????????????????????????????????????????????????????????????????????????????????????????????????????????????????????????????????????????????????????????????????????????????????????????????????????????????????????????????????????????????????????????????????????????????????????????????????????????????????????????????????????????????????????????????????????????????????????????????????????????????????????????????????????????????????????????????????????????????????????????????????????????????????????????????????????????????????????????????????????????????????????????????????????????????????????????????????????????????????????????????????????????????????????????????????????????????????????????????????????????????????????????????????????????????????????????????????????????????????????????????????????????????????????????????????????????????????????????????????????????????????????????????????????????????????????????????????????????????????????????????????????????????????????????????????????????????????????????????????????????????????????????????????????????????????????????????????????????????????????????????????????????????????????????????????????????????????????????????????????????????????????????????????????????????????????????????????????????????????????????????????????????????????????????????????????????????????????????????????????????????????????????????????????????????????????????????????????????????????????????????????????????????????????????????????????????????????????????????????????????????????????????????????????????????????????????????????????????????????????????????????

*Aerosteon* 1???300000001001110000201001111110?101012010010013100100-0??001112002122001000011121?000-01100--02???????????????????????????????????????????????????????????????????????????????????????0??10-???????????????????????????????????????????????????????????????????????????????????????????????????????????????????????000?0?????????01?100?????0?11111?00???????????001201?0010001111?0?01001?03???????0??10??????1????????????????????100??????????????????????????????????????????????????????????????????????????????110100000100?0010?00000100?000?010100?011--00??????????????????????????????????????????????????????????????????????????????????????????????????????????????????????????????????????????????????????????????????????????????????????????????????????????????????????????????????????????????????????????????????????????????????????????????????????????????????????????????????????????????????????????????????????????????????????????????????????????????????????????????????????????????????????????????????????????????????????????????????????????????01?000?111?????????????????????????????????????????????????????????????????????????????????110121???????11?01001?1?00010001011010111?????2?110??0?????????0101100121????????????????????????????????01011010010221110110201201?120??????????????????????????1001?412????01112021110???????????????????????????????????????????????????????????????????????????????????????????????????????????????????????????????????????????????????????????????????????????????????????????????????????????????????????????????????????????????????????????????????????????????????????????????????????????????????????????????????????????????????????????????????????????????????????????????????????????????????????????????????????????????????????????????????????????????????????????????????????????????????????????????????????????????????????????????????????????????????????????????????????????????????????????????????????????????????????????????????????????????????????????????????????????????????????????????????????????????????????????????????????????????????????????????????????????????????????????????????????????????????????????????????????????????????????????????????????????????????????????????????????????????????????????????????????????????????????????????????????????????????????????????????????????????????????????????????????????????????????????????????

*Sinraptor* 0000000000101001010200201001001000210100101011000100010010100111100031220110100011010001200-------??001000000000?10000000?010-000110110000?000000100000020000000010101??11210--?11001100-010010011000-00010003000001000010000101000001000101000100100-??10-?0-0-0?001011000-?0000-010100?0111010000??010101010000002100?00000000000?010100110111010-10100001011?00110011010101010011000001?0100300000?????0002?00?0??1?1001???1??1??10????000?????1010100?0??????0????????????????????????????0??11101210?????1000000100100101001???000?0?100001000000001100??11010000100000011010000-00010?1000110011001000101010111?000000?00?000000100001100?0011000000110?0100?0???????????????????????????????????????????????????????????????????????????????????????????????????????????????????????????????????????????????????????????????????????????????????????????????????????????????????????????????????????????????????????????????????????????????????????????????????????????????????????????????????????????????????????00001000000000000000010002111111000110100000211000011100010001001?1100001120110110001001001001120100111?11111000000110020011111000000010?0?100001001100110110001001000012110001100011001211010?0?1?000?000010???1?01011100??????????????????????2???????11?00020101001010111011220121110000210110101110111011110000110000221210101101021110121011100101??????????????????????????????????????????????????????????????????????????????????????????????????????????????????????????????????????????????????????????????????????????????????????????????????????????????????????????????????????????????????????????????????????????????????????????????????????????????000000000000100000?000000?1000000000011012000100000000000000000200002?00?00?00?000001000000000000100?000000000000010001000?0?00000000000000000000100100001000000000?011000001000000000?0000????????????????????000000??????0?????????????????????0???00000000????????????10???001???00???001000?000100?0000000000000000000000001000000000000?1000100000010000000000000000000000000?00000000000000000?110000000000?000010001000100000001101100201020000000001000000000000001000000000101010010000000100100000000001?010000000011?0000?0??0????????????01010000001000001000000001000000001100000010000000000000?00??000000000000??0000000000000000000000000000010000000000000?????????????????????????????????1000000000010000000000????0000000000??000?0010?00000000000000

*Acrocanthosaurus* 1000000000100001020200203000001000120100??1??0000???0???0010010120002122010?100011011002101202100????010100?0??101?0??10?0?10-?0?1?0110001001000?0?0?0112011??0?0001010??1110--?11?0?????01101111?00??10110?020?0??1?0??0?????010?0???1?0101?0?10000????10-????????0????200-?0000-?10???0?10111???0??0?0??101??000021001?00?0?0??1?0???1000?1??1?111110??001?11??0?10??2?1??01????10?1?00??01?0?1?????????0?02??????10?1?0????1?010?????????0???1?101?10?0?100000??10???02??00?101??0?0??01111?0?10100220002??100?0???00???10?????????????10??????????101010??0?0[01]0??0????00?11?10?00???01012?1001?011?0??00100?101?????0?0?????0??0??100????00?000-00??0?110??2?010???????????????????????????????????????????????????????????????????????????????????????????????????????????????????????????????????????????????????????????????????????????????????????????????????????????????????????????????????????????????????????????????????????????????????????????????????????????????????????????????????????????????????????020?1100?0000001111000000211111100010?000000211101211100020212011?112111102011012100000100110100012011111?111002100101020010?11111?000100010000010011012?00110011?00?0012110001000110011211110?0021000000101?1?110110120012001210?001011110000113110111111000????010?10??1110110201201?120?201111000220111011110000110010312?1010?1110?1??01210111001?1???????????????????????????????????????????????????????????????????????????????????????????????????????????????????????????????????????????????????????????????????????????????????????????????????????????????????????????????????????????????????????????????????????????????????????????????????????????????????????????????????????????????????????????????????????????????????????????????????????????????????????????????????????????????????????????????????????????????????????????????????????????????????????????????????????????????????????????????????????????????????????????????????????????????????????????????????????????????????????????????????????????????????????????????????????????????????????????????????????????????????????????????????????????????????????????????????????????????????????????????????????????????????????????????????????????????????????????????????????????????????????????????????????????????????????????????????????????????????????????????????????????????????????????????????????????????????????????????????????????????????????

*Shaochilong* 1???0?0000100000020100203001201100?2010????????010200000?0?????????03122010000???????00??00-------???????????????????????????????????????????????????????????????????????????????????????????????????????????????????????????????????????????????????????????????????????????????????????????????????????????????????????????????????????????????????????????????????????????????????????????????????????????????????????????????????????????????????????????????????????????????????????????????????????????????????????????????????????????????????????????????????????????????????????????????????????????????????????????????????????????????????????????????????????????????????????????????????????????????????????????????????????????????????????????????????????????????????????????????????????????????????????????????????????????????????????????????????????????????????????????????????????????????????????????????????????????????????????????????????????????????????????????????????????????????????????????????????000??11?10100100110111?00???1???????????????????????????1?011?????11???1?0000100?0011??120?????????????????????????????1????????????????????????????????????????????????????????????????????????????????????????????????????????????????????????????????????????????????????????????????????????????????????????????????????????????????????????????????????????????????????????????????????????????????????????????????????????????????????????????????????????????????????????????????????????????????????????????????????????????????????????????????????????????????????????????????????????????????????????????????????????????????????????????????????????????????????????????????????????????????????????????????????????????????????????????????????????????????????????????????????????????????????????????????????????????????????????????????????????????????????????????????????????????????????????????????????????????????????????????????????????????????????????????????????????????????????????????????????????????????????????????????????????????????????????????????????????????????????????????????????????????????????????????????????????????????????????????????????????????????????????????????????????????????????????????????????????????????????????????????????????????????????????????????????????????????????????????????????????????????????????????????????????????????????????????????????????????

*Giganotosaurus* 1???000000100000020000202001211000?20100211100011000100000??02012000?????1?0?0001?01?0021010-10000???0?00????????0????10????10???1?001000100??01?1?0????2??????????1010??1110--??1?0?????01101111?01??1??????[12]??????????1??????????0??0??101????????????1?????0-???1????201-?0?0???10???0???1?11????????????1???000200?0?10?0?0??0?0???110001??1?111110??[01]?1?11??0?100?2?0??0?????10010??1?01?????0???0?000?01??????000???0???1000??????????0???????1?10?0?????????????????????????????????????????????????????????????0???10000?110?1??00?000?11?????101010??010100?0???????11?1???????01012?100??01??0?????0??10111???0?0??1?00????1?????????????????????????????????????????????????????????????????????????????????????????????????????????????????????????????????????????????????????????????????????????????????????????????????????????????????????????????????????????????????????????????????????????????????????????????????????????????????????????????????????????????????????????????????????????????????????02???000??0?00?11?100001001101???10?1110100021110????????2021211??11211??0??110121???00100?0011211?1??????111??201010?????1?1111110?0020000??00010011012?01?0??111001?01?1?00?100?11001?21?2?????2?00000???1??0?10??0???01?0????????????????????????????????00?11010010211110110201201?120020111100022011101??1?0???1001?31221010?10??????????????????????????????????????????????????????????????????????????????????????????????????????????????????????????????????????????????????????????????????????????????????????????????????????????????????????????????????????????????????????????????????????????????????????????????????????????????????????????????????????????????????????????????????????????????????????????????????????????????????????????????????????????????????????????????????????????????????????????????????????????????????????????????????????????????????????????????????????????????????????????????????????????????????????????????????????????????????????????????????????????????????????????????????????????????????????????????????????????????????????????????????????????????????????????????????????????????????????????????????????????????????????????????????????????????????????????????????????????????????????????????????????????????????????????????????????????????????????????????????????????????????????????????????????????????????????????????????????????????????????????????????????????

*Mapusaurus* 1???0000001000000?000??0??0????????1010022110001102000?0?0??0101200021?211100000100110021010-11100?????0?????????0?-??10????10?0???001000100?1?1?1?0??1?2??1??????01010????1????1100?????011?1111???????????????????????????????????????????????????????????????????????201-?0?????10???0???1?1???0??0101?????????0?00?0?10?0?0??0?0???110??1????11111???001?11??0?100?2????0?????10010001?0????1?????????0?01??????1001??????1???0????10?????????1?1??0???100?0???11?1????????????????????????0??0??????????????0?????0???10000?110???????00??110????10??????????????????00?11?10?00???0?012?1001?011?0????10??10?110000?00?0?00???1?100011??0?????????0?110????????????????????????????????????????????????????????????????????????????????????????????????????????????????????????????????????????????????????????????????????????????????????????????????????????????????????????????????????????????????????????????????????????????????????????????????????????????????????????????????????????????????????????????????????0???00000111100001001101?101011110100021110??1111102?2121121????????[02]0?????????????????????????????????00201010?01001??????1000020000?0???10011012???01???1??0?0010??0001??01?00102012?????210000?????????1?11012?0????1???10?10???????0????1????????000???0?0?102?1110110[12]?????????0?011?100022??1101111?00?1100??31221010110??2111012101??0010????????????????????????????????????????????????????????????????????????????????????????????????????????????????????????????????????????????????????????????????????????????????????????????????????????????????????????????????????????????????????????????????????????????????????????????????????????????????????????????????????????????????????????????????????????????????????????????????????????????????????????????????????????????????????????????????????????????????????????????????????????????????????????????????????????????????????????????????????????????????????????????????????????????????????????????????????????????????????????????????????????????????????????????????????????????????????110????????????????00????1000000????1100????????????????????????0????????????00101010?100?????100100000?????1?0100???0011?????001?00????????????????00000010000010????000????00000110000001000000??00??0?????100?0???????000000?????0?00000000???0?0?000??000???000??000?0?????????????????????????????0?0000?00000?00???????????00?0?0??0?00??0???????0???????1???0

*Ornitholestes* ?101?00000112?0??10100101000201011????0??00?002???????0?10??0101??002122[12]00??0000111???22?0-------??00?0000?01?00101011100??10000?01101000000000?0?00111000110000010-10011100--00101?000?10000-000201000?0000[23]0????100000???0?0???0?00111101?100-????-??????????????0010?10-?0010-00-100?00-011100011010001012011102101-001000000?010100-????????10-1?101200011??1111?11?101??01001??1001010100?0??10?0?10?0020000?10?0??????????1?1??????????????????????010?0100001001?0??0?0?0?0?????0???????????????????????????????1?0101??0100000?0001001100000011????010?01000010?010001?10000-0????????????10?0?10?????????????????00??0?????????????00??????0?100??0?0?0?10???????????????????????????????????????????????????????????????????????????????????????????????????????????????????????????????????????????????????????????????????????????????????????????????????????????????????????????????????????????????????????????????????????????????????????????????????????????????????????????????????????????????????????000??000?00000??????000001100?1?00001000001010?0012?11000001000?1??10001?0201000?00???0????0??0??????????????00000?0??0?0?0?0100111?000000??00001001100[12]?????????0100101?0?000?0001000000010100??1?00010??010??11??????????00111100000000???01??11??111??1??00?0001011121111011020????????010010000021??211??0100001???????2??0?0???????????2001??0??????????????????????????????????????????????????????????????????????????????????????????????????????????????????????????????????????????????????????????????????????????????????????????????????????????????????????????????????????????????????????????????????????????????????????????????????????????????????200000000000110010?10011011011?00?0?0000000001101101010000001?1??00?100??1??0?00?00??101?0?0???001???00000000000100000000000?00000100000000?000001?0100???1011000?0?0011001010000??100?000??000????11???????????????????????1100000110000?00?????1?????0????0?0??0????????????0000001000?001000?000010?00010000010???001001000010000101?0000??00??0?????????0101010????000000?000?????????001???0000?0110???0?0?00?1???0?010?011100001?000000?00?10000?01011?????00000000000?00000?0101000???00011?110110000??100??0010??1????????????????0?000??????00010001??010?0010?000101?10000001??0????????00000????00000?000?00?0?00?00010000100010010?01??1000?100?000?00001110???????11010?0????????0?????000100??010010?0?101?0???????0????000000100000??0??1101??0000000001?1

*Bicentenaria* ??01?00000112?0??10000201001001001?????????????0101101001???01?1??00????220000???????10??00-------???????????????????????????????????????????????????????????????????????????????????????????????????????????????????????????????????????????????????????????????????????????????????????????????????????????????????????????????????????????????????????????????????????????????????????????????????????????????????????????????????????????????????????????????????????????????????????????????????????????????????????????????????????????????????????????????????????????????????????????????????????????????????????????????????????????????????????????????????????????????????????????????????????????????????????????????????????????????????????????????????????????????????????????????????????????????????????????????????????????????????????????????????????????????????????????????????????????????????????????????????????????????????????????????????????????????????????????????????????????????????????????????????????????????????????????????????????????????????????????????????????????????????????????????????????????????????????????????????????????????????????????????????????????????????????????????????????????????????????????????????????????????????????????????????????????????????????????????????????????????????????????????????????????????????????????????????????????????????????????????????????????????????????????????????????????????????????????????????????????????????????????????????????????????????????????????????????????????????????????????????????????????????????????????????????????????????????????????????????????????????????????????????????????????????????????????????????????????????????????????????????????????????????????????????????????????????????????????????????????????????????????????????????????????????????????????????????????????????????????????????????????????????????????????????????????????????????????????????????????????????????????????????????????????????????????????????????????????????????????????????????????????????????????????0??0??00??????????????????????????0????0010?100000?101??0??????0?0000?0000??000???0100????????????0?0?00???100??????000???[01]0??10??????0??????100000010110???0011000?0?0?00??0?0?0?00???00????00?0?0??????00????1????????????1?10????1????????0???????????????0?????000000???????0??0?????0??????0????00??0??????0?????0?0???????10?00011

*Zuolong* 1?0?0000001110000200001010?1201011?00?00??0??0201????10000??01112210220021000000010010022?0-------??00??00?00?010101001?0?0?0-0000001110000000??????00???????0??01?0-0--1?10111011????0??00000-0?1100-00?1000300??0????0??????????????????????????????????????????????????????????????????????????????????10100001020?0000100???????010100???????10-0?101200011??0?10??00?01???????????????????40001?000??000000??0?00????????????????????????????1?1?1???010001???010???????00?000???????????????????????????????0???00100??????????00?0??0???100?0000011?0010112?00???????????????????1?011000100001001000001010111?0???011??000????????????00????10001011000??????????????????????????????????????????????????????????????????????????????????????????????????????????????????????????????????????????????????????????????????????????????????????????????????????????????????????????????????????????????????????????????????????????????????????????????????????????????????????????????????????????????????????????????????????????????????????????????????????????????????????????????????????????????????????????????????????????????????????????????????????????????????????????????????????????????????????????????????????????????????????????????????????????????????????????????????????????????????????????????????????????????????????????????????????????????????????????????????????????????????????????????????????????????????????????????????????????????????????????????????????????????????????????????????????????????????????????????????????????????????????????????????????????????????????????????????????????????????????????????????????????????????????????????????????????????????????????????????????????????????????????????????????????????????????????????????????????????????????????????????????????????????????????????????????????????????????????????????????????????????????????????????????????????????????????????????????????????????????????????????????????????????????????????????????????????????????????????????????????????????????????????????????0?1???????????????0000?001000?????00002?10?0???00???????????0?????????????0?01?10???1?0100010000???0???01000??00????????????????????000?0???????0?0???0010?0????????????01000100?0?10?00?0???00010???00??0??0????????????0010?????0?00000??????0????????0?1????0??????????10000??????????0??????????????????01?0101?0??????????????100?00000??????0?0???000?0000?11?

*Proceratosaurus* 1?203000001000000?000??????????????????0???????00???0???00?00211110?013??11?001------000-00-------??10??000000011101001100111000?001110000???0??1???010?01-10001000?????01?0????01??????????00-?0??????0110002?0??0??0?000110101?011?????????????0?????????????????0001?000-00001000-100000-1111100??0???01010000102101-0010001000010100-0?????????????????????????????????????????????????????????????????????????????????????????????????????????????????????????????????????????????????????????????????????????????????????????????????????????????????????????????????????????????????????????????????????????????????????????????????????????????????????????????????????????????????????????????????????????????????????????????????????????????????????????????????????????????????????????????????????????????????????????????????????????????????????????????????????????????????????????????????????????????????????????????????????????????????????????????????????????????????????????????????????????????????000??000?00100??????000002100?1??0????????0???????201100000????????????1??20??????????????????0??????????????00[02]00?0??0???00??1?1?110000?0??0000000????????????????????????????????????????????????????????????????????????????????????????????????????????????????????????????????????????????????????????????????????????????????????????????????????00?1001101111?01000?10100?00?1?000010?1???0?0?????????????????0?1000100000110001?????0??0??0?0???1000000??0???????????????0000??000?????0??0?0???????00?100???????0001???110?000000001000?????111000???????????????????????????????????????????????????????????????????????????????????????????????????????????????????????????????????????????????????????????????????????????????????????????????????????????????????????????????????????????????????????????????????????????????????????????????????????????????????????????????????????????????????????????????????????????????????????????????????????????????????????????????????????????????????????????????????????????????????????????????????????????????????????????????????????????????????????????????????????????????????????????????????????????????????????????????????????????????????????????????????????????????????????????????????????????????????????????????????????????????????????????????????????????????????????????????????????????????????????????????????????????????????????????????????????????????????

*Guanlong* ?0200????????????100?0201???20?010??0?00?111000?111001000???0?0??1???????10???1------?????0-------??101000000111110100110011110000?011100001001011000101201100010000-11011100--0?1000100-00000-001200-0011010300?1???00000100001000001110001000??000100010-110100000001?000-00000-010100?00-1111??1??0????101000010200[01]1001000?000000100-1??????010-10101000010?1001001001010000001001001100100300010?00100000000?0??2000000?????11?????????00010?1?10000?01001100001001110001000001010000010101011100210002121000????001001010101000000002100110010000110110001000000100000000-100010011?01100011001100100000101111100?2000?1?0010001100111100?00110000001100021010??????????????????????????????????????????????????????????????????????????????????????????????????????????????????????????????????????????????????????????????????????????????????????????????????????????????????????????????????????????????????????????????????????????????????????????????????????????????????????????????????????????????????????????????????????????????????????????????????????????????????????????????????????????????????????????????????????????????????????????????????????????????????????????????????????????????????????????????????????????????????????????????????????????????????????????????????????????????????????????????????????????????????????????????????????????????0001000101111001000?10100?00?10000010?1000000?003??00000??0??00?10001000001100010??010?10000?00001000000100000011?00100000?00000?????????????020?010000010000000000000????10?0000000010???????1010100???????0000001000001010000?0?000000100000010000000000001000000000001000000000000?0010000000010000000?0000000001000000100001?000000110101000010000?21001010000000000000????00011??????0?00000??000?0001????????0000000000010?0001000?0?000000100100001000001101000???????00?0?0??00?10?0??0??000?0??????0??????00????????1?0??0??0??0011000001000?11???111010010?0?000?001001000100000100000000000003100000001000010001000??100001000000?100?00?01?000?1000?0??0????0001?101000000??0?0??001?0000000000??00000?????????????????????????????????????????????????????????????????????????????????????????????????????????????????????????????????????????????????????????????????????????????????????????????????????????????????????????????????????????????????????????????????????????????????????????????????????????????????????????????????????????????????????????????????????

*Eotyrannus* ?????00000100?0??10000101001110001????????110?00002001000???02??2?00?????1?1??1------100-00-------???????????????????????????????????????????????????????????????????????????????????????????????????????????????????????????????????????????????????????????????????????????????????????????????????????????????????????????????????????????????????????????????????????????????????????????????????????????????????????????????????????????????????????????????????????????????????????????????????????????????????????????????????????????????????????????????????????????????????????????????????????????????????????????????????????????????????????????????????????????????????????????????????????????????????????????????????????????????????????????????????????????????????????????????????????????????????????????????????????????????????????????????????????????????????????????????????????????????????????????????????????????????????????????????????????????????????????????????????????????????????????????????????????????????????????????????????????????????????????????????????????????????????????????????????????????????????????????????????????????????????????????????????????????????????????????????????????????????????????????????????????????????????????????????????????????????????????????????????????????????????????????????????????????????????0??11?100?01110????????00?0?01????20110000?1010?0?1??0?00?00????????????????0??????????????????????????0?????????????????????001??????0?00????????????????????????000?100002110100?????????2?2??00???00??0??00???1?0???????????00???011011011??0000???00?2???????????????????????????????????01???000?00100?????????????????????????????????????????????????????????????????????????????????????????????????????????????????????????????????????????????????????????????????????????????????????????????????????????????????????????????????????????????????????????????????????????????????????????????????????????????????????????????????????????????????????????????????????????????????????????????????????????????????????????0?1?????????????0?0?????????????????????00??0?0???????0000?10??01???00??1?1???????????????????????????????0?0?0?000????????000???????????????????????????????????????0???00????????????0?0???00??????00???00??00?00??11??0?0?????01?111?00?0?????00????11?0??0??1?0?0??0?0??0?00????????????????????????????1??????1??000??0?00?0???00??????????

*Xiongguanlong* 1020000000102?0?0100001010010010112?0100??2????10???010?00??0211220???????0?001------?00-?0-------????????????????????????????????????????????????????????????????????????????????????????????????????????????????????????????????????????????????????????????????????????????????????????????????????????????????????????????????????????????????????????????????????????????????????????????????????????????????????????????????????????????????????????????????????????????????????????????????????????????????????????????????????????????????????????????????????????????????????????????????????????????????????????????????????????????????????????????????????????????????????????????????????????????????????????????????????????????????????????????????????????????????????????????????????????????????????????????????????????????????????????????????????????????????????????????????????????????????????????????????????????????????????????????????????????????????????????????????????????????????????????????????????????????????????????????????????????????????????????????????????????????????????????????????????????????????????????????????????????????????????????????????????????????????????????????????????????????????????????????????????????????????????????????????????????????????????????????????????????????????????????????????????????????????00100?1102000????0??1??00?01?0?01?0001001010??0010?00100??0??0??0?????0???01000?0???00?01000???0021?1111?1????100?1?111??100?100????????????00100100000?10?1??????????????????????????????????2?2?000??000000000110000010??????????????????????????????????11?101??0011??????????????01010012??????????????????????????????????????????????????????????????????????????????????????????????????????????????????????????????????????????????????????????????????????????????????????????????????????????????????????????????????????????????????????????????????????????????????????????????????????????????????????????????????????????????????????????????????????????????????????????????????????????????????????????????????????????????????????????????????????????????????????????????????????????????????????????????????????????????????????????????????????????????????????????????????????????????????????????????????????????????????????????????????????????????????????????????????????????????????????????????????????????????????????????????????????????????????????????

*Alioramus* 10003000000022100?0[01]00?0?001?0?1???00100212001120310010000?10101220011312201001------000-010-0-200????????????????????????????????????????????????????????????????????????????????????????????????????????????????????????????????????????????????????????????????????????????????????????????????????????????????????????????????????????????????????????????????????????????????????????????????????????????????????????????????????????????????????????????????????????????????????????????????????????????????????????????????????????????????????????????????????????????????????????????????????????????????????????????????????????????????????????????????????????????????????????????????????????????????????????????????????????????????????????????????????????????????????????????????????????????????????????????????????????????????????????????????????????????????????????????????????????????????????????????????????????????????????????????????????????????????????????????????????????????????????????????????????????????????????????????????????????????????????????????????????????????????????????????????????????????????????????????????????????????????????????????????????????????????????????????????????????????????????????????????????????????????????????????????????????????????????????????????????????????????????????????????????????????????001011??2?????0000011110111110?0111012011010120121010102111010111000100001020000100001001011100113201111011012100111111111?101110010011101000110110111111011011111011110010111212211110??10000????00000100000111110?11011?21011111??????????????????????????1?111110110?????????21?11?10111121??00?11111?11?01?????????????????????????????????????????????????????????????????????????????????????????????????????????????????????????????????????????????????????????????????????????????????????????????????????????????????????????????????????????????????????????????????????????????????????????????????????????????????????????????????????????????????????????????????????????????????????????????????????????????????????????????????????????????????????????????????????????????????????????????????????????????????????????????????????????????????????????????????????????????????????????????????????????????????????????????????????????????????????????????????????????????????????????????????????????????????????????????????????????????????????????????????????????

*Albertosaurus* 20013000000022100?0000???001??0???2?1?00212020100310010100?101112210013122??001------100-010-10210??1????0000011??0????10?1???0??0??0100000101110?11??00201100110011111011201??000-1200010111100112010101?0103000001000000000001000101?1000?00110000100?10-?10??0000001??00-?0?00-00-1000010111?011100???010??00?10?00??0?0000000?0001010???011?0???????0000011?00????0?0?0???????1??0000?00?00410?00???10??0??00??0??0?00?0????0??0000??01000010???00?000011?001???????00??0?00??01000000010?0???????320?0??-?0000????????10101?10000000?20???1000000?010??0001???00010000000102000101?1101100110000100100000001?1110011001?1000100?2000110?00?001100000111000?001???????????????????????????????????????????????????????????????????????????????????????????????????????????????????????????????????????????????????????????????????????????????????????????????????????????????????????????????????????????????????????????????????????????????????????????????????????????????????????????????????????????????????????????????????????????????????????????????????????????????????????????????????????????????????????????????????????????????????????????????????????????????????????????????????????????????????????????????????????????????????????????????????????????????????????????????????????????????????????????????????????????????????????????????????????????????01101011120000000000[01]12001[01]1001011101201201111002111[01]1[02]1?01?0010000001000102010020011100100101?113101111011012100111111111000[01]000110[01][01]1111000010110111101111110111111110010111212111100?12?10?2121011????????11??????????????????01111110212101111111?010112111111111101211111012111??1???1???1111111111111201?????????????????????????????????????????????????????????????????????????????????????????????????????????????????????????????????????????????????????????????????????????????????????????????????????????????????????????????????????????????????????????????????????????????????????????????????????????????????????????????????????????????????????????????????????????????????????????????????????????????????????????????????????????????????????????????????????????????????????????????????????????????????????????????????????????????????????????????????????????????????????????????????????????????????????????????????????????????????????????????????????????????????????????????????????????????????????????????????????????????????????????????????????????

*Daspletosaurus* 200130000000221001000010000?10?0112?01001020011?1010010110?10111?200013?2201001------100-010-??200??100020100111110100??00?10-00000101?000???11101??????2??000110011111011101100011?0101?000?0-0112010101101?30???01?0000??????????0????????????????100?10-?0-100000??1?000-0000??00-100001011????????211?10100001020000000000000000010100??????????????0???0????0????????????????????????????0???????????????????????????????1?111????????????1???????????????01????????????????????????????????????????????-????????0???????????????????20??????????????????????????????????????????????01?0002???1?0?10?????01?111???2?????????????????????????????????????????????????????????????????????????????????????????????????????????????????????????????????????????????????????????????????????????????????????????????????????????????????????????????????????????????????????????????????????????????????????????????????????????????????????????????????????????????????????????????????????????????????????????????????????????????????????????????????????????????????????????????????????????????????????????????????????????????????????????????????????????????????????????????????????????????????????????????????????????????????????????????????????????????????????????????????????????????????????????????????????????????????????????????????????????????????????????0110101122000110112011200211102111101201211012012101101[12]1111111111111011010200002110011011[01]111111321211101?112101221111111110111001101111[01]100111110111111011111111111110010111222211101112?100212101100011101111110211110????????1111111?212111?11111?111112111111??1??1?11111?12111??1?????2?11???1?111?11???000011000000000?00?10001111010100011010012101100001000000100000200002100?0000?0000000000000110000100010000000000001100100000?100000100100000000001001000010000010?000100100010?000?0?0000000000?00001????????0010100010000000010000010000100?01101001000?0010?????100??1??1???000??0?0?0?03100000001000011001000001100010010001100001010?01001000000000?010001010000?000002100?001?00000000000000000?????????????????????????????????????????????????????????????????????????????????????????????????????????????????????????????????????????????????????????????????????????????????????????????????????????????????????????????????????????????????????????????????????????????????????????????????????????????????????????????????????????????????????????????????????

*Tyrannosaurus* 20013000000020100100001000010011112011002321001000200100101?0111220001322201001------000-010-0-210??10?0000000110101011100110-0000000100000101110110100020110011001111101110110?11110100-011?10011201110110103?0000100000010010110?0010000010010-0?010?010-?0-1000000011000-00000-00-1000010111?011100101010100001020000000000000000010100000111010-11?00000011?00010001000000000010000001001003100000?0100000100000020000000-100110???101??0001001010000001100010012?00010001001001?0?0000101000----03200021--00000?000100101011100000001200011000000101011000101000010000000102100101011012001210010001000100010111?0010?0??0?010002000110100?0011100001-100020000??????????????????????????????????????????????????????????????????????????????????????????????????????????????????????????????????????????????????????????????????????????????????????????????????????????????????????????????????????????????????????????????????????????????????????????????????????????????????????????????????????????????????????????????????????????????????????????????????????????????????????????????????????????????????????????????????????????????????????????????????????????????????????????????????????????????????????????????????????????????????????????????????????????????????????????????????????????????????????????????????????????????????????????????????????????????11101112220001101121012[01]0[13]01102111101201211112010??110121011111011[01]1101111021010211002101111111113[12]12111011113121230111111111111101101111111111111011100201111111111111112011122222110111211112121121111110111111102111101211111111111110212111011111111111211111111110121111111211111112111211111111111111211000011000000000010?10001111000100011010012101000001000000100000200002100?0000?00000010002001100001?0010000000000001100100000010000010010000000000100100001000001000001001000100000001000000?000?00001??1?????0010100010000000010000010000100?0110100100010010001001000?0??1???00000?0?0??03100000001000011001000001100010010001100001010001001000000000101000101000000000021000001?00000000000000000?10000?00?0000000210000110101000000000101201021100010001?00000000000001001000000101011010000000100101000000101?00000000??1????0000000010100??0?010001001000100000101101101000000000100001000100000200000001000000000000010001000000000001011000000000001011000000000000101000000?001110001000?0?00?0????01001001010110?0000000???000100000000000010000100000000100111

*Sinosauropteryx* 0?00??000?????????0?0??????1??1??????????????????????????????????????????2??001------??12?121?????000000000001?001??0?0?????0-0000????0?0????0000???????????100???00-0--?1100????11???????0??0-00??????00??00??????????0?????????????????0???????0?????????????????0001?000-00?0??0??0-0?0????????????????10100?1002101-0?1000000?000100-0??????010-1?1?0?10?10??11??0?20??100????????10?01???03????????000?0?000??1120?10000-1?1110????????00010?111010000200?00?00????01?0??00000?1100?0111101?1110?22??121110100010001000-1000???0???0?0?001020?000?111?????1???000100000011?100?0-?1??01?0001??0??0?1????0?01??10?00?0???0??0?00?2000110100?001100??00???00200??????????????????????????????????????????????????????????????????????????????????????????????????????????????????????????????????????????????????????????????????????????????????????????????????????????????????????????????????????????????????????????????????????????????????????????????????????????????????????????????????????????????????????????????????????????????????????????????????????????????????????????????????????????????????????????????????????????????????????????????????????????????????????????????????????????????????????????????????????????????????????????????????????????????????????????????????????????????????????????????????????????????????????????????????????????????????????????????????????????????????????????????????????????????????????????????????????????????????????????????????????????????????????????????????????????????????????????????????????????????????????????????????????????????????????????????????????????????????????????????????????????????????????????????00000?000000110000?10011011011???0000000000001?0?001000??00?1?1??0?0?????????????????????????????????000000??0?010?????1?0?????0001000000001000001?010?0??100?00?000001?0?11?0??00????000?0?002?1000000000?????1000?00000000000?0?00000001?0?10101000000?0000?00011000100100000001001100?00000??000????002?0000???00110?001000?100001001?0000??01?0??00?010001?10100000000000???010000000000?0000000?01?0??????????????0001???1??0?0?????0000??0???00????????????000?01?0?????0000101010?00??01??100????0?0?0????1??11200000100????00000?00?10??1010001000020??????00100?100?01?0??00?00?000?0?01000000000?011??000?000000???0???000000001101000100100001?0???0?0?0001101?0001000000000?0000010001000000100001001010?1?110000000000?10000??010000000001110000?0???0000?11

*Garudimimus* 002030000[01]110?000?010??????1??1??1000100??1??0001?20000000??02012100112?120??01------??12?121???????1000002?000001001?0101110-00000111000----0?0000000??01-011000010-0--111011101120?000-000?0-00?000-00?00003?01?01001000?11?0??000101000010?01100?11?????????0??00??-0001001??0-00-100?00?101?1000-0?1??11------1----------2------------000?-1?????????????0-???2??00001010001001???0000001?04010100?0??0000???00?0??0??0????????????????????????????????????????????????????????????????????????????????????????????0100111000100111001001001001000101001000101020??????????????????10101100011001000110010001011100000?0100?010002000110?0000011100000?101020000????????????????????????????????????????????????????????????????????????????????????????????????????????????????????????????????????????????????????????????????????????????????????????????????????????????????????????????????????????????????????????????????????????????????????????????????????????????????????????????????????????????????????????????????????????????????????????????????????????????????????????????????????????????????????????????????????????????????????????????????????????????????????????????????????????????????????????????????????????????????????????????????????????????????????????????????????????????????????????????????????????????????????????????????????????????????????????????????????????????????????????????????????????????????????????????????????????????????????????????????????????????????????????????????????????????????????????????????????????????????????????????????????????????????????????????????????????????????????????????????????????????????????????????100100000020012020?11001001001100000000100000110?101010000?011100000000??????010101?0000?00?1?1001???00010000000100010000000200011?????1?????1??????????10?11?000?0?10100010?0??01??0??00???0???????????????????????????1???????????1?0??????????1???????????????????????????????????????0010000000110000010000??0??0??????????????????10000010?0001000?010001010100?00000100000010000????00000??001?0110????01101????021001101001000??0020000000000100?0001??1??000000000002001?1?3???????01100???000??0?001??0????????????????????????????????????????10000001100??????????00?000000010000?0001000001??0000?01??110??11?0010????0??1100110120010010010100000?????11110????????????????????????????????????0????010110110010???00??????000?1000??000???1????000000000111

*Sinornithomimus* 0?21000??[01]110?000?000???????????????0?00??1???000???0?0?00??0201210?2????2???01------??12?121???????????????????????????????????????????????????????????????????????????????????????????????????????????????????????????????????????????????????????????????????????????????????????????????????????????????????????????????????????????????????????????????????????????????????????????????????????????????????????????????????????????????????????????????????????????????????????????????????????????????????????????????????????????????????????????????????????????????????????????????????????????????????????????????????????????????????????????????????????????????????????????????????????????????????????????????????????????????????????????????????????????????????????????????????????????????????????????????????????????????????????????????????????????????????????????????????????????????????????????????????????????????????????????????????????????????????????????????????????????????????????????????????????????????????????????????????????????????????????????????????????????????????????????????????????????????????????????????????????????????????????????????????????????????????????????????????????????????????????????????????????????????????????????????????????????????????????????????????????????????????????????????????????????????????????????????????????????????????????????????????????????????????????????????????????????????????????????????????????????????????????????????????????????????????????????????????????????????????????????????????????????????????????????????????????????????????????????????????????????????????????????????????100100000020012020?1?001001001??00000001000001111101010000101110?00000????????1?????0000???????????0?00010000000100010000000?00011?????1?????1?????????0101?11000?0110100????????1?10??000??000??000100?0??????00010010211011120000110010??0?00101311100?110?10000100001100020011000110010010000000110000010000010000000000100?1000010010000?1000?0100??010001110201???0002100100??0??000?000??00001?????????????????????????????????????????????????????????????????????????????????????????????????????????????????????????????????????????????????????????????????????????????????????????????????????????????????????????????????????????????????????????????????????????????????????????????????????????????????????????????????????????????????????????????????????

*Ornithomimus* ?0200?????????????????????????????0?0??[01]?00??10?2??100??????1400110???????????1------??12?121???????1?????210000???11???01????0?020?????????????00???011???1110000?0-0--????1111112?000000???0-?00?????010001??????????0?0?11??110???0??000?0?0??0?0???1110?1-10010?10-0??10?1010-00-10??01?1?1?111??0???011------1?-???-?-??2??????????????10-?010-?1??22100???102???00??0???01??1??0?00??0?00501?00???100?0??0???0??0?01?00-??0??0????????11111???01?010002?0?????2????0??0000??1?0?0001??0?1?????0?22??0??2?00012210???010100?10001100??????1010000?010??010????00010??10001010000-110?????00?1001?0?10?0???0??????00???0??0?0100??0001???00?????100?01??0?0?000?????????????????????????????????????????????????????????????????????????????????????????????????????????????????????????????????????????????????????????????????????????????????????????????????????????????????????????????????????????????????????????????????????????????????????????????????????????????????????????????????????????????????????????????????????????????????????????????????????????????????????????????????????????????????????????????????????????????????????????????????????????????????????????????????????????????????????????????????????????????????????????????????????????????????????????????????????????????????????????????????????????????????????????????????????????????????????????????????????????????????????????????????????????????????????????????????????????????????????????????????????????????????????????????????????????????????????????????????????????????????????????????????????????????????????????????????????????????????????????????????????????????????????????????100100000020012020?110010012?1100000?00100000111110101000010111000000000?10110?0101?00001?00???00????0001000000010?010100000??0011?????1?????1?????????0101??1000001101000101000010100?00010000?000010010??????3001111021100112000011001010000??01111100?11001000010001110002001100022001001000000011000001000001000000?000100110000100100000100010?0001010001110201???0002100?00??0001000000??00001?01110?110?101?101021001101?010021100000000000000000000??????00000000010??01?1?3??????001?0011000010000010100000000?001001?????011201020100000?101001000000110001100100000010000000110001000100000203?000011111101?11100?000??0??11011101200100100101?0?0??????1?1100?0011011010?01?0001110001100002200200001010?111?0???10000110000?000200000?0011?11100??0000100111

*Shuvuuia* 0121000000110000020000202??0000101202?22??0??10?2???100?00?1031012003122101?000001012000-?0-------??000000110000010101?101102100000?11000-???00000000011000-11001-?100--1220111011202000-10000-001000-0100000??01?120000002110010001001111?00100-0010-??110010111000100?001000001000-0-0000-0010011110??001????0??03201-212110202?10--10-0??10-??10-10100211110?11210?101?0???0???0??100??01?105020?1?0010012200110100200101????1110110???001000-0110000000230000101?1-002????110?11?10101111-01101--0221112---1101121201000-10000--011101012001111000?30---1--11-00000-0000----22000-010011112-200100101???0??1???101?001??????10111211011010000011000102-1000?0011????????????????????????????????????????????????????????????????????????????????????????????????????????????????????????????????????????????????????????????????????????????????????????????????????????????????????????????????????????????????????????????????????????????????????????????????????????????????????????????????????????????????????????????????????????????????????????????????????????????????????????????????????????????????????????????????????????????????????????????????????????????????????????????????????????????????????????????????????????????????????????????????????????????????????????????????????????????????????????????????????????????????????????????????????????????????????????????????????????????????????????????????????????????????????????????????????????????????????????????????????????????????????????????????????????????????????????????????????????????????????????????????????????????????????????????????????????????????????????????????????????????????????????????0001000000100110100100110?1001?00000000000000110?1011101?0001?11?000010101100001100?010?210001??0??100000001000010000?00000021000??0?0?0100220000???010?10111100100101?1?1??0?10?1?20100120?002??000100?110?0??000000102000000301210000002?1?112112?100??1??0?0001????????????0111001?0????00??20?10101001103001????201000000002?????211?1111000101?11102111011102001000002110?001000000000000000000?0110100000000?112010000?00??00?111??00001000010100010???1?11000100000002100?021??000??0111111011101?0??1?201??2012000100?1000?00020?03021??1122011000?1?1011?2000?00021032?0??1101001110112110000201000?1100001100000010001?1??0?100110??0010010010110?00??0?0???????001?00000??0???001?000??0?000110???0????1??111?000?0010011000?00002?001100012111101?0?0?0100?11

*Falcarius* 1???1000011020000?0110201002002011?00100211021201020000000??011112003142111100001100?0022010-10000??????????????????????0???????????010??001?0??????????????????????????????????????10000?0?00-00??0????????????????????????1?0?00?001?1000?010??0?011?110-1101100????1?00100000??00-1010?????????????????????????0?20??1[01]00101110001110-0???????1101?212100010?1121111201020011101001010?0010041101000?1000120010011000010???101111???1010?10010?1?101001?1001?0010001111001100?0001100100101000111102200021110010000001011110011101010100000011010??1110010101010001121023001022000-010?01101011000000100000101111100010?11??01000010001101001001100??00100???0010????????????????????????????????????????????????????????????????????????????????????????????????????????????????????????????????????????????????????????????????????????????????????????????????????????????????????????????????????????????????????????????????????????????????????????????????????????????????????????????????????????????????????????????????????????????????????????????????????????????????????????????????????????????????????????????????????????????????????????????????????????????????????????????????????????????????????????????????????????????????????????????????????????????????????????????????????????????????????????????????????????????????????????????????????????????????????????????????????????????????????????????????????????????????????????????????????????????????????????????????????????????????????????????????????????????????????????????????????????????????????????????????????????????????????????????????????????????????????????????????????????????????????????????????????????????????1???0??????0??0000???????11??????????????1???00????0101000?0?01?100020001?????????20000??001?????????????????0?0???0100010010110010???110000?101?0110010110000?110?001??002??0001????????00???000?0100?0?101010?100?11101?100100?00?1000000100100010000000000000000001010001001010100210000010000?011?02000110??1??1001101000000001001000?020000?000000000000??000000?0010?00100?01??01000??01?0121??????0???1????????????00??????00000???????0??000?????????00110000????10?01101?101?000?110011102?011?????????00?100011011?0000000100?11001010220001?10101000000010001001010000000?00000??0??10?00?0?0111111111????????????????????00?????0001?11???0?????11?01?0111000100010000000100021?101????0?0?0?00?000010???000000000000?0??0?0???????00????

*Erlikosaurus* 1010400001112?0001100020????00?1010?0??120132?001420000000?10211120?22421010000011001000-?0-------??100000100110000?-0?101110-00001101000????0?00????11????100000110-0--?1100--?110?1000010000-00100??0000000???1012100001---010???000?000000100-??00-????????10?0001010?011?1011010-101000-011?0000-0100011-?----0220??1?0013111?001010-???????????????????????????????????????????????????????????????????1??????????????????????????????????????????????10????????????????????????????????????????????????????????????????????????????????????????????????????????????????????????????????????????????????????????????????????????????????0???????0??0?????????0??????????????????????????????????????????????????????????????????????????????????????????????????????????????????????????????????????????????????????????????????????????????????????????????????????????????????????????????????????????????????????????????????????????????????????????????????????????????????????????????????????????????????????????????????????????????????????????????????????????????????????????????????????????????????????????????????????????????????????????????????????????????????????????????????????????????????????????????????????????????????????????????????????????????????????????????????????????????????????????????????????????????????????????????????????????????????????????????????????????????????????????????????????????????????????????????????????????????????????????????????????????????????????????????????????????????????????????????????????????????????????????????????????????????????????????????????????????????????????????????????????????????????????????????000000000001010200110110?0??110000000000000011011010100100011110000110????02?10?1?00100?00001110??1102200000101000000000000200001????00100010112000010??????????????????????????????????????????????????????????????????????001111?????????????????????????????????????????????????????????????????????????????????????????????????????????????????????????00?200?0211000????????000000000111100100?0110??02?1?1?0???010011100??10001?10000010001100000100??1111220010000002001?00100001?????????????????????????????????????????????????0???????????????????????????????????????????????????????0?00?021????00??0??00?0?000111?1?11?0001001?001001000010000000001?????????????0????????????????????????????????????0?0?001100001??????????0??111100???1?????0100???01??

*Avimimus* 101?100002-------00000101000100111??2??110110?021120110001?10??????0?122?11?011------100-00-------??????????1?1?????????????????????????????????????????????11??1????????????????????000?????0-?01??????0?0?0????????????1?1?0?110?0001?110?0?0??0?00-?110-???????0?00????11?????????11??10-0?0?00???????111-??-??????????2??2????-?--??????110?111010012000110?1121101001010001111000000000110601010?0?2?0000???00?????????????????????????2001001100001001100100101000100100000001?111????0???????0??2???????????????0100??1???100001111010001111?00?0100100011-00011110230010?0000-011011210110100000101000001001100011?1110001011200011011100?11000102-101020001?????????????????????????????????????????????????????????????????????????????????????????????????????????????????????????????????????????????????????????????????????????????????????????????????????????????????????????????????????????????????????????????????????????????????????????????????????????????????????????????????????????????????????????????????????????????????????????????????????????????????????????????????????????????????????????????????????????????????????????????????????????????????????????????????????????????????????????????????????????????????????????????????????????????????????????????????????????????????????????????????????????????????????????????????????????????????????????????????????????????????????????????????????????????????????????????????????????????????????????????????????????????????????????????????????????????????????????????????????????????????????????????????????????????????????????????????????????????????????????????????????????????????????????????????000?1??????????????????????1?0100???????110???1???1??00011??00?110??0??100001?00110?1000????????02???????0?110000?001???01100???????????1??????????10110100110?001100?0111?0??100????????????????????????????001?0?01?01100000?????10???12011???????0???????????????????????????????0?00???0010101?0210100010000?0?100?000110?????100111000000?0??101111101021????000210?100??0000100001??00000?????????????????????????????????????????????????????????????????????????????????????????????????????????????????????????????????????????????????????????????????????????????????????????????????????????????????????????????????????????????????????????????????????????????????????????????????????????????????????????????????????????????????????????????????????

Oviraptoridae 10[01]00010001000200000200000010001012020101011000001201010011102002[12]1011[02]01210001------000-0[01]0-[01]1000??1000000010100101?0?110100-00020111000----0000100101?001010000100-1101100101?10-111000100?0-001200-000?000?011011111101?0??010101001111000100-0?001??11111110000021-0201101111100-110-10-01010001100-0111------1----------2------------??10-?110-?????210010??1????1?0?1???????1??1000??0?1?52???0???10?????1???0??2?01?1????0??000111111200?????00?110010?1?????1?00?0???000??0?1?10?00???0?????0?22??0????0010000?????0-100????001?1??????0-01000?110??0110???20?11102300102?000-01??????2110010?0?10?????0????????????????0000??000????00??0???00?00??0?0?00??????????????????????????????????????????????????????????????????????????????????????????????????????????????????????????????????????????????????????????????????????????????????????????????????????????????????????????????????????????????????????????????????????????????????????????????????????????????????????????????????????????????????????????????????????????????????????????????????????????????????????????????????????????????????????????????????????????????????????????????????????????????????????????????????????????????????????????????????????????????????????????????????????????????????????????????????????????????????????????????????????????????????????????????????????????????????????????????????????????????????????????????????????????????????????????????????????????????????????????????????????????????????????????????????????????????????????????????????????????????????????????????????????????????????????????????????????????????????????????????????????????????????????????????110000001101110120?100110010011110011000132001101101111110000?110001110?1220010001??0110110001111000202101021000?1?000101110101101?????1?????1?????????11011010000?110?110?01?1101??201??????02???00?11?1011101013000?010?0010000001100110?01120010000001000000100000000100000000100000110010?1100?0101?02?011?000??0?0?10?2000110????11?1?11000100?00010?10010101000000001000?00?001000000010000000?011001001001??2210001111110001011?1001321000110000011000110221120?011001011?1?3??????1011001100101111001??20???002100111?011111?010000100?11000001110021100?110220?0??1010010?1101000000100?000001000000010010110?000000000??0???01010110101001110101001??????10????010?0010110100101000000000000000100??????1??101?00000001011001000001??00000100?10?01?1110?0?10??

*Buitreraptor* 1?210010011120000?010020?0?0000????00?0???1???001121000000??02112200??????????100000???0-?121??????????????0000??1?10???0?1???0??????????1???????0???????????00???0??????????????????110010??0-001?0????0??0??????????????????????????????????0???????????????????????1?0?0-?0????01010???????????????????????????0?201-0020??100?00??00-0??110?010-??201210110??12??010???????0??1????0???1?1??01??0???????1??01??10?2?01??????1??2????10??2?1????11??10111001??????????0??1??????1?110??????????????22???????????????0100??????????01?1??????111?111?1????010????201???2???0?1??0?1001???????1??1??????????????????????1????????11?20001????0?0011?10??1-10?1?01??????????????????????????????????????????????????????????????????????????????????????????????????????????????????????????????????????????????????????????????????????????????????????????????????????????????????????????????????????????????????????????????????????????????????????????????????????????????????????????????????????????????????????????????????????????????????????????????????????????????????????????????????????????????????????????????????????????????????????????????????????????????????????????????????????????????????????????????????????????????????????????????????????????????????????????????????????????????????????????????????????????????????????????????????????????????????????????????????????????????????????????????????????????????????????????????????????????????????????????????????????????????????????????????????????????????????????????????????????????????????????????????????????????????????????????????????????????????????????????????????????????????????????????????301?0???????????1??1?0110?101???000000?????00110?1010?00????1?1?0000?0?????????????????????0?????????000?00??0101????????0????????1????0000220?00???1?001010110010011011011011100??100?00121102??0011????????00000010?11010111000002100?101??1?00?????????????????????????0??????????????0???1???110111?0210?0?01???110012?210011?101111???1?010100000??0110?1010200?000110000?10?????0001???011???0?0110???????????????00001011??00??????????100?10???0?????????00??001?????????021??100?0010111100111?010???100011012?021???????01101111010?1?100?0??????11?101??122??1?111?110???11?0??0??1?011?011210000???????1??00?0????000????0???????1001001?0?01?0??????0?0?111?00?10011120????????????????????????0?101?1????1?0???0?2??????12011021??000?0??1??1?1?0??0?000??1

*Bambiraptor* 102100100111000003013020300000010023010?001000011010020000??0200?2000250200200000000?000-00-------??????????????????????????????????????????????????????????????????????????????????????????????????????????????????????????????????????????????????????????????????????????????????????????????????????????????????????????????????????????????????????????????????????????????????????????????????????????????????????????????????????????????????????????????????????????????????????????????????????????????????????????????????????????????????????????????????????????????????????????????????????????????????????????????????????????????????????????????????????????????????????????????????????????????????????????????????????????????????????????????????????????????????????????????????????????????????????????????????????????????????????????????????????????????????????????????????????????????????????????????????????????????????????????????????????????????????????????????????????????????????????????????????????????????????????????????????????????????????????????????????????????????????????????????????????????????????????????????????????????????????????????????????????????????????????????????????????????????????????????????????????????????????????????????????????????????????????????????????????????????????????????????????????????????????????????????????????????????????????????????????????????????????????????????????????????????????????????????????????????????????????????????????????????????????????????????????????????????????????????????????????????????????????????????????????????????????????????????????????????????????????????????000000000000?1?001?1001101131??00?00000101?00110?10101001???111???101?????????0??0?1????1??0?????????000001??00?10110????0???000000??0?00001000001101?0?11201100?00?001111001110?0??101001211?1100011???10?1100000010?11010111000002100?1010?1210101001010000001001000000000110000110001?0?10111?1101010021020001000110012?0000110?11111011??01010000000011001010100?0001100001100011001011010110010?0110?????0?0?1??1??00?0?01110000??10?01??10?1?????0?????????00??010??1??000?01011100?????0?120011??10??0???0011??110?????0011001011110?001?1010000010?21?001110020001?1021100?1?110000??10010001101?0?000?00?01??0000????000?0000000?1013001??100101?10?011100011011000?00111201011010001000001011001010201101101?100001001100?001200101100000100001011110???000011?

*Tsaagan* 20200010011100000201302030001100002301011[14]1000001010020000?00200220002502011000000101001000-------??00000020000?011010?100110-000000010001???00000100?1?000100010110-0--1100?????0-111001100?0-001200-00001?03????0?000000100101010001110101110???0000??11110-100000001?000-00000-00-0-0?010011101011111?010101?0002101?0?1000000?000100-??????????????00?0?????110??????????????????????????????????????????????????????????????????????????????????????1??????????????????????????????????????????????????????????????????????????????????????????????????????????????????????????????????????????????????????????????????????????????????????????????????????????????????????????????????????????????????????????????????????????????????????????????????????????????????????????????????????????????????????????????????????????????????????????????????????????????????????????????????????????????????????????????????????????????????????????????????????????????????????????????????????????????????????????????????????????????????????????????????????????????????????????????????????????????????????????????????????????????????????????????????????????????????????????????????????????????????????????????????????????????????????????????????????????????????????????????????????????????????????????????????????????????????????????????????????????????????????????????????????????????????????????????????????????????????????????????????????????????????????????????????????????????????????????????????????????????????????????????????????????????????????????????????????????????????????????????????????????????????????????????????????????????????????????????????????000000000020012010?1000111130110000?0001012001101201010010011?1111101000120001000001100110100???01???0000010?0001011000000?11100000?10?00001000001?0100011201100??0100?????????????????001212?11000??11111?????0000??1??0101110?0?010????????????1??0?????????????????????????000????????????????????????????00010??1?0????????????????1?11100101??000???11101?101000?00??0000??0?0????101?010010000?011001001000012011200001010100001?100012110110001?00001?0???00000100?1111001010101000????0??2?????????????????????????????????????????????????????????????????????????????????????????????????????????????0??0??00?0?0000???0?0??00010013?0001000001?1???0?0?0?????????????????????????????????????????????????????????????????????????????????0???1?????0???????0??

*Dromaeosaurus* 1020001001110000030110?0??010100002001011011000210200000?0100201220021501002000010011000-00-------????????2?0??1??1?1??100????0???0?010??10100??0????011???100??011??????1???????0-121111???????0??0????????031???0?0??000100001000000??0011?11010000-1110-10-1000000011000-00000-00-0-000100111010111101010?000110?000?00000000000?0100-0???????????0????????????????????????????????????0?????????????????????????????????????????????????????????????????????????????????????????????????????????????????????????????????????????????1????????????????????????????????????????????????????????????????????????????????????????????????????????????????????????1????????????????????????????????????????????????????????????????????????????????????????????????????????????????????????????????????????????????????????????????????????????????????????????????????????????????????????????????????????????????????????????????????????????????????????????????????????????????????????????????????????????????????????????????????????????????????????????????????????????????????????????????????????????????????????????????????????????????????????????????????????????????????????????????????????????????????????????????????????????????????????????????????????????????????????????????????????????????????????????????????????????????????????????????????????????????????????????????????????????????????????????????????????????????????????????????????????????????????????????????????????????????????????????????????????????????????????????????????????????????????????????????????????????????????????????????????????????????????????????????????????????????????????????????00?00000?0???001??0??11??????0????00?????00??0120101001?0111111110??00100000000000000110?100?0010??000?000000010110?00001111000002?000000000000100100?????????????????????????????????????????????????????????????????????????????????????????0??0?0????????????????????????????????????????????????????????????????????????????????????????????????????????????????0011??????????110101?0?????????0??001000000000010??0?0?0???0??01110????1111????1?1001100???000001001111100?000101000?????????????????????????????????????????????????????????????????????????????????????????????????????????????1??????00?????00?0?0000???0?00?000?00????0??00?0010010100000??????????????????????????????????????????????????????????0111???????????????????0??01?????000????00??

Troodontidae 1020001?0[01]110?00030110101001000101200?00?01?00000110010000?10[12]10?2?03[12]2210?1?01------000-0[01]210--00??0???001?0?0?010?011100?10-0000?1??0??1??????00???0????????00???110--????100??0-?000001??00-?01??0-0???00?30??1?11??0?0011000??00101?1000001???010-1110-10-10111?1?1??010?100??0110-00????????0????????101?00?00?201?0?1000220?001010-????111?10-111??210110?01?10?100101??????1??1011??0?10501?10?0????011?0?0?20?0??1?????????2??????????????1?00110???0?1?0?10??01?0????????0????000010?0???????220??????001?????????0-??????????????????????????010??0001???0??11??23??1?21000-0?0001?11120110?0?1???0?00110101??1????1?0100012000110?00?????100111??011?11???????????????????????????????????????????????????????????????????????????????????????????????????????????????????????????????????????????????????????????????????????????????????????????????????????????????????????????????????????????????????????????????????????????????????????????????????????????????????????????????????????????????????????????????????????????????????????????????????????????????????????????????????????????????????????????????????????????????????????????????????????????????????????????????????????????????????????????????????????????????????????????????????????????????????????????????????????????????????????????????????????????????????????????????????????????????????????????????????????????????????????????????????????????????????????????????????????????????????????????????????????????????????????????????????????????????????????????????????????????????????????????????????????????????????????????????????????????????????????????????????????????????????????????????????1????????10?21?002111110??00???0?01022???1??????????0?0?1100000211110012?101010010000010??????0??10000??010??????????????????00?0?0300110001011001???11?1101001?011001?111111?100??012??12??0?1??????????????0???????????0?????100?10????2?01?0?0????????????????????????????????????????????1?????????10???????????????0??????????01?1?0??10??????01?01?1111001?01012100111?001011111010020000???1?1112?1101000001???0?010100??????20220000210?001100????0?10??001?????????011010100???1111100101101111?1000?1020??11???????????????010????0??????????1????????????????1??0?011?100??????0100001210?01????0?0???0??01000???0?30??????0?1????????10?000???1?10??111?????????1?01?0???????????????????????????????11?101011110??????0?101??0??000??0?????10???01?????

;

ccode + 0 2 3 10 17 19 22 24 28 61 65 68 73;

proc/;

## Morphometric analysis on the mandibular articulation in non-avian theropods (MorphoJ file)

The file is downloadable at

<https://drive.google.com/file/d/0B_-0b-kZatHiVF82UjY5OFFWTUU/view?usp=sharing>

and can be obtained by request to the corresponding author.

<?xml version="1.0" encoding="UTF-8"?>

<morphojroot version="1.02b">

<morphojproject>

<morphojproject_contents visiblename="Mandibular_articulation">

<uniquenumber>264</uniquenumber>

</morphojproject_contents>

<morphojset>

<morphojset_contents visiblename="Mandibular_articulation" uniqueid="1" alignmentmethod="0" dimension="2" hasprocrustesfit="true" hasrawdata="true" linkedoutlinenumber="0" linkedwireframenumber="42" nincl="37" nlmk="10" nobs="37" objsymmetry="false" procsumsquare="2.71703376844889" procrustesfitfromparentset="false">

<alignmentvectors>

<vector>0.0 </vector>

<vector>0.0 </vector>

<vector>0.0 </vector>

<vector>0.0 </vector>

<vector>0.0 </vector>

<vector>0.0 </vector>

<vector>0.0 </vector>

<vector>0.0 </vector>

<vector>0.0 </vector>

<vector>0.0 </vector>

</alignmentvectors>

<datavector>

<datamatrix visiblename="Mandibular_articulation, raw data" matrixtype="0" iscoordinates="true" issymmetrycomponent="false" isasymmetrycomponent="false">

<data>

<vector>701.0 312.0 588.0 325.0 324.0 270.0 108.0 138.0 368.0 134.0 466.0 143.0 573.0 67.0 849.0 201.0 840.0 306.0 707.0 282.0 </vector>

<vector>597.0 412.0 530.0 424.0 320.0 406.0 62.0 136.0 369.0 175.0 331.0 142.0 569.0 96.0 782.0 243.0 839.0 466.0 690.0 380.0 </vector>

<vector>558.0 416.0 519.0 397.0 145.0 295.0 57.0 161.0 229.0 177.0 407.0 95.0 479.0 53.0 731.0 159.0 794.0 521.0 528.0 271.0 </vector>

<vector>634.0 411.0 507.0 437.0 131.0 314.0 24.0 157.0 259.0 98.0 425.0 190.0 454.0 61.0 790.0 172.0 855.0 414.0 616.0 320.0 </vector>

<vector>492.0 515.0 442.0 517.0 240.0 436.0 122.0 229.0 379.0 265.0 356.0 100.0 493.0 86.0 774.0 296.0 759.0 621.0 585.0 427.0 </vector>

<vector>589.0 488.0 544.0 440.0 238.0 336.0 100.0 146.0 368.0 173.0 360.0 131.0 581.0 106.0 809.0 278.0 783.0 556.0 641.0 353.0 </vector>

<vector>594.0 389.0 501.0 389.0 164.0 257.0 72.0 311.0 163.0 147.0 511.0 200.0 617.0 182.0 785.0 232.0 850.0 386.0 726.0 329.0 </vector>

<vector>663.0 425.0 642.0 421.0 284.0 331.0 16.0 181.0 136.0 140.0 531.0 243.0 651.0 114.0 828.0 248.0 813.0 418.0 586.0 203.0 </vector>

<vector>673.0 519.0 550.0 558.0 268.0 393.0 105.0 56.0 394.0 236.0 472.0 190.0 544.0 151.0 722.0 284.0 762.0 482.0 571.0 398.0 </vector>

<vector>434.0 419.0 405.0 494.0 133.0 482.0 88.0 230.0 330.0 247.0 156.0 171.0 268.0 66.0 705.0 147.0 808.0 510.0 459.0 381.0 </vector>

<vector>684.0 501.0 610.0 501.0 302.0 404.0 131.0 205.0 392.0 235.0 297.0 173.0 521.0 87.0 820.0 280.0 784.0 518.0 625.0 354.0 </vector>

<vector>568.0 494.0 432.0 483.0 222.0 341.0 157.0 131.0 372.0 161.0 455.0 125.0 562.0 128.0 820.0 349.0 760.0 497.0 573.0 380.0 </vector>

<vector>693.0 502.0 616.0 481.0 135.0 210.0 40.0 298.0 199.0 146.0 420.0 150.0 565.0 122.0 811.0 319.0 806.0 478.0 661.0 372.0 </vector>

<vector>509.0 375.0 420.0 379.0 252.0 350.0 15.0 270.0 151.0 136.0 373.0 151.0 507.0 91.0 800.0 211.0 774.0 447.0 628.0 387.0 </vector>

<vector>552.0 522.0 486.0 521.0 282.0 402.0 34.0 178.0 249.0 168.0 428.0 164.0 566.0 101.0 818.0 321.0 779.0 551.0 642.0 381.0 </vector>

<vector>485.0 449.0 466.0 451.0 197.0 346.0 46.0 137.0 285.0 199.0 380.0 119.0 486.0 99.0 746.0 259.0 808.0 555.0 589.0 406.0 </vector>

<vector>664.0 379.0 610.0 346.0 193.0 178.0 49.0 240.0 167.0 83.0 362.0 90.0 553.0 49.0 825.0 257.0 824.0 445.0 675.0 290.0 </vector>

<vector>473.0 429.0 346.0 482.0 129.0 407.0 68.0 225.0 252.0 271.0 370.0 227.0 655.0 137.0 860.0 210.0 803.0 393.0 624.0 333.0 </vector>

<vector>610.0 379.0 426.0 389.0 184.0 284.0 58.0 477.0 339.0 138.0 511.0 161.0 634.0 120.0 795.0 182.0 816.0 351.0 647.0 307.0 </vector>

<vector>546.0 342.0 515.0 346.0 213.0 295.0 91.0 237.0 241.0 152.0 398.0 137.0 438.0 119.0 759.0 215.0 842.0 367.0 617.0 306.0 </vector>

<vector>652.0 511.0 600.0 541.0 284.0 451.0 42.0 308.0 364.0 291.0 283.0 263.0 650.0 227.0 811.0 382.0 835.0 531.0 673.0 430.0 </vector>

<vector>605.0 376.0 552.0 381.0 357.0 323.0 126.0 127.0 411.0 169.0 382.0 105.0 527.0 46.0 817.0 228.0 791.0 441.0 660.0 305.0 </vector>

<vector>530.0 504.0 423.0 483.0 195.0 426.0 39.0 303.0 319.0 235.0 183.0 161.0 420.0 106.0 758.0 254.0 755.0 603.0 546.0 391.0 </vector>

<vector>437.0 479.0 397.0 481.0 176.0 391.0 195.0 192.0 386.0 260.0 299.0 159.0 469.0 86.0 773.0 195.0 773.0 472.0 460.0 332.0 </vector>

<vector>490.0 385.0 456.0 393.0 174.0 368.0 98.0 142.0 428.0 114.0 391.0 66.0 454.0 41.0 765.0 254.0 721.0 566.0 547.0 457.0 </vector>

<vector>449.0 427.0 355.0 480.0 92.0 317.0 98.0 130.0 358.0 190.0 316.0 96.0 446.0 77.0 733.0 218.0 714.0 604.0 506.0 441.0 </vector>

<vector>468.0 503.0 368.0 510.0 158.0 325.0 77.0 31.0 315.0 203.0 418.0 163.0 497.0 97.0 753.0 270.0 816.0 520.0 590.0 360.0 </vector>

<vector>587.0 449.0 448.0 446.0 202.0 387.0 71.0 250.0 257.0 204.0 363.0 197.0 479.0 139.0 770.0 258.0 830.0 541.0 612.0 363.0 </vector>

<vector>479.0 374.0 374.0 379.0 223.0 393.0 44.0 270.0 245.0 201.0 409.0 225.0 445.0 283.0 629.0 129.0 880.0 323.0 657.0 403.0 </vector>

<vector>622.0 285.0 574.0 289.0 212.0 263.0 23.0 280.0 74.0 161.0 339.0 84.0 391.0 61.0 750.0 104.0 788.0 258.0 597.0 203.0 </vector>

<vector>437.0 347.0 400.0 280.0 260.0 380.0 118.0 310.0 227.0 231.0 397.0 242.0 429.0 307.0 613.0 214.0 754.0 293.0 602.0 343.0 </vector>

<vector>756.0 415.0 701.0 423.0 314.0 322.0 30.0 302.0 205.0 156.0 434.0 156.0 538.0 111.0 756.0 177.0 840.0 387.0 654.0 289.0 </vector>

<vector>775.0 440.0 723.0 432.0 345.0 338.0 12.0 197.0 351.0 212.0 509.0 193.0 575.0 151.0 767.0 223.0 838.0 384.0 683.0 294.0 </vector>

<vector>610.0 401.0 576.0 418.0 206.0 324.0 53.0 256.0 273.0 185.0 289.0 178.0 489.0 116.0 733.0 147.0 829.0 338.0 562.0 275.0 </vector>

<vector>572.0 417.0 524.0 419.0 139.0 262.0 49.0 179.0 374.0 151.0 441.0 135.0 520.0 141.0 765.0 260.0 806.0 532.0 599.0 388.0 </vector>

<vector>592.0 341.0 568.0 349.0 144.0 235.0 39.0 381.0 57.0 168.0 423.0 135.0 480.0 116.0 716.0 223.0 833.0 436.0 607.0 311.0 </vector>

<vector>434.0 312.0 364.0 323.0 181.0 263.0 85.0 117.0 297.0 95.0 327.0 85.0 613.0 53.0 817.0 232.0 744.0 367.0 603.0 254.0 </vector>

</data>

</datamatrix>

<datamatrix visiblename="Mandibular_articulation, centroid size" matrixtype="1" iscoordinates="false" issymmetrycomponent="false" isasymmetrycomponent="false">

<data>

<vector>765.8237395119062 6.640952038186516 </vector>

<vector>835.9491013213664 6.7285677276443385 </vector>

<vector>853.0912026272455 6.748866461620527 </vector>

<vector>913.3646040875462 6.817135148179956 </vector>

<vector>827.4993655586692 6.7184083405544515 </vector>

<vector>841.7993822758484 6.735541722522764 </vector>

<vector>872.8720983053588 6.771789036864937 </vector>

<vector>906.5618566871209 6.80965926469768 </vector>

<vector>797.3662897313881 6.681314158823368 </vector>

<vector>863.7132047155468 6.76124077471348 </vector>

<vector>825.2938870487288 6.715739549626737 </vector>

<vector>793.0030264759397 6.67582703811677 </vector>

<vector>953.379252973338 6.860012781440324 </vector>

<vector>855.0245610507338 6.751130194899293 </vector>

<vector>892.1599071915304 6.793645384665334 </vector>

<vector>855.666757563948 6.751880998432805 </vector>

<vector>934.1657775791189 6.839653914533723 </vector>

<vector>886.7921966278233 6.7876106782309815 </vector>

<vector>837.278687176498 6.730156974660336 </vector>

<vector>772.610121600798 6.649774550846339 </vector>

<vector>857.0466148349225 6.753492310158115 </vector>

<vector>751.5893160496629 6.622190052496371 </vector>

<vector>865.5460704087333 6.7633606030190565 </vector>

<vector>755.4525795839207 6.627317012812253 </vector>

<vector>826.166327079481 6.716796117752871 </vector>

<vector>849.615265870382 6.744783618629427 </vector>

<vector>882.4135085094741 6.782660776649713 </vector>

<vector>835.9876195255525 6.728613803795083 </vector>

<vector>776.2090568912475 6.65442188711106 </vector>

<vector>858.8722838699593 6.755620230918598 </vector>

<vector>602.8600169193509 6.401685025365147 </vector>

<vector>880.8525415754897 6.78089023573595 </vector>

<vector>838.2636816658586 6.731332706914134 </vector>

<vector>808.2088838907922 6.694820544777467 </vector>

<vector>841.9698925733628 6.735744256564267 </vector>

<vector>896.250746164264 6.798220224453806 </vector>

<vector>798.7211027636617 6.683011826963639 </vector>

</data>

<namesvector>Centroid Size Log Centroid Size</namesvector>

</datamatrix>

<datamatrix visiblename="Mandibular_articulation, Procrustes coordinates" matrixtype="2" iscoordinates="true" issymmetrycomponent="false" isasymmetrycomponent="false">

<data>

<vector>0.1951871472998794 0.12607836341490491 0.0472370286147979 0.14311341066244065 -0.303196114638409 0.06965502746297707 -0.5870395012781343 -0.10465359851781644 -0.24436855054372322 -0.11164869709192189 -0.11492365578696051 -0.10100094462217933 0.027545330360365952 -0.20061793442359366 0.392593373801903 -0.024441433536841767 0.38283364245509915 0.11910195785206425 0.20413129971518218 0.08441384879996619 </vector>

<vector>0.12025154283854465 0.1432068696626537 0.03950999293175189 0.1639901696400206 -0.2224426579369091 0.15905410848086765 -0.5575032207034619 -0.1379290892498785 -0.183999852027815 -0.12401347628698843 -0.22668069726936227 -0.1601898940603652 0.05342679835613007 -0.2390135074609791 0.3299639656203274 -0.08224889103192415 0.4200488618129742 0.18433590064206296 0.22742526637782048 0.09280780966453041 </vector>

<vector>0.15138456016511787 0.17935572331885047 0.10153555235147863 0.16197830218476506 -0.35068355934247636 0.08024540688984783 -0.47265528672689244 -0.06976972878768413 -0.263855809866341 -0.0713188747332409 -0.06418730684905206 -0.18466611880757558 0.019866581986814316 -0.24247980697439453 0.33173797314632497 -0.14223772925305483 0.4432793752037886 0.2762968530628688 0.1035779199312379 0.012595973099617632 </vector>

<vector>0.18595102486754467 0.16506216576062677 0.04730690625210692 0.19733764440997958 -0.372008726560252 0.07376671775600134 -0.4977108841967188 -0.09643268944143843 -0.2385189815176675 -0.16899182209527827 -0.05345077695410588 -0.07557179169870719 -0.02188713629884726 -0.2174113805342009 0.35316946280980716 -0.10455952498206003 0.4323858688406318 0.16210882967460508 0.16476324275750073 0.06469185115047234 </vector>

<vector>0.07961190310875545 0.1946611951410154 0.01659079935426917 0.2091486590222286 -0.2588393617370827 0.15634129033472005 -0.45554789752452923 -0.06380574441778887 -0.13400529398672026 -0.08596329413601793 -0.18959294809537036 -0.27102823629640976 -0.024762961650608022 -0.32292387799583094 0.36826347200775145 -0.13830360527786725 0.42979857901118534 0.2562502954072815 0.1684837095123492 0.0656233182186693 </vector>

<vector>0.14931869404096232 0.19713153354521484 0.08414496560463586 0.15309295912357376 -0.3002210656785855 0.10663707388894059 -0.5093152831205645 -0.081069908152743 -0.1891223063390893 -0.1167236328530485 -0.20652168723447797 -0.16366567943265237 0.044713656450276335 -0.24770905672720106 0.3554558038893529 -0.10318023265217517 0.39468254258985996 0.22835107275359254 0.17686467979762963 0.027135870506498447 </vector>

<vector>0.11871574623637687 0.13337317969718085 0.008504357715085753 0.13471576400995192 -0.3959078557128905 -0.017253853252735124 -0.5177746122440644 0.0241481765436372 -0.3888377971486907 -0.1597186189068883 -6.139492552643448E-4 -0.1065025682311643 0.13179068005482056 -0.13315228854560324 0.35078753590373485 -0.06507592388036093 0.4278043797101948 0.13242332291350808 0.26553151474069725 0.057042809652473964 </vector>

<vector>0.17381182569238648 0.17488646761432658 0.1402424068617573 0.17273731321626723 -0.27783389648184964 0.07696934528976164 -0.5834590306506943 -0.09074450303200052 -0.42399200716247115 -0.1438842098972589 -0.001469519068262477 -0.05204283392853763 0.14020228935002366 -0.19562703188746092 0.37033728832618207 -0.04522134698215293 0.36713127357901043 0.1673533521724377 0.09502936955391761 -0.06442655256538193 </vector>

<vector>0.25732639176397276 0.19591537890831404 0.1159729733683741 0.2742824321641643 -0.29015860542150124 0.14723535275185437 -0.5871425761212826 -0.2165615360767945 -0.17742535120112493 -0.08878752663778497 -0.08764490157841434 -0.16548977693961103 0.001682804626048659 -0.23572506577758506 0.2760991824655091 -0.11499033018841405 0.37995129386138593 0.13214522612726667 0.11133878823703193 0.0719758456685905 </vector>

<vector>0.0707937930523469 0.13966990465685183 0.02083970000199982 0.218754734084055 -0.3214329862120867 0.17074522052394647 -0.3790533782258588 -0.12358363447143035 -0.08182415454897712 -0.09501593006348018 -0.2441390060704704 -0.19438082594223532 -0.09259166735568403 -0.3064758798709113 0.41586409321232537 -0.16609140468242808 0.5051103656336863 0.27018426510242977 0.10643324051271832 0.08619355066320211 </vector>

<vector>0.2158236391473198 0.205222192324827 0.12335043570915546 0.2106236612516283 -0.26733663284669595 0.1105706610375029 -0.49346868528783344 -0.12136123471140753 -0.16781673029787492 -0.10704505340679103 -0.2745973748260007 -0.17548805407605725 -0.007472042832000213 -0.2943028054129374 0.3769088568965917 -0.07903898246114267 0.35404555983641056 0.22043785525659867 0.1405629745009271 0.030381760197778922 </vector>

<vector>0.16446862977401797 0.19434969409519628 -0.00303785181087767 0.23374999673589814 -0.3158296032090202 0.14378130884169754 -0.47876534738330484 -0.0838727879181677 -0.20534388022567643 -0.13245355023804073 -0.117838230274857 -0.20767399793454983 0.014312875962880492 -0.24686353062968394 0.4134241240118624 -0.08079566050504278 0.4008044641971498 0.12498206818854998 0.1278048189578251 0.054796459364143346 </vector>

<vector>0.22732400478855957 0.18940028180051063 0.1412626387792109 0.1762414490106687 -0.3941344796303907 -0.05846872635000784 -0.4973647523327667 0.026299225313590212 -0.32996560013519455 -0.14530501500740986 -0.09919174522220843 -0.1638827038932076 0.05632327047087244 -0.21017834194671614 0.3474564275158328 -0.025091713076904245 0.3616839743222371 0.15695889911332572 0.18660626144384748 0.05402664503615016 </vector>

<vector>0.08417968681133416 0.11534627842874048 -0.021552774435733474 0.12233657384135552 -0.2317350400767907 0.08836347336946773 -0.5136113911426816 -0.005307729275154925 -0.3476678910416658 -0.1639281117583537 -0.0887191396308396 -0.1530917675315696 0.07074734720374984 -0.22746741552228317 0.42396664534636785 -0.0905704674128727 0.40235220797322574 0.19292618120849916 0.22204034899303388 0.12139298465217133 </vector>

<vector>0.11688399612421783 0.19808278567047646 0.04261568661056578 0.2107230781608993 -0.21293644334271758 0.12056044830772028 -0.5334684771237911 -0.07637676808785611 -0.29451275606804395 -0.13265378197716354 -0.09777564487622942 -0.17426802866270322 0.04364044657980178 -0.27251973655747713 0.3712364676874173 -0.08135394444704544 0.3772757660411042 0.1839916776823782 0.18704095836767445 0.023814269910771853 </vector>

<vector>0.07599747875400609 0.16200198065877802 0.05330111322650596 0.16865637701772843 -0.28207304378124337 0.10709138805667605 -0.5034506519731277 -0.09985576223413806 -0.21317751119455258 -0.08318723695714189 -0.1208423601131344 -0.1956599687516383 -0.00192093601908374 -0.24290395146326696 0.3349030598999642 -0.11614608110550699 0.47179952131961483 0.21162865722504434 0.18546332988105063 0.08837459755346531 </vector>

<vector>0.19491470021350804 0.14891521972819918 0.13122614115532313 0.11880185000862255 -0.33349634393115984 -0.03614364493511184 -0.49227794171721956 0.02573439294605307 -0.36073585947902204 -0.1472054547857194 -0.1514816594867031 -0.15351356850942613 0.05412751391820709 -0.21132518228014002 0.3699058283283917 -0.004006483861092799 0.38399783376790786 0.20922549813546398 0.20381978723076705 0.049517373553151715 </vector>

<vector>0.03728187901853817 0.14262674081092783 -0.1047990214649886 0.20608225098636776 -0.3799751366640846 0.12670923483146115 -0.47667550502271705 -0.08052629430588625 -0.24773265906381223 -0.04768337896136571 -0.11181339959546585 -0.1034331736165926 0.20420222044682662 -0.21970636057119325 0.4635145515630765 -0.13746883291025302 0.4188116822807115 0.09139875294472609 0.1971853885019156 0.022001060791808285 </vector>

<vector>0.1278667513525309 0.14731639372091784 -0.08750824369075327 0.13898466345834637 -0.39665054004146094 -0.017137864267937536 -0.5879299089302238 0.15437472211893755 -0.19959649937419283 -0.19219855369336772 0.0030290492185790618 -0.15244615086917093 0.1672090465035979 -0.19310946883331845 0.3888787452387072 -0.08688428005798494 0.4019732141862704 0.1440990048279648 0.1827283855369447 0.05700153359561304 </vector>

<vector>0.10056653569739009 0.1241730946793254 0.05845326013329342 0.1278063190066643 -0.3335226656125167 0.04329130423647648 -0.4912482527935145 -0.041612898066041994 -0.28775439183828944 -0.1431747744479987 -0.08362527189605255 -0.1542418108642573 -0.027296814126713736 -0.17661597152034322 0.38566586638136485 -0.032612494536435555 0.48452558500757903 0.1734241376980838 0.19423614904745937 0.07956309381452677 </vector>

<vector>0.16223097517705942 0.13742916909319547 0.09944637445059534 0.1739171917157656 -0.2822760007384342 0.07855687847056532 -0.5730605856750948 -0.08302603186269591 -0.19338928113069934 -0.11762577180826689 -0.2796474697429457 -0.14805155550561386 0.14368584284744934 -0.20595035545037788 0.35082396465559035 -0.029102406443939476 0.38687561722946684 0.15468479519225634 0.18531056292701295 0.03916808659911146 </vector>

<vector>0.12938696535749336 0.15802690199773298 0.057993351226850495 0.17231835667242634 -0.21679480300260429 0.1208984789148095 -0.5515137201883616 -0.10683938882101685 -0.16544439548874276 -0.0944578812606924 -0.207712599600125 -0.17457735721556966 -0.0217441477407012 -0.27375450504519633 0.3926092153893656 -0.07329167341248537 0.3908731535165467 0.21728294459888145 0.19234698053027927 0.054394123571110516 </vector>

<vector>0.1427886125259803 0.18097500753132986 0.01663182208561174 0.16337072289593937 -0.2644597724441503 0.10464588146824066 -0.45773221625757665 -0.03516199104132145 -0.13037489932923432 -0.12833628470023195 -0.2778717169040456 -0.2063616203874117 -0.005548086118430457 -0.2831500391991691 0.40069713714381816 -0.1256792555669142 0.41792558736360014 0.28222372497449544 0.15794353193442695 0.04747385402504267 </vector>

<vector>0.04340281677073364 0.23519528414466107 -0.01735514718277725 0.24457874075301 -0.34955316652204477 0.15249424761185293 -0.3817222510207044 -0.11385173615006504 -0.10104191151423302 -0.06547251385376839 -0.20517681155577847 -0.18094493825111407 0.01457070119355935 -0.3019655407942967 0.4469045150748752 -0.1927549709988079 0.49301976647564105 0.18405673268079265 0.05695148828072882 0.03866469485773572 </vector>

<vector>0.08759206863232466 0.12057235972866581 0.044626578160002796 0.1412046901299123 -0.3084037490116289 0.19519849674643128 -0.48248831484424065 -0.04497187469208005 -0.10053554697164727 -0.18636667536497123 -0.14692335362067865 -0.23056589178842918 -0.07073327742391611 -0.2839001575603182 0.3719743077497281 -0.13493815233539225 0.4275422973841876 0.2502671317111621 0.17734898994586862 0.17350007342501933 </vector>

<vector>0.0982335645908732 0.13776350778427782 0.007387499308474579 0.2269477903355617 -0.3576999524774965 0.13029884700723782 -0.42745637203606873 -0.08539459253590996 -0.10576283205311407 -0.11037049526932903 -0.17401627812816717 -0.2006350563408445 -0.027645930452529197 -0.268607172139774 0.3542906650686059 -0.2009442319651443 0.465106594440861 0.24413276285905022 0.16756304173856143 0.12680864026487423 </vector>

<vector>0.09442007647076782 0.21829539074743645 -0.013780707663920684 0.2574989421965418 -0.31271184632872595 0.12271624328769233 -0.5008647848954842 -0.17086050096378183 -0.18089097069533555 -0.06650600675476742 -0.07849147421606464 -0.14257445112458303 -0.007094123305801926 -0.24036127654716496 0.33534126202510534 -0.1306353921922373 0.48318902640525613 0.12881411862398343 0.1808835422042035 0.02361293272688058 </vector>

<vector>0.15809643119546737 0.14306627620514567 -0.008172303658612951 0.14845476135434252 -0.3081798916487507 0.09299220539276788 -0.4747546260953984 -0.06310459658897712 -0.25364700907936283 -0.13023978102014555 -0.12691748728533525 -0.14562935459051324 0.008987236428648255 -0.22259390777030805 0.36594974725376833 -0.09830052665277746 0.4558583896359029 0.2371621364463524 0.1827795132536738 0.03819278722411354 </vector>

<vector>0.05373607934244909 0.12103278237271378 -0.08346517575071019 0.10895632335381138 -0.317258145998264 0.08380035850450414 -0.5344912277938972 -0.11632251849773695 -0.24467408740663418 -0.16891062551143582 -0.03989409259666207 -0.11478867128064976 0.015618836846831923 -0.04949829589807812 0.31016447875810854 -0.1766331953553978 0.5772121149133086 0.13940570435650748 0.26305121968546885 0.17295813795576187 </vector>

<vector>0.18452827789938178 0.1578442081131409 0.12580263945163941 0.14877321497520082 -0.28272369152520255 0.00903933062461452 -0.5057523741244405 -0.03676325504434809 -0.39890332745081597 -0.1551588782239383 -0.08070251983357296 -0.1632708212016624 -0.007721617701859853 -0.17684133862130177 0.39098171083180355 -0.017190155790538612 0.39081080742466445 0.178461151468841 0.18368009502840232 0.05510654369999177 </vector>

<vector>0.026751225258850236 0.10967594482783842 -0.02177861433850373 0.0011797851828254354 -0.3228036714517068 0.08807600028113069 -0.5448209635059172 -0.08648907881465104 -0.3110551674755309 -0.17867884607236129 -0.04458587305389383 -0.11372740959736538 0.009971959160801347 -0.01888920451666681 0.3688845682094308 -0.07483930872522432 0.5615893500912009 0.13517417949502733 0.27784718710526946 0.13851793793944717 </vector>

<vector>0.23777130427833806 0.20375958855342538 0.17064769194926735 0.20385552247477504 -0.25636079075605106 0.02275397977209667 -0.5742784738088463 -0.05788718630520164 -0.34057505703045365 -0.19029897509135862 -0.08509115041100752 -0.15372026906664024 0.046494746848152274 -0.18925987040819986 0.2950315578288288 -0.07179084241479965 0.3544720161050883 0.18949282037489018 0.15188815499668423 0.043095232111012814 </vector>

<vector>0.24754122050013938 0.20544146702457722 0.18075819443223692 0.19362465357885852 -0.2749342982365849 0.05088573964160347 -0.660717067885786 -0.14764659228603336 -0.25168401110160704 -0.11177697923054991 -0.060636685712820784 -0.12461967017753713 0.03275863159116241 -0.17339023641889326 0.2772484678835192 -0.06541877495862927 0.35147969882869506 0.14911432116402448 0.15818584970104618 0.023786071662579563 </vector>

<vector>0.1573502102447867 0.1964909582085713 0.11108726952476863 0.21114749162370838 -0.32781162730366614 0.02847431450613884 -0.5052779650557467 -0.08497969985280472 -0.21992435796446777 -0.13247756517990966 -0.19662981662922788 -0.1387610463632254 0.060188153371549966 -0.17904664533106893 0.35751718342783756 -0.09441709184431087 0.44044889909347845 0.16048874673743388 0.1230520512906871 0.03308053749546694 </vector>

<vector>0.14130370132503525 0.12970755375631332 0.08428683175515975 0.14378184791272985 -0.4046870541393999 0.053423825024320595 -0.5333963423733887 -0.02443902770968508 -0.15985768331192723 -0.13598638526475829 -0.0836987561851257 -0.17120155148332827 0.012820753571713042 -0.18500359985824577 0.33152919290067867 -0.1036188537291002 0.44525394117795114 0.20618907693425859 0.16644541527930426 0.08714711441749542 </vector>

<vector>0.16077557539723733 0.1095156462397735 0.12438286462612014 0.11655930417191748 -0.34813836841514095 -0.057981261715176355 -0.4971924604345988 0.06460646028332771 -0.42263995057939313 -0.1623894890336857 -0.02467454793813525 -0.16142008216232598 0.05598714556331154 -0.18267125151911254 0.33044874488855347 -0.030830753356393115 0.4366426876792925 0.2368886671337143 0.18440830921275328 0.06772275995796062 </vector>

<vector>0.01981473558862308 0.13544030982839367 -0.06674715585977656 0.16592655219665298 -0.3241356872263724 0.13207010774324235 -0.48742602613164226 -0.0286025804702394 -0.22183949519644225 -0.1097262356454012 -0.1803192834763619 -0.13094886409193607 0.1615214834493024 -0.2417060411714546 0.4701072132668567 -0.06961735112950476 0.4206669688754865 0.12811136012720667 0.20835724671032643 0.019052742613040304 </vector>

</data>

</datamatrix>

</datavector>

<classifiernames>Taxa</classifiernames>

<classifiermat>

<vector>Acrocanthosaurus</vector>

<vector>Aerosteon</vector>

<vector>Afrovenator</vector>

<vector>Allosaurus</vector>

<vector>Aucasaurus</vector>

<vector>Avimimus</vector>

<vector>Bambiraptor</vector>

<vector>Baryonyx</vector>

<vector>Bicentenaria</vector>

<vector>Carnotaurus</vector>

<vector>Ceratosaurus</vector>

<vector>Citipati</vector>

<vector>Dilophosaurus</vector>

<vector>Dromaeosaurus</vector>

<vector>Eodromaeus</vector>

<vector>Eotyrannus</vector>

<vector>Eustreptospondylus</vector>

<vector>Falcarius</vector>

<vector>Gallimimus</vector>

<vector>Giganotosaurus</vector>

<vector>Guanlong</vector>

<vector>Herrerasaurus</vector>

<vector>Ilokelesia</vector>

<vector>Ingenia</vector>

<vector>Majungasaurus</vector>

<vector>Masiakasaurus</vector>

<vector>Ornitholestes</vector>

<vector>Qianzhousaurus</vector>

<vector>Saurornithoides</vector>

<vector>Shaochilong</vector>

<vector>Shuvuuia</vector>

<vector>Spinosaurus_I</vector>

<vector>Spinosaurus_II</vector>

<vector>Tawa</vector>

<vector>Torvosaurus</vector>

<vector>Tsaagan</vector>

<vector>Tyrannosaurus</vector>

</classifiermat>

<idvec>Acrocanthosaurus Aerosteon Afrovenator Allosaurus Aucasaurus Avimimus Bambiraptor Baryonyx Bicentenaria Carnotaurus Ceratosaurus Citipati Dilophosaurus Dromaeosaurus Eodromaeus Eotyrannus Eustreptospondylus Falcarius Gallimimus Giganotosaurus Guanlong Herrerasaurus Ilokelesia Ingenia Majungasaurus Masiakasaurus Ornitholestes Qianzhousaurus Saurornithoides Shaochilong Shuvuuia Spinosaurus_I Spinosaurus_II Tawa Torvosaurus Tsaagan Tyrannosaurus</idvec>

<meanconfig>0.13495631814332032 0.16154075134581164 0.047755353507686074 0.17203387836539474 -0.31079316962390124 0.08271234990638376 -0.51103806369261 -0.06715247516695218 -0.23736469910379962 -0.12718055089786032 -0.1250018957230973 -0.15702801365668548 0.03448588140856004 -0.2192626495455337 0.3667739636867369 -0.09217087296522046 0.4253854840073151 0.18539389683426025 0.17484082738978995 0.061113685780401716 </meanconfig>

<omitflag>false false false false false false false false false false false false false false false false false false false false false false false false false false false false false false false false false false false false false</omitflag>

<uniquenumvec>2 3 4 5 6 7 8 9 10 11 12 13 14 15 16 17 18 19 20 21 22 23 24 25 26 27 28 29 30 31 32 33 34 35 36 37 38 </uniquenumvec>

<linkedsets>41 </linkedsets>

<linksetmatrix>

<vector>0 </vector>

<vector>1 </vector>

<vector>2 </vector>

<vector>3 </vector>

<vector>4 </vector>

<vector>5 </vector>

<vector>6 </vector>

<vector>7 </vector>

<vector>8 </vector>

<vector>9 </vector>

<vector>10 </vector>

<vector>11 </vector>

<vector>12 </vector>

<vector>13 </vector>

<vector>14 </vector>

<vector>15 </vector>

<vector>16 </vector>

<vector>17 </vector>

<vector>18 </vector>

<vector>19 </vector>

<vector>20 </vector>

<vector>21 </vector>

<vector>22 </vector>

<vector>23 </vector>

<vector>24 </vector>

<vector>25 </vector>

<vector>26 </vector>

<vector>27 </vector>

<vector>28 </vector>

<vector>29 </vector>

<vector>30 </vector>

<vector>31 </vector>

<vector>32 </vector>

<vector>33 </vector>

<vector>34 </vector>

<vector>35 </vector>

<vector>36 </vector>

</linksetmatrix>

</morphojset_contents>

<covariancematrix>

<covariancematrix_contents visiblename="CovMatrix, Mandibular_articulation, Procrustes coordinates" df="36" dim="2" fromexternaldata="false" frommorphojset="true" hasobjectsymmetry="false" isasymmetrycomponent="false" issymmetrycomponent="false" linkedoutlinenumber="0" linkedwireframenumber="0" matrixtype="2" pooledwithingroupcovariances="false" uniqueid="39">

<covariancearray>

<vector>0.003916764928738676 7.82753978669761E-4 0.0036363992249821156 7.448993111482708E-4 3.295716691163733E-4 -0.0017508442877589292 -0.0017096213045337256 -1.81006490210751E-4 -0.0016121412430689856 -4.146118137433635E-4 4.3626092713564934E-4 3.123701051067582E-4 -1.973498229710078E-4 -5.356519818791763E-5 -0.0015378280142254517 0.0015343355143664786 -0.002380137774313465 -4.134503699161509E-6 -8.819185908601745E-4 -9.701966156911462E-4 </vector>

<vector>7.82753978669761E-4 0.0011706678969751738 8.037386602363394E-4 0.0012321050275635246 3.694863559821969E-4 3.403999681910214E-4 7.893027062794183E-5 -8.91043087834953E-4 6.855141732913338E-4 5.257606997980057E-4 -5.747078278408449E-4 -2.8633517764146434E-4 -2.533904889733994E-4 -9.281017846288484E-4 -2.391903129018267E-4 -2.9906846550503154E-4 -6.408226828706296E-4 2.160601853451359E-5 -0.0010123121262208756 -8.859910954519414E-4 </vector>

<vector>0.0036363992249821156 8.037386602363394E-4 0.005363415758359532 4.248988590082929E-4 8.005443022546713E-4 -0.0017808828943331105 -0.0013854766593671213 -5.290938884764177E-4 -0.002130865678572756 -2.802383431351729E-4 -1.66241290011435E-4 -4.33896775793209E-5 -9.106285484534044E-4 -1.8575628777861675E-4 -0.0018294798091518833 0.0017820632555997228 -0.0021819546958575086 8.734697433892447E-4 -0.0011957126041822026 -0.0010648094269309633 </vector>

<vector>7.448993111482708E-4 0.0012321050275635246 4.248988590082929E-4 0.002542910626637792 2.883041303325755E-4 0.001278837461340928 5.91050611887062E-4 -0.0016515369154017278 0.0021022234918618024 0.0010875475864491183 -0.0012526422072942334 -5.385803330147615E-4 -4.2124951785761785E-4 -0.002078774509764911 -5.3642022758093355E-5 -9.274180031548385E-4 -8.20789366267349E-4 -4.89561733457986E-5 -0.0016030532900607143 -8.961347673093248E-4 </vector>

<vector>3.295716691163733E-4 3.694863559821969E-4 8.005443022546713E-4 2.883041303325755E-4 0.002638854120923429 0.001339470470268723 -7.531820612698617E-4 -0.0014815460169043423 2.0746571335586404E-4 1.625714102978401E-4 -0.0015320859199516557 -4.6456246491935704E-4 -7.249110674363633E-4 -9.036136003149909E-4 -1.825223695259114E-4 2.762232589085462E-4 -7.923102059341791E-4 5.467444069705628E-4 8.575818467635024E-6 -1.330779506217531E-4 </vector>

<vector>-0.0017508442877589292 3.403999681910214E-4 -0.0017808828943331105 0.001278837461340928 0.001339470470268723 0.0041012811723282975 0.0010994521819534205 -0.002872321246878561 0.00427500069523572 0.0011693399124963447 -0.002475247785426753 -9.662455260724906E-4 -0.001499766947107926 -0.0020786366909809244 7.39725565955092E-4 -0.001999277671004947 7.811403730362016E-4 5.418224868481373E-4 -7.280473718224392E-4 4.84800133732195E-4 </vector>

<vector>-0.0017096213045337256 7.893027062794183E-5 -0.0013854766593671213 5.91050611887062E-4 -7.531820612698617E-4 0.0010994521819534205 0.0031885085347329754 1.8694446997754135E-4 0.0019093958688661628 6.503553495934471E-4 -0.0017889431185160324 -0.0011203897117348831 -0.0013524968071910422 -0.0016853314654682104 0.0013156875832362306 -0.0014497910057680423 0.0013132189569278574 0.0013375371577382224 -7.370909928854447E-4 3.112421411934983E-4 </vector>

<vector>-1.81006490210751E-4 -8.91043087834953E-4 -5.290938884764177E-4 -0.0016515369154017278 -0.0014815460169043423 -0.002872321246878561 1.8694446997754135E-4 0.004590154322416953 -0.002519672845541284 -0.0013923790583061232 0.001644406166890126 -1.0511953871660843E-4 0.0017772774004870971 6.935282757859966E-4 7.041863384639773E-4 0.0012039993953329017 -4.8606179498349476E-4 3.3919239855865064E-4 8.845666602975489E-4 8.552545504346537E-5 </vector>

<vector>-0.0016121412430689856 6.855141732913338E-4 -0.002130865678572756 0.0021022234918618024 2.0746571335586404E-4 0.00427500069523572 0.0019093958688661628 -0.002519672845541284 0.008662954488241921 0.0015740386035398685 -0.0047553972082000715 -0.0021287389602813937 -0.0026057573192901813 -0.003296396501524152 5.834015278705676E-4 -0.0031043639627684493 0.0011543856688092501 0.0013499191359609432 -0.0014134418180117736 0.0010624761702256083 </vector>

<vector>-4.146118137433635E-4 5.257606997980057E-4 -2.802383431351729E-4 0.0010875475864491183 1.625714102978401E-4 0.0011693399124963447 6.503553495934471E-4 -0.0013923790583061232 0.0015740386035398685 0.0013764156718429294 -0.0012389810795071345 -3.333207267486249E-4 -1.4062584553800234E-4 -0.0012176797763255778 3.169375056656045E-4 -7.092230187628274E-4 6.517898608774009E-5 9.936681817509158E-5 -6.946247732608259E-4 -6.058281086183364E-4 </vector>

<vector>4.3626092713564934E-4 -5.747078278408449E-4 -1.66241290011435E-4 -0.0012526422072942334 -0.0015320859199516557 -0.002475247785426753 -0.0017889431185160324 0.001644406166890126 -0.0047553972082000715 -0.0012389810795071345 0.006320041410981817 0.0016261057750704265 0.0012688367383740186 0.0030387417033180346 -0.0010685626739050807 7.798576520670745E-4 3.9554259930522337E-4 -0.0016242213762280933 8.905485347875656E-4 7.668897895140308E-5 </vector>

<vector>3.123701051067582E-4 -2.8633517764146434E-4 -4.33896775793209E-5 -5.385803330147615E-4 -4.6456246491935704E-4 -9.662455260724906E-4 -0.0011203897117348831 -1.0511953871660843E-4 -0.0021287389602813937 -3.333207267486249E-4 0.0016261057750704265 0.0017593612376633255 0.0014468242106375109 0.0016204587658741024 -7.64672042101374E-5 7.196535045983408E-4 -4.5931828859254985E-5 -0.0013373297476531522 4.941797567696526E-4 -5.325424582886649E-4 </vector>

<vector>-1.973498229710078E-4 -2.533904889733994E-4 -9.106285484534044E-4 -4.2124951785761785E-4 -7.249110674363633E-4 -0.001499766947107926 -0.0013524968071910422 0.0017772774004870971 -0.0026057573192901813 -1.4062584553800234E-4 0.0012688367383740186 0.0014468242106375109 0.0043412538397470035 0.0011425993834067135 6.404682825181078E-4 0.0010847719326789363 -0.001117098654040505 -0.0017892860812035425 6.576833587433739E-4 -0.0013471540465297663 </vector>

<vector>-5.356519818791763E-5 -9.281017846288484E-4 -1.8575628777861675E-4 -0.002078774509764911 -9.036136003149909E-4 -0.0020786366909809244 -0.0016853314654682104 6.935282757859966E-4 -0.003296396501524152 -0.0012176797763255778 0.0030387417033180346 0.0016204587658741024 0.0011425993834067135 0.003909273859116876 -8.845673040452206E-4 9.693313950552245E-4 0.0011488793720133448 -0.0016040619802705637 0.0016790098985810158 7.146624461386257E-4 </vector>

<vector>-0.0015378280142254517 -2.391903129018267E-4 -0.0018294798091518833 -5.3642022758093355E-5 -1.825223695259114E-4 7.39725565955092E-4 0.0013156875832362306 7.041863384639773E-4 5.834015278705676E-4 3.169375056656045E-4 -0.0010685626739050807 -7.64672042101374E-5 6.404682825181078E-4 -8.845673040452206E-4 0.0019884611693884676 -1.4217214670841135E-4 1.6906815161688222E-4 -1.335702105199256E-4 -7.869384782192965E-5 -2.312402089410592E-4 </vector>

<vector>0.0015343355143664786 -2.9906846550503154E-4 0.0017820632555997228 -9.274180031548385E-4 2.762232589085462E-4 -0.001999277671004947 -0.0014497910057680423 0.0012039993953329017 -0.0031043639627684493 -7.092230187628274E-4 7.798576520670745E-4 7.196535045983408E-4 0.0010847719326789363 9.693313950552245E-4 -1.4217214670841135E-4 0.002410030907894992 -0.0014175714846964697 -7.121230049665846E-4 6.566469863206171E-4 -6.55905039487231E-4 </vector>

<vector>-0.002380137774313465 -6.408226828706296E-4 -0.0021819546958575086 -8.20789366267349E-4 -7.923102059341791E-4 7.811403730362016E-4 0.0013132189569278574 -4.8606179498349476E-4 0.0011543856688092501 6.517898608774009E-5 3.9554259930522337E-4 -4.5931828859254985E-5 -0.001117098654040505 0.0011488793720133448 1.6906815161688222E-4 -0.0014175714846964697 0.0028544157210288485 1.3693383637217396E-4 5.848702324575948E-4 0.0012790445901677374 </vector>

<vector>-4.134503699161509E-6 2.160601853451359E-5 8.734697433892447E-4 -4.89561733457986E-5 5.467444069705628E-4 5.418224868481373E-4 0.0013375371577382224 3.3919239855865064E-4 0.0013499191359609432 9.936681817509158E-5 -0.0016242213762280933 -0.0013373297476531522 -0.0017892860812035425 -0.0016040619802705637 -1.335702105199256E-4 -7.121230049665846E-4 1.3693383637217396E-4 0.0024663790738083872 -6.933921087804199E-4 2.34104110311315E-4 </vector>

<vector>-8.819185908601745E-4 -0.0010123121262208756 -0.0011957126041822026 -0.0016030532900607143 8.575818467635024E-6 -7.280473718224392E-4 -7.370909928854447E-4 8.845666602975489E-4 -0.0014134418180117736 -6.946247732608259E-4 8.905485347875656E-4 4.941797567696526E-4 6.576833587433739E-4 0.0016790098985810158 -7.869384782192965E-5 6.566469863206171E-4 5.848702324575948E-4 -6.933921087804199E-4 0.0021651799093053577 0.0010170263681764415 </vector>

<vector>-9.701966156911462E-4 -8.859910954519414E-4 -0.0010648094269309633 -8.961347673093248E-4 -1.330779506217531E-4 4.84800133732195E-4 3.112421411934983E-4 8.552545504346537E-5 0.0010624761702256083 -6.058281086183364E-4 7.668897895140308E-5 -5.325424582886649E-4 -0.0013471540465297663 7.146624461386257E-4 -2.312402089410592E-4 -6.55905039487231E-4 0.0012790445901677374 2.34104110311315E-4 0.0010170263681764415 0.0020573093239298957 </vector>

</covariancearray>

</covariancematrix_contents>

<principal_component_analysis>

<pca_contents visiblename="PCA: CovMatrix, Mandibular_articulation, Procrustes coordinates" dimension="2" linkedoutlinenumber="0" linkedwireframenumber="42" npcs="16" uniqueid="40">

<meanconfig>0.13495631814332032 0.16154075134581164 0.047755353507686074 0.17203387836539474 -0.31079316962390124 0.08271234990638376 -0.51103806369261 -0.06715247516695218 -0.23736469910379962 -0.12718055089786032 -0.1250018957230973 -0.15702801365668548 0.03448588140856004 -0.2192626495455337 0.3667739636867369 -0.09217087296522046 0.4253854840073151 0.18539389683426025 0.17484082738978995 0.061113685780401716 </meanconfig>

<eigenvectors>

<vector>-0.14169506979502988 -0.45105579950524743 -0.06869328064783572 -0.04579066343182553 0.0970863273547006 -0.26705830962668026 0.0035281782882306866 0.24478476772992644 0.0021686898175562685 -0.1578620383734781 0.2878436646566466 0.3507062811919441 -0.39124473344707666 0.22214893668204047 -0.2013460707622901 0.10455737088065377 </vector>

<vector>0.06336606039314437 -0.1730571185733934 0.02473224328240748 0.12670190418279292 0.17782335204201713 0.04814135603964183 0.003196488800769339 -0.05270792056284574 -0.2653718701895081 -0.40769176250752304 -0.3153129975414936 -0.010652378323620381 -0.23097487922457116 -0.4339604482028704 0.26232770932583743 0.34882340178621957 </vector>

<vector>-0.13184842587354847 -0.5448578293142966 -0.12733271548132052 -0.23449355495932128 0.013976793983342157 0.053457664766389364 -0.2374323413416295 -0.1630491747193709 0.1650627465634048 0.2456166888779313 -0.04254925355205783 -0.43610329764999123 0.17197228269487702 -0.20575249726706732 0.19873804649928045 -0.0753755807950401 </vector>

<vector>0.16997109279823397 -0.2056216243909717 0.06877672987831536 0.2510328324301761 0.31514252291926453 -0.005905422486385089 0.08080249793391504 0.1755901902928901 -0.39061670747887905 -0.05315925027645716 0.1556786189103844 -0.13876619235725024 0.4190226484421206 0.1175122586206868 -0.17126474597619168 -0.4181806446687354 </vector>

<vector>0.07781543499150029 -0.17651730327054088 -0.12568917709880706 0.0721289577732909 -0.4818572104180535 0.311915186621863 0.3513780628495994 -0.1270487712663023 -0.15831780682732466 -0.3502101456653973 -0.10263356548931156 0.021070307235443987 0.1720725329672025 0.2752314305575059 0.06962239172606802 0.0192112863714557 </vector>

<vector>0.3443042721362864 0.05888545809302214 -0.15610759148379993 0.23309807099089003 -0.13712486663622472 0.18052388015595425 0.2723793165489211 0.002181353147516463 0.329223094627067 0.12824655801901644 0.18375868842559345 -0.2028092044380174 -0.2797842769677883 -0.28793369603667357 -0.33065283002261825 -0.0033909794097932173 </vector>

<vector>0.20852844336089105 0.14335628251380678 0.23601812483210458 -0.254723152041976 0.24810169691134115 0.35192817139211263 -0.2649246769935457 0.19537823317299258 -0.17110026386734534 -0.014247405921652774 0.2456860588732806 -0.04356285712361764 -0.12072737687038593 -0.16556187927429214 -0.13395471602641296 0.0653765027465996 </vector>

<vector>-0.2218761869353888 0.16266878065103305 0.4975159291623869 -0.3716514756512925 0.03518390409310835 -0.24437836960580783 0.24460356958838472 -0.0682996943543665 0.006095021866455692 -0.13600474237449595 -0.12198198211975649 -0.06072230577267006 0.09484943831005845 -0.002852903196825354 -0.03479283300855934 -0.014925208163565216 </vector>

<vector>0.542772541192403 0.045418130171341595 -0.05481900255558545 0.026289950930205298 0.047542863098356966 -0.63316745404881 -7.209972980209303E-4 -0.04985366875737162 0.1993817196298657 -0.01690641169006376 -0.2357574221014323 0.06437309232435025 0.12063695793014105 -0.051213857030737164 0.03699545498724098 -0.05626344501955511 </vector>

<vector>0.13920943355335466 -0.04815542498907616 0.04724314729883598 0.1907329231706255 0.09424955819576651 0.13065242334611002 -0.1607028179368594 -0.11995738611083812 -0.1732734332609899 0.5300411681506056 -0.28299163792694176 0.22215146171414624 -0.22979054673334748 0.38532426814739823 0.23929325164074094 0.0454215664245096 </vector>

<vector>-0.37464604892602305 0.17291406286946545 -0.2579053430541227 0.07273072753420187 0.5118299904531788 0.09689432219836434 0.5206147021588923 0.022724349732224803 0.1510130014180229 0.16262330568408803 -0.15220519891437617 0.018175292904227893 0.005606155922862396 -0.0211630573519286 0.03603587637202985 -0.014575247724234366 </vector>

<vector>-0.16703346906470481 0.04668036483924596 -0.07467572547592491 0.2622732311492203 0.008610224560943243 0.04161956153991861 -0.243430567983353 0.0517965500364554 0.34971273396508784 -0.10023176216868027 0.30343466482509673 0.3853849266214893 0.45882572153949175 -0.13827558176116503 0.22589556235822997 0.19472187556388165 </vector>

<vector>-0.21800780593764163 0.0934510069171677 0.4074061248178358 0.45428172598710753 -0.1010351677346827 -0.16609203250570428 -0.0572895966644008 -0.4419720085488601 -0.06323324627975331 0.08163488918075919 0.2609791472865598 -0.23684147231965297 -0.08590711100623565 0.06105602608408688 -0.10203658134830104 0.1712320933491256 </vector>

<vector>-0.28818365836608506 0.2061377635762178 -0.3354832205917499 0.030386041600594008 -0.06960862300869661 -0.11810943207351345 -0.3395641242466351 -0.11104814558613886 0.009189015986409475 -0.2964568556615208 -0.046114539690582115 -0.1500237832126668 -0.3162066758377008 0.06669361111234262 -0.009312302096270025 -0.49815943184496997 </vector>

<vector>0.07665608132276017 0.15951827129527327 0.34489325584300373 0.0785131159638729 -0.09787480970337273 0.20941451587857846 -0.04921338511414583 0.3937498904083493 0.3466081622190072 -0.11718868968254385 -0.10753805614414838 -0.05319108259743232 -0.11644361976509546 0.143333250906901 0.3717679793604251 -0.2628108401142557 </vector>

<vector>-0.22474146739492068 -0.14885617888795344 0.10772942740185415 -0.02734859655569307 -0.28106778046724856 0.0041690910256028425 -0.09748006971625657 0.3462395786699816 0.12761386549190182 0.17700657075253298 -0.39266906655230027 -0.019396820126730115 0.11186431004340905 0.009097915285391061 -0.4701159275142777 0.16665232829511248 </vector>

<vector>0.08310615266052575 0.3430168071265802 -0.255192479052669 -0.1245350090591679 0.12829165882724514 0.13998881126846413 -0.3040799821020786 -0.17709817016480175 -0.04516771272662798 -0.14136904261703534 -0.19108861068186483 0.00203910142850363 0.20889350640512427 0.20469841765404126 -0.3323272018149286 0.2550868369190167 </vector>

<vector>0.13748715822343585 -0.09357674725985152 0.07277470461272287 -0.4392505989158024 -0.007858511358938709 0.16300101884827448 0.12065586671116779 -0.46640193814928504 0.15814427886180943 0.03871046033486281 0.17411504204228254 0.3321170411663236 -0.027265736247171803 0.03234447956568766 -0.017740000737723725 -0.17558429049824897 </vector>

<vector>-0.1226813029958373 0.21475637119644922 -0.09868550760260345 -0.04440209869638844 -0.3660621427720565 -0.0972808759445778 0.03814003621709813 0.10238455241321211 -0.4264152899468019 0.3079088502073986 0.037263236066706305 0.3133346346062254 0.03514140516858583 -0.46277677096054787 0.05650482100688936 -0.2064389766137533 </vector>

<vector>0.047496764656643944 0.19489472694172746 -0.2525056440850486 -0.25597433240150985 -0.13534978033999095 -0.1997141067897951 0.11953984029994602 0.24260741261663182 -0.15071599986935683 0.11953961573166101 0.34208320962771904 -0.35728274527100173 -5.400033245038007E-4 0.25205009646602605 0.3063621160308358 0.3546213825155951 </vector>

</eigenvectors>

<eigenvalues>0.024281890033311583 0.013597841096230315 0.006967602626591791 0.006628995718352393 0.004715414325630483 0.003362649377523066 0.002492214348495644 0.001474420473739301 0.0010562693859631916 8.067713130402834E-4 7.550054215464351E-4 6.469667559778586E-4 3.484114880634536E-4 3.2725961299711617E-4 2.5810040487080614E-4 1.0382159172896895E-4 </eigenvalues>

<percentvar>35.801517274343524 20.04882413323715 10.273118997511046 9.773872807946963 6.9524648700389085 4.95793159477273 3.674551483703636 2.1739036783301127 1.557376572253756 1.189513545306067 1.1131892782907598 0.9538957411590087 0.5137021826295743 0.4825155979142428 0.38054641095979846 0.15307583160270163 </percentvar>

</pca_contents>

<morphojset>

<morphojset_contents visiblename="PC scores, CovMatrix, Mandibular_articulation, Procrustes coordinates" uniqueid="41" alignmentmethod="0" dimension="1" hasprocrustesfit="false" hasrawdata="false" linkedoutlinenumber="0" linkedwireframenumber="0" nincl="37" nlmk="10" nobs="37" objsymmetry="false" procsumsquare="0.0" procrustesfitfromparentset="false">

<alignmentvectors>

<vector>0.0 </vector>

<vector>0.0 </vector>

<vector>0.0 </vector>

<vector>0.0 </vector>

<vector>0.0 </vector>

<vector>0.0 </vector>

<vector>0.0 </vector>

<vector>0.0 </vector>

<vector>0.0 </vector>

<vector>0.0 </vector>

</alignmentvectors>

<datavector>

<datamatrix visiblename="PC scores, CovMatrix, Mandibular_articulation, Procrustes coordinates" matrixtype="9" iscoordinates="false" issymmetrycomponent="false" isasymmetrycomponent="false">

<data>

<vector>-0.07407441946607675 -0.030944532103437762 -0.04373524480657211 0.059358699460411515 -0.06582798141499999 -0.04507237196101295 -0.007886068400516408 0.08167504423167864 0.03869330565548954 0.030704149490119986 -0.00690830729782867 0.03382033905113749 -0.002447324575263387 0.029001260257303374 0.018632126765414186 0.010730701898589698 </vector>

<vector>0.09881980468606923 -0.02269212419574982 -0.053655441503745745 0.04987948096641691 -0.14757096282291407 -0.01926950294298207 0.009075269812611732 -0.026243410349920563 -0.021087671171722038 0.01605851641036596 0.023417857696357774 -0.0014437366882686757 0.012387795380694924 -0.006072169365135733 -0.019785295485002505 0.01713555081006478 </vector>

<vector>0.008730157034063265 -0.05183966319343974 0.0010874020888086717 -0.05405682845630833 0.12105963230246182 0.06104752481002845 0.002801885581973901 -0.08686572113770126 0.025035799103707037 0.03424722174948665 -0.004719301591433925 0.01783113325439433 -0.03907779206887204 0.011906277778962035 8.692777724355095E-4 0.0036694165954075236 </vector>

<vector>-0.03578538789199292 -0.005106722311740984 -0.06340253990797007 0.00672304728259361 0.09453920325214317 -0.013200499075567206 -0.008571893195579876 0.06461332022407414 0.036856358486962434 -0.015844087955356728 0.04192856801673233 0.04146496211367286 0.030784963647356365 -0.03149478718200867 2.086866768514291E-4 0.009318540924226258 </vector>

<vector>0.23003457104822445 -0.006939861187613066 0.0390332443991234 -0.0563631632703632 -0.006962316279664112 0.025724272184645187 0.07268483845039306 -0.028066347678863204 -0.04770481155725164 0.01488494043320513 -0.03856762748052459 -0.006739793564446761 -0.012218841187808544 -0.007736117972830532 -0.007838079796914034 -0.003268049965417125 </vector>

<vector>0.07306726945919192 -0.06957612459960834 0.02970745372359915 -0.0060137125538983534 -0.03994625227357998 -0.01725748698448425 -0.024936886687680355 -0.04574273641324161 0.009191877138783517 -0.0022526745237638635 0.0012278587652539003 0.018584942187315386 -0.021299476920659012 -0.03938154593340692 0.009184689774364917 0.006413497606565453 </vector>

<vector>-0.2768864185441912 0.12756755319245058 0.03966557387249317 0.012269075812092523 0.037402657948281307 -0.00823420103561992 -0.009888814937066155 3.9157392285671063E-4 -0.044288003411199144 0.025665631701620815 0.023763804038861424 0.011527729407772956 7.957936347641389E-4 -0.03045495781798602 -0.0035670094431652574 -0.008754704352106197 </vector>

<vector>-0.23708280493433173 -0.123728879014261 0.018154591926256798 0.13367853343930125 0.040457269141063144 0.11938334731750691 0.02193642485282636 -0.054921014272055695 0.09121747377635185 -0.015595269546525552 0.01532941384635133 -0.009468603129386584 0.015753338988777985 -0.005580467179359068 -0.009125813563495566 -4.1316427460940525E-4 </vector>

<vector>0.06690409456286403 -0.18710582864637443 -0.15497552179236934 0.11645131775867337 0.06987002583202886 -0.05255968328157101 0.03676662838249399 0.022382196691795014 -0.0128148650735696 0.021597630676767596 0.025086430773087753 -0.01789768198500252 -0.020237268129916403 0.02727474679065089 -0.016939764315515166 0.007829077395937006 </vector>

<vector>0.3295753916203034 0.03634909083778483 3.8295951006786337E-4 -0.08798291273293585 0.043262069105644285 0.04780807818815472 -0.04881152804654255 0.017969082820704217 0.0351111655850546 1.3992076951048656E-4 0.014615778133723237 0.003113708858441852 0.027355660110095513 0.0182424570513862 -0.008415583735090274 -0.01077526558691734 </vector>

<vector>0.146479741442677 -0.19085672899501943 0.03790779786948987 -0.008985396915182547 -0.05293433366413976 -0.011127159829806051 -0.03908990285631423 0.024118513471768153 0.0014387634851884554 -0.01439152220221497 0.013163899499077855 0.018616315013993058 -0.015107276402579448 -0.01225711956195578 0.00907709393514565 0.0016917066356894224 </vector>

<vector>0.0770731382755203 -0.012967649981257894 0.02629359094901625 0.055799665857359904 0.044756878573067066 0.0021144543625427146 0.04112263816668279 0.08987861053574124 -0.003978390447392363 -0.030495537272670063 -0.014099173770890261 -0.03417086925206333 -0.04239315761536837 0.005842675501249598 -0.034585854741453585 -0.005663646817595826 </vector>

<vector>-0.19214166212762812 -0.10112578593042224 0.08426577096286021 -0.07595707454389798 0.058695352829619074 -0.05112789036335766 -0.046247322912334785 0.0492132859093383 -0.04728048350340061 0.015730379306248148 -0.003744962185755328 -0.010982481779117002 -0.009473161168085824 -0.018382713507512794 0.013126875817812887 0.01089156668682543 </vector>

<vector>-0.08576032316605335 0.10202688865058637 0.045725128290492634 -0.01859393292712598 -0.09480085860141728 0.07460709190878978 0.09581406724092371 0.016737720911750836 -0.0078076697099093584 -0.010716102560322727 0.043141704985829664 -0.011128398861404844 0.0027466059973859126 0.026504065066871994 0.029535628423911685 0.009733454398397713 </vector>

<vector>-0.005151794665477164 -0.05355863007086613 0.019199699235100964 0.05451787113211316 -0.03708771965009674 0.0802361387369558 0.09882352080703598 -0.0028449866196078753 -0.03308182406869897 -0.015013498476973582 -0.01554786100873189 -0.0030155327963219247 0.015827877492744884 -0.019063187941119775 -0.0017250405993813021 -0.004342530128424534 </vector>

<vector>0.07584296489493268 0.025485860599546425 -0.054550703333135636 -0.026074533926013985 -0.002634352241412657 0.03569492816953406 0.01614351976124044 -0.03911180256230955 -0.030323381508726443 0.040038357229500596 -0.031149568036270836 -0.012072349772731007 0.012680237279243887 0.009572130991218102 0.00699739426108187 0.014129416995808462 </vector>

<vector>-0.17956509780411745 -0.07525542274166432 0.09399326059413925 -0.1106248368408316 -0.04030751378139304 0.01101124338259498 -0.055273224720574804 0.019029656336355068 -0.017829362661585295 0.014433799773808044 0.001992993513589768 0.011133466067135316 -0.004387898348374415 -0.002406519891764364 0.0018344886809213388 0.013185243303265124 </vector>

<vector>0.012642765476891561 0.18314565436893343 0.12849858310596618 0.21483085895193532 0.02847725758088266 0.020332586106144517 -0.028163929082044646 0.003758546581178761 -0.010490809913275043 0.04826189084769837 0.03314174334109498 -7.861761937124831E-5 -0.022645219933568276 0.007214080609834004 0.01935397197727083 -0.011491142557494099 </vector>

<vector>-0.1759601708013632 0.16490061243356904 0.15174669399911275 0.01327341817249831 0.06560443937590511 -0.18207848815502287 0.10638561566342228 -0.022947688328165225 0.02126846553807994 -0.04598234599846712 -0.03584666840892194 0.015201761482050971 0.00958398390568851 0.017682088341126483 -0.0027622482338895407 0.00557855104868227 </vector>

<vector>-0.07783994098814478 0.0660248346509535 -0.03453243673152475 -0.0773749390207566 -0.00153807326560272 0.038861571616182575 -0.03476431725251634 0.04668189097911473 0.01905265980654937 0.013610169577122898 -0.04752772383232513 -0.008353370343960408 0.02128850990063457 0.007795224066715971 -0.016598394763029864 -0.003421289037317068 </vector>

<vector>0.008336877881833767 -0.08956529011591412 0.05627729453836107 0.07139164714071376 -0.1392115995517448 -0.086521200215378 -0.08528644034237812 -0.038498352648543774 -0.0027519426558626995 0.011771787370387452 0.009475295314911026 -0.029661627412901355 0.011484192340816927 0.015518590497073458 -0.028912770890783424 8.571824521952211E-4 </vector>

<vector>0.12235169332782041 -0.07009800151712757 -0.010402590739079978 0.001280580494033067 -0.10742054817627888 0.004793965024484311 0.030727575409761548 0.008860445433891096 0.011483440930149538 0.009779113567837483 -0.0394246964356818 0.033780558504713384 0.017829195210772284 0.013986921015872329 0.0232609860828506 -0.014374034894293279 </vector>

<vector>0.192738761293728 -0.030438172377309412 0.08702468607761071 -0.08516053879861503 -0.06271873188410876 -0.017907734510559718 -0.007470316918785922 -0.026722724467523325 -4.0220684235497007E-4 -0.05346952624958742 0.0029702581690017344 0.044052192170499604 -0.017263396297666156 0.004183313309849809 0.002239890974685667 -0.005842383712529326 </vector>

<vector>0.28926417525762765 0.06171898511242188 0.0769399601252512 0.048767181188567967 0.11984233993353623 0.049138511903336526 -0.057815636388227586 -8.257294972913868E-4 0.009711271339717708 -0.041528090703237125 -0.021634508097840795 -0.03493344028414215 -7.033282789350274E-5 0.0059208122245789025 0.02632574638850126 0.024335794239597513 </vector>

<vector>0.19146418185985895 0.053391943086381094 -0.04717104613919758 -0.13355227267123798 -0.031002533246324085 -0.048974696487553045 0.09578697872457434 0.023443547855231676 0.057267365098236746 0.02414356781735743 0.02604216848668502 -0.05161200060701826 0.0011743563515544818 -0.03209704420288015 0.001403443529979606 0.004883358793241935 </vector>

<vector>0.2186251940591412 0.0637266699479892 -0.017067141585372158 -0.06818799857924404 0.0674120524228605 -0.03787861857863757 1.0934146978832174E-4 -0.00869655313525321 -0.026563359643868377 0.02359840667969551 0.05691101912317677 -0.002575063249927425 0.011082889155598937 0.003419765686935368 0.010208766530553491 -0.018084663999538144 </vector>

<vector>0.10587823269954236 0.031647875430519 -0.0854434495512192 0.11633959153775596 0.08915923786158222 0.031015160218520556 8.055923963159965E-4 0.015938167060267877 -0.05661579822340085 0.007437002316734735 -0.04901487576866615 0.033435729355734585 0.02860628485999974 -0.017908086643978353 -0.011899770157180392 0.0035876833261276 </vector>

<vector>0.014298391082011199 0.03425081368167789 0.0032681909869804173 -0.03512942310119519 0.003590271666078787 0.035669600395445804 9.96514225669249E-4 -0.0052514095166889905 0.010093153747269655 -0.01675087951464606 0.021331120722155924 0.06938210653352386 -0.01884982659377732 0.00826246179654069 -0.036039881159537365 8.075527815541977E-4 </vector>

<vector>-0.056687163608274554 0.270220328157836 -0.21138365079754826 0.00987417210299021 -0.009959661176353465 -0.02206355837626869 -0.021936208357794813 -0.02965593357704594 -0.0442030168093165 -0.04540120906946575 0.03068064351772592 0.006025298016567732 -0.0014554248601967468 0.015953630985029298 0.0054611523209381965 -5.231694721561653E-4 </vector>

<vector>-0.18066735556674549 -0.07465748266764956 -1.57493865551895E-4 -0.07220560565455038 -0.019759836868760382 0.08519213670765745 -0.006414278539600735 0.06108346150661763 -0.010594738069324918 -0.012631416849901421 -0.01431327008469181 -0.014708602215290587 -0.013791987574220224 0.010717246557477675 0.00854178006812209 -0.01940783960518954 </vector>

<vector>-0.15487444420409524 0.26570338789666087 -0.17877166877094797 -0.03122628880370694 -0.09224209883430382 0.0295961127656815 -0.059451617120358596 -0.008195178742465156 0.03628801091335119 -0.01788999551562196 -0.036433618257772665 -0.016577242846284177 -0.03345722743665864 -0.01983729605700732 0.002274573591044028 0.00224569358429098 </vector>

<vector>-0.15936410477827845 -0.1827938789522531 -0.0376513914412589 -0.01571536898396652 0.005167616012918643 -2.0649222850904932E-4 0.03559295966354 -0.023653448596465766 -0.029459051991426337 -0.052549967525288545 0.02856744562343018 -0.029029769565627542 0.0073336286867819915 -0.010341342598880872 0.0032940404243412044 -0.00821357404230914 </vector>

<vector>-0.12029063356367553 -0.2085676174208213 -0.1313179228234012 0.08187431692620316 0.00868907457270658 -0.060824085658187456 0.01707275594471195 -0.030084265769451272 0.009851116056157107 0.00885472901260369 -0.030191139622164107 0.006484098015473201 -0.002748227329872872 -0.0016369400494643128 0.026967791612141218 -0.01032049820135074 </vector>

<vector>4.621783225930707E-4 -0.07098657290544473 0.013028625952167562 0.019459963093367377 0.022887789545317984 -0.030054860894253858 -0.10658988818475114 -0.019750942970576037 -0.013401900967593215 -0.05107080298640798 -0.003981465900275998 -0.03444559101517 0.019530857858400807 0.009542272935484757 0.006606608612078112 -0.007327578457949905 </vector>

<vector>-0.014830849686827092 0.02682686866385204 -0.03274035985649997 -0.07327160610280259 0.06405778211683448 -0.10689006338493508 -0.01760433524531467 -0.025540892359840223 0.04315072830568552 0.036948315920892934 -0.017436754821229618 -0.011679028259246443 3.3000669456985154E-4 -0.004725591008317481 8.731445145580699E-4 -0.014345367269834867 </vector>

<vector>-0.27114628082930314 -0.0038258806558260972 0.05061612232390655 -0.152881925418815 0.039626206789424905 0.04537378630484819 -4.4604044280733266E-4 -0.029575106562340185 -0.014245201611694906 0.02720825952138406 0.007465128246978923 -0.008662575834373635 0.018852153298119116 0.02830962054949915 -0.016256631547031025 0.006601309084294323 </vector>

<vector>0.035519268341681814 0.14964348287263898 0.15414197311459021 0.1235889379844202 -0.07263178312926172 0.013648083860652475 -0.015997476922776328 0.007419180732986299 0.01921353487483891 0.01646913677810481 -0.009711609213018118 -0.015217962950370539 0.011495508476779477 -0.017473755100053237 -0.011826020303535672 -0.007056396185729412 </vector>

</data>

</datamatrix>

</datavector>

<classifiernames>Taxa</classifiernames>

<classifiermat>

<vector>Acrocanthosaurus</vector>

<vector>Aerosteon</vector>

<vector>Afrovenator</vector>

<vector>Allosaurus</vector>

<vector>Aucasaurus</vector>

<vector>Avimimus</vector>

<vector>Bambiraptor</vector>

<vector>Baryonyx</vector>

<vector>Bicentenaria</vector>

<vector>Carnotaurus</vector>

<vector>Ceratosaurus</vector>

<vector>Citipati</vector>

<vector>Dilophosaurus</vector>

<vector>Dromaeosaurus</vector>

<vector>Eodromaeus</vector>

<vector>Eotyrannus</vector>

<vector>Eustreptospondylus</vector>

<vector>Falcarius</vector>

<vector>Gallimimus</vector>

<vector>Giganotosaurus</vector>

<vector>Guanlong</vector>

<vector>Herrerasaurus</vector>

<vector>Ilokelesia</vector>

<vector>Ingenia</vector>

<vector>Majungasaurus</vector>

<vector>Masiakasaurus</vector>

<vector>Ornitholestes</vector>

<vector>Qianzhousaurus</vector>

<vector>Saurornithoides</vector>

<vector>Shaochilong</vector>

<vector>Shuvuuia</vector>

<vector>Spinosaurus_I</vector>

<vector>Spinosaurus_II</vector>

<vector>Tawa</vector>

<vector>Torvosaurus</vector>

<vector>Tsaagan</vector>

<vector>Tyrannosaurus</vector>

</classifiermat>

<idvec>Acrocanthosaurus Aerosteon Afrovenator Allosaurus Aucasaurus Avimimus Bambiraptor Baryonyx Bicentenaria Carnotaurus Ceratosaurus Citipati Dilophosaurus Dromaeosaurus Eodromaeus Eotyrannus Eustreptospondylus Falcarius Gallimimus Giganotosaurus Guanlong Herrerasaurus Ilokelesia Ingenia Majungasaurus Masiakasaurus Ornitholestes Qianzhousaurus Saurornithoides Shaochilong Shuvuuia Spinosaurus_I Spinosaurus_II Tawa Torvosaurus Tsaagan Tyrannosaurus</idvec>

<meanconfig>0.13495631814332032 0.16154075134581164 0.047755353507686074 0.17203387836539474 -0.31079316962390124 0.08271234990638376 -0.51103806369261 -0.06715247516695218 -0.23736469910379962 -0.12718055089786032 -0.1250018957230973 -0.15702801365668548 0.03448588140856004 -0.2192626495455337 0.3667739636867369 -0.09217087296522046 0.4253854840073151 0.18539389683426025 0.17484082738978995 0.061113685780401716 </meanconfig>

<omitflag>false false false false false false false false false false false false false false false false false false false false false false false false false false false false false false false false false false false false false</omitflag>

<uniquenumvec>2 3 4 5 6 7 8 9 10 11 12 13 14 15 16 17 18 19 20 21 22 23 24 25 26 27 28 29 30 31 32 33 34 35 36 37 38 </uniquenumvec>

<linkedsets>1 </linkedsets>

<linksetmatrix>

<vector>0 </vector>

<vector>1 </vector>

<vector>2 </vector>

<vector>3 </vector>

<vector>4 </vector>

<vector>5 </vector>

<vector>6 </vector>

<vector>7 </vector>

<vector>8 </vector>

<vector>9 </vector>

<vector>10 </vector>

<vector>11 </vector>

<vector>12 </vector>

<vector>13 </vector>

<vector>14 </vector>

<vector>15 </vector>

<vector>16 </vector>

<vector>17 </vector>

<vector>18 </vector>

<vector>19 </vector>

<vector>20 </vector>

<vector>21 </vector>

<vector>22 </vector>

<vector>23 </vector>

<vector>24 </vector>

<vector>25 </vector>

<vector>26 </vector>

<vector>27 </vector>

<vector>28 </vector>

<vector>29 </vector>

<vector>30 </vector>

<vector>31 </vector>

<vector>32 </vector>

<vector>33 </vector>

<vector>34 </vector>

<vector>35 </vector>

<vector>36 </vector>

</linksetmatrix>

</morphojset_contents>

<phylogeny>

<phylogeny_content visiblename="Theropoda_Quadrate" matchestablished="true" mappingdone="true" ntaxa="37" rooted="true" weightedparsimony="false" withbranchlengths="false" uniqueid="154" matchclassifierindex="0" datatype="9" haspermtest="false" permiterations="0" permpval="NaN" treelength="0.9862015574327782">

<descendancest>

<vector>1 0 </vector>

<vector>2 0 </vector>

<vector>3 2 </vector>

<vector>4 2 </vector>

<vector>5 4 </vector>

<vector>6 4 </vector>

<vector>7 6 </vector>

<vector>8 6 </vector>

<vector>9 8 </vector>

<vector>10 9 </vector>

<vector>11 9 </vector>

<vector>12 11 </vector>

<vector>13 11 </vector>

<vector>14 13 </vector>

<vector>15 13 </vector>

<vector>16 15 </vector>

<vector>17 15 </vector>

<vector>18 17 </vector>

<vector>19 17 </vector>

<vector>20 8 </vector>

<vector>21 20 </vector>

<vector>22 21 </vector>

<vector>23 22 </vector>

<vector>24 22 </vector>

<vector>25 24 </vector>

<vector>26 24 </vector>

<vector>27 21 </vector>

<vector>28 27 </vector>

<vector>29 27 </vector>

<vector>30 29 </vector>

<vector>31 29 </vector>

<vector>32 20 </vector>

<vector>33 32 </vector>

<vector>34 33 </vector>

<vector>35 33 </vector>

<vector>36 35 </vector>

<vector>37 35 </vector>

<vector>38 37 </vector>

<vector>39 37 </vector>

<vector>40 39 </vector>

<vector>41 39 </vector>

<vector>42 32 </vector>

<vector>43 42 </vector>

<vector>44 42 </vector>

<vector>45 44 </vector>

<vector>46 45 </vector>

<vector>47 45 </vector>

<vector>48 47 </vector>

<vector>49 47 </vector>

<vector>50 49 </vector>

<vector>51 49 </vector>

<vector>52 44 </vector>

<vector>53 52 </vector>

<vector>54 52 </vector>

<vector>55 54 </vector>

<vector>56 54 </vector>

<vector>57 56 </vector>

<vector>58 56 </vector>

<vector>59 58 </vector>

<vector>60 58 </vector>

<vector>61 60 </vector>

<vector>62 61 </vector>

<vector>63 61 </vector>

<vector>64 63 </vector>

<vector>65 63 </vector>

<vector>66 60 </vector>

<vector>67 66 </vector>

<vector>68 66 </vector>

<vector>69 68 </vector>

<vector>70 68 </vector>

<vector>71 70 </vector>

<vector>72 70 </vector>

</descendancest>

<nodeNames>Root Herrerasaurus Node 1 Eodromaeus Node 2 Tawa Node 3 Dilophosaurus Node 4 Node 5 Ceratosaurus Node 6 Masiakasaurus Node 7 Ilokelesia Node 8 Carnotaurus Node 9 Majungasaurus Aucasaurus Node 10 Node 11 Node 12 Eustreptospondylus Node 13 Afrovenator Torvosaurus Node 14 Baryonyx Node 15 Spinosaurus_II Spinosaurus_I Node 16 Node 17 Allosaurus Node 18 Aerosteon Node 19 Acrocanthosaurus Node 20 Shaochilong Giganotosaurus Node 21 Bicentenaria Node 22 Node 23 Guanlong Node 24 Eotyrannus Node 25 Qianzhousaurus Tyrannosaurus Node 26 Gallimimus Node 27 Ornitholestes Node 28 Falcarius Node 29 Shuvuuia Node 30 Node 31 Avimimus Node 32 Citipati Ingenia Node 33 Saurornithoides Node 34 Dromaeosaurus Node 35 Bambiraptor Tsaagan</nodeNames>

<taxanames>Acrocanthosaurus Aerosteon Afrovenator Allosaurus Aucasaurus Avimimus Bambiraptor Baryonyx Bicentenaria Carnotaurus Ceratosaurus Citipati Dilophosaurus Dromaeosaurus Eodromaeus Eotyrannus Eustreptospondylus Falcarius Gallimimus Giganotosaurus Guanlong Herrerasaurus Ilokelesia Ingenia Majungasaurus Masiakasaurus Ornitholestes Qianzhousaurus Saurornithoides Shaochilong Shuvuuia Spinosaurus_I Spinosaurus_II Tawa Torvosaurus Tsaagan Tyrannosaurus</taxanames>

<taxonvec>0 1 2 3 4 5 6 7 8 9 10 11 12 13 14 15 16 17 18 19 20 21 22 23 24 25 26 27 28 29 30 31 32 33 34 35 36 </taxonvec>

<ancestorvec>53 54 59 55 63 41 37 58 51 64 67 40 69 38 71 48 60 44 46 52 49 72 65 40 63 66 45 47 39 52 43 57 57 70 59 37 47 38 39 42 41 42 43 44 45 46 50 48 49 50 51 56 53 54 55 56 62 58 61 60 61 62 68 64 65 66 67 68 69 70 71 72 </ancestorvec>

<branchlengthvec>1.0 1.0 1.0 1.0 1.0 1.0 1.0 1.0 1.0 1.0 1.0 1.0 1.0 1.0 1.0 1.0 1.0 1.0 1.0 1.0 1.0 1.0 1.0 1.0 1.0 1.0 1.0 1.0 1.0 1.0 1.0 1.0 1.0 1.0 1.0 1.0 1.0 1.0 1.0 1.0 1.0 1.0 1.0 1.0 1.0 1.0 1.0 1.0 1.0 1.0 1.0 1.0 1.0 1.0 1.0 1.0 1.0 1.0 1.0 1.0 1.0 1.0 1.0 1.0 1.0 1.0 1.0 1.0 1.0 1.0 1.0 1.0 </branchlengthvec>

<interntermcov>

<vector>6.0 6.0 5.0 6.0 4.0 13.0 16.0 5.0 7.0 4.0 4.0 13.0 3.0 15.0 1.0 8.0 5.0 11.0 9.0 6.0 8.0 0.0 4.0 13.0 4.0 4.0 10.0 8.0 14.0 6.0 12.0 5.0 5.0 2.0 5.0 16.0 8.0 </vector>

<vector>6.0 6.0 5.0 6.0 4.0 13.0 15.0 5.0 7.0 4.0 4.0 13.0 3.0 15.0 1.0 8.0 5.0 11.0 9.0 6.0 8.0 0.0 4.0 13.0 4.0 4.0 10.0 8.0 14.0 6.0 12.0 5.0 5.0 2.0 5.0 15.0 8.0 </vector>

<vector>6.0 6.0 5.0 6.0 4.0 13.0 14.0 5.0 7.0 4.0 4.0 13.0 3.0 14.0 1.0 8.0 5.0 11.0 9.0 6.0 8.0 0.0 4.0 13.0 4.0 4.0 10.0 8.0 14.0 6.0 12.0 5.0 5.0 2.0 5.0 14.0 8.0 </vector>

<vector>6.0 6.0 5.0 6.0 4.0 14.0 13.0 5.0 7.0 4.0 4.0 15.0 3.0 13.0 1.0 8.0 5.0 11.0 9.0 6.0 8.0 0.0 4.0 15.0 4.0 4.0 10.0 8.0 13.0 6.0 12.0 5.0 5.0 2.0 5.0 13.0 8.0 </vector>

<vector>6.0 6.0 5.0 6.0 4.0 14.0 13.0 5.0 7.0 4.0 4.0 14.0 3.0 13.0 1.0 8.0 5.0 11.0 9.0 6.0 8.0 0.0 4.0 14.0 4.0 4.0 10.0 8.0 13.0 6.0 12.0 5.0 5.0 2.0 5.0 13.0 8.0 </vector>

<vector>6.0 6.0 5.0 6.0 4.0 13.0 13.0 5.0 7.0 4.0 4.0 13.0 3.0 13.0 1.0 8.0 5.0 11.0 9.0 6.0 8.0 0.0 4.0 13.0 4.0 4.0 10.0 8.0 13.0 6.0 12.0 5.0 5.0 2.0 5.0 13.0 8.0 </vector>

<vector>6.0 6.0 5.0 6.0 4.0 12.0 12.0 5.0 7.0 4.0 4.0 12.0 3.0 12.0 1.0 8.0 5.0 11.0 9.0 6.0 8.0 0.0 4.0 12.0 4.0 4.0 10.0 8.0 12.0 6.0 12.0 5.0 5.0 2.0 5.0 12.0 8.0 </vector>

<vector>6.0 6.0 5.0 6.0 4.0 11.0 11.0 5.0 7.0 4.0 4.0 11.0 3.0 11.0 1.0 8.0 5.0 11.0 9.0 6.0 8.0 0.0 4.0 11.0 4.0 4.0 10.0 8.0 11.0 6.0 11.0 5.0 5.0 2.0 5.0 11.0 8.0 </vector>

<vector>6.0 6.0 5.0 6.0 4.0 10.0 10.0 5.0 7.0 4.0 4.0 10.0 3.0 10.0 1.0 8.0 5.0 10.0 9.0 6.0 8.0 0.0 4.0 10.0 4.0 4.0 10.0 8.0 10.0 6.0 10.0 5.0 5.0 2.0 5.0 10.0 8.0 </vector>

<vector>6.0 6.0 5.0 6.0 4.0 9.0 9.0 5.0 7.0 4.0 4.0 9.0 3.0 9.0 1.0 8.0 5.0 9.0 9.0 6.0 8.0 0.0 4.0 9.0 4.0 4.0 9.0 8.0 9.0 6.0 9.0 5.0 5.0 2.0 5.0 9.0 8.0 </vector>

<vector>6.0 6.0 5.0 6.0 4.0 8.0 8.0 5.0 7.0 4.0 4.0 8.0 3.0 8.0 1.0 10.0 5.0 8.0 8.0 6.0 9.0 0.0 4.0 8.0 4.0 4.0 8.0 11.0 8.0 6.0 8.0 5.0 5.0 2.0 5.0 8.0 11.0 </vector>

<vector>6.0 6.0 5.0 6.0 4.0 8.0 8.0 5.0 7.0 4.0 4.0 8.0 3.0 8.0 1.0 10.0 5.0 8.0 8.0 6.0 9.0 0.0 4.0 8.0 4.0 4.0 8.0 10.0 8.0 6.0 8.0 5.0 5.0 2.0 5.0 8.0 10.0 </vector>

<vector>6.0 6.0 5.0 6.0 4.0 8.0 8.0 5.0 7.0 4.0 4.0 8.0 3.0 8.0 1.0 9.0 5.0 8.0 8.0 6.0 9.0 0.0 4.0 8.0 4.0 4.0 8.0 9.0 8.0 6.0 8.0 5.0 5.0 2.0 5.0 8.0 9.0 </vector>

<vector>6.0 6.0 5.0 6.0 4.0 8.0 8.0 5.0 7.0 4.0 4.0 8.0 3.0 8.0 1.0 8.0 5.0 8.0 8.0 6.0 8.0 0.0 4.0 8.0 4.0 4.0 8.0 8.0 8.0 6.0 8.0 5.0 5.0 2.0 5.0 8.0 8.0 </vector>

<vector>6.0 6.0 5.0 6.0 4.0 7.0 7.0 5.0 7.0 4.0 4.0 7.0 3.0 7.0 1.0 7.0 5.0 7.0 7.0 6.0 7.0 0.0 4.0 7.0 4.0 4.0 7.0 7.0 7.0 6.0 7.0 5.0 5.0 2.0 5.0 7.0 7.0 </vector>

<vector>9.0 8.0 5.0 7.0 4.0 6.0 6.0 5.0 6.0 4.0 4.0 6.0 3.0 6.0 1.0 6.0 5.0 6.0 6.0 10.0 6.0 0.0 4.0 6.0 4.0 4.0 6.0 6.0 6.0 10.0 6.0 5.0 5.0 2.0 5.0 6.0 6.0 </vector>

<vector>9.0 8.0 5.0 7.0 4.0 6.0 6.0 5.0 6.0 4.0 4.0 6.0 3.0 6.0 1.0 6.0 5.0 6.0 6.0 9.0 6.0 0.0 4.0 6.0 4.0 4.0 6.0 6.0 6.0 9.0 6.0 5.0 5.0 2.0 5.0 6.0 6.0 </vector>

<vector>8.0 8.0 5.0 7.0 4.0 6.0 6.0 5.0 6.0 4.0 4.0 6.0 3.0 6.0 1.0 6.0 5.0 6.0 6.0 8.0 6.0 0.0 4.0 6.0 4.0 4.0 6.0 6.0 6.0 8.0 6.0 5.0 5.0 2.0 5.0 6.0 6.0 </vector>

<vector>7.0 7.0 5.0 7.0 4.0 6.0 6.0 5.0 6.0 4.0 4.0 6.0 3.0 6.0 1.0 6.0 5.0 6.0 6.0 7.0 6.0 0.0 4.0 6.0 4.0 4.0 6.0 6.0 6.0 7.0 6.0 5.0 5.0 2.0 5.0 6.0 6.0 </vector>

<vector>6.0 6.0 5.0 6.0 4.0 6.0 6.0 5.0 6.0 4.0 4.0 6.0 3.0 6.0 1.0 6.0 5.0 6.0 6.0 6.0 6.0 0.0 4.0 6.0 4.0 4.0 6.0 6.0 6.0 6.0 6.0 5.0 5.0 2.0 5.0 6.0 6.0 </vector>

<vector>5.0 5.0 6.0 5.0 4.0 5.0 5.0 7.0 5.0 4.0 4.0 5.0 3.0 5.0 1.0 5.0 6.0 5.0 5.0 5.0 5.0 0.0 4.0 5.0 4.0 4.0 5.0 5.0 5.0 5.0 5.0 8.0 8.0 2.0 6.0 5.0 5.0 </vector>

<vector>5.0 5.0 6.0 5.0 4.0 5.0 5.0 7.0 5.0 4.0 4.0 5.0 3.0 5.0 1.0 5.0 6.0 5.0 5.0 5.0 5.0 0.0 4.0 5.0 4.0 4.0 5.0 5.0 5.0 5.0 5.0 7.0 7.0 2.0 6.0 5.0 5.0 </vector>

<vector>5.0 5.0 8.0 5.0 4.0 5.0 5.0 6.0 5.0 4.0 4.0 5.0 3.0 5.0 1.0 5.0 7.0 5.0 5.0 5.0 5.0 0.0 4.0 5.0 4.0 4.0 5.0 5.0 5.0 5.0 5.0 6.0 6.0 2.0 8.0 5.0 5.0 </vector>

<vector>5.0 5.0 7.0 5.0 4.0 5.0 5.0 6.0 5.0 4.0 4.0 5.0 3.0 5.0 1.0 5.0 7.0 5.0 5.0 5.0 5.0 0.0 4.0 5.0 4.0 4.0 5.0 5.0 5.0 5.0 5.0 6.0 6.0 2.0 7.0 5.0 5.0 </vector>

<vector>5.0 5.0 6.0 5.0 4.0 5.0 5.0 6.0 5.0 4.0 4.0 5.0 3.0 5.0 1.0 5.0 6.0 5.0 5.0 5.0 5.0 0.0 4.0 5.0 4.0 4.0 5.0 5.0 5.0 5.0 5.0 6.0 6.0 2.0 6.0 5.0 5.0 </vector>

<vector>5.0 5.0 5.0 5.0 4.0 5.0 5.0 5.0 5.0 4.0 4.0 5.0 3.0 5.0 1.0 5.0 5.0 5.0 5.0 5.0 5.0 0.0 4.0 5.0 4.0 4.0 5.0 5.0 5.0 5.0 5.0 5.0 5.0 2.0 5.0 5.0 5.0 </vector>

<vector>4.0 4.0 4.0 4.0 9.0 4.0 4.0 4.0 4.0 8.0 5.0 4.0 3.0 4.0 1.0 4.0 4.0 4.0 4.0 4.0 4.0 0.0 7.0 4.0 9.0 6.0 4.0 4.0 4.0 4.0 4.0 4.0 4.0 2.0 4.0 4.0 4.0 </vector>

<vector>4.0 4.0 4.0 4.0 8.0 4.0 4.0 4.0 4.0 8.0 5.0 4.0 3.0 4.0 1.0 4.0 4.0 4.0 4.0 4.0 4.0 0.0 7.0 4.0 8.0 6.0 4.0 4.0 4.0 4.0 4.0 4.0 4.0 2.0 4.0 4.0 4.0 </vector>

<vector>4.0 4.0 4.0 4.0 7.0 4.0 4.0 4.0 4.0 7.0 5.0 4.0 3.0 4.0 1.0 4.0 4.0 4.0 4.0 4.0 4.0 0.0 7.0 4.0 7.0 6.0 4.0 4.0 4.0 4.0 4.0 4.0 4.0 2.0 4.0 4.0 4.0 </vector>

<vector>4.0 4.0 4.0 4.0 6.0 4.0 4.0 4.0 4.0 6.0 5.0 4.0 3.0 4.0 1.0 4.0 4.0 4.0 4.0 4.0 4.0 0.0 6.0 4.0 6.0 6.0 4.0 4.0 4.0 4.0 4.0 4.0 4.0 2.0 4.0 4.0 4.0 </vector>

<vector>4.0 4.0 4.0 4.0 5.0 4.0 4.0 4.0 4.0 5.0 5.0 4.0 3.0 4.0 1.0 4.0 4.0 4.0 4.0 4.0 4.0 0.0 5.0 4.0 5.0 5.0 4.0 4.0 4.0 4.0 4.0 4.0 4.0 2.0 4.0 4.0 4.0 </vector>

<vector>4.0 4.0 4.0 4.0 4.0 4.0 4.0 4.0 4.0 4.0 4.0 4.0 3.0 4.0 1.0 4.0 4.0 4.0 4.0 4.0 4.0 0.0 4.0 4.0 4.0 4.0 4.0 4.0 4.0 4.0 4.0 4.0 4.0 2.0 4.0 4.0 4.0 </vector>

<vector>3.0 3.0 3.0 3.0 3.0 3.0 3.0 3.0 3.0 3.0 3.0 3.0 3.0 3.0 1.0 3.0 3.0 3.0 3.0 3.0 3.0 0.0 3.0 3.0 3.0 3.0 3.0 3.0 3.0 3.0 3.0 3.0 3.0 2.0 3.0 3.0 3.0 </vector>

<vector>2.0 2.0 2.0 2.0 2.0 2.0 2.0 2.0 2.0 2.0 2.0 2.0 2.0 2.0 1.0 2.0 2.0 2.0 2.0 2.0 2.0 0.0 2.0 2.0 2.0 2.0 2.0 2.0 2.0 2.0 2.0 2.0 2.0 2.0 2.0 2.0 2.0 </vector>

<vector>1.0 1.0 1.0 1.0 1.0 1.0 1.0 1.0 1.0 1.0 1.0 1.0 1.0 1.0 1.0 1.0 1.0 1.0 1.0 1.0 1.0 0.0 1.0 1.0 1.0 1.0 1.0 1.0 1.0 1.0 1.0 1.0 1.0 1.0 1.0 1.0 1.0 </vector>

<vector>0.0 0.0 0.0 0.0 0.0 0.0 0.0 0.0 0.0 0.0 0.0 0.0 0.0 0.0 0.0 0.0 0.0 0.0 0.0 0.0 0.0 0.0 0.0 0.0 0.0 0.0 0.0 0.0 0.0 0.0 0.0 0.0 0.0 0.0 0.0 0.0 0.0 </vector>

</interntermcov>

<phylocov>

<vector>10.0 8.0 5.0 7.0 4.0 6.0 6.0 5.0 6.0 4.0 4.0 6.0 3.0 6.0 1.0 6.0 5.0 6.0 6.0 9.0 6.0 0.0 4.0 6.0 4.0 4.0 6.0 6.0 6.0 9.0 6.0 5.0 5.0 2.0 5.0 6.0 6.0 </vector>

<vector>8.0 9.0 5.0 7.0 4.0 6.0 6.0 5.0 6.0 4.0 4.0 6.0 3.0 6.0 1.0 6.0 5.0 6.0 6.0 8.0 6.0 0.0 4.0 6.0 4.0 4.0 6.0 6.0 6.0 8.0 6.0 5.0 5.0 2.0 5.0 6.0 6.0 </vector>

<vector>5.0 5.0 9.0 5.0 4.0 5.0 5.0 6.0 5.0 4.0 4.0 5.0 3.0 5.0 1.0 5.0 7.0 5.0 5.0 5.0 5.0 0.0 4.0 5.0 4.0 4.0 5.0 5.0 5.0 5.0 5.0 6.0 6.0 2.0 8.0 5.0 5.0 </vector>

<vector>7.0 7.0 5.0 8.0 4.0 6.0 6.0 5.0 6.0 4.0 4.0 6.0 3.0 6.0 1.0 6.0 5.0 6.0 6.0 7.0 6.0 0.0 4.0 6.0 4.0 4.0 6.0 6.0 6.0 7.0 6.0 5.0 5.0 2.0 5.0 6.0 6.0 </vector>

<vector>4.0 4.0 4.0 4.0 10.0 4.0 4.0 4.0 4.0 8.0 5.0 4.0 3.0 4.0 1.0 4.0 4.0 4.0 4.0 4.0 4.0 0.0 7.0 4.0 9.0 6.0 4.0 4.0 4.0 4.0 4.0 4.0 4.0 2.0 4.0 4.0 4.0 </vector>

<vector>6.0 6.0 5.0 6.0 4.0 15.0 13.0 5.0 7.0 4.0 4.0 14.0 3.0 13.0 1.0 8.0 5.0 11.0 9.0 6.0 8.0 0.0 4.0 14.0 4.0 4.0 10.0 8.0 13.0 6.0 12.0 5.0 5.0 2.0 5.0 13.0 8.0 </vector>

<vector>6.0 6.0 5.0 6.0 4.0 13.0 17.0 5.0 7.0 4.0 4.0 13.0 3.0 15.0 1.0 8.0 5.0 11.0 9.0 6.0 8.0 0.0 4.0 13.0 4.0 4.0 10.0 8.0 14.0 6.0 12.0 5.0 5.0 2.0 5.0 16.0 8.0 </vector>

<vector>5.0 5.0 6.0 5.0 4.0 5.0 5.0 8.0 5.0 4.0 4.0 5.0 3.0 5.0 1.0 5.0 6.0 5.0 5.0 5.0 5.0 0.0 4.0 5.0 4.0 4.0 5.0 5.0 5.0 5.0 5.0 7.0 7.0 2.0 6.0 5.0 5.0 </vector>

<vector>6.0 6.0 5.0 6.0 4.0 7.0 7.0 5.0 8.0 4.0 4.0 7.0 3.0 7.0 1.0 7.0 5.0 7.0 7.0 6.0 7.0 0.0 4.0 7.0 4.0 4.0 7.0 7.0 7.0 6.0 7.0 5.0 5.0 2.0 5.0 7.0 7.0 </vector>

<vector>4.0 4.0 4.0 4.0 8.0 4.0 4.0 4.0 4.0 9.0 5.0 4.0 3.0 4.0 1.0 4.0 4.0 4.0 4.0 4.0 4.0 0.0 7.0 4.0 8.0 6.0 4.0 4.0 4.0 4.0 4.0 4.0 4.0 2.0 4.0 4.0 4.0 </vector>

<vector>4.0 4.0 4.0 4.0 5.0 4.0 4.0 4.0 4.0 5.0 6.0 4.0 3.0 4.0 1.0 4.0 4.0 4.0 4.0 4.0 4.0 0.0 5.0 4.0 5.0 5.0 4.0 4.0 4.0 4.0 4.0 4.0 4.0 2.0 4.0 4.0 4.0 </vector>

<vector>6.0 6.0 5.0 6.0 4.0 14.0 13.0 5.0 7.0 4.0 4.0 16.0 3.0 13.0 1.0 8.0 5.0 11.0 9.0 6.0 8.0 0.0 4.0 15.0 4.0 4.0 10.0 8.0 13.0 6.0 12.0 5.0 5.0 2.0 5.0 13.0 8.0 </vector>

<vector>3.0 3.0 3.0 3.0 3.0 3.0 3.0 3.0 3.0 3.0 3.0 3.0 4.0 3.0 1.0 3.0 3.0 3.0 3.0 3.0 3.0 0.0 3.0 3.0 3.0 3.0 3.0 3.0 3.0 3.0 3.0 3.0 3.0 2.0 3.0 3.0 3.0 </vector>

<vector>6.0 6.0 5.0 6.0 4.0 13.0 15.0 5.0 7.0 4.0 4.0 13.0 3.0 16.0 1.0 8.0 5.0 11.0 9.0 6.0 8.0 0.0 4.0 13.0 4.0 4.0 10.0 8.0 14.0 6.0 12.0 5.0 5.0 2.0 5.0 15.0 8.0 </vector>

<vector>1.0 1.0 1.0 1.0 1.0 1.0 1.0 1.0 1.0 1.0 1.0 1.0 1.0 1.0 2.0 1.0 1.0 1.0 1.0 1.0 1.0 0.0 1.0 1.0 1.0 1.0 1.0 1.0 1.0 1.0 1.0 1.0 1.0 1.0 1.0 1.0 1.0 </vector>

<vector>6.0 6.0 5.0 6.0 4.0 8.0 8.0 5.0 7.0 4.0 4.0 8.0 3.0 8.0 1.0 11.0 5.0 8.0 8.0 6.0 9.0 0.0 4.0 8.0 4.0 4.0 8.0 10.0 8.0 6.0 8.0 5.0 5.0 2.0 5.0 8.0 10.0 </vector>

<vector>5.0 5.0 7.0 5.0 4.0 5.0 5.0 6.0 5.0 4.0 4.0 5.0 3.0 5.0 1.0 5.0 8.0 5.0 5.0 5.0 5.0 0.0 4.0 5.0 4.0 4.0 5.0 5.0 5.0 5.0 5.0 6.0 6.0 2.0 7.0 5.0 5.0 </vector>

<vector>6.0 6.0 5.0 6.0 4.0 11.0 11.0 5.0 7.0 4.0 4.0 11.0 3.0 11.0 1.0 8.0 5.0 12.0 9.0 6.0 8.0 0.0 4.0 11.0 4.0 4.0 10.0 8.0 11.0 6.0 11.0 5.0 5.0 2.0 5.0 11.0 8.0 </vector>

<vector>6.0 6.0 5.0 6.0 4.0 9.0 9.0 5.0 7.0 4.0 4.0 9.0 3.0 9.0 1.0 8.0 5.0 9.0 10.0 6.0 8.0 0.0 4.0 9.0 4.0 4.0 9.0 8.0 9.0 6.0 9.0 5.0 5.0 2.0 5.0 9.0 8.0 </vector>

<vector>9.0 8.0 5.0 7.0 4.0 6.0 6.0 5.0 6.0 4.0 4.0 6.0 3.0 6.0 1.0 6.0 5.0 6.0 6.0 11.0 6.0 0.0 4.0 6.0 4.0 4.0 6.0 6.0 6.0 10.0 6.0 5.0 5.0 2.0 5.0 6.0 6.0 </vector>

<vector>6.0 6.0 5.0 6.0 4.0 8.0 8.0 5.0 7.0 4.0 4.0 8.0 3.0 8.0 1.0 9.0 5.0 8.0 8.0 6.0 10.0 0.0 4.0 8.0 4.0 4.0 8.0 9.0 8.0 6.0 8.0 5.0 5.0 2.0 5.0 8.0 9.0 </vector>

<vector>0.0 0.0 0.0 0.0 0.0 0.0 0.0 0.0 0.0 0.0 0.0 0.0 0.0 0.0 0.0 0.0 0.0 0.0 0.0 0.0 0.0 1.0 0.0 0.0 0.0 0.0 0.0 0.0 0.0 0.0 0.0 0.0 0.0 0.0 0.0 0.0 0.0 </vector>

<vector>4.0 4.0 4.0 4.0 7.0 4.0 4.0 4.0 4.0 7.0 5.0 4.0 3.0 4.0 1.0 4.0 4.0 4.0 4.0 4.0 4.0 0.0 8.0 4.0 7.0 6.0 4.0 4.0 4.0 4.0 4.0 4.0 4.0 2.0 4.0 4.0 4.0 </vector>

<vector>6.0 6.0 5.0 6.0 4.0 14.0 13.0 5.0 7.0 4.0 4.0 15.0 3.0 13.0 1.0 8.0 5.0 11.0 9.0 6.0 8.0 0.0 4.0 16.0 4.0 4.0 10.0 8.0 13.0 6.0 12.0 5.0 5.0 2.0 5.0 13.0 8.0 </vector>

<vector>4.0 4.0 4.0 4.0 9.0 4.0 4.0 4.0 4.0 8.0 5.0 4.0 3.0 4.0 1.0 4.0 4.0 4.0 4.0 4.0 4.0 0.0 7.0 4.0 10.0 6.0 4.0 4.0 4.0 4.0 4.0 4.0 4.0 2.0 4.0 4.0 4.0 </vector>

<vector>4.0 4.0 4.0 4.0 6.0 4.0 4.0 4.0 4.0 6.0 5.0 4.0 3.0 4.0 1.0 4.0 4.0 4.0 4.0 4.0 4.0 0.0 6.0 4.0 6.0 7.0 4.0 4.0 4.0 4.0 4.0 4.0 4.0 2.0 4.0 4.0 4.0 </vector>

<vector>6.0 6.0 5.0 6.0 4.0 10.0 10.0 5.0 7.0 4.0 4.0 10.0 3.0 10.0 1.0 8.0 5.0 10.0 9.0 6.0 8.0 0.0 4.0 10.0 4.0 4.0 11.0 8.0 10.0 6.0 10.0 5.0 5.0 2.0 5.0 10.0 8.0 </vector>

<vector>6.0 6.0 5.0 6.0 4.0 8.0 8.0 5.0 7.0 4.0 4.0 8.0 3.0 8.0 1.0 10.0 5.0 8.0 8.0 6.0 9.0 0.0 4.0 8.0 4.0 4.0 8.0 12.0 8.0 6.0 8.0 5.0 5.0 2.0 5.0 8.0 11.0 </vector>

<vector>6.0 6.0 5.0 6.0 4.0 13.0 14.0 5.0 7.0 4.0 4.0 13.0 3.0 14.0 1.0 8.0 5.0 11.0 9.0 6.0 8.0 0.0 4.0 13.0 4.0 4.0 10.0 8.0 15.0 6.0 12.0 5.0 5.0 2.0 5.0 14.0 8.0 </vector>

<vector>9.0 8.0 5.0 7.0 4.0 6.0 6.0 5.0 6.0 4.0 4.0 6.0 3.0 6.0 1.0 6.0 5.0 6.0 6.0 10.0 6.0 0.0 4.0 6.0 4.0 4.0 6.0 6.0 6.0 11.0 6.0 5.0 5.0 2.0 5.0 6.0 6.0 </vector>

<vector>6.0 6.0 5.0 6.0 4.0 12.0 12.0 5.0 7.0 4.0 4.0 12.0 3.0 12.0 1.0 8.0 5.0 11.0 9.0 6.0 8.0 0.0 4.0 12.0 4.0 4.0 10.0 8.0 12.0 6.0 13.0 5.0 5.0 2.0 5.0 12.0 8.0 </vector>

<vector>5.0 5.0 6.0 5.0 4.0 5.0 5.0 7.0 5.0 4.0 4.0 5.0 3.0 5.0 1.0 5.0 6.0 5.0 5.0 5.0 5.0 0.0 4.0 5.0 4.0 4.0 5.0 5.0 5.0 5.0 5.0 9.0 8.0 2.0 6.0 5.0 5.0 </vector>

<vector>5.0 5.0 6.0 5.0 4.0 5.0 5.0 7.0 5.0 4.0 4.0 5.0 3.0 5.0 1.0 5.0 6.0 5.0 5.0 5.0 5.0 0.0 4.0 5.0 4.0 4.0 5.0 5.0 5.0 5.0 5.0 8.0 9.0 2.0 6.0 5.0 5.0 </vector>

<vector>2.0 2.0 2.0 2.0 2.0 2.0 2.0 2.0 2.0 2.0 2.0 2.0 2.0 2.0 1.0 2.0 2.0 2.0 2.0 2.0 2.0 0.0 2.0 2.0 2.0 2.0 2.0 2.0 2.0 2.0 2.0 2.0 2.0 3.0 2.0 2.0 2.0 </vector>

<vector>5.0 5.0 8.0 5.0 4.0 5.0 5.0 6.0 5.0 4.0 4.0 5.0 3.0 5.0 1.0 5.0 7.0 5.0 5.0 5.0 5.0 0.0 4.0 5.0 4.0 4.0 5.0 5.0 5.0 5.0 5.0 6.0 6.0 2.0 9.0 5.0 5.0 </vector>

<vector>6.0 6.0 5.0 6.0 4.0 13.0 16.0 5.0 7.0 4.0 4.0 13.0 3.0 15.0 1.0 8.0 5.0 11.0 9.0 6.0 8.0 0.0 4.0 13.0 4.0 4.0 10.0 8.0 14.0 6.0 12.0 5.0 5.0 2.0 5.0 17.0 8.0 </vector>

<vector>6.0 6.0 5.0 6.0 4.0 8.0 8.0 5.0 7.0 4.0 4.0 8.0 3.0 8.0 1.0 10.0 5.0 8.0 8.0 6.0 9.0 0.0 4.0 8.0 4.0 4.0 8.0 11.0 8.0 6.0 8.0 5.0 5.0 2.0 5.0 8.0 12.0 </vector>

</phylocov>

<nodelabels>Acrocanthosaurus Aerosteon Afrovenator Allosaurus Aucasaurus Avimimus Bambiraptor Baryonyx Bicentenaria Carnotaurus Ceratosaurus Citipati Dilophosaurus Dromaeosaurus Eodromaeus Eotyrannus Eustreptospondylus Falcarius Gallimimus Giganotosaurus Guanlong Herrerasaurus Ilokelesia Ingenia Majungasaurus Masiakasaurus Ornitholestes Qianzhousaurus Saurornithoides Shaochilong Shuvuuia Spinosaurus_I Spinosaurus_II Tawa Torvosaurus Tsaagan Tyrannosaurus Node 35 Node 34 Node 33 Node 32 Node 31 Node 30 Node 29 Node 28 Node 27 Node 26 Node 25 Node 24 Node 23 Node 22 Node 21 Node 20 Node 19 Node 18 Node 17 Node 16 Node 15 Node 14 Node 13 Node 12 Node 11 Node 10 Node 9 Node 8 Node 7 Node 6 Node 5 Node 4 Node 3 Node 2 Node 1 Root</nodelabels>

<ancestpheno>

<vector>-0.22479043947343566 0.08061763374691532 0.02837697696408298 -0.054929576435869536 0.014483737036494665 0.024152355825533413 0.007907462549127421 -0.010613717133051752 -0.02569797590706322 0.015775536577523175 0.019593818355908742 -3.419787323170713E-4 0.007122766504630857 0.003459522520639322 -0.0028026507510841587 6.831733459415849E-4 </vector>

<vector>-0.1263386190468225 0.11811122870411418 -0.005150765304154657 -0.024175879700899995 -0.03357765362821859 0.03531748220737367 0.03405724302725459 -0.002657618759672925 -0.018560722698294892 -0.0055472814904339845 0.02755252278188498 -0.003891089770348591 0.0017203525810108745 0.012523904830405521 0.011415688736942493 0.004202915305636528 </vector>

<vector>-0.0684650945009794 0.17168916371484072 -0.08955440116704022 9.95870260293517E-4 -0.020415839319733932 0.007192998887797588 -0.001549800708286659 -0.014096860057717719 -0.02217652247791188 -0.02170127848850278 0.019922045003915968 -2.028917173227847E-4 -0.004708314758983395 0.007608126903704595 0.007514088538000336 0.00219211817256992 </vector>

<vector>0.14371371365135144 0.025426726884557327 0.0359099149433739 0.040622018370336986 0.05451765001145015 0.018103310580680557 -0.011473289768329755 0.026429859858209424 0.00282808648620427 -0.029059862919636897 -0.01305502726610002 -0.023933858727436672 -0.02038497753102662 -0.001218029732529993 -0.0012412763410464666 0.00816581856960019 </vector>

<vector>0.06480382742090064 0.027528845522506895 0.0044961937558520015 0.017299208065075875 -0.001046268472252837 0.0030569654761614627 -0.017726871083443344 -0.009763301463822205 0.0027513785662878504 -0.015155960783002999 -0.0034313999295697666 -0.0026972666461029388 -0.018691442149816617 -0.015417576923419347 0.004536279329812818 0.005825308286798387 </vector>

<vector>-0.022369500847844448 0.1267359342825734 -0.052128787399418494 0.01728931837878694 -0.01771020315462727 0.008325072832287159 -0.016770436794320127 -0.00997702783643455 -0.0037658279261243662 -0.014155344905608233 0.0015329687121368417 -0.0027428833981867906 -0.014389871997763815 -0.005653155104321615 0.005665424556119783 0.002896608684229307 </vector>

<vector>-0.06344723546345377 0.1809897936103716 -0.07132815478706754 0.03357287681099158 -0.031668501671897935 0.014725254132902836 -0.031034638591229697 -0.006070921987763561 0.008127660133251422 -0.0056087954453191355 -0.01189173893793619 -0.005328491831134648 -0.019769859084491224 -0.009150015293250172 0.004945905800546636 6.723995933193439E-4 </vector>

<vector>-0.013097761338424163 0.1505300586518779 0.016915991809163693 0.11465560085788988 0.014946796973239335 0.006254576800739492 -0.01688186185901008 -4.055938439160266E-5 -0.00813920258747257 0.015218954085273212 -7.74567268173168E-4 0.0033346507510679125 -0.011462477819050354 -0.001959594718421661 0.006897719254475751 -0.003125103488562213 </vector>

<vector>0.011511185971287537 0.08745472797632924 -0.006422452891408165 0.09556306681073957 0.04803163501073372 -0.01629410983682845 0.008552982096244364 0.0021906972534102447 -0.022054457982394065 0.0030037668534407297 -0.023573706207678277 0.015411061703710125 0.008027645560908977 -0.003942849471848905 -0.003606720014390207 0.0014434324984879416 </vector>

<vector>-0.058246913447256965 0.08018624984659252 0.04926009906783061 0.055694008036569424 0.03998887019738093 -0.08615206652974537 0.041735215751427586 -0.009325515915645462 -0.0014083731363084386 -0.013644655841686058 -0.020931675586195554 0.009462805004328376 0.006939129641778069 0.008039132946853161 -0.005818109140466135 0.003867717657898442 </vector>

<vector>0.02971977583069113 0.06886905110700618 0.0549696454022525 0.03614712874071355 -0.03300553389409357 0.016905783654506124 -0.00698078729242391 -0.0069386260608226375 0.006537728325248306 0.006270358513644027 2.670491017723077E-4 0.017390955417207095 -2.9066101422409174E-4 -9.084382374328253E-4 -0.019139674237392434 -1.0305449367874198E-4 </vector>

<vector>0.03934166806837697 0.022712856766703496 0.007498772105187313 0.019981871338911716 -0.02997509021909551 0.0013996667074195925 -0.005941399180164227 -0.022983649398765238 -0.009693503646363698 0.01909281827747361 -0.01081836420382119 -0.001991277331531229 0.006482335074326417 0.006485978591213791 -0.009553121249104212 0.00593967992313873 </vector>

<vector>0.012462263479505499 -0.026216341406443487 0.022077374246444965 0.04987301920203234 -0.05428538452177885 -0.04840171170178123 -0.026986930009308813 -0.022900519573163452 -0.005294857755612623 0.010969739089276313 -0.0015725736769654566 -0.011292437639069557 0.007057428957960021 0.010794243019856244 -0.01651708377100207 0.003792677267286373 </vector>

<vector>-0.010291755511696504 -0.011796590870120603 0.0024560560957861397 0.0582455391264681 0.006330536205505 -0.06008360159738503 0.010267049494616438 -0.007219556672181312 -0.003439126964611341 0.002044611619968279 -0.003374652141986449 -0.002224408172775503 0.0032057594587374377 0.01037815997128158 -0.011085359173118666 0.0045811694265250075 </vector>

<vector>0.014909383432663757 -0.08935968105051206 -0.06396930502691636 0.06916959014080361 0.0332881229409127 -0.04569702656062831 0.016052862741730758 0.01056736547226515 -0.003614150001912903 0.008808751612314604 0.012380292837201646 -0.0048435918835853 -0.004379280223525801 0.012301103947135242 -0.010920884607887609 0.006083113354390166 </vector>

<vector>-0.10495379538123953 -0.010385483340219853 -0.02440232390535528 -0.04503906252325144 -0.02360708804678386 0.039074401752819635 -0.016510186040877877 0.05307082366591517 0.008475776158724704 0.005478226854700669 -0.022296656175521172 -0.003109338995987563 0.0038125525462292097 0.010369292272155193 -0.0015275749924166335 -0.005945487267432751 </vector>

<vector>-0.05635408958883237 -0.02252380200396547 -0.03851704111898994 0.01446335710554426 -0.04952335400598482 -0.00683050306538047 -0.008351962330515248 0.05144711851201195 0.016969406738950008 0.015455927836881489 -0.0050489746095469384 0.013733955571289666 0.003941135312274387 0.012595406192272496 0.0034738897176570943 0.004992666840208744 </vector>

<vector>0.009965946080818404 -0.02624139056824066 -0.047413554645042814 0.02907043437946926 -0.05913499255616933 -0.01449353898794795 -6.596325501512894E-4 0.01959548763844146 0.003739138402635998 0.010185407165824082 0.014058039644708597 0.01049086665871958 0.010458177965858031 -0.001584333952640806 -0.006682882620026592 0.010192785889469381 </vector>

<vector>-0.012567876854784707 -0.03350824550500651 -0.050068181312393246 0.022868465066443265 0.019689339160393032 -0.01738061095548139 -0.0027022051325499875 0.03358275475323244 0.015335679640680177 -9.582227497748462E-4 0.023805235847314945 0.019182381093138107 0.015045603204605127 -0.011276238685058764 -0.003737242092734754 0.00845014001813475 </vector>

<vector>-0.011884188753179314 -0.0691766236350396 -0.03938844938416689 0.03281191353726443 0.023663806785205993 -0.024447794802928812 0.0011249103480816328 0.01653945639718141 0.005411542032442324 0.0027840125402082932 0.015429099880503606 0.005591314507022568 0.0038936680006018444 -7.495949205264607E-4 -0.004737530335029402 0.005839093240708795 </vector>

<vector>-0.1472812366749499 -0.17396710678729133 -0.060380291143025365 0.041930346369468796 0.012639077592230694 -0.006559488011591671 0.021505901000129474 -0.028614494740268524 0.006166206138588404 -0.01814048452512662 0.0020642473099105503 -0.009582075439492976 0.0037446052856990614 -0.005570996038329573 0.010302561465744292 -0.00677290578566605 </vector>

<vector>-0.16218897168289725 -0.13053982398879976 -0.012171559164416994 0.05963209116616397 0.024060542191068596 0.04135211385192156 0.011851987392136724 -0.0321057698548892 0.03810655435103387 -0.010726215062694782 0.007816435928464833 -0.006200554768323742 0.006648414500188864 -0.004734705466643005 6.458523607501699E-4 -0.0017846451133382984 </vector>

<vector>-0.03750875615022681 -0.030527161561909502 5.931761268232732E-4 -0.06116534335355297 0.06676625116455032 -0.014410486261534555 -0.013445867173392984 -0.041371498088576306 0.025458275338094263 0.02869719020856415 -0.0073024411369083456 0.0037548494145606577 -0.01502299440564919 0.00201036090474095 9.778205902381716E-4 -0.0021263092499696004 </vector>

<vector>-0.1064255757979182 -0.0665686901561404 0.033432486148160134 -0.056167595501554306 0.01518133907435612 0.0026110797903031967 -0.025535151856837888 -0.011707880768188152 0.008188298604890017 0.01489603295531329 2.487330019380063E-4 0.005112443248534758 -0.006321197842644597 -0.0011496040564212516 0.0011910394837204453 0.004297022924518707 </vector>

<vector>-0.10220287343941081 -0.09392348616484697 0.005711021723517413 0.0032873936897187313 0.019085279839912636 0.011232482249849247 -0.00788636367654576 -0.012781800552343625 0.01693598313816128 0.0015571088835679013 0.006055646629132366 4.490142639085766E-4 4.4729922609007197E-4 -0.003052653182240044 7.608091800015634E-4 0.001832134720260609 </vector>

<vector>-0.03799407283741771 -0.08466194434960062 -0.004127861813190986 0.006397685404546372 0.01801395825431297 -0.010265746892676765 -0.009975926564936021 0.005468248966046469 0.004513096458559939 5.015087580853123E-4 0.01010177095699424 0.002435154311514689 0.0010146810207260527 -0.003273650023655956 4.4553569553404393E-4 0.0029840263496013677 </vector>

<vector>0.2253325339695928 0.020723108268106083 0.0018379219302967387 -0.09169951457107457 -0.01056054525028794 -0.0038917396839153566 0.05638345219084892 -5.55618761706944E-4 0.00814581747264008 0.012328591546050705 -0.0011492313560484552 -0.01930129858757987 -0.001071114633080978 -0.012376960691496755 -0.003247891691335934 -0.0016351326095153645 </vector>

<vector>0.2544988490006931 0.01571724290555035 0.013651567530963743 -0.08518310777162447 0.006283213775124932 0.01157520525116151 6.785393975790484E-4 0.0029559435385105247 0.014874898876934804 -0.002042733612410383 0.009077764925693618 4.478984087256839E-4 0.007831140937012074 0.002702280101220427 -0.003309038807073275 -0.006520706656371116 </vector>

<vector>0.20858862141218135 -0.009920470389240384 0.038733821152526146 -0.07586689601086487 -0.01385188252998143 -0.009190722750755064 -0.005536305951569068 -0.00854563344346624 0.0013677135731094674 -0.018596713152792006 0.0137667479994058 0.017531284955315394 -0.002791122665977846 0.002241343943771944 0.0017363590052064098 -0.007151721772680598 </vector>

<vector>0.1785282539421228 -0.015040481695961574 0.015525209849003754 -0.05725704146235582 0.014879870519039565 -0.021239638992866797 -0.009817140333500039 -0.0018701194013859375 -0.010369551315251573 -2.778795963782211E-4 0.029252220903521687 0.008093764286721162 0.0010588873627210699 -1.6156157975440524E-4 0.006278224848006934 -0.009092074949141493 </vector>

<vector>0.10837094635504603 -0.09892764464663234 0.024908949979856668 -0.027716229796960583 -0.008920558335760297 -0.016649575649207885 -0.02402445651871937 0.011631828374561413 -0.005913007874995656 -0.00583533231603795 0.01707889558798221 0.009325071154775696 -0.00511510440145754 -0.006145794369970478 0.006889549008260864 -0.0020398390752057564 </vector>

<vector>1.048436803376962E-4 -0.09088572324891557 0.02129384222107613 -0.01690625101334431 0.011292788137819906 -0.01758192812495055 -0.023166326366343425 0.01264709105330181 -0.008808235794924086 -0.002836595149520415 0.008820566361346743 0.0012651341636131345 -0.0012969241645137825 -0.0060187019682011915 0.0053133282416300606 0.0012808510878347338 </vector>

<vector>-0.07006234247661572 -0.08906758075051414 0.04310043849656303 -0.02940020864761838 0.024784964494906234 -0.025830461832967267 -0.035498596015374764 0.020841195819297727 -0.02502479596833646 -0.00317596189060868 -7.189674609362588E-4 -0.007964822975451012 2.0965088719019156E-4 -0.008636661510977247 0.008604900021095275 0.0028983659891085277 </vector>

<vector>-0.018150208982557287 -0.07519123307220424 0.023741702305752066 0.004662699614385231 0.0043667525172809 -0.008781567010593183 -0.03708213876744636 6.632104952525742E-4 -0.018985668606684387 -0.022421669828553394 -0.007232506558400475 -0.01417712131084908 0.011399037994170455 -0.0015085690572176234 0.007374496003842659 -0.003477319807334572 </vector>

<vector>0.015149537206350358 -0.06551954556065373 0.015096042468525223 0.023928344397406126 -0.03457249648838211 0.029540621695441888 0.03084206789778811 8.993786370360829E-4 -0.01853030888412343 -0.013018244608643313 -0.016997086313989267 -1.2094994192577216E-4 0.014456605236920547 -0.005431318596160503 0.006911979378354667 -0.006002746953162292 </vector>

<vector>0.0687506152670854 -0.06780877353889055 0.002346725864722529 0.012604462445719616 -0.07099652233233048 0.017167293359963077 0.03078482165377484 0.0048799120354635845 -0.0035234339769869413 -0.0016195655204028904 -0.02821089137483553 0.016829804281393825 0.01614290022384642 0.004277801209855904 0.01508648273060263 -0.010188390923727789 </vector>

</ancestpheno>

<taxapheno>

<vector>-0.07407441946607675 -0.030944532103437762 -0.04373524480657211 0.059358699460411515 -0.06582798141499999 -0.04507237196101295 -0.007886068400516408 0.08167504423167864 0.03869330565548954 0.030704149490119986 -0.00690830729782867 0.03382033905113749 -0.002447324575263387 0.029001260257303374 0.018632126765414186 0.010730701898589698 </vector>

<vector>0.09881980468606923 -0.02269212419574982 -0.053655441503745745 0.04987948096641691 -0.14757096282291407 -0.01926950294298207 0.009075269812611732 -0.026243410349920563 -0.021087671171722038 0.01605851641036596 0.023417857696357774 -0.0014437366882686757 0.012387795380694924 -0.006072169365135733 -0.019785295485002505 0.01713555081006478 </vector>

<vector>0.008730157034063265 -0.05183966319343974 0.0010874020888086717 -0.05405682845630833 0.12105963230246182 0.06104752481002845 0.002801885581973901 -0.08686572113770126 0.025035799103707037 0.03424722174948665 -0.004719301591433925 0.01783113325439433 -0.03907779206887204 0.011906277778962035 8.692777724355095E-4 0.0036694165954075236 </vector>

<vector>-0.03578538789199292 -0.005106722311740984 -0.06340253990797007 0.00672304728259361 0.09453920325214317 -0.013200499075567206 -0.008571893195579876 0.06461332022407414 0.036856358486962434 -0.015844087955356728 0.04192856801673233 0.04146496211367286 0.030784963647356365 -0.03149478718200867 2.086866768514291E-4 0.009318540924226258 </vector>

<vector>0.23003457104822445 -0.006939861187613066 0.0390332443991234 -0.0563631632703632 -0.006962316279664112 0.025724272184645187 0.07268483845039306 -0.028066347678863204 -0.04770481155725164 0.01488494043320513 -0.03856762748052459 -0.006739793564446761 -0.012218841187808544 -0.007736117972830532 -0.007838079796914034 -0.003268049965417125 </vector>

<vector>0.07306726945919192 -0.06957612459960834 0.02970745372359915 -0.0060137125538983534 -0.03994625227357998 -0.01725748698448425 -0.024936886687680355 -0.04574273641324161 0.009191877138783517 -0.0022526745237638635 0.0012278587652539003 0.018584942187315386 -0.021299476920659012 -0.03938154593340692 0.009184689774364917 0.006413497606565453 </vector>

<vector>-0.2768864185441912 0.12756755319245058 0.03966557387249317 0.012269075812092523 0.037402657948281307 -0.00823420103561992 -0.009888814937066155 3.9157392285671063E-4 -0.044288003411199144 0.025665631701620815 0.023763804038861424 0.011527729407772956 7.957936347641389E-4 -0.03045495781798602 -0.0035670094431652574 -0.008754704352106197 </vector>

<vector>-0.23708280493433173 -0.123728879014261 0.018154591926256798 0.13367853343930125 0.040457269141063144 0.11938334731750691 0.02193642485282636 -0.054921014272055695 0.09121747377635185 -0.015595269546525552 0.01532941384635133 -0.009468603129386584 0.015753338988777985 -0.005580467179359068 -0.009125813563495566 -4.1316427460940525E-4 </vector>

<vector>0.06690409456286403 -0.18710582864637443 -0.15497552179236934 0.11645131775867337 0.06987002583202886 -0.05255968328157101 0.03676662838249399 0.022382196691795014 -0.0128148650735696 0.021597630676767596 0.025086430773087753 -0.01789768198500252 -0.020237268129916403 0.02727474679065089 -0.016939764315515166 0.007829077395937006 </vector>

<vector>0.3295753916203034 0.03634909083778483 3.8295951006786337E-4 -0.08798291273293585 0.043262069105644285 0.04780807818815472 -0.04881152804654255 0.017969082820704217 0.0351111655850546 1.3992076951048656E-4 0.014615778133723237 0.003113708858441852 0.027355660110095513 0.0182424570513862 -0.008415583735090274 -0.01077526558691734 </vector>

<vector>0.146479741442677 -0.19085672899501943 0.03790779786948987 -0.008985396915182547 -0.05293433366413976 -0.011127159829806051 -0.03908990285631423 0.024118513471768153 0.0014387634851884554 -0.01439152220221497 0.013163899499077855 0.018616315013993058 -0.015107276402579448 -0.01225711956195578 0.00907709393514565 0.0016917066356894224 </vector>

<vector>0.0770731382755203 -0.012967649981257894 0.02629359094901625 0.055799665857359904 0.044756878573067066 0.0021144543625427146 0.04112263816668279 0.08987861053574124 -0.003978390447392363 -0.030495537272670063 -0.014099173770890261 -0.03417086925206333 -0.04239315761536837 0.005842675501249598 -0.034585854741453585 -0.005663646817595826 </vector>

<vector>-0.19214166212762812 -0.10112578593042224 0.08426577096286021 -0.07595707454389798 0.058695352829619074 -0.05112789036335766 -0.046247322912334785 0.0492132859093383 -0.04728048350340061 0.015730379306248148 -0.003744962185755328 -0.010982481779117002 -0.009473161168085824 -0.018382713507512794 0.013126875817812887 0.01089156668682543 </vector>

<vector>-0.08576032316605335 0.10202688865058637 0.045725128290492634 -0.01859393292712598 -0.09480085860141728 0.07460709190878978 0.09581406724092371 0.016737720911750836 -0.0078076697099093584 -0.010716102560322727 0.043141704985829664 -0.011128398861404844 0.0027466059973859126 0.026504065066871994 0.029535628423911685 0.009733454398397713 </vector>

<vector>-0.005151794665477164 -0.05355863007086613 0.019199699235100964 0.05451787113211316 -0.03708771965009674 0.0802361387369558 0.09882352080703598 -0.0028449866196078753 -0.03308182406869897 -0.015013498476973582 -0.01554786100873189 -0.0030155327963219247 0.015827877492744884 -0.019063187941119775 -0.0017250405993813021 -0.004342530128424534 </vector>

<vector>0.07584296489493268 0.025485860599546425 -0.054550703333135636 -0.026074533926013985 -0.002634352241412657 0.03569492816953406 0.01614351976124044 -0.03911180256230955 -0.030323381508726443 0.040038357229500596 -0.031149568036270836 -0.012072349772731007 0.012680237279243887 0.009572130991218102 0.00699739426108187 0.014129416995808462 </vector>

<vector>-0.17956509780411745 -0.07525542274166432 0.09399326059413925 -0.1106248368408316 -0.04030751378139304 0.01101124338259498 -0.055273224720574804 0.019029656336355068 -0.017829362661585295 0.014433799773808044 0.001992993513589768 0.011133466067135316 -0.004387898348374415 -0.002406519891764364 0.0018344886809213388 0.013185243303265124 </vector>

<vector>0.012642765476891561 0.18314565436893343 0.12849858310596618 0.21483085895193532 0.02847725758088266 0.020332586106144517 -0.028163929082044646 0.003758546581178761 -0.010490809913275043 0.04826189084769837 0.03314174334109498 -7.861761937124831E-5 -0.022645219933568276 0.007214080609834004 0.01935397197727083 -0.011491142557494099 </vector>

<vector>-0.1759601708013632 0.16490061243356904 0.15174669399911275 0.01327341817249831 0.06560443937590511 -0.18207848815502287 0.10638561566342228 -0.022947688328165225 0.02126846553807994 -0.04598234599846712 -0.03584666840892194 0.015201761482050971 0.00958398390568851 0.017682088341126483 -0.0027622482338895407 0.00557855104868227 </vector>

<vector>-0.07783994098814478 0.0660248346509535 -0.03453243673152475 -0.0773749390207566 -0.00153807326560272 0.038861571616182575 -0.03476431725251634 0.04668189097911473 0.01905265980654937 0.013610169577122898 -0.04752772383232513 -0.008353370343960408 0.02128850990063457 0.007795224066715971 -0.016598394763029864 -0.003421289037317068 </vector>

<vector>0.008336877881833767 -0.08956529011591412 0.05627729453836107 0.07139164714071376 -0.1392115995517448 -0.086521200215378 -0.08528644034237812 -0.038498352648543774 -0.0027519426558626995 0.011771787370387452 0.009475295314911026 -0.029661627412901355 0.011484192340816927 0.015518590497073458 -0.028912770890783424 8.571824521952211E-4 </vector>

<vector>0.12235169332782041 -0.07009800151712757 -0.010402590739079978 0.001280580494033067 -0.10742054817627888 0.004793965024484311 0.030727575409761548 0.008860445433891096 0.011483440930149538 0.009779113567837483 -0.0394246964356818 0.033780558504713384 0.017829195210772284 0.013986921015872329 0.0232609860828506 -0.014374034894293279 </vector>

<vector>0.192738761293728 -0.030438172377309412 0.08702468607761071 -0.08516053879861503 -0.06271873188410876 -0.017907734510559718 -0.007470316918785922 -0.026722724467523325 -4.0220684235497007E-4 -0.05346952624958742 0.0029702581690017344 0.044052192170499604 -0.017263396297666156 0.004183313309849809 0.002239890974685667 -0.005842383712529326 </vector>

<vector>0.28926417525762765 0.06171898511242188 0.0769399601252512 0.048767181188567967 0.11984233993353623 0.049138511903336526 -0.057815636388227586 -8.257294972913868E-4 0.009711271339717708 -0.041528090703237125 -0.021634508097840795 -0.03493344028414215 -7.033282789350274E-5 0.0059208122245789025 0.02632574638850126 0.024335794239597513 </vector>

<vector>0.19146418185985895 0.053391943086381094 -0.04717104613919758 -0.13355227267123798 -0.031002533246324085 -0.048974696487553045 0.09578697872457434 0.023443547855231676 0.057267365098236746 0.02414356781735743 0.02604216848668502 -0.05161200060701826 0.0011743563515544818 -0.03209704420288015 0.001403443529979606 0.004883358793241935 </vector>

<vector>0.2186251940591412 0.0637266699479892 -0.017067141585372158 -0.06818799857924404 0.0674120524228605 -0.03787861857863757 1.0934146978832174E-4 -0.00869655313525321 -0.026563359643868377 0.02359840667969551 0.05691101912317677 -0.002575063249927425 0.011082889155598937 0.003419765686935368 0.010208766530553491 -0.018084663999538144 </vector>

<vector>0.10587823269954236 0.031647875430519 -0.0854434495512192 0.11633959153775596 0.08915923786158222 0.031015160218520556 8.055923963159965E-4 0.015938167060267877 -0.05661579822340085 0.007437002316734735 -0.04901487576866615 0.033435729355734585 0.02860628485999974 -0.017908086643978353 -0.011899770157180392 0.0035876833261276 </vector>

<vector>0.014298391082011199 0.03425081368167789 0.0032681909869804173 -0.03512942310119519 0.003590271666078787 0.035669600395445804 9.96514225669249E-4 -0.0052514095166889905 0.010093153747269655 -0.01675087951464606 0.021331120722155924 0.06938210653352386 -0.01884982659377732 0.00826246179654069 -0.036039881159537365 8.075527815541977E-4 </vector>

<vector>-0.056687163608274554 0.270220328157836 -0.21138365079754826 0.00987417210299021 -0.009959661176353465 -0.02206355837626869 -0.021936208357794813 -0.02965593357704594 -0.0442030168093165 -0.04540120906946575 0.03068064351772592 0.006025298016567732 -0.0014554248601967468 0.015953630985029298 0.0054611523209381965 -5.231694721561653E-4 </vector>

<vector>-0.18066735556674549 -0.07465748266764956 -1.57493865551895E-4 -0.07220560565455038 -0.019759836868760382 0.08519213670765745 -0.006414278539600735 0.06108346150661763 -0.010594738069324918 -0.012631416849901421 -0.01431327008469181 -0.014708602215290587 -0.013791987574220224 0.010717246557477675 0.00854178006812209 -0.01940783960518954 </vector>

<vector>-0.15487444420409524 0.26570338789666087 -0.17877166877094797 -0.03122628880370694 -0.09224209883430382 0.0295961127656815 -0.059451617120358596 -0.008195178742465156 0.03628801091335119 -0.01788999551562196 -0.036433618257772665 -0.016577242846284177 -0.03345722743665864 -0.01983729605700732 0.002274573591044028 0.00224569358429098 </vector>

<vector>-0.15936410477827845 -0.1827938789522531 -0.0376513914412589 -0.01571536898396652 0.005167616012918643 -2.0649222850904932E-4 0.03559295966354 -0.023653448596465766 -0.029459051991426337 -0.052549967525288545 0.02856744562343018 -0.029029769565627542 0.0073336286867819915 -0.010341342598880872 0.0032940404243412044 -0.00821357404230914 </vector>

<vector>-0.12029063356367553 -0.2085676174208213 -0.1313179228234012 0.08187431692620316 0.00868907457270658 -0.060824085658187456 0.01707275594471195 -0.030084265769451272 0.009851116056157107 0.00885472901260369 -0.030191139622164107 0.006484098015473201 -0.002748227329872872 -0.0016369400494643128 0.026967791612141218 -0.01032049820135074 </vector>

<vector>4.621783225930707E-4 -0.07098657290544473 0.013028625952167562 0.019459963093367377 0.022887789545317984 -0.030054860894253858 -0.10658988818475114 -0.019750942970576037 -0.013401900967593215 -0.05107080298640798 -0.003981465900275998 -0.03444559101517 0.019530857858400807 0.009542272935484757 0.006606608612078112 -0.007327578457949905 </vector>

<vector>-0.014830849686827092 0.02682686866385204 -0.03274035985649997 -0.07327160610280259 0.06405778211683448 -0.10689006338493508 -0.01760433524531467 -0.025540892359840223 0.04315072830568552 0.036948315920892934 -0.017436754821229618 -0.011679028259246443 3.3000669456985154E-4 -0.004725591008317481 8.731445145580699E-4 -0.014345367269834867 </vector>

<vector>-0.27114628082930314 -0.0038258806558260972 0.05061612232390655 -0.152881925418815 0.039626206789424905 0.04537378630484819 -4.4604044280733266E-4 -0.029575106562340185 -0.014245201611694906 0.02720825952138406 0.007465128246978923 -0.008662575834373635 0.018852153298119116 0.02830962054949915 -0.016256631547031025 0.006601309084294323 </vector>

<vector>0.035519268341681814 0.14964348287263898 0.15414197311459021 0.1235889379844202 -0.07263178312926172 0.013648083860652475 -0.015997476922776328 0.007419180732986299 0.01921353487483891 0.01646913677810481 -0.009711609213018118 -0.015217962950370539 0.011495508476779477 -0.017473755100053237 -0.011826020303535672 -0.007056396185729412 </vector>

</taxapheno>

<treelengthvec>0.0 0.0 0.0 0.0 0.0 0.0 0.0 0.0 0.0 0.0 0.0 0.0 0.0 0.0 0.0 0.0 </treelengthvec>

</phylogeny_content>

<morphojset>

<morphojset_contents visiblename="BranchDiffs, unweighted: PC scores, CovMatrix, Mandibular_articulation, Procrustes coordinates" uniqueid="155" alignmentmethod="0" dimension="1" hasprocrustesfit="false" hasrawdata="false" linkedoutlinenumber="0" linkedwireframenumber="0" nincl="72" nlmk="10" nobs="72" objsymmetry="false" procsumsquare="0.0" procrustesfitfromparentset="false">

<datavector>

<datamatrix visiblename="PC scores" matrixtype="9" iscoordinates="false" issymmetrycomponent="false" isasymmetrycomponent="false">

<data>

<vector>-0.01772032987724438 -0.008420730099472293 -0.005218203687582169 0.04489534235486725 -0.016304627409015164 -0.03824186889563248 4.658939299988403E-4 0.03022792571966669 0.021723898916539534 0.015248221653238497 -0.001859332688281732 0.02008638347984782 -0.006388459887537774 0.016405854065030877 0.015158237047757091 0.0057380350583809536 </vector>

<vector>0.08885385860525083 0.003549266372490839 -0.00624188685870293 0.020809046586947648 -0.08843597026674474 -0.004775963955034119 0.009734902362763021 -0.04583889798836202 -0.024826809574358035 0.005873109244541876 0.009359818051649177 -0.011934603346988256 0.001929617414836892 -0.004487835412494927 -0.013102412864975913 0.006942764920595397 </vector>

<vector>0.046238913184290076 -0.021312501631530235 4.942259619853985E-4 0.007108514897244636 0.0542933811379115 0.07545801107156301 0.016247752755366884 -0.045494223049124954 -4.224762343872264E-4 0.005550031540922498 0.0025831395454744208 0.014076283839833673 -0.02405479766322285 0.009895916874221086 -1.0854281780266206E-4 0.005795725845377124 </vector>

<vector>-0.023217511037208212 0.028401523193265523 -0.013334358595576822 -0.016145417783849654 0.07484986409175014 0.004180111879914183 -0.005869688063029888 0.031030565470841695 0.021520678846282257 -0.014885865205581881 0.018123332169417385 0.02228258102053475 0.01573936044275124 -0.020218548496949904 0.0039459287695861826 8.684009060915075E-4 </vector>

<vector>0.004702037078631649 -0.02766296945571915 0.03719532246882666 0.035336351300711374 0.003598228970623829 0.029616011868560544 0.016301386259544137 -0.02751072891715626 -0.05585062902989172 0.0025563488871544263 -0.03741839612447614 0.01256150502313311 -0.011147726554727566 0.004640842718666223 -0.0045901881055781 -0.0016329173559017605 </vector>

<vector>0.008263442038291283 -0.09710497012211523 0.02521125996774715 -0.023312920618974227 -0.03889998380132714 -0.020314452460645713 -0.007210015604237011 -0.0359794349494194 0.0064404985724956666 0.012903286259239135 0.004659258694823667 0.021282208833418324 -0.0026080347708423955 -0.02396396900998757 0.004648410444552099 5.881893197670664E-4 </vector>

<vector>-0.052095979070755555 0.04694991944553527 0.011288596908410192 0.06719865224796207 0.02291892091178664 -0.03238655686115333 -0.017796277486193578 0.011005291055908462 -0.018590027504135923 0.00989009512409764 0.004169985682952682 0.011869708140090028 -0.006326972869866718 -0.03391448033862534 -7.643586920810987E-4 -0.009437877698047782 </vector>

<vector>-0.07489383325143448 0.0068109449745387685 0.03032615109067379 0.07404644227313728 0.01639672694999455 0.07803123346558535 0.010084437460689637 -0.022815244417166494 0.05311091942531798 -0.00486905448383077 0.0075129779178864965 -0.003268048361062842 0.009104924488589121 -8.457617127160628E-4 -0.009771665924245736 0.0013714808387288932 </vector>

<vector>0.05199471113020027 -0.09774614759586236 -0.09100621676545298 0.047281727617869765 0.036581902891116166 -0.006862656720942703 0.020713765640763233 0.011814831219529864 -0.009200715071656696 0.012788879064452993 0.012706137935886107 -0.01305409010141722 -0.015857987906390602 0.014973642843515649 -0.006018879707627557 0.0017459640415468398 </vector>

<vector>0.07507654261961033 0.02063184793223448 -0.01326860802089588 -0.0027998049613113807 0.03697885533051935 0.036232872936993205 -0.0494900674441216 0.015013139282193692 0.020236266708119798 0.0021826543819208695 0.005538013208029619 0.002665810449716168 0.01952451917308344 0.015540176950165773 -0.005106544928016999 -0.004254558930546225 </vector>

<vector>0.03810879508763097 -0.09192908434838709 0.012998847889633201 0.018730832881778035 -0.04401377532837946 0.005522415819401834 -0.015065446337594861 0.01248668509720674 0.007351771360184112 -0.008556189886177021 -0.0039149960889043565 0.009291243859217362 -0.009992172001121909 -0.006111325191985301 0.0021875449268847855 0.003731545710895179 </vector>

<vector>-0.06664057537583114 -0.03839437686581522 -0.009616323994357651 0.015177647487022917 -0.009760771438383081 -0.015988856218137844 0.05259592793501254 0.06344875067753182 -0.006806476933596633 -0.0014356743530331666 -0.0010441465047902401 -0.010237010524626656 -0.022008180084341748 0.007060705233779591 -0.03334457840040712 -0.013829465387196016 </vector>

<vector>-0.1220793196510124 -0.01205820517990809 0.04116533246629718 -0.0465568658962796 0.03391038833471284 -0.02529742853039039 -0.01074872689696002 0.028372090090040575 -0.022255687535064152 0.018906341196856826 -0.003025994724819069 -0.003017658803665991 -0.009682812055276015 -0.009746051996535547 0.004521975796717611 0.007993200697716902 </vector>

<vector>0.04057829588076915 -0.01608434005352781 0.05087589359464729 0.0055819467737740144 -0.06122320497319869 0.03928960970141611 0.061756824213669126 0.01939533967142376 0.010753052988385534 -0.005168821069888743 0.015589182203944684 -0.007237309091056254 0.0010262534163750382 0.013980160236466473 0.01811993968696919 0.005530539092761185 </vector>

<vector>-0.020301331871827522 0.011960915489787602 0.004103656766575741 0.030589526734707037 -0.0025152231617146317 0.05069551704151391 0.06798145290924787 -0.003744365256643958 -0.014551515184575543 -0.0019952538683302695 0.0014492253052573768 -0.0028945828543961525 0.0013712722558243372 -0.01363186934495927 -0.00863701997773597 0.001660216824737758 </vector>

<vector>0.036501296826555715 0.0027730038328429296 -0.06204947543832295 -0.0460564052649257 0.02734073797768285 0.034295261462114465 0.022084918941404668 -0.01612815316354431 -0.020629877862362745 0.020945538952026985 -0.020331203832449646 -0.010081072441199778 0.00619790220491747 0.003086152400004311 0.016550515510186083 0.008189737072669732 </vector>

<vector>-0.07313952200619925 -0.00868673258552391 0.06056077444597911 -0.0544572413392773 -0.05548885285574916 0.008400163592291784 -0.029738072863736916 0.03073753710454322 -0.02601766126647531 -4.62233181505246E-4 0.0017442605116517618 0.006021022818600558 0.0019332994942701824 -0.0012569158353431123 6.434491972008936E-4 0.008888220378746417 </vector>

<vector>0.025740526815315724 0.03261559571705552 0.11158259129680248 0.10017525809404544 0.013530460607643326 0.014078009305405025 -0.011282067223034566 0.003799105965570364 -0.002351607325802472 0.03304293676242516 0.03391631060926815 -0.003413268370439161 -0.011182742114517922 0.009173675328255665 0.01245625272279508 -0.008366039068931887 </vector>

<vector>-0.11771325735410623 0.08471436258697652 0.10248659493128215 -0.042420589864071115 0.025615569178524186 -0.0959264216252775 0.0646503999119947 -0.013622172412519763 0.022676838674388376 -0.03233769015678106 -0.014914992822726389 0.005738956477722596 0.0026448542639104407 0.009642955394273322 0.0030558609065765947 0.0017108333907838282 </vector>

<vector>0.027113854393094744 0.07641031799117336 -0.01013011282616947 -0.032335876497505155 0.02206901478118114 -2.1283013663705996E-4 -0.01825413121163846 -0.006388932686800443 0.010576883647824664 0.00813194272242223 -0.02523106765680396 -0.005244031347972845 0.01747595735440536 -0.0025740682054392217 -0.01507081977061323 0.0025241982301156827 </vector>

<vector>-0.004125385597671732 -0.06334894870947064 0.034199920291916104 0.021518627938681423 -0.08492621502996596 -0.03811948851359677 -0.05829951033306931 -0.015597833075380322 0.0025429150997499234 8.020482811111392E-4 0.011047868991876483 -0.0183691897738318 0.004426763382856906 0.004724347477217214 -0.012395687119781354 -0.002935494815091152 </vector>

<vector>0.05360107806073501 -0.002289227978237024 -0.012749316603802506 -0.01132388195168655 -0.03642402584394841 -0.012373328335478766 -5.724624401329259E-5 0.003980533398427512 0.01500687490713648 0.011398679088240373 -0.01121380506084627 0.01695075422331956 0.0016862949869258653 0.009709119806016426 0.008174503352247968 -0.00418564397056549 </vector>

<vector>-0.01584986011845335 -0.02051770198806903 0.04829086492508457 -0.009293642787750162 -0.04886684935412733 -0.008717011759804653 -0.0019340109672168534 -0.018177091024057086 -0.0017699204154644375 -0.03487281309679541 -0.010796489830404066 0.02652090721518421 -0.01447227363168831 0.001941969366077865 5.035319694792571E-4 0.0013093380601512722 </vector>

<vector>0.14555046160627622 0.036292258227864554 0.0410300451818773 0.00814516281823098 0.06532468992208608 0.03103520132265597 -0.046342346619897834 -0.02725558935550081 0.0068831848535134375 -0.012468227783600228 -0.008579480831740774 -0.010999581556705478 0.020314644703133117 0.0071388419571088955 0.027567022729547724 0.016169975669997325 </vector>

<vector>-0.03386835210973385 0.03266883481827501 -0.04900896806949432 -0.04185275810016341 -0.020441987996036144 -0.04508295680363769 0.03940352653372542 0.02399916661693862 0.049121547625596666 0.011814976271306725 0.027191399842733475 -0.03231070201943839 0.00224547098463546 -0.019720083511383393 0.00465133522131554 0.006518491402757299 </vector>

<vector>0.04009694011701839 0.07876715164395078 -0.032592351434375916 -0.01093095711688822 0.05253218190382093 -0.016638979585770774 0.00992648180328836 -0.006826433733867273 -0.016193808328616804 0.02387628627607373 0.02765879821965508 -0.010668827536648587 0.010024001792877867 0.003581327266689773 0.003930541682546557 -0.008992589050396651 </vector>

<vector>0.09436704672825483 -0.055806852545810234 -0.07902099665981104 0.020776524727016382 0.0411276028508485 0.047309270055349004 -0.007747389699928367 0.013747469806857633 -0.03456134024100678 0.0044332354632940045 -0.025441169560987874 0.01802466765202446 0.020578639299090762 -0.013965237172129448 -0.008293050142790186 0.0021442508276396585 </vector>

<vector>-0.015421384748679932 -0.03461823742532829 -0.05170145441527208 -0.07127655184190873 0.036595805560172355 0.01876381674093968 0.00797730151809316 0.001687216544133647 0.0035554254220213485 -0.02302123802829009 0.021064071620383616 0.05199115111631677 -0.018559165579553227 0.009170900033973514 -0.01690020692214493 9.106072752329396E-4 </vector>

<vector>0.011777930892704846 0.09853116444299526 -0.12182924963050804 0.008878301842696694 0.010456178143380467 -0.02925655726406628 -0.020386407649508154 -0.015559073519328222 -0.02202649433140462 -0.02369993058096297 0.010758598513809954 0.006228189733890516 0.0032528898987866485 0.008345504081324702 -0.002052936217062139 -0.0027152876447260853 </vector>

<vector>-0.07571356018550596 -0.0642719993274297 0.024244830039803385 -0.02716654313129894 0.0038472511780234774 0.046117734954837813 0.010095907501277143 0.008012637840702455 -0.019070514228049625 -0.01810964370460209 0.007983386090829362 -0.011599263219303024 -0.017604540120449434 3.4795428532248193E-4 0.010069355060538724 -0.01346235233775679 </vector>

<vector>-0.09142720874064147 0.08471359428628927 -0.10744351398388043 -0.06479916561469852 -0.06057359716240588 0.014870858632778665 -0.0284169785291289 -0.002124256754701595 0.028160350780099767 -0.012281200070302825 -0.024541879319836476 -0.011248751015149529 -0.013687368352167415 -0.010687280763757148 -0.002671332209502608 0.0015732939909716363 </vector>

<vector>-0.012082868103328537 -0.008826772164961777 0.022728899701766468 -0.057645715353435316 -0.007471461579312051 0.0063529957830826215 0.014087058663410527 0.004961046143802758 -0.03562525813001474 -0.034409483000161924 0.02650319831351963 -0.019447694126134566 0.00358902340108293 -0.004770346560551299 -0.007008521041403088 -0.0014406682566430904 </vector>

<vector>0.02699060311127438 -0.03460051063352998 -0.07093763168037584 0.03994397055673436 -0.003950003019524114 -0.05426459764659579 -0.004433145055417524 -0.0014697710291827482 0.0036849099175687025 0.02699521353773031 -0.03225538693207466 0.016066173454966175 -0.006492832615571933 0.00393405598886526 0.016665230146396925 -0.003547592415684691 </vector>

<vector>0.018612387305150357 0.004204660166759508 -0.010713076353584504 0.014797263478982145 0.018521037028037084 -0.021273293883660675 -0.06950774941730478 -0.02041415346582861 0.005583767639091172 -0.028649133157854587 0.003251040658124477 -0.020268469704320922 0.008131819864230352 0.01105084199270238 -7.678873917645471E-4 -0.003850258650615333 </vector>

<vector>0.022677906463399716 0.05735403022576154 -0.03333353598332325 -0.01210626274924962 -0.0027084690477158363 -0.09247957712340052 -0.004158468071921686 0.015830605728736083 0.017692452967591255 0.008251125712328784 -0.010134313684321272 -0.0154338776738071 0.015353001100219041 -0.0067359519130584305 -1.0467607568010167E-4 -0.012219058019865267 </vector>

<vector>-0.04635584135586748 -0.08444351440274141 0.02223914535982357 -0.09795234898294547 0.02514246975293024 0.021221430479314775 -0.008353502991934754 -0.018961389429288433 0.011452774295368316 0.011432722943860885 -0.01212869010892982 -0.008320597102056564 0.011729386793488259 0.02485009802885983 -0.013453980795946867 0.005918135738352738 </vector>

<vector>0.005799492510990684 0.0807744317656328 0.09917232771233772 0.08744180924370665 -0.03962624923516815 -0.003257699793853649 -0.009016689630352417 0.014357806793808936 0.012675806549590603 0.010198778264460782 -0.009978658314790426 -0.032608918367577636 0.011786169491003569 -0.016565316862620413 0.007313653933856763 -0.00695334169205067 </vector>

<vector>-0.09845182042661316 -0.03749359495719887 0.03352774226823763 -0.03075369673496954 0.04806139066471325 -0.011165126381840258 -0.026149780478127167 -0.007956098373378826 -0.007137253208768329 0.02132281806795716 -0.007958704425976237 0.0035491110380315195 0.005402413923619983 -0.009064382309766198 -0.014218339488026652 -0.003519741959694943 </vector>

<vector>-0.0578735245458431 -0.05357793501072654 0.08440363586288556 -0.025171749961193514 -0.013161814308484655 0.028124483319576083 0.035607043735541244 0.011439241298044793 0.0036157997796169884 0.016153996998068798 0.007630477777969012 -0.003688198053025806 0.00642866733999427 0.004915777926700926 0.0039016001989421577 0.002010797133066608 </vector>

<vector>-0.04609559365313495 0.044953229432267316 -0.03742561376762173 -0.016293448118493425 -0.002705636165106662 -0.0011320739444895707 0.015220636086033468 -0.004119832221283169 -0.018410694551787513 -0.007545933582894548 0.018389076291779126 0.002539991680864006 0.00968155723878042 0.013261282008026211 0.001848663981880553 -7.044905116593871E-4 </vector>

<vector>0.0789098862304508 -0.002102118637949568 0.0314137211875219 0.02332281030526111 0.055563918483702984 0.015046345104519094 0.006253581315113589 0.036193161322031625 7.670791991641959E-5 -0.013903902136633898 -0.009623627336530254 -0.021236592081333733 -0.001693535381210004 0.014199547190889353 -0.005777555670859285 0.0023405102828018033 </vector>

<vector>0.08717332826874509 -0.09920708876006651 0.056624981155270496 9.88968628893444E-6 0.016663934682374433 -0.005268107356125696 -9.564342891232175E-4 2.1372637261234465E-4 0.006517206492412216 -0.001000615877394766 -0.004964368641706608 4.561675208385185E-5 -0.004301570152052802 -0.009764421819097733 -0.0011291452263069643 0.0029286996025690796 </vector>

<vector>0.04107773461560932 -0.05425385932779819 0.019199367387649047 -0.01628355843220464 0.013958298517270665 -0.006400181300615678 0.01426420179690957 -0.0039061058486709885 -0.011893488059375788 -0.008546549460289097 0.013424707650073031 0.0025856084329478578 0.005379987086727409 0.003496860188928557 7.195187555731463E-4 0.002224209090909963 </vector>

<vector>-0.0503494741250296 0.03045973495849369 -0.08824414659623123 -0.0810827240468983 -0.04661529864513727 0.008470677332163345 -0.014152776732219617 -0.006030362603371958 0.016266862720723994 -0.020827749530592347 -0.011117171669763021 -0.008663142582202561 -0.00830738126544087 -0.007190420574828511 -0.001951813453929115 0.003797503081881557 </vector>

<vector>-0.0246089473097117 0.06307533067554867 0.023338444700571857 0.019092534047150306 -0.03308483803749439 0.02254868663756794 -0.025434843955254444 -0.0022312566378018474 0.013915255394921494 0.012215187231832483 0.02279913893950511 -0.012076410952642213 -0.019490123379959333 0.0019832547534272436 0.010504439268865958 -0.004568535987050155 </vector>

<vector>0.0697580994185445 0.007268478129736716 -0.055682551959238775 0.03986905877417015 0.008042764813352796 0.06985795669291692 -0.03318223365518322 0.011516213169055706 -0.020646084846085627 0.01664842269512679 -0.002642030621482723 0.005948256699381749 0.0010885159191309085 -0.011981982418702065 0.0022113891260759286 -0.0024242851594105004 </vector>

<vector>-0.04795515793556046 0.09198284071671312 0.04680404297204447 -0.0025515310898986746 0.03365833399187593 -0.02606846493236034 0.03146816625681115 -0.00210595924346415 0.0020307538283029026 -0.015689267461654336 -0.017557023444209105 0.011687213177103879 0.003733370183040631 -0.00233902702442842 0.005267250032652531 -7.134517686265655E-4 </vector>

<vector>-0.009621892237685836 0.046156194340302686 0.04747087329706519 0.01616525740180183 -0.003030443674998058 0.015506116947086531 -0.001039388112259683 0.0160450233379426 0.016231231971612005 -0.012822459763829584 0.011085413305593497 0.019382232748738324 -0.006772996088550509 -0.007394416828646617 -0.009586552988288222 -0.006042734416817472 </vector>

<vector>0.026879404588871468 0.04892919817314698 -0.014578602141257653 -0.029891147863120623 0.02431029430268334 0.04980137840920082 0.021045530829144586 -8.312982560178608E-5 -0.004398645890751075 0.008123079188197298 -0.009245790526855733 0.009301160307538327 -5.750938836336043E-4 -0.004308264428642452 0.006963962521897857 0.002147002655852357 </vector>

<vector>0.022754018991202003 -0.014419750536322884 0.019621318150658824 -0.008372519924435759 -0.06061592072728385 0.0116818898956038 -0.037253979503925255 -0.01568096290098214 -0.0018557307910012817 0.008925127469308035 0.0018020784650209926 -0.009068029466294054 0.0038516694992225837 4.1608304857466277E-4 -0.005431724597883403 -7.884921592386344E-4 </vector>

<vector>-0.02520113894436026 0.07756309018039145 0.0664253611227025 -0.010924051014335509 -0.0269575867354077 -0.014386575036756721 -0.005785813247114319 -0.017786922144446462 1.7502303730156186E-4 -0.006764139992346325 -0.015754944979188095 0.002619183710809797 0.007585039682263239 -0.0019229439758536616 -1.6447456523105766E-4 -0.0015019439278651583 </vector>

<vector>0.02679357218584307 -0.020183057415472466 -0.02458085564274947 0.036357676603539175 0.009624316155706705 -0.021249231757699497 0.014927952393649125 -0.005972090924916261 -0.009025692034355227 0.00602473907210631 -0.00304880704330196 -0.010434906390607868 -0.008272948224127646 0.013050698867661702 -0.006183354272858207 2.4402011368137091E-4 </vector>

<vector>-0.04859970579240716 0.012138318663745616 0.014114717213634657 -0.0595024196287957 0.025916265959200963 0.0459049048182001 -0.008158223710362629 0.0016237051539032266 -0.008493630580225304 -0.00997770098218082 -0.017247681565974234 -0.016843294567277227 -1.285827660451773E-4 -0.0022261139201173036 -0.005001464710073728 -0.010938154107641495 </vector>

<vector>-0.06632003566965078 0.00371758856427519 0.008896513526052877 -0.014607077273925 0.009611638550184508 0.007663035922567479 -0.007692329780363959 0.031851630873570486 0.013230268336314011 0.005270520671057407 -0.019107014254255535 0.0032430889125700853 -0.0065170426535836445 0.014179740144913303 0.010156772337683686 -0.005200119049260637 </vector>

<vector>0.02253382293560311 0.007266854936765849 0.002654626667350432 0.006201969313025996 -0.07882433171656236 0.0028870719675334403 0.002042572582398698 -0.01398726711479098 -0.01159654123804418 0.011143629915598929 -0.009747196202606348 -0.008691514434418526 -0.004587425238747096 0.009691904732417957 -0.002945640527291838 0.0017426458713346304 </vector>

<vector>-6.836881016053925E-4 0.03566837813003309 -0.010679731928226355 -0.009943448470821167 -0.003974467624812961 0.0070671838474474225 -0.0038271154806316203 0.01704329835605103 0.009924137608237852 -0.0037422352899831395 0.00837613596681134 0.013591066586115539 0.011151935204003283 -0.010526643764532304 0.001000288242294648 0.0026110467774259558 </vector>

<vector>0.026109884084238394 0.015485320714561018 -0.03526058757097591 0.02641422813271806 0.005649848530893023 -0.014182047910252047 0.011100836913017654 0.011071207431134942 8.984455738823855E-4 0.002282503782122981 0.005327328923509365 0.003156160195507879 0.0028789869798757917 0.0025240551031294953 -0.005183066030563446 0.0028550668911074273 </vector>

<vector>0.014907735007947343 -0.04342728279849156 -0.04820873197860837 -0.017701744796695174 -0.011421464598837902 -0.04791160186351323 0.00965391360799275 0.003491275114620676 -0.031940348212445464 -0.007414269462431839 -0.0057521886185542825 -0.0033815206711692337 -0.002903809214489803 -8.362905716865678E-4 0.009656709104994122 -0.004988260672327751 </vector>

<vector>-0.05998609824348644 -0.036616337823952794 -0.01788258088793441 0.05634469747644524 0.00497526235115596 0.03011963160207231 0.019738351068682485 -0.019323969302545575 0.02117057121287259 -0.012283323946262683 0.0017607892993324673 -0.0066495690322323185 0.006201115274098792 -0.001682052284402961 -1.1495681925139355E-4 -0.0036167798335989074 </vector>

<vector>0.06891681964769139 0.036041528594230904 -0.03283931002133686 -0.004997747851998663 0.051584912090194196 -0.01702156605183775 0.012089284683444904 -0.029663617320388153 0.017269976733204247 0.01380115725325086 -0.007551174138846352 -0.0013575938339741005 -0.008701796563004592 0.0031599649611622014 -2.1321889348227367E-4 -0.006423332174488308 </vector>

<vector>-0.004222702358507385 0.027354796008706564 0.02772146442464272 -0.05945498919127304 -0.0039039407655565156 -0.00862140245954605 -0.017648788180292127 0.0010739197841554728 -0.008747684533271263 0.01333892407174539 -0.005806913627194359 0.004663428984626182 -0.006768497068734669 0.0019030491258187924 4.3023030371888184E-4 0.0024648882042580984 </vector>

<vector>-0.0642088006019931 -0.009261541815246355 0.0098388835367084 -0.003110291714827641 0.001071321585599666 0.021498229142526012 0.0020895628883902603 -0.018250049518390092 0.012422886679601342 0.001055600125482589 -0.004046124327861875 -0.0019861400476061126 -5.673817946359808E-4 2.2099684141591198E-4 3.152734844675195E-4 -0.0011518916293407587 </vector>

<vector>-0.038098916517755405 0.006223778899314955 -0.025421704034267117 0.02330393641789068 0.006721170116493064 0.007316181232273785 0.013190399801407404 -0.007178842087255341 0.013321332253484024 0.0033381039076057274 0.0012812045956474977 0.0011700201479015546 0.002311605185239835 0.0027450519445452355 -0.004867792546096017 0.0017031752617666339 </vector>

<vector>-0.029166315031100287 0.005005865362555734 -0.011813645600667005 -0.006516406799450106 -0.016843759025412873 -0.015466944935076866 0.055704912793269876 -0.0035115623002174687 -0.006729081404294724 0.014371325158461087 -0.010226996281742073 -0.019749196996305556 -0.008902255570093052 -0.015079240792717183 6.114711573734083E-5 0.004885574046855751 </vector>

<vector>0.04591022758851174 0.025637713294790733 -0.025082253621562405 -0.009316211760759599 0.020135096305106362 0.020765928001916574 0.006214845349148117 0.011501576981976765 0.013507185303825336 0.01655397954038162 -0.004688983073712183 -0.01708338654658971 0.01062226360298992 4.6093615744848297E-4 -0.0050453978122796846 6.310151163094824E-4 </vector>

<vector>0.030060367470058552 0.005120011306721191 0.023208611303522392 -0.018609854548509046 -0.028731753049020994 0.012048916242111733 0.00428083438193097 -0.006675514042080302 0.01173726488836104 -0.018318833556413783 -0.015485472904115886 0.009437520668594232 -0.0038500100286989157 0.0024029055235263493 -0.004541865842800525 0.001940353176460895 </vector>

<vector>0.07015730758707676 0.08388716295067077 -0.009383740130852913 -0.02954081166539524 0.02380042885479986 -0.004590063343658912 0.01420731618521933 -0.013501947775947352 -0.004456543440255916 0.0055574527196597286 0.012173325315539475 -0.0012313068680545342 0.0061739917641786095 0.005984232790216073 -6.113241602539298E-4 -0.007052235873935737 </vector>

<vector>0.10826610267470833 -0.00804192139771677 0.0036151077587805378 -0.010809978783616274 -0.020213346473580203 9.323524757426649E-4 -8.581301523759438E-4 -0.0010152626787403966 0.0028952279199284297 -0.0029987371665175346 0.008258329226635469 0.008059936991162562 -0.003818180236943757 -1.2709240176928677E-4 0.0015762207666308037 -0.0033206901630404902 </vector>

<vector>0.07016718615695341 -0.001818142498401426 -0.0218065962754869 0.01249395763427407 -0.013492176357086327 0.008248533708016717 0.012332269649031339 -0.008194104765995917 0.016216560173412373 3.393667410882648E-4 0.009539533822283001 0.009229957139064146 -0.001506575051703974 0.0026179595427760554 -0.0032915717794652147 -0.001617514901273794 </vector>

<vector>-0.05191213349405843 -0.013876347678309905 0.019358736190810966 -0.03406290826200361 0.020418211977625333 -0.017048894822374084 0.0015835427520715928 0.020177985324045154 -0.006039127361652071 0.019245707937944716 0.006513539097464216 0.0062122983353980685 -0.011189387106980263 -0.0071280924537596236 0.001230404017252616 0.0063756857964431 </vector>

<vector>-0.033299746188907645 -0.009671687511550509 0.008645659837226843 -0.019265644783020897 0.03893924900566301 -0.03832218870603507 -0.06792420666523447 -2.361681417835087E-4 -4.553597225609579E-4 -0.009403425219910081 0.009764579755588792 -0.014056171368923308 -0.0030575672427500915 0.00392274953894288 4.6251662548799175E-4 0.00252542714582772 </vector>

<vector>-0.05360107806073504 0.002289227978236816 0.012749316603802694 0.01132388195168651 0.03642402584394837 0.01237332833547881 5.7246244013268305E-5 -0.003980533398427502 -0.015006874907136487 -0.011398679088240422 0.011213805060846262 -0.016950754223319597 -0.0016862949869258723 -0.009709119806016407 -0.008174503352247963 0.004185643970565497 </vector>

</data>

</datamatrix>

</datavector>

<idvec>Acrocanthosaurus - Node 19 Aerosteon - Node 18 Afrovenator - Node 13 Allosaurus - Node 17 Aucasaurus - Node 9 Avimimus - Node 31 Bambiraptor - Node 35 Baryonyx - Node 14 Bicentenaria - Node 21 Carnotaurus - Node 8 Ceratosaurus - Node 5 Citipati - Node 32 Dilophosaurus - Node 3 Dromaeosaurus - Node 34 Eodromaeus - Node 1 Eotyrannus - Node 24 Eustreptospondylus - Node 12 Falcarius - Node 28 Gallimimus - Node 26 Giganotosaurus - Node 20 Guanlong - Node 23 Herrerasaurus - Root Ilokelesia - Node 7 Ingenia - Node 32 Majungasaurus - Node 9 Masiakasaurus - Node 6 Ornitholestes - Node 27 Qianzhousaurus - Node 25 Saurornithoides - Node 33 Shaochilong - Node 20 Shuvuuia - Node 29 Spinosaurus_I - Node 15 Spinosaurus_II - Node 15 Tawa - Node 2 Torvosaurus - Node 13 Tsaagan - Node 35 Tyrannosaurus - Node 25 Node 35 - Node 34 Node 34 - Node 33 Node 33 - Node 30 Node 32 - Node 31 Node 31 - Node 30 Node 30 - Node 29 Node 29 - Node 28 Node 28 - Node 27 Node 27 - Node 26 Node 26 - Node 22 Node 25 - Node 24 Node 24 - Node 23 Node 23 - Node 22 Node 22 - Node 21 Node 21 - Node 16 Node 20 - Node 19 Node 19 - Node 18 Node 18 - Node 17 Node 17 - Node 16 Node 16 - Node 10 Node 15 - Node 14 Node 14 - Node 11 Node 13 - Node 12 Node 12 - Node 11 Node 11 - Node 10 Node 10 - Node 4 Node 9 - Node 8 Node 8 - Node 7 Node 7 - Node 6 Node 6 - Node 5 Node 5 - Node 4 Node 4 - Node 3 Node 3 - Node 2 Node 2 - Node 1 Node 1 - Root</idvec>

<meanconfig>0.13495631814332032 0.16154075134581164 0.047755353507686074 0.17203387836539474 -0.31079316962390124 0.08271234990638376 -0.51103806369261 -0.06715247516695218 -0.23736469910379962 -0.12718055089786032 -0.1250018957230973 -0.15702801365668548 0.03448588140856004 -0.2192626495455337 0.3667739636867369 -0.09217087296522046 0.4253854840073151 0.18539389683426025 0.17484082738978995 0.061113685780401716 </meanconfig>

<omitflag>false false false false false false false false false false false false false false false false false false false false false false false false false false false false false false false false false false false false false false false false false false false false false false false false false false false false false false false false false false false false false false false false false false false false false false false false</omitflag>

<uniquenumvec>156 157 158 159 160 161 162 163 164 165 166 167 168 169 170 171 172 173 174 175 176 177 178 179 180 181 182 183 184 185 186 187 188 189 190 191 192 193 194 195 196 197 198 199 200 201 202 203 204 205 206 207 208 209 210 211 212 213 214 215 216 217 218 219 220 221 222 223 224 225 226 227 </uniquenumvec>

</morphojset_contents>

</morphojset>

<morphojset>

<morphojset_contents visiblename="IndContrasts: PC scores, CovMatrix, Mandibular_articulation, Procrustes coordinates" uniqueid="228" alignmentmethod="0" dimension="1" hasprocrustesfit="false" hasrawdata="false" linkedoutlinenumber="0" linkedwireframenumber="0" nincl="36" nlmk="10" nobs="36" objsymmetry="false" procsumsquare="0.0" procrustesfitfromparentset="false">

<datavector>

<datamatrix visiblename="PC scores" matrixtype="9" iscoordinates="false" issymmetrycomponent="false" isasymmetrycomponent="false">

<data>

<vector>-0.004058890303142021 0.09290918807750248 -0.007743207067706245 0.11677939289012257 -0.0015722864638721273 -0.03790657137420759 -0.006677049878105784 0.0211896429807333 -0.02124346887827286 -0.001090802592201609 0.011524904176801139 0.014276701750948152 -0.01276777436150155 -0.04155283185721706 0.008972917840338187 -0.010858341232870545 </vector>

<vector>0.11906356541169122 0.02539691747380334 3.695312398732664E-4 0.03270585156237152 -0.08431599327882666 0.035441099926179075 0.0638663051020533 0.01981450751533589 0.013571820761871405 -0.023497650848184472 0.01740975448781039 -0.007944258536383982 -0.0044761202080394625 0.017441057784689955 0.02494875729344361 0.006836940454955527 </vector>

<vector>0.0647309807157205 0.11427063374341039 -0.15930715379654936 0.030483459667971734 0.019544823559880964 -0.04605144916823024 -0.047975195064124454 -0.021000233838558383 -0.01724813001957264 -0.030727326838644726 -8.993547317722484E-4 0.007522276636027005 -0.004361667702008893 2.978442351987726E-4 -0.005144648555251308 -0.0036792194214359 </vector>

<vector>-0.15004172115705358 -0.05281142613874614 -0.03581239108699307 0.004972717597893209 -0.05309343889650823 -0.03325102996600171 0.06995992485667357 0.06413765392040781 -0.00968005268181588 0.0078011933445568775 0.005328286001094491 5.392191479193613E-4 -0.029926756406193574 -5.525100692584887E-5 -0.04307100621192124 -0.02121280820334754 </vector>

<vector>-0.06963423148713613 -0.05942033069935279 -0.01385667178407603 -0.03687034621353154 -0.07731507139089899 -0.02712220413633157 -0.010492682401598206 -0.05709124032306514 0.004000557428776851 0.02135115451082318 0.012076548457546077 0.03360685092566399 -4.2837287744348175E-5 -0.028627018029726756 0.008420983469321363 -0.0018483994250395926 </vector>

<vector>0.11930932426601559 -0.12901775102569654 0.08424101984963579 0.01468701356194488 0.01730638801058236 -0.003680807129525614 -0.014565142683154559 0.0039021154363497856 0.022400745914211522 0.005905029369253148 -0.020995697106445783 -0.002247485222639761 -0.012559997178035211 -0.02065926394165883 -0.0026729149468584217 0.003247884776922244 </vector>

<vector>-0.09885218406935001 0.10903684664957267 -0.0848485931834029 -0.021156885581172333 -0.051211607412321974 0.015775610819818587 -0.03233701069351311 0.002939261204482325 0.029629882595211286 0.0018724225218531058 -0.029118426173888105 -0.00950318953065773 -0.013969811495849544 -0.01014952849526698 -0.002370443006672511 -0.0014564642379251232 </vector>

<vector>0.06672384391743998 -0.010726937992378342 0.15782529429651485 0.14357046770353551 0.055439435927010505 9.703713187082254E-5 0.0073659452704582275 0.008432009891402229 -0.017888433434496243 0.04137568806678068 0.03209772450044985 0.006657041082898061 0.0015186943836323651 0.01291028551308657 0.009634273738978717 -0.008984079644653404 </vector>

<vector>0.08292871000281456 -0.09764026014413912 -0.07217846149038018 -0.006290700051594425 0.05853700569207304 0.0066344483103536935 0.020689740184116 0.010725214786693145 -0.03528215638704111 -0.009496277511352413 -0.0385472658114004 0.02322734709427719 0.03222997266418962 -0.01061131947780732 -0.015642647828199092 0.005899962935705475 </vector>

<vector>-0.14251736376799579 0.045080895977268005 0.119028976155851 -0.06609282937465528 0.007785086622386238 -0.12915360888050256 0.07314186292543311 -0.019936942502834037 0.03466441370853126 -0.03663596895295852 -0.006574402752713771 -0.002403100926497377 5.456799004053071E-4 0.017943776609408175 -3.234585808990557E-4 0.0034820823849733467 </vector>

<vector>-0.015005426213040489 -0.08159493888414457 -0.10668387444569462 -0.11223082942245367 0.05389713182175787 0.015571563673764043 0.012016566380490031 -0.008959460287181547 -0.0064490833423100314 -0.02349009879173173 0.0219505248437042 0.059821282820914826 -0.021457392205768668 0.01819825348966937 -0.017121785209986293 0.005560651641671187 </vector>

<vector>0.03221357556589394 -0.042033809057787294 -0.08427835864155306 -0.04446433800116278 0.020166732284021497 0.00697983366893677 0.014953779301299576 -0.025421985347306875 -0.028445779057482187 0.025411575399506083 -0.02337512879174044 -0.02476343052192504 0.010345325803472827 0.008966813902242514 0.019562067798291605 0.010912285733301084 </vector>

<vector>-0.029230294051198764 -0.0878387110709751 0.035675914560079944 0.04300561125964468 -0.07679156991749116 -0.07305750922759746 -0.057038836099671156 -0.009590877541518182 0.00594172800229152 -0.007562942540864717 0.016025990883446667 -0.0206214234920179 0.0033160150441720673 0.007204911406793503 -0.01459766475450712 -0.0039509398380368575 </vector>

<vector>0.0635934662231906 -0.09572498704705691 -0.02449025586658672 -0.005225345689978317 -0.08474118808704127 0.033952111997305405 -0.06178343684825664 -0.012191747365808485 -0.003494456500437615 0.02213583958018624 0.01741764291872953 -0.01866294711745861 1.006108151286672E-4 0.0024785673086696017 -0.009619283347162801 -6.632962586251032E-5 </vector>

<vector>0.058220911944300444 -0.14202548583538685 -0.12598492002322845 0.040002197192497146 0.05091911773928013 0.011429414613915815 0.018603831199635962 0.0262440020245316 -0.005679142556801663 0.014930581392364485 0.024583163423556154 -0.01061602671520348 -0.017647819172361273 0.01101015684363182 -0.00341411810922304 0.0026626003911364143 </vector>

<vector>0.072709962140409 0.0994774205690219 -0.02430675520342954 -0.0036552706774583 0.012884732608971002 -0.03276065675238662 -0.020046504620803762 -0.010183448179733622 0.020963876082566352 0.018555603711641756 -0.023486165478358457 0.004493827552230441 0.02480565765186738 -0.0020661819179972063 -0.017776788103322914 0.011304198314324276 </vector>

<vector>0.034898408515101925 -0.01684115751788342 -0.016690678266770922 0.08484325933031578 -0.034898280465909724 -0.06773549803797453 0.00803422776914898 0.017577436882840084 0.021797165518376536 0.01910950041300322 0.015186642187138775 0.028682696590500024 -0.003918432474484654 0.012487850256738812 0.014331716912968036 0.014005896129098196 </vector>

<vector>0.12091272802450326 -0.0014877193245615376 -0.012698867451061364 0.02739950247469771 -0.0643830767931913 -0.010565788122277365 0.013670258815526484 -0.060033746382335086 -0.028525036254371448 -0.0015874733288516682 0.024764180273748604 -0.010619572173014877 0.00766341516629003 -0.01685347981469059 -0.018204112000428253 0.009465683110065142 </vector>

<vector>-0.03686479471151213 0.010303365168281696 -0.010896881862020995 -0.01617841925835184 0.1250182980293471 -2.990447998482025E-4 -0.005669764338169063 0.03315909073467001 0.024890661513478848 -0.0203356460961475 0.02094267075200626 0.022460042679959134 0.01431461831029711 -0.022183012473945336 0.005382259457506994 -0.0012037003911534803 </vector>

<vector>-0.025001645811301296 0.050320682023451704 0.012809086369958918 -0.0420016352297109 -0.012320675147230968 0.025672627153148303 -0.017016404707962875 0.020702146618169624 0.017104984914559508 -0.00883609125321138 0.010277864432688294 0.021672271739592536 0.017519546783846325 -0.021308482988381932 0.006524466450122229 0.002116634179341908 </vector>

<vector>-0.027629116460343092 0.01822478524765314 0.06623223951053539 -0.06900632868094625 -0.0024900472272934636 0.042863111373334706 0.01309576163853963 0.004547274431588956 -0.027796486396028997 -0.04341967731864571 0.041548594080087535 -0.025112096592757423 0.007128948756323047 -0.006154942068869921 -0.016739870001016472 0.0014898203603040793 </vector>

<vector>-0.061509738368879505 0.0455063576981694 0.06491476087088552 0.06362443167803702 0.02120555337096189 0.09480422174748161 -0.0027805483412645807 -0.017741741936477207 0.06389156968500197 0.0039543331621828305 0.010208629715873106 0.001141096917842766 0.008513255164068482 2.584682236864453E-4 -0.015341302813014338 0.005599680229691848 </vector>

<vector>0.016660147623923392 -0.05562563812871858 0.023919839863891974 0.013586899572827778 0.040306394806436746 0.11874980742877259 0.014429377125365907 -0.04336320228392947 -0.012809189279433197 -0.0019099620052248426 0.008992597418211293 0.02086683532020566 -0.0278655217372646 0.011760507203290225 -2.734199575962098E-6 0.012738375832722897 </vector>

<vector>-0.1116377310576433 -0.03968596829256879 0.06945610175934862 -0.029700503629257947 -0.08403197648619291 0.021460805353974842 -0.030276911159253118 0.04758150369447314 -0.03283875228307238 -0.01338526931266045 0.00826683999609671 0.005095955785952297 0.009477975022584661 -0.003792749354443045 6.092302074279324E-4 0.011715112100240118 </vector>

<vector>0.04987629754201814 0.05721752153902541 0.040789498147161145 -0.10357438846487767 -0.007941800701998583 -0.034651034272165274 -0.033440073937983 0.018244426638145805 -0.02675970144664042 0.0229172353218502 -0.006768759270895198 0.010118653038006362 -0.011600373936158462 0.003206612183794461 4.8763018696759236E-4 0.00543960925969442 </vector>

<vector>-0.08571250085484622 -0.023552003576681028 0.04297871354523864 -0.028163029355794237 -0.00437992714518808 0.0338918097735079 -0.008620244789149214 -0.02784682880551304 0.010902578397232403 -0.0011798849845725632 -0.008916531418380252 -0.004893516095859555 -0.0032857949700998553 -0.0022005954409491145 0.00524691910957035 -0.0038162947062006366 </vector>

<vector>0.02727338374809754 -0.042661027923360795 0.060955638407022096 0.054580942691108844 0.016999000438321157 0.05282014729575284 -0.016335680047796432 -0.03642299643036914 -0.07422653794900802 -0.006546838207813557 -0.04568602485949316 0.03172944188660906 -0.009470420801854576 0.017225776133253316 -0.006534743812940156 -0.005763916409471238 </vector>

<vector>0.07515217064141971 0.00829974549907301 0.002815603725238919 0.004411254157545015 0.039366874485856566 0.03758891326224911 -0.08414658732208985 0.0128265034964957 0.019182305966736077 -0.01225340431032181 0.013204727651607495 0.02042173990658054 0.020793811335638325 0.024133894768247337 -0.003287671836726868 -0.007325681831628282 </vector>

<vector>-0.05538536911631104 -0.03816427431430162 0.054837288437128796 0.0034805982422548634 -0.05028554405830634 -0.026011632145922448 -0.007366276232213327 -0.02268571369039849 -0.014500539198538488 -0.03805334825369196 -0.0020429245847166577 0.0333990349670307 -0.019515560267912298 7.469815228956264E-4 0.005318713899302352 1.8587465410626963E-4 </vector>

<vector>-0.005232522953141278 0.04359121122158765 -0.043335723207019616 0.01182966684544997 0.061182346719506515 -0.022323940679887293 0.0018620206048747064 0.002446853852727383 -0.02173737178684101 0.033061927774476314 0.03257067879112226 -0.016023875581263336 0.010043963206833173 -1.8568300031815768E-4 0.006967163507574073 -0.007498696907479197 </vector>

<vector>-0.04659144292248724 -0.14069279837053567 0.017416513028926377 0.04111307700391847 -0.0510005153716577 0.008002597761520729 -0.023516614471390293 0.021217482740250638 0.008999787381308532 -0.010844941336039013 -0.014591093164301967 0.006973832285813354 -0.01234892984265115 -0.009760529731507926 0.00196330017489915 0.00935746302582641 </vector>

<vector>0.13199382862660292 -0.01324736175607971 0.0289066275375494 -0.03296136070119536 -0.024245731692999057 -0.006618277271600411 -0.014121245470519806 0.006407467193076846 -0.011018059901979491 -0.005971845564676988 0.005820285973892341 0.005762033524227575 -0.005636978349474582 -0.002894374688458743 0.006297240135415346 -0.004590819888200428 </vector>

<vector>-0.149584965914994 -0.005101700469049594 0.0483904828157355 -0.041273676395964735 0.03491844125552609 -0.024040719083853457 -0.019975124733426868 0.02579398549508245 -0.031056737748852984 0.010775802929259988 -0.012332369075296555 -0.011985161260806131 -0.004045176917562117 -0.0086428716253083 0.006306487846055598 0.0065028664148889 </vector>

<vector>0.06409315937684289 0.016659257532808624 -0.026217558336786666 0.04364265783797653 -0.009341228907614334 0.004233077389413567 -0.04428759556020646 -0.03300560619865637 0.009555327100292395 -0.03711649625205581 -0.004611613978652871 -0.0187481222127184 0.016344832686844658 0.014017116807932661 -0.0017197504614096476 -0.008832303611022281 </vector>

<vector>0.020834368113765232 0.0170772848469258 -0.006129953923809986 0.03819367972432958 -0.04057388895773806 0.06970470670304527 0.11004205000383158 -0.002075314593441293 -0.008528594180120242 0.00819115155938407 -0.008890211097939558 0.012298352837836667 0.003910673918882549 -0.01234803828647054 -0.0057964584634248905 -0.0015056100686333016 </vector>

<vector>0.08674021054860832 -0.0037045545352846 -0.02063164485818054 -0.01832492815916663 -0.05894336056360844 -0.020023199977253898 -9.263901843943537E-5 0.006441517924026515 0.024284949784903486 0.018445968996550186 -0.018146795684346167 0.027430642134145908 0.0027288552302487225 0.015711831304237325 0.013228430612922804 -0.006773439125179123 </vector>

</data>

</datamatrix>

</datavector>

<idvec>Bambiraptor - Tsaagan Dromaeosaurus - Node 35 Saurornithoides - Node 34 Citipati - Ingenia Avimimus - Node 32 Node 31 - Node 33 Shuvuuia - Node 30 Falcarius - Node 29 Ornitholestes - Node 28 Gallimimus - Node 27 Qianzhousaurus - Tyrannosaurus Eotyrannus - Node 25 Guanlong - Node 24 Node 23 - Node 26 Bicentenaria - Node 22 Giganotosaurus - Shaochilong Acrocanthosaurus - Node 20 Aerosteon - Node 19 Allosaurus - Node 18 Node 17 - Node 21 Spinosaurus_I - Spinosaurus_II Baryonyx - Node 15 Afrovenator - Torvosaurus Eustreptospondylus - Node 13 Node 12 - Node 14 Node 11 - Node 16 Aucasaurus - Majungasaurus Carnotaurus - Node 9 Ilokelesia - Node 8 Masiakasaurus - Node 7 Ceratosaurus - Node 6 Node 5 - Node 10 Dilophosaurus - Node 4 Tawa - Node 3 Eodromaeus - Node 2 Herrerasaurus - Node 1</idvec>

<meanconfig>0.13495631814332032 0.16154075134581164 0.047755353507686074 0.17203387836539474 -0.31079316962390124 0.08271234990638376 -0.51103806369261 -0.06715247516695218 -0.23736469910379962 -0.12718055089786032 -0.1250018957230973 -0.15702801365668548 0.03448588140856004 -0.2192626495455337 0.3667739636867369 -0.09217087296522046 0.4253854840073151 0.18539389683426025 0.17484082738978995 0.061113685780401716 </meanconfig>

<omitflag>false false false false false false false false false false false false false false false false false false false false false false false false false false false false false false false false false false false false</omitflag>

<uniquenumvec>229 230 231 232 233 234 235 236 237 238 239 240 241 242 243 244 245 246 247 248 249 250 251 252 253 254 255 256 257 258 259 260 261 262 263 264 </uniquenumvec>

</morphojset_contents>

</morphojset>

</phylogeny>

</morphojset>

</principal_component_analysis>

</covariancematrix>

<wireframelinks>

<wireframelinks_content visiblename="Mandibular_articulation wireframe" nlmk="10" uniquenumber="42">

<links>

<vector>0 1 </vector>

<vector>1 2 </vector>

<vector>2 3 </vector>

<vector>3 4 </vector>

<vector>4 5 </vector>

<vector>5 6 </vector>

<vector>6 7 </vector>

<vector>7 8 </vector>

<vector>8 9 </vector>

<vector>6 9 </vector>

<vector>0 8 </vector>

<vector>1 4 </vector>

</links>

</wireframelinks_content>

</wireframelinks>

<phylogeny>

<phylogeny_content visiblename="Theropoda_Quadrate" matchestablished="true" mappingdone="true" ntaxa="37" rooted="true" weightedparsimony="false" withbranchlengths="false" uniqueid="43" matchclassifierindex="0" datatype="2" haspermtest="false" permiterations="0" permpval="NaN" treelength="0.9862015574327767">

<descendancest>

<vector>1 0 </vector>

<vector>2 0 </vector>

<vector>3 2 </vector>

<vector>4 2 </vector>

<vector>5 4 </vector>

<vector>6 4 </vector>

<vector>7 6 </vector>

<vector>8 6 </vector>

<vector>9 8 </vector>

<vector>10 9 </vector>

<vector>11 9 </vector>

<vector>12 11 </vector>

<vector>13 11 </vector>

<vector>14 13 </vector>

<vector>15 13 </vector>

<vector>16 15 </vector>

<vector>17 15 </vector>

<vector>18 17 </vector>

<vector>19 17 </vector>

<vector>20 8 </vector>

<vector>21 20 </vector>

<vector>22 21 </vector>

<vector>23 22 </vector>

<vector>24 22 </vector>

<vector>25 24 </vector>

<vector>26 24 </vector>

<vector>27 21 </vector>

<vector>28 27 </vector>

<vector>29 27 </vector>

<vector>30 29 </vector>

<vector>31 29 </vector>

<vector>32 20 </vector>

<vector>33 32 </vector>

<vector>34 33 </vector>

<vector>35 33 </vector>

<vector>36 35 </vector>

<vector>37 35 </vector>

<vector>38 37 </vector>

<vector>39 37 </vector>

<vector>40 39 </vector>

<vector>41 39 </vector>

<vector>42 32 </vector>

<vector>43 42 </vector>

<vector>44 42 </vector>

<vector>45 44 </vector>

<vector>46 45 </vector>

<vector>47 45 </vector>

<vector>48 47 </vector>

<vector>49 47 </vector>

<vector>50 49 </vector>

<vector>51 49 </vector>

<vector>52 44 </vector>

<vector>53 52 </vector>

<vector>54 52 </vector>

<vector>55 54 </vector>

<vector>56 54 </vector>

<vector>57 56 </vector>

<vector>58 56 </vector>

<vector>59 58 </vector>

<vector>60 58 </vector>

<vector>61 60 </vector>

<vector>62 61 </vector>

<vector>63 61 </vector>

<vector>64 63 </vector>

<vector>65 63 </vector>

<vector>66 60 </vector>

<vector>67 66 </vector>

<vector>68 66 </vector>

<vector>69 68 </vector>

<vector>70 68 </vector>

<vector>71 70 </vector>

<vector>72 70 </vector>

</descendancest>

<nodeNames>Root Herrerasaurus Node 1 Eodromaeus Node 2 Tawa Node 3 Dilophosaurus Node 4 Node 5 Ceratosaurus Node 6 Masiakasaurus Node 7 Ilokelesia Node 8 Carnotaurus Node 9 Majungasaurus Aucasaurus Node 10 Node 11 Node 12 Eustreptospondylus Node 13 Afrovenator Torvosaurus Node 14 Baryonyx Node 15 Spinosaurus_II Spinosaurus_I Node 16 Node 17 Allosaurus Node 18 Aerosteon Node 19 Acrocanthosaurus Node 20 Shaochilong Giganotosaurus Node 21 Bicentenaria Node 22 Node 23 Guanlong Node 24 Eotyrannus Node 25 Qianzhousaurus Tyrannosaurus Node 26 Gallimimus Node 27 Ornitholestes Node 28 Falcarius Node 29 Shuvuuia Node 30 Node 31 Avimimus Node 32 Citipati Ingenia Node 33 Saurornithoides Node 34 Dromaeosaurus Node 35 Bambiraptor Tsaagan</nodeNames>

<taxanames>Acrocanthosaurus Aerosteon Afrovenator Allosaurus Aucasaurus Avimimus Bambiraptor Baryonyx Bicentenaria Carnotaurus Ceratosaurus Citipati Dilophosaurus Dromaeosaurus Eodromaeus Eotyrannus Eustreptospondylus Falcarius Gallimimus Giganotosaurus Guanlong Herrerasaurus Ilokelesia Ingenia Majungasaurus Masiakasaurus Ornitholestes Qianzhousaurus Saurornithoides Shaochilong Shuvuuia Spinosaurus_I Spinosaurus_II Tawa Torvosaurus Tsaagan Tyrannosaurus</taxanames>

<taxonvec>0 1 2 3 4 5 6 7 8 9 10 11 12 13 14 15 16 17 18 19 20 21 22 23 24 25 26 27 28 29 30 31 32 33 34 35 36 </taxonvec>

<ancestorvec>53 54 59 55 63 41 37 58 51 64 67 40 69 38 71 48 60 44 46 52 49 72 65 40 63 66 45 47 39 52 43 57 57 70 59 37 47 38 39 42 41 42 43 44 45 46 50 48 49 50 51 56 53 54 55 56 62 58 61 60 61 62 68 64 65 66 67 68 69 70 71 72 </ancestorvec>

<branchlengthvec>1.0 1.0 1.0 1.0 1.0 1.0 1.0 1.0 1.0 1.0 1.0 1.0 1.0 1.0 1.0 1.0 1.0 1.0 1.0 1.0 1.0 1.0 1.0 1.0 1.0 1.0 1.0 1.0 1.0 1.0 1.0 1.0 1.0 1.0 1.0 1.0 1.0 1.0 1.0 1.0 1.0 1.0 1.0 1.0 1.0 1.0 1.0 1.0 1.0 1.0 1.0 1.0 1.0 1.0 1.0 1.0 1.0 1.0 1.0 1.0 1.0 1.0 1.0 1.0 1.0 1.0 1.0 1.0 1.0 1.0 1.0 1.0 </branchlengthvec>

<interntermcov>

<vector>6.0 6.0 5.0 6.0 4.0 13.0 16.0 5.0 7.0 4.0 4.0 13.0 3.0 15.0 1.0 8.0 5.0 11.0 9.0 6.0 8.0 0.0 4.0 13.0 4.0 4.0 10.0 8.0 14.0 6.0 12.0 5.0 5.0 2.0 5.0 16.0 8.0 </vector>

<vector>6.0 6.0 5.0 6.0 4.0 13.0 15.0 5.0 7.0 4.0 4.0 13.0 3.0 15.0 1.0 8.0 5.0 11.0 9.0 6.0 8.0 0.0 4.0 13.0 4.0 4.0 10.0 8.0 14.0 6.0 12.0 5.0 5.0 2.0 5.0 15.0 8.0 </vector>

<vector>6.0 6.0 5.0 6.0 4.0 13.0 14.0 5.0 7.0 4.0 4.0 13.0 3.0 14.0 1.0 8.0 5.0 11.0 9.0 6.0 8.0 0.0 4.0 13.0 4.0 4.0 10.0 8.0 14.0 6.0 12.0 5.0 5.0 2.0 5.0 14.0 8.0 </vector>

<vector>6.0 6.0 5.0 6.0 4.0 14.0 13.0 5.0 7.0 4.0 4.0 15.0 3.0 13.0 1.0 8.0 5.0 11.0 9.0 6.0 8.0 0.0 4.0 15.0 4.0 4.0 10.0 8.0 13.0 6.0 12.0 5.0 5.0 2.0 5.0 13.0 8.0 </vector>

<vector>6.0 6.0 5.0 6.0 4.0 14.0 13.0 5.0 7.0 4.0 4.0 14.0 3.0 13.0 1.0 8.0 5.0 11.0 9.0 6.0 8.0 0.0 4.0 14.0 4.0 4.0 10.0 8.0 13.0 6.0 12.0 5.0 5.0 2.0 5.0 13.0 8.0 </vector>

<vector>6.0 6.0 5.0 6.0 4.0 13.0 13.0 5.0 7.0 4.0 4.0 13.0 3.0 13.0 1.0 8.0 5.0 11.0 9.0 6.0 8.0 0.0 4.0 13.0 4.0 4.0 10.0 8.0 13.0 6.0 12.0 5.0 5.0 2.0 5.0 13.0 8.0 </vector>

<vector>6.0 6.0 5.0 6.0 4.0 12.0 12.0 5.0 7.0 4.0 4.0 12.0 3.0 12.0 1.0 8.0 5.0 11.0 9.0 6.0 8.0 0.0 4.0 12.0 4.0 4.0 10.0 8.0 12.0 6.0 12.0 5.0 5.0 2.0 5.0 12.0 8.0 </vector>

<vector>6.0 6.0 5.0 6.0 4.0 11.0 11.0 5.0 7.0 4.0 4.0 11.0 3.0 11.0 1.0 8.0 5.0 11.0 9.0 6.0 8.0 0.0 4.0 11.0 4.0 4.0 10.0 8.0 11.0 6.0 11.0 5.0 5.0 2.0 5.0 11.0 8.0 </vector>

<vector>6.0 6.0 5.0 6.0 4.0 10.0 10.0 5.0 7.0 4.0 4.0 10.0 3.0 10.0 1.0 8.0 5.0 10.0 9.0 6.0 8.0 0.0 4.0 10.0 4.0 4.0 10.0 8.0 10.0 6.0 10.0 5.0 5.0 2.0 5.0 10.0 8.0 </vector>

<vector>6.0 6.0 5.0 6.0 4.0 9.0 9.0 5.0 7.0 4.0 4.0 9.0 3.0 9.0 1.0 8.0 5.0 9.0 9.0 6.0 8.0 0.0 4.0 9.0 4.0 4.0 9.0 8.0 9.0 6.0 9.0 5.0 5.0 2.0 5.0 9.0 8.0 </vector>

<vector>6.0 6.0 5.0 6.0 4.0 8.0 8.0 5.0 7.0 4.0 4.0 8.0 3.0 8.0 1.0 10.0 5.0 8.0 8.0 6.0 9.0 0.0 4.0 8.0 4.0 4.0 8.0 11.0 8.0 6.0 8.0 5.0 5.0 2.0 5.0 8.0 11.0 </vector>

<vector>6.0 6.0 5.0 6.0 4.0 8.0 8.0 5.0 7.0 4.0 4.0 8.0 3.0 8.0 1.0 10.0 5.0 8.0 8.0 6.0 9.0 0.0 4.0 8.0 4.0 4.0 8.0 10.0 8.0 6.0 8.0 5.0 5.0 2.0 5.0 8.0 10.0 </vector>

<vector>6.0 6.0 5.0 6.0 4.0 8.0 8.0 5.0 7.0 4.0 4.0 8.0 3.0 8.0 1.0 9.0 5.0 8.0 8.0 6.0 9.0 0.0 4.0 8.0 4.0 4.0 8.0 9.0 8.0 6.0 8.0 5.0 5.0 2.0 5.0 8.0 9.0 </vector>

<vector>6.0 6.0 5.0 6.0 4.0 8.0 8.0 5.0 7.0 4.0 4.0 8.0 3.0 8.0 1.0 8.0 5.0 8.0 8.0 6.0 8.0 0.0 4.0 8.0 4.0 4.0 8.0 8.0 8.0 6.0 8.0 5.0 5.0 2.0 5.0 8.0 8.0 </vector>

<vector>6.0 6.0 5.0 6.0 4.0 7.0 7.0 5.0 7.0 4.0 4.0 7.0 3.0 7.0 1.0 7.0 5.0 7.0 7.0 6.0 7.0 0.0 4.0 7.0 4.0 4.0 7.0 7.0 7.0 6.0 7.0 5.0 5.0 2.0 5.0 7.0 7.0 </vector>

<vector>9.0 8.0 5.0 7.0 4.0 6.0 6.0 5.0 6.0 4.0 4.0 6.0 3.0 6.0 1.0 6.0 5.0 6.0 6.0 10.0 6.0 0.0 4.0 6.0 4.0 4.0 6.0 6.0 6.0 10.0 6.0 5.0 5.0 2.0 5.0 6.0 6.0 </vector>

<vector>9.0 8.0 5.0 7.0 4.0 6.0 6.0 5.0 6.0 4.0 4.0 6.0 3.0 6.0 1.0 6.0 5.0 6.0 6.0 9.0 6.0 0.0 4.0 6.0 4.0 4.0 6.0 6.0 6.0 9.0 6.0 5.0 5.0 2.0 5.0 6.0 6.0 </vector>

<vector>8.0 8.0 5.0 7.0 4.0 6.0 6.0 5.0 6.0 4.0 4.0 6.0 3.0 6.0 1.0 6.0 5.0 6.0 6.0 8.0 6.0 0.0 4.0 6.0 4.0 4.0 6.0 6.0 6.0 8.0 6.0 5.0 5.0 2.0 5.0 6.0 6.0 </vector>

<vector>7.0 7.0 5.0 7.0 4.0 6.0 6.0 5.0 6.0 4.0 4.0 6.0 3.0 6.0 1.0 6.0 5.0 6.0 6.0 7.0 6.0 0.0 4.0 6.0 4.0 4.0 6.0 6.0 6.0 7.0 6.0 5.0 5.0 2.0 5.0 6.0 6.0 </vector>

<vector>6.0 6.0 5.0 6.0 4.0 6.0 6.0 5.0 6.0 4.0 4.0 6.0 3.0 6.0 1.0 6.0 5.0 6.0 6.0 6.0 6.0 0.0 4.0 6.0 4.0 4.0 6.0 6.0 6.0 6.0 6.0 5.0 5.0 2.0 5.0 6.0 6.0 </vector>

<vector>5.0 5.0 6.0 5.0 4.0 5.0 5.0 7.0 5.0 4.0 4.0 5.0 3.0 5.0 1.0 5.0 6.0 5.0 5.0 5.0 5.0 0.0 4.0 5.0 4.0 4.0 5.0 5.0 5.0 5.0 5.0 8.0 8.0 2.0 6.0 5.0 5.0 </vector>

<vector>5.0 5.0 6.0 5.0 4.0 5.0 5.0 7.0 5.0 4.0 4.0 5.0 3.0 5.0 1.0 5.0 6.0 5.0 5.0 5.0 5.0 0.0 4.0 5.0 4.0 4.0 5.0 5.0 5.0 5.0 5.0 7.0 7.0 2.0 6.0 5.0 5.0 </vector>

<vector>5.0 5.0 8.0 5.0 4.0 5.0 5.0 6.0 5.0 4.0 4.0 5.0 3.0 5.0 1.0 5.0 7.0 5.0 5.0 5.0 5.0 0.0 4.0 5.0 4.0 4.0 5.0 5.0 5.0 5.0 5.0 6.0 6.0 2.0 8.0 5.0 5.0 </vector>

<vector>5.0 5.0 7.0 5.0 4.0 5.0 5.0 6.0 5.0 4.0 4.0 5.0 3.0 5.0 1.0 5.0 7.0 5.0 5.0 5.0 5.0 0.0 4.0 5.0 4.0 4.0 5.0 5.0 5.0 5.0 5.0 6.0 6.0 2.0 7.0 5.0 5.0 </vector>

<vector>5.0 5.0 6.0 5.0 4.0 5.0 5.0 6.0 5.0 4.0 4.0 5.0 3.0 5.0 1.0 5.0 6.0 5.0 5.0 5.0 5.0 0.0 4.0 5.0 4.0 4.0 5.0 5.0 5.0 5.0 5.0 6.0 6.0 2.0 6.0 5.0 5.0 </vector>

<vector>5.0 5.0 5.0 5.0 4.0 5.0 5.0 5.0 5.0 4.0 4.0 5.0 3.0 5.0 1.0 5.0 5.0 5.0 5.0 5.0 5.0 0.0 4.0 5.0 4.0 4.0 5.0 5.0 5.0 5.0 5.0 5.0 5.0 2.0 5.0 5.0 5.0 </vector>

<vector>4.0 4.0 4.0 4.0 9.0 4.0 4.0 4.0 4.0 8.0 5.0 4.0 3.0 4.0 1.0 4.0 4.0 4.0 4.0 4.0 4.0 0.0 7.0 4.0 9.0 6.0 4.0 4.0 4.0 4.0 4.0 4.0 4.0 2.0 4.0 4.0 4.0 </vector>

<vector>4.0 4.0 4.0 4.0 8.0 4.0 4.0 4.0 4.0 8.0 5.0 4.0 3.0 4.0 1.0 4.0 4.0 4.0 4.0 4.0 4.0 0.0 7.0 4.0 8.0 6.0 4.0 4.0 4.0 4.0 4.0 4.0 4.0 2.0 4.0 4.0 4.0 </vector>

<vector>4.0 4.0 4.0 4.0 7.0 4.0 4.0 4.0 4.0 7.0 5.0 4.0 3.0 4.0 1.0 4.0 4.0 4.0 4.0 4.0 4.0 0.0 7.0 4.0 7.0 6.0 4.0 4.0 4.0 4.0 4.0 4.0 4.0 2.0 4.0 4.0 4.0 </vector>

<vector>4.0 4.0 4.0 4.0 6.0 4.0 4.0 4.0 4.0 6.0 5.0 4.0 3.0 4.0 1.0 4.0 4.0 4.0 4.0 4.0 4.0 0.0 6.0 4.0 6.0 6.0 4.0 4.0 4.0 4.0 4.0 4.0 4.0 2.0 4.0 4.0 4.0 </vector>

<vector>4.0 4.0 4.0 4.0 5.0 4.0 4.0 4.0 4.0 5.0 5.0 4.0 3.0 4.0 1.0 4.0 4.0 4.0 4.0 4.0 4.0 0.0 5.0 4.0 5.0 5.0 4.0 4.0 4.0 4.0 4.0 4.0 4.0 2.0 4.0 4.0 4.0 </vector>

<vector>4.0 4.0 4.0 4.0 4.0 4.0 4.0 4.0 4.0 4.0 4.0 4.0 3.0 4.0 1.0 4.0 4.0 4.0 4.0 4.0 4.0 0.0 4.0 4.0 4.0 4.0 4.0 4.0 4.0 4.0 4.0 4.0 4.0 2.0 4.0 4.0 4.0 </vector>

<vector>3.0 3.0 3.0 3.0 3.0 3.0 3.0 3.0 3.0 3.0 3.0 3.0 3.0 3.0 1.0 3.0 3.0 3.0 3.0 3.0 3.0 0.0 3.0 3.0 3.0 3.0 3.0 3.0 3.0 3.0 3.0 3.0 3.0 2.0 3.0 3.0 3.0 </vector>

<vector>2.0 2.0 2.0 2.0 2.0 2.0 2.0 2.0 2.0 2.0 2.0 2.0 2.0 2.0 1.0 2.0 2.0 2.0 2.0 2.0 2.0 0.0 2.0 2.0 2.0 2.0 2.0 2.0 2.0 2.0 2.0 2.0 2.0 2.0 2.0 2.0 2.0 </vector>

<vector>1.0 1.0 1.0 1.0 1.0 1.0 1.0 1.0 1.0 1.0 1.0 1.0 1.0 1.0 1.0 1.0 1.0 1.0 1.0 1.0 1.0 0.0 1.0 1.0 1.0 1.0 1.0 1.0 1.0 1.0 1.0 1.0 1.0 1.0 1.0 1.0 1.0 </vector>

<vector>0.0 0.0 0.0 0.0 0.0 0.0 0.0 0.0 0.0 0.0 0.0 0.0 0.0 0.0 0.0 0.0 0.0 0.0 0.0 0.0 0.0 0.0 0.0 0.0 0.0 0.0 0.0 0.0 0.0 0.0 0.0 0.0 0.0 0.0 0.0 0.0 0.0 </vector>

</interntermcov>

<phylocov>

<vector>10.0 8.0 5.0 7.0 4.0 6.0 6.0 5.0 6.0 4.0 4.0 6.0 3.0 6.0 1.0 6.0 5.0 6.0 6.0 9.0 6.0 0.0 4.0 6.0 4.0 4.0 6.0 6.0 6.0 9.0 6.0 5.0 5.0 2.0 5.0 6.0 6.0 </vector>

<vector>8.0 9.0 5.0 7.0 4.0 6.0 6.0 5.0 6.0 4.0 4.0 6.0 3.0 6.0 1.0 6.0 5.0 6.0 6.0 8.0 6.0 0.0 4.0 6.0 4.0 4.0 6.0 6.0 6.0 8.0 6.0 5.0 5.0 2.0 5.0 6.0 6.0 </vector>

<vector>5.0 5.0 9.0 5.0 4.0 5.0 5.0 6.0 5.0 4.0 4.0 5.0 3.0 5.0 1.0 5.0 7.0 5.0 5.0 5.0 5.0 0.0 4.0 5.0 4.0 4.0 5.0 5.0 5.0 5.0 5.0 6.0 6.0 2.0 8.0 5.0 5.0 </vector>

<vector>7.0 7.0 5.0 8.0 4.0 6.0 6.0 5.0 6.0 4.0 4.0 6.0 3.0 6.0 1.0 6.0 5.0 6.0 6.0 7.0 6.0 0.0 4.0 6.0 4.0 4.0 6.0 6.0 6.0 7.0 6.0 5.0 5.0 2.0 5.0 6.0 6.0 </vector>

<vector>4.0 4.0 4.0 4.0 10.0 4.0 4.0 4.0 4.0 8.0 5.0 4.0 3.0 4.0 1.0 4.0 4.0 4.0 4.0 4.0 4.0 0.0 7.0 4.0 9.0 6.0 4.0 4.0 4.0 4.0 4.0 4.0 4.0 2.0 4.0 4.0 4.0 </vector>

<vector>6.0 6.0 5.0 6.0 4.0 15.0 13.0 5.0 7.0 4.0 4.0 14.0 3.0 13.0 1.0 8.0 5.0 11.0 9.0 6.0 8.0 0.0 4.0 14.0 4.0 4.0 10.0 8.0 13.0 6.0 12.0 5.0 5.0 2.0 5.0 13.0 8.0 </vector>

<vector>6.0 6.0 5.0 6.0 4.0 13.0 17.0 5.0 7.0 4.0 4.0 13.0 3.0 15.0 1.0 8.0 5.0 11.0 9.0 6.0 8.0 0.0 4.0 13.0 4.0 4.0 10.0 8.0 14.0 6.0 12.0 5.0 5.0 2.0 5.0 16.0 8.0 </vector>

<vector>5.0 5.0 6.0 5.0 4.0 5.0 5.0 8.0 5.0 4.0 4.0 5.0 3.0 5.0 1.0 5.0 6.0 5.0 5.0 5.0 5.0 0.0 4.0 5.0 4.0 4.0 5.0 5.0 5.0 5.0 5.0 7.0 7.0 2.0 6.0 5.0 5.0 </vector>

<vector>6.0 6.0 5.0 6.0 4.0 7.0 7.0 5.0 8.0 4.0 4.0 7.0 3.0 7.0 1.0 7.0 5.0 7.0 7.0 6.0 7.0 0.0 4.0 7.0 4.0 4.0 7.0 7.0 7.0 6.0 7.0 5.0 5.0 2.0 5.0 7.0 7.0 </vector>

<vector>4.0 4.0 4.0 4.0 8.0 4.0 4.0 4.0 4.0 9.0 5.0 4.0 3.0 4.0 1.0 4.0 4.0 4.0 4.0 4.0 4.0 0.0 7.0 4.0 8.0 6.0 4.0 4.0 4.0 4.0 4.0 4.0 4.0 2.0 4.0 4.0 4.0 </vector>

<vector>4.0 4.0 4.0 4.0 5.0 4.0 4.0 4.0 4.0 5.0 6.0 4.0 3.0 4.0 1.0 4.0 4.0 4.0 4.0 4.0 4.0 0.0 5.0 4.0 5.0 5.0 4.0 4.0 4.0 4.0 4.0 4.0 4.0 2.0 4.0 4.0 4.0 </vector>

<vector>6.0 6.0 5.0 6.0 4.0 14.0 13.0 5.0 7.0 4.0 4.0 16.0 3.0 13.0 1.0 8.0 5.0 11.0 9.0 6.0 8.0 0.0 4.0 15.0 4.0 4.0 10.0 8.0 13.0 6.0 12.0 5.0 5.0 2.0 5.0 13.0 8.0 </vector>

<vector>3.0 3.0 3.0 3.0 3.0 3.0 3.0 3.0 3.0 3.0 3.0 3.0 4.0 3.0 1.0 3.0 3.0 3.0 3.0 3.0 3.0 0.0 3.0 3.0 3.0 3.0 3.0 3.0 3.0 3.0 3.0 3.0 3.0 2.0 3.0 3.0 3.0 </vector>

<vector>6.0 6.0 5.0 6.0 4.0 13.0 15.0 5.0 7.0 4.0 4.0 13.0 3.0 16.0 1.0 8.0 5.0 11.0 9.0 6.0 8.0 0.0 4.0 13.0 4.0 4.0 10.0 8.0 14.0 6.0 12.0 5.0 5.0 2.0 5.0 15.0 8.0 </vector>

<vector>1.0 1.0 1.0 1.0 1.0 1.0 1.0 1.0 1.0 1.0 1.0 1.0 1.0 1.0 2.0 1.0 1.0 1.0 1.0 1.0 1.0 0.0 1.0 1.0 1.0 1.0 1.0 1.0 1.0 1.0 1.0 1.0 1.0 1.0 1.0 1.0 1.0 </vector>

<vector>6.0 6.0 5.0 6.0 4.0 8.0 8.0 5.0 7.0 4.0 4.0 8.0 3.0 8.0 1.0 11.0 5.0 8.0 8.0 6.0 9.0 0.0 4.0 8.0 4.0 4.0 8.0 10.0 8.0 6.0 8.0 5.0 5.0 2.0 5.0 8.0 10.0 </vector>

<vector>5.0 5.0 7.0 5.0 4.0 5.0 5.0 6.0 5.0 4.0 4.0 5.0 3.0 5.0 1.0 5.0 8.0 5.0 5.0 5.0 5.0 0.0 4.0 5.0 4.0 4.0 5.0 5.0 5.0 5.0 5.0 6.0 6.0 2.0 7.0 5.0 5.0 </vector>

<vector>6.0 6.0 5.0 6.0 4.0 11.0 11.0 5.0 7.0 4.0 4.0 11.0 3.0 11.0 1.0 8.0 5.0 12.0 9.0 6.0 8.0 0.0 4.0 11.0 4.0 4.0 10.0 8.0 11.0 6.0 11.0 5.0 5.0 2.0 5.0 11.0 8.0 </vector>

<vector>6.0 6.0 5.0 6.0 4.0 9.0 9.0 5.0 7.0 4.0 4.0 9.0 3.0 9.0 1.0 8.0 5.0 9.0 10.0 6.0 8.0 0.0 4.0 9.0 4.0 4.0 9.0 8.0 9.0 6.0 9.0 5.0 5.0 2.0 5.0 9.0 8.0 </vector>

<vector>9.0 8.0 5.0 7.0 4.0 6.0 6.0 5.0 6.0 4.0 4.0 6.0 3.0 6.0 1.0 6.0 5.0 6.0 6.0 11.0 6.0 0.0 4.0 6.0 4.0 4.0 6.0 6.0 6.0 10.0 6.0 5.0 5.0 2.0 5.0 6.0 6.0 </vector>

<vector>6.0 6.0 5.0 6.0 4.0 8.0 8.0 5.0 7.0 4.0 4.0 8.0 3.0 8.0 1.0 9.0 5.0 8.0 8.0 6.0 10.0 0.0 4.0 8.0 4.0 4.0 8.0 9.0 8.0 6.0 8.0 5.0 5.0 2.0 5.0 8.0 9.0 </vector>

<vector>0.0 0.0 0.0 0.0 0.0 0.0 0.0 0.0 0.0 0.0 0.0 0.0 0.0 0.0 0.0 0.0 0.0 0.0 0.0 0.0 0.0 1.0 0.0 0.0 0.0 0.0 0.0 0.0 0.0 0.0 0.0 0.0 0.0 0.0 0.0 0.0 0.0 </vector>

<vector>4.0 4.0 4.0 4.0 7.0 4.0 4.0 4.0 4.0 7.0 5.0 4.0 3.0 4.0 1.0 4.0 4.0 4.0 4.0 4.0 4.0 0.0 8.0 4.0 7.0 6.0 4.0 4.0 4.0 4.0 4.0 4.0 4.0 2.0 4.0 4.0 4.0 </vector>

<vector>6.0 6.0 5.0 6.0 4.0 14.0 13.0 5.0 7.0 4.0 4.0 15.0 3.0 13.0 1.0 8.0 5.0 11.0 9.0 6.0 8.0 0.0 4.0 16.0 4.0 4.0 10.0 8.0 13.0 6.0 12.0 5.0 5.0 2.0 5.0 13.0 8.0 </vector>

<vector>4.0 4.0 4.0 4.0 9.0 4.0 4.0 4.0 4.0 8.0 5.0 4.0 3.0 4.0 1.0 4.0 4.0 4.0 4.0 4.0 4.0 0.0 7.0 4.0 10.0 6.0 4.0 4.0 4.0 4.0 4.0 4.0 4.0 2.0 4.0 4.0 4.0 </vector>

<vector>4.0 4.0 4.0 4.0 6.0 4.0 4.0 4.0 4.0 6.0 5.0 4.0 3.0 4.0 1.0 4.0 4.0 4.0 4.0 4.0 4.0 0.0 6.0 4.0 6.0 7.0 4.0 4.0 4.0 4.0 4.0 4.0 4.0 2.0 4.0 4.0 4.0 </vector>

<vector>6.0 6.0 5.0 6.0 4.0 10.0 10.0 5.0 7.0 4.0 4.0 10.0 3.0 10.0 1.0 8.0 5.0 10.0 9.0 6.0 8.0 0.0 4.0 10.0 4.0 4.0 11.0 8.0 10.0 6.0 10.0 5.0 5.0 2.0 5.0 10.0 8.0 </vector>

<vector>6.0 6.0 5.0 6.0 4.0 8.0 8.0 5.0 7.0 4.0 4.0 8.0 3.0 8.0 1.0 10.0 5.0 8.0 8.0 6.0 9.0 0.0 4.0 8.0 4.0 4.0 8.0 12.0 8.0 6.0 8.0 5.0 5.0 2.0 5.0 8.0 11.0 </vector>

<vector>6.0 6.0 5.0 6.0 4.0 13.0 14.0 5.0 7.0 4.0 4.0 13.0 3.0 14.0 1.0 8.0 5.0 11.0 9.0 6.0 8.0 0.0 4.0 13.0 4.0 4.0 10.0 8.0 15.0 6.0 12.0 5.0 5.0 2.0 5.0 14.0 8.0 </vector>

<vector>9.0 8.0 5.0 7.0 4.0 6.0 6.0 5.0 6.0 4.0 4.0 6.0 3.0 6.0 1.0 6.0 5.0 6.0 6.0 10.0 6.0 0.0 4.0 6.0 4.0 4.0 6.0 6.0 6.0 11.0 6.0 5.0 5.0 2.0 5.0 6.0 6.0 </vector>

<vector>6.0 6.0 5.0 6.0 4.0 12.0 12.0 5.0 7.0 4.0 4.0 12.0 3.0 12.0 1.0 8.0 5.0 11.0 9.0 6.0 8.0 0.0 4.0 12.0 4.0 4.0 10.0 8.0 12.0 6.0 13.0 5.0 5.0 2.0 5.0 12.0 8.0 </vector>

<vector>5.0 5.0 6.0 5.0 4.0 5.0 5.0 7.0 5.0 4.0 4.0 5.0 3.0 5.0 1.0 5.0 6.0 5.0 5.0 5.0 5.0 0.0 4.0 5.0 4.0 4.0 5.0 5.0 5.0 5.0 5.0 9.0 8.0 2.0 6.0 5.0 5.0 </vector>

<vector>5.0 5.0 6.0 5.0 4.0 5.0 5.0 7.0 5.0 4.0 4.0 5.0 3.0 5.0 1.0 5.0 6.0 5.0 5.0 5.0 5.0 0.0 4.0 5.0 4.0 4.0 5.0 5.0 5.0 5.0 5.0 8.0 9.0 2.0 6.0 5.0 5.0 </vector>

<vector>2.0 2.0 2.0 2.0 2.0 2.0 2.0 2.0 2.0 2.0 2.0 2.0 2.0 2.0 1.0 2.0 2.0 2.0 2.0 2.0 2.0 0.0 2.0 2.0 2.0 2.0 2.0 2.0 2.0 2.0 2.0 2.0 2.0 3.0 2.0 2.0 2.0 </vector>

<vector>5.0 5.0 8.0 5.0 4.0 5.0 5.0 6.0 5.0 4.0 4.0 5.0 3.0 5.0 1.0 5.0 7.0 5.0 5.0 5.0 5.0 0.0 4.0 5.0 4.0 4.0 5.0 5.0 5.0 5.0 5.0 6.0 6.0 2.0 9.0 5.0 5.0 </vector>

<vector>6.0 6.0 5.0 6.0 4.0 13.0 16.0 5.0 7.0 4.0 4.0 13.0 3.0 15.0 1.0 8.0 5.0 11.0 9.0 6.0 8.0 0.0 4.0 13.0 4.0 4.0 10.0 8.0 14.0 6.0 12.0 5.0 5.0 2.0 5.0 17.0 8.0 </vector>

<vector>6.0 6.0 5.0 6.0 4.0 8.0 8.0 5.0 7.0 4.0 4.0 8.0 3.0 8.0 1.0 10.0 5.0 8.0 8.0 6.0 9.0 0.0 4.0 8.0 4.0 4.0 8.0 11.0 8.0 6.0 8.0 5.0 5.0 2.0 5.0 8.0 12.0 </vector>

</phylocov>

<nodelabels>Acrocanthosaurus Aerosteon Afrovenator Allosaurus Aucasaurus Avimimus Bambiraptor Baryonyx Bicentenaria Carnotaurus Ceratosaurus Citipati Dilophosaurus Dromaeosaurus Eodromaeus Eotyrannus Eustreptospondylus Falcarius Gallimimus Giganotosaurus Guanlong Herrerasaurus Ilokelesia Ingenia Majungasaurus Masiakasaurus Ornitholestes Qianzhousaurus Saurornithoides Shaochilong Shuvuuia Spinosaurus_I Spinosaurus_II Tawa Torvosaurus Tsaagan Tyrannosaurus Node 35 Node 34 Node 33 Node 32 Node 31 Node 30 Node 29 Node 28 Node 27 Node 26 Node 25 Node 24 Node 23 Node 22 Node 21 Node 20 Node 19 Node 18 Node 17 Node 16 Node 15 Node 14 Node 13 Node 12 Node 11 Node 10 Node 9 Node 8 Node 7 Node 6 Node 5 Node 4 Node 3 Node 2 Node 1 Root</nodelabels>

<ancestpheno>

<vector>0.12498827716752975 0.12198037137802255 0.04293543689121951 0.12449159001217908 -0.3468417683149114 -0.007497800319225714 -0.5097537318338753 0.02371771886015954 -0.38228527058279743 -0.16071857466461803 -0.02897776788745554 -0.13605241393029707 0.08450592814116593 -0.16252067107285342 0.35254893790475555 -0.06342283423211245 0.43715807149733926 0.1825566610845211 0.22572188701702978 0.07746595288422448 </vector>

<vector>0.09547350986898026 0.12305228819711135 -0.004080911667538198 0.12219970185466493 -0.29647908081670443 0.052741714010226974 -0.5142941228229622 -0.017601480246482518 -0.33537806402031467 -0.16004761605328224 -0.061644806468962685 -0.14023459139740171 0.06573995880535861 -0.17173847315384316 0.3764105329219732 -0.09436182545957987 0.4470271471025319 0.17835799320634735 0.22722583709763816 0.10763228904223902 </vector>

<vector>0.07725256562807689 0.13183021478457088 -0.03362539745810028 0.11977094171045938 -0.3108604340584106 0.07735946898043808 -0.51951724549233 -0.07121443032445127 -0.2761810304364806 -0.15549616173687594 -0.06723751188859253 -0.13155959273033818 0.04196660107115842 -0.12522733286639284 0.3527160155147959 -0.1290921747337541 0.5015711618370305 0.1595911373260228 0.23391527528285197 0.12403792959032134 </vector>

<vector>0.1069351426685018 0.20464650525608483 0.0012044829584512556 0.21452949643915095 -0.3269016812790054 0.13637500970952315 -0.45006805802408983 -0.09424534657332308 -0.1695963276801631 -0.10600400859679729 -0.15946755616581815 -0.18364725043782593 0.02198817443511134 -0.25608018756830053 0.41350184450173494 -0.13001420381875206 0.4445786822629354 0.16455964872210316 0.1178252963223417 0.049880336868137 </vector>

<vector>0.1129339814607552 0.1843945375283957 0.024006447869011786 0.1652597518285422 -0.3153222741059516 0.11284947267501544 -0.4897165756682615 -0.0850115156517332 -0.20240319130058193 -0.12008596169858517 -0.15538762666681621 -0.16232281512781394 0.03708094614889068 -0.21941149128091975 0.38017689441846453 -0.11649197995240386 0.4399118161160156 0.18464014529696998 0.1687195817284733 0.05617985638253277 </vector>

<vector>0.08254810767280168 0.15140557378388692 -0.013330104956051789 0.128156799922901 -0.3188440753602656 0.09553633442658085 -0.5097663858601302 -0.07971929222913231 -0.24849093988249416 -0.13753024364591077 -0.10017363660015033 -0.1396555155129629 0.0445410075612835 -0.15444522954725612 0.37157303486430454 -0.1162815033862844 0.4804742234952526 0.16100971441521456 0.2114687690654496 0.09152336177296337 </vector>

<vector>0.05745777592957263 0.13799196903869404 -0.03037136527906653 0.09943970622970075 -0.3303495179164325 0.09640006162428988 -0.5200653364198 -0.08293193071121294 -0.2668885979104196 -0.13700860750227142 -0.07789577124504357 -0.1250841386807367 0.05457547546380093 -0.11869686449445607 0.3818261946596539 -0.10326035547269456 0.49993969253271076 0.1387978606226511 0.23177145018502407 0.09435229934603609 </vector>

<vector>0.06307399485706766 0.15289438850435655 -0.05600537654264126 0.1689825335833754 -0.34940080693732606 0.10558785016515536 -0.5056086598933525 -0.08258742108985326 -0.24111968637323478 -0.09481673278854297 -0.08892780408108505 -0.12186949093188235 0.1092134596693158 -0.1827561594194458 0.4050209809052245 -0.1186602543065742 0.45775550401167875 0.1202096879577139 0.20599839438435286 0.053015598325697535 </vector>

<vector>0.09448232962309237 0.17806445566344697 -0.032845742883867934 0.20142564353405681 -0.33787776623146154 0.09365425403971361 -0.5200851382375398 -0.08430403825245929 -0.20873780214547388 -0.09975821190199256 -0.07707424140274445 -0.13709116049831777 0.06886268309731822 -0.20986525319268773 0.36972219649294247 -0.11525157453677459 0.45451513722161463 0.13043245030576572 0.18903834446611967 0.04269343483924893 </vector>

<vector>0.1259529175414414 0.16300358773854717 -0.028751144445041968 0.17779545482225229 -0.3515206454283336 0.052658668666291854 -0.5537819699237818 5.358072962591692E-4 -0.20420274936785182 -0.13795189616266854 -0.06380344591108203 -0.14682953943848803 0.1044687129284391 -0.20647832361145202 0.3688043465484973 -0.09645907711151241 0.42260088124791023 0.14227354433560116 0.18023309680980287 0.05145177346516947 </vector>

<vector>0.09376297225526192 0.1435770339604347 -0.014566924227500722 0.16132918517387032 -0.3098596853854862 0.1086566268550382 -0.4921042200959234 -0.05641495223888442 -0.2305279902621273 -0.11470545423317627 -0.15321113627262056 -0.14755278657943321 0.07520605728562953 -0.231691526199173 0.3996095679254444 -0.08610119279802377 0.43932339721977454 0.18400228177756758 0.19236796155754796 0.03890078428178006 </vector>

<vector>0.10337774998169517 0.15222451584776392 0.031218686835887577 0.16960624197061458 -0.2972634772813375 0.10090756742910165 -0.5141320080607289 -0.07753767965743366 -0.21609746651057804 -0.10415034603398313 -0.15239663805616194 -0.16608014105585028 0.05510945197893666 -0.2307746296557558 0.3627717432557075 -0.09038570061178894 0.4414448331479351 0.18673334875914502 0.18596712470864438 0.05945682300818674 </vector>

<vector>0.14037279893581833 0.15109453292407884 0.05492187150865917 0.1788331637202447 -0.29985770267728334 0.08697468737558905 -0.5468411521131351 -0.07634232449927705 -0.2045868980750551 -0.11455834691163183 -0.18313641778272954 -0.15502766783647973 0.09204323467026243 -0.21772841130482776 0.3538026019417133 -0.06890982793183559 0.41321158090441595 0.16456910727482452 0.180070082687334 0.05109508718931498 </vector>

<vector>0.1555096716487012 0.16362991383127648 0.03410055323949605 0.19297605747435345 -0.320033630012079 0.08145961622709838 -0.5533308626035816 -0.0684632619777 -0.20427394658388912 -0.12189892289264614 -0.11736514554907941 -0.15095130694797526 0.07733440918439957 -0.2164602488083497 0.3478120979138412 -0.08724137673977778 0.4113142923358462 0.15228917787307372 0.16893256042634475 0.054660351960646975 </vector>

<vector>0.2002032984688439 0.1767916208312039 0.07613093265487098 0.22229955388056413 -0.3087225419306195 0.104745492639415 -0.5593694657738278 -0.1295832687300827 -0.20403219230875963 -0.11318652560363754 -0.10515557295342762 -0.1509967135689582 0.03549127995449734 -0.22517401150877037 0.32082934525131357 -0.09635522517598544 0.39813041485521156 0.15002488200879546 0.14649450178189688 0.0614341952274559 </vector>

<vector>0.150599413623786 0.13993641109933944 0.08166229593829075 0.14258228771367162 -0.3040373546859745 0.04168462007135852 -0.5152942669164705 -0.056310417614320735 -0.3162850241120604 -0.14232912111059548 -0.0948040438747798 -0.1485175156556511 -0.006348628012626104 -0.18518792020401428 0.384217895035222 -0.030536859851614667 0.42800711806119696 0.16769388198088475 0.19228259494341549 0.07098463357094187 </vector>

<vector>0.1667034272745881 0.13779193050555083 0.06073098822994264 0.1511673291591484 -0.2958657069202058 0.07272322535298083 -0.5488821738314555 -0.09055509973256848 -0.26219735304707814 -0.1286537106598505 -0.12008433989471022 -0.12803991490103472 0.01597254779069159 -0.20210645047039866 0.3760061078924949 -0.04180792922786914 0.4086849617513486 0.1511963567757339 0.19893154075438405 0.07828426319830757 </vector>

<vector>0.15432372090009971 0.14736101700240786 0.053293640136741205 0.16780628910133272 -0.2803636514362341 0.10683002852460546 -0.5443127532997613 -0.11070128306556715 -0.22593848448545112 -0.13198331377703443 -0.15052532002238972 -0.13460128442527433 0.026720941024333023 -0.22051349678358872 0.3512070548403585 -0.07044549429515065 0.4152141247377502 0.16679323049425468 0.20038072760455375 0.07945430722401457 </vector>

<vector>0.17601619258716725 0.1610842508390183 0.05963993924853013 0.18826136850482839 -0.3227825894515887 0.08871275173996589 -0.5265528653643662 -0.10361966021425258 -0.23161824838146175 -0.14328275438426502 -0.10481092290309421 -0.11557404431442327 0.010763476926176432 -0.22042053241938792 0.34765109100825187 -0.08727966262565841 0.41690855064892873 0.1648474340649686 0.1747853756814565 0.06727084880920618 </vector>

<vector>0.18777383199385805 0.17082956975402003 0.07831927135674352 0.19964017200317297 -0.3159753903582798 0.08554150893929002 -0.5376349585966183 -0.10372500813575143 -0.23039727914126645 -0.1288731272804825 -0.11045667173278695 -0.1365490568192886 0.027456626053041902 -0.22333671994037535 0.33857675537458914 -0.08683396859976425 0.40312565836840436 0.1656402420260478 0.15921215668231437 0.05766638805313149 </vector>

<vector>0.22719781669369116 0.1967457327339272 0.1642042405554638 0.19206899798338814 -0.2732150672467374 0.043035481773996014 -0.6033065295023239 -0.09618593560800452 -0.3126967579386052 -0.14809630788007555 -0.0668017366907858 -0.1275181656065557 0.05407760234759562 -0.1868799126801639 0.30419074832621434 -0.06506242653271138 0.3592523052848003 0.17155807558819353 0.14709737817068747 0.020334460228006418 </vector>

<vector>0.19628092530259655 0.18103614262377837 0.14120683528488853 0.17872681789653005 -0.2883501127475768 0.05546672590828529 -0.5749240468123386 -0.0830240282327764 -0.3458312056837555 -0.14221296931831867 -0.054677373948527744 -0.10421455757549099 0.08297942860346923 -0.1979896312133986 0.3402922192662934 -0.057977662224705115 0.37180520092061903 0.1760670852256686 0.13121812981433206 -0.005877923089572273 </vector>

<vector>0.15760157727570984 0.15594765980959688 0.10098339398908485 0.15093205534888077 -0.36420119486136615 0.05140742518356228 -0.506054926162758 -0.03987919032554822 -0.24158514887050178 -0.11297245311966703 -0.08636258933447205 -0.16927490657168923 0.025855989338323303 -0.213154668981329 0.3382569934519864 -0.10065318074585115 0.429828725851396 0.23074663950540147 0.14567717932259805 0.04690061989664328 </vector>

<vector>0.18011647033697667 0.15877970235362576 0.11712779786061761 0.147036015949146 -0.33723297110222306 0.02055304363651607 -0.5121131493879918 -0.025428814479273018 -0.30104195343323803 -0.13161209936100274 -0.11120170496923688 -0.15195704942416496 0.044880632456439626 -0.21198060011134667 0.35150381430895394 -0.05610295925539818 0.40095286117245 0.20975398851908 0.1670082027572522 0.040958772172817924 </vector>

<vector>0.1878331335217122 0.17147622752308073 0.11917385843744532 0.1713741424899342 -0.31400137451414395 0.04639535066109681 -0.538006580283997 -0.06214164605832279 -0.30080485195019047 -0.13465839017762204 -0.09576086608653445 -0.13308267319138015 0.05465839411278698 -0.211461949072571 0.34634862114648307 -0.06364921315925079 0.38903202389804725 0.18928982791637597 0.15152764171839112 0.02645832306865923 </vector>

<vector>0.18710200492556325 0.1746128375918379 0.09918694216683001 0.18835959362412644 -0.31642103969263186 0.06316628243848893 -0.5269825446516607 -0.07797209546291882 -0.25554139673357834 -0.13015010185354492 -0.12140351934183843 -0.1430764125744844 0.03611512127845217 -0.22441561589296755 0.3472498298642018 -0.07686701799764885 0.3943380096010727 0.1820484100043793 0.15635659258358936 0.04429412012273206 </vector>

<vector>0.08695430821005273 0.15638027088231318 0.02905017132189347 0.18233181181206967 -0.28974906128938055 0.16886482056935104 -0.454827182288586 -0.06461747185963401 -0.11376097207249342 -0.1289253365194904 -0.1862008936563313 -0.23789970891224832 -0.050731127382191835 -0.3014936683817288 0.3781139299058664 -0.14085860945962578 0.43919925186567665 0.2564872858497625 0.16195157538549385 0.10973060601923088 </vector>

<vector>0.09365895288907805 0.15390725777725797 0.02593313645140933 0.19664208628406787 -0.3020040731194304 0.15505467462690004 -0.42644533449698824 -0.08507479646903214 -0.10674207525911342 -0.11444604005748253 -0.22208637925294392 -0.21210499865190585 -0.056697143072052286 -0.29765696958903665 0.39410400996011885 -0.14933407076561744 0.46025687920165764 0.26294443043084437 0.14002202669826422 0.09006842641400424 </vector>

<vector>0.12322875740483484 0.16567159779260854 0.027909538030335704 0.1888397129560784 -0.2948301718568239 0.1255539827874013 -0.4454554429765203 -0.06702328307603116 -0.12464109915587034 -0.11939685358947724 -0.2359192380320294 -0.20403446110123405 -0.02676863447828163 -0.2850013605144696 0.38833400676216384 -0.1410521981547981 0.4364610201056103 0.26216174034034173 0.15168126419658093 0.07428112255957998 </vector>

<vector>0.13323870679944572 0.16213252806923764 0.04116365555398592 0.20650632968822788 -0.3180266700068909 0.11696139226706276 -0.4521887781749961 -0.08083306171773949 -0.13680632287926336 -0.1154082360107174 -0.20779961793909826 -0.19363676426438456 -0.018060674244362713 -0.27419707275520316 0.3702008731825544 -0.14814326813186263 0.43120059375157344 0.24131706561568572 0.15707823395705195 0.08530108723969314 </vector>

<vector>0.17825379840262853 0.18296247863082596 0.08819392932314743 0.20373148577304281 -0.3015498856863526 0.09503134700654882 -0.48365451951239885 -0.09008130954127667 -0.18001503742880529 -0.1164573591733462 -0.21346333765709802 -0.17624077535107546 2.3254219772155145E-4 -0.2689826856113657 0.3679779477168932 -0.10243337427564563 0.39203416670825 0.2176566936476661 0.15199039593601382 0.05481349889462591 </vector>

<vector>0.18569904926111971 0.18153271549841304 0.100067696706301 0.1940644663792722 -0.3192863542054711 0.05756198771508016 -0.505306095074367 -0.06804963219468249 -0.23542205910927785 -0.12691878810253016 -0.15799302020619413 -0.1595975077127847 0.02623034366952701 -0.23844817866595658 0.3568241130715331 -0.08011787223393162 0.3908563465367664 0.19121516007071437 0.1583299793500627 0.04875764924640576 </vector>

<vector>0.19174134445516755 0.18702283027257519 0.11282221862892564 0.19010231974064726 -0.3398881372374285 0.014488333700202707 -0.5052812210590417 -0.036095491579851785 -0.2707097431654501 -0.1341489032806996 -0.13911220361964627 -0.1594753352127941 0.04234336753240769 -0.2219462344935365 0.3552445616335045 -0.061053224428499905 0.38619686330097586 0.17394037656009745 0.1666429495305852 0.04716532872185919 </vector>

<vector>0.16220097931582314 0.19013549351880152 0.09713632040126573 0.2000010438320004 -0.3062435778764243 0.04437173973553516 -0.5131728157699911 -0.06653606785846257 -0.2467415702518783 -0.13022290673215878 -0.16015184543053526 -0.15494579403239023 0.044476488456822595 -0.2172121828679366 0.361453144313147 -0.07795008797466392 0.40605026904392516 0.1736470704962532 0.15499260779784507 0.038711691883021794 </vector>

<vector>0.13751138324751508 0.1868926920752578 0.0674994730501027 0.19875332013164515 -0.25103096908817757 0.09015257100026414 -0.5289592611951851 -0.07853301214273092 -0.24959060962571736 -0.12404225173586736 -0.14471351604273092 -0.16660100052115126 0.030897944466509595 -0.2506436687792047 0.37159768787809877 -0.07837994765118084 0.39150504473732095 0.18651208819122875 0.17528282257226357 0.035889209431739524 </vector>

<vector>0.13344917430250414 0.17245979703649528 0.06274641213847654 0.18553583840203564 -0.23391288604539082 0.1055255249575368 -0.5402364906917732 -0.09268620048187388 -0.2075175025572299 -0.10925006649827981 -0.17621305782142785 -0.17058917886836036 0.004576898362904155 -0.26219908691220034 0.382103451633732 -0.0758358105318331 0.39118909912693367 0.20189751639505502 0.18381490155127136 0.04514166650142502 </vector>

</ancestpheno>

<taxapheno>

<vector>0.1951871472998794 0.12607836341490491 0.0472370286147979 0.14311341066244065 -0.303196114638409 0.06965502746297707 -0.5870395012781343 -0.10465359851781644 -0.24436855054372322 -0.11164869709192189 -0.11492365578696051 -0.10100094462217933 0.027545330360365952 -0.20061793442359366 0.392593373801903 -0.024441433536841767 0.38283364245509915 0.11910195785206425 0.20413129971518218 0.08441384879996619 </vector>

<vector>0.12025154283854465 0.1432068696626537 0.03950999293175189 0.1639901696400206 -0.2224426579369091 0.15905410848086765 -0.5575032207034619 -0.1379290892498785 -0.183999852027815 -0.12401347628698843 -0.22668069726936227 -0.1601898940603652 0.05342679835613007 -0.2390135074609791 0.3299639656203274 -0.08224889103192415 0.4200488618129742 0.18433590064206296 0.22742526637782048 0.09280780966453041 </vector>

<vector>0.15138456016511787 0.17935572331885047 0.10153555235147863 0.16197830218476506 -0.35068355934247636 0.08024540688984783 -0.47265528672689244 -0.06976972878768413 -0.263855809866341 -0.0713188747332409 -0.06418730684905206 -0.18466611880757558 0.019866581986814316 -0.24247980697439453 0.33173797314632497 -0.14223772925305483 0.4432793752037886 0.2762968530628688 0.1035779199312379 0.012595973099617632 </vector>

<vector>0.18595102486754467 0.16506216576062677 0.04730690625210692 0.19733764440997958 -0.372008726560252 0.07376671775600134 -0.4977108841967188 -0.09643268944143843 -0.2385189815176675 -0.16899182209527827 -0.05345077695410588 -0.07557179169870719 -0.02188713629884726 -0.2174113805342009 0.35316946280980716 -0.10455952498206003 0.4323858688406318 0.16210882967460508 0.16476324275750073 0.06469185115047234 </vector>

<vector>0.07961190310875545 0.1946611951410154 0.01659079935426917 0.2091486590222286 -0.2588393617370827 0.15634129033472005 -0.45554789752452923 -0.06380574441778887 -0.13400529398672026 -0.08596329413601793 -0.18959294809537036 -0.27102823629640976 -0.024762961650608022 -0.32292387799583094 0.36826347200775145 -0.13830360527786725 0.42979857901118534 0.2562502954072815 0.1684837095123492 0.0656233182186693 </vector>

<vector>0.14931869404096232 0.19713153354521484 0.08414496560463586 0.15309295912357376 -0.3002210656785855 0.10663707388894059 -0.5093152831205645 -0.081069908152743 -0.1891223063390893 -0.1167236328530485 -0.20652168723447797 -0.16366567943265237 0.044713656450276335 -0.24770905672720106 0.3554558038893529 -0.10318023265217517 0.39468254258985996 0.22835107275359254 0.17686467979762963 0.027135870506498447 </vector>

<vector>0.11871574623637687 0.13337317969718085 0.008504357715085753 0.13471576400995192 -0.3959078557128905 -0.017253853252735124 -0.5177746122440644 0.0241481765436372 -0.3888377971486907 -0.1597186189068883 -6.139492552643448E-4 -0.1065025682311643 0.13179068005482056 -0.13315228854560324 0.35078753590373485 -0.06507592388036093 0.4278043797101948 0.13242332291350808 0.26553151474069725 0.057042809652473964 </vector>

<vector>0.17381182569238648 0.17488646761432658 0.1402424068617573 0.17273731321626723 -0.27783389648184964 0.07696934528976164 -0.5834590306506943 -0.09074450303200052 -0.42399200716247115 -0.1438842098972589 -0.001469519068262477 -0.05204283392853763 0.14020228935002366 -0.19562703188746092 0.37033728832618207 -0.04522134698215293 0.36713127357901043 0.1673533521724377 0.09502936955391761 -0.06442655256538193 </vector>

<vector>0.25732639176397276 0.19591537890831404 0.1159729733683741 0.2742824321641643 -0.29015860542150124 0.14723535275185437 -0.5871425761212826 -0.2165615360767945 -0.17742535120112493 -0.08878752663778497 -0.08764490157841434 -0.16548977693961103 0.001682804626048659 -0.23572506577758506 0.2760991824655091 -0.11499033018841405 0.37995129386138593 0.13214522612726667 0.11133878823703193 0.0719758456685905 </vector>

<vector>0.0707937930523469 0.13966990465685183 0.02083970000199982 0.218754734084055 -0.3214329862120867 0.17074522052394647 -0.3790533782258588 -0.12358363447143035 -0.08182415454897712 -0.09501593006348018 -0.2441390060704704 -0.19438082594223532 -0.09259166735568403 -0.3064758798709113 0.41586409321232537 -0.16609140468242808 0.5051103656336863 0.27018426510242977 0.10643324051271832 0.08619355066320211 </vector>

<vector>0.2158236391473198 0.205222192324827 0.12335043570915546 0.2106236612516283 -0.26733663284669595 0.1105706610375029 -0.49346868528783344 -0.12136123471140753 -0.16781673029787492 -0.10704505340679103 -0.2745973748260007 -0.17548805407605725 -0.007472042832000213 -0.2943028054129374 0.3769088568965917 -0.07903898246114267 0.35404555983641056 0.22043785525659867 0.1405629745009271 0.030381760197778922 </vector>

<vector>0.16446862977401797 0.19434969409519628 -0.00303785181087767 0.23374999673589814 -0.3158296032090202 0.14378130884169754 -0.47876534738330484 -0.0838727879181677 -0.20534388022567643 -0.13245355023804073 -0.117838230274857 -0.20767399793454983 0.014312875962880492 -0.24686353062968394 0.4134241240118624 -0.08079566050504278 0.4008044641971498 0.12498206818854998 0.1278048189578251 0.054796459364143346 </vector>

<vector>0.22732400478855957 0.18940028180051063 0.1412626387792109 0.1762414490106687 -0.3941344796303907 -0.05846872635000784 -0.4973647523327667 0.026299225313590212 -0.32996560013519455 -0.14530501500740986 -0.09919174522220843 -0.1638827038932076 0.05632327047087244 -0.21017834194671614 0.3474564275158328 -0.025091713076904245 0.3616839743222371 0.15695889911332572 0.18660626144384748 0.05402664503615016 </vector>

<vector>0.08417968681133416 0.11534627842874048 -0.021552774435733474 0.12233657384135552 -0.2317350400767907 0.08836347336946773 -0.5136113911426816 -0.005307729275154925 -0.3476678910416658 -0.1639281117583537 -0.0887191396308396 -0.1530917675315696 0.07074734720374984 -0.22746741552228317 0.42396664534636785 -0.0905704674128727 0.40235220797322574 0.19292618120849916 0.22204034899303388 0.12139298465217133 </vector>

<vector>0.11688399612421783 0.19808278567047646 0.04261568661056578 0.2107230781608993 -0.21293644334271758 0.12056044830772028 -0.5334684771237911 -0.07637676808785611 -0.29451275606804395 -0.13265378197716354 -0.09777564487622942 -0.17426802866270322 0.04364044657980178 -0.27251973655747713 0.3712364676874173 -0.08135394444704544 0.3772757660411042 0.1839916776823782 0.18704095836767445 0.023814269910771853 </vector>

<vector>0.07599747875400609 0.16200198065877802 0.05330111322650596 0.16865637701772843 -0.28207304378124337 0.10709138805667605 -0.5034506519731277 -0.09985576223413806 -0.21317751119455258 -0.08318723695714189 -0.1208423601131344 -0.1956599687516383 -0.00192093601908374 -0.24290395146326696 0.3349030598999642 -0.11614608110550699 0.47179952131961483 0.21162865722504434 0.18546332988105063 0.08837459755346531 </vector>

<vector>0.19491470021350804 0.14891521972819918 0.13122614115532313 0.11880185000862255 -0.33349634393115984 -0.03614364493511184 -0.49227794171721956 0.02573439294605307 -0.36073585947902204 -0.1472054547857194 -0.1514816594867031 -0.15351356850942613 0.05412751391820709 -0.21132518228014002 0.3699058283283917 -0.004006483861092799 0.38399783376790786 0.20922549813546398 0.20381978723076705 0.049517373553151715 </vector>

<vector>0.03728187901853817 0.14262674081092783 -0.1047990214649886 0.20608225098636776 -0.3799751366640846 0.12670923483146115 -0.47667550502271705 -0.08052629430588625 -0.24773265906381223 -0.04768337896136571 -0.11181339959546585 -0.1034331736165926 0.20420222044682662 -0.21970636057119325 0.4635145515630765 -0.13746883291025302 0.4188116822807115 0.09139875294472609 0.1971853885019156 0.022001060791808285 </vector>

<vector>0.1278667513525309 0.14731639372091784 -0.08750824369075327 0.13898466345834637 -0.39665054004146094 -0.017137864267937536 -0.5879299089302238 0.15437472211893755 -0.19959649937419283 -0.19219855369336772 0.0030290492185790618 -0.15244615086917093 0.1672090465035979 -0.19310946883331845 0.3888787452387072 -0.08688428005798494 0.4019732141862704 0.1440990048279648 0.1827283855369447 0.05700153359561304 </vector>

<vector>0.10056653569739009 0.1241730946793254 0.05845326013329342 0.1278063190066643 -0.3335226656125167 0.04329130423647648 -0.4912482527935145 -0.041612898066041994 -0.28775439183828944 -0.1431747744479987 -0.08362527189605255 -0.1542418108642573 -0.027296814126713736 -0.17661597152034322 0.38566586638136485 -0.032612494536435555 0.48452558500757903 0.1734241376980838 0.19423614904745937 0.07956309381452677 </vector>

<vector>0.16223097517705942 0.13742916909319547 0.09944637445059534 0.1739171917157656 -0.2822760007384342 0.07855687847056532 -0.5730605856750948 -0.08302603186269591 -0.19338928113069934 -0.11762577180826689 -0.2796474697429457 -0.14805155550561386 0.14368584284744934 -0.20595035545037788 0.35082396465559035 -0.029102406443939476 0.38687561722946684 0.15468479519225634 0.18531056292701295 0.03916808659911146 </vector>

<vector>0.12938696535749336 0.15802690199773298 0.057993351226850495 0.17231835667242634 -0.21679480300260429 0.1208984789148095 -0.5515137201883616 -0.10683938882101685 -0.16544439548874276 -0.0944578812606924 -0.207712599600125 -0.17457735721556966 -0.0217441477407012 -0.27375450504519633 0.3926092153893656 -0.07329167341248537 0.3908731535165467 0.21728294459888145 0.19234698053027927 0.054394123571110516 </vector>

<vector>0.1427886125259803 0.18097500753132986 0.01663182208561174 0.16337072289593937 -0.2644597724441503 0.10464588146824066 -0.45773221625757665 -0.03516199104132145 -0.13037489932923432 -0.12833628470023195 -0.2778717169040456 -0.2063616203874117 -0.005548086118430457 -0.2831500391991691 0.40069713714381816 -0.1256792555669142 0.41792558736360014 0.28222372497449544 0.15794353193442695 0.04747385402504267 </vector>

<vector>0.04340281677073364 0.23519528414466107 -0.01735514718277725 0.24457874075301 -0.34955316652204477 0.15249424761185293 -0.3817222510207044 -0.11385173615006504 -0.10104191151423302 -0.06547251385376839 -0.20517681155577847 -0.18094493825111407 0.01457070119355935 -0.3019655407942967 0.4469045150748752 -0.1927549709988079 0.49301976647564105 0.18405673268079265 0.05695148828072882 0.03866469485773572 </vector>

<vector>0.08759206863232466 0.12057235972866581 0.044626578160002796 0.1412046901299123 -0.3084037490116289 0.19519849674643128 -0.48248831484424065 -0.04497187469208005 -0.10053554697164727 -0.18636667536497123 -0.14692335362067865 -0.23056589178842918 -0.07073327742391611 -0.2839001575603182 0.3719743077497281 -0.13493815233539225 0.4275422973841876 0.2502671317111621 0.17734898994586862 0.17350007342501933 </vector>

<vector>0.0982335645908732 0.13776350778427782 0.007387499308474579 0.2269477903355617 -0.3576999524774965 0.13029884700723782 -0.42745637203606873 -0.08539459253590996 -0.10576283205311407 -0.11037049526932903 -0.17401627812816717 -0.2006350563408445 -0.027645930452529197 -0.268607172139774 0.3542906650686059 -0.2009442319651443 0.465106594440861 0.24413276285905022 0.16756304173856143 0.12680864026487423 </vector>

<vector>0.09442007647076782 0.21829539074743645 -0.013780707663920684 0.2574989421965418 -0.31271184632872595 0.12271624328769233 -0.5008647848954842 -0.17086050096378183 -0.18089097069533555 -0.06650600675476742 -0.07849147421606464 -0.14257445112458303 -0.007094123305801926 -0.24036127654716496 0.33534126202510534 -0.1306353921922373 0.48318902640525613 0.12881411862398343 0.1808835422042035 0.02361293272688058 </vector>

<vector>0.15809643119546737 0.14306627620514567 -0.008172303658612951 0.14845476135434252 -0.3081798916487507 0.09299220539276788 -0.4747546260953984 -0.06310459658897712 -0.25364700907936283 -0.13023978102014555 -0.12691748728533525 -0.14562935459051324 0.008987236428648255 -0.22259390777030805 0.36594974725376833 -0.09830052665277746 0.4558583896359029 0.2371621364463524 0.1827795132536738 0.03819278722411354 </vector>

<vector>0.05373607934244909 0.12103278237271378 -0.08346517575071019 0.10895632335381138 -0.317258145998264 0.08380035850450414 -0.5344912277938972 -0.11632251849773695 -0.24467408740663418 -0.16891062551143582 -0.03989409259666207 -0.11478867128064976 0.015618836846831923 -0.04949829589807812 0.31016447875810854 -0.1766331953553978 0.5772121149133086 0.13940570435650748 0.26305121968546885 0.17295813795576187 </vector>

<vector>0.18452827789938178 0.1578442081131409 0.12580263945163941 0.14877321497520082 -0.28272369152520255 0.00903933062461452 -0.5057523741244405 -0.03676325504434809 -0.39890332745081597 -0.1551588782239383 -0.08070251983357296 -0.1632708212016624 -0.007721617701859853 -0.17684133862130177 0.39098171083180355 -0.017190155790538612 0.39081080742466445 0.178461151468841 0.18368009502840232 0.05510654369999177 </vector>

<vector>0.026751225258850236 0.10967594482783842 -0.02177861433850373 0.0011797851828254354 -0.3228036714517068 0.08807600028113069 -0.5448209635059172 -0.08648907881465104 -0.3110551674755309 -0.17867884607236129 -0.04458587305389383 -0.11372740959736538 0.009971959160801347 -0.01888920451666681 0.3688845682094308 -0.07483930872522432 0.5615893500912009 0.13517417949502733 0.27784718710526946 0.13851793793944717 </vector>

<vector>0.23777130427833806 0.20375958855342538 0.17064769194926735 0.20385552247477504 -0.25636079075605106 0.02275397977209667 -0.5742784738088463 -0.05788718630520164 -0.34057505703045365 -0.19029897509135862 -0.08509115041100752 -0.15372026906664024 0.046494746848152274 -0.18925987040819986 0.2950315578288288 -0.07179084241479965 0.3544720161050883 0.18949282037489018 0.15188815499668423 0.043095232111012814 </vector>

<vector>0.24754122050013938 0.20544146702457722 0.18075819443223692 0.19362465357885852 -0.2749342982365849 0.05088573964160347 -0.660717067885786 -0.14764659228603336 -0.25168401110160704 -0.11177697923054991 -0.060636685712820784 -0.12461967017753713 0.03275863159116241 -0.17339023641889326 0.2772484678835192 -0.06541877495862927 0.35147969882869506 0.14911432116402448 0.15818584970104618 0.023786071662579563 </vector>

<vector>0.1573502102447867 0.1964909582085713 0.11108726952476863 0.21114749162370838 -0.32781162730366614 0.02847431450613884 -0.5052779650557467 -0.08497969985280472 -0.21992435796446777 -0.13247756517990966 -0.19662981662922788 -0.1387610463632254 0.060188153371549966 -0.17904664533106893 0.35751718342783756 -0.09441709184431087 0.44044889909347845 0.16048874673743388 0.1230520512906871 0.03308053749546694 </vector>

<vector>0.14130370132503525 0.12970755375631332 0.08428683175515975 0.14378184791272985 -0.4046870541393999 0.053423825024320595 -0.5333963423733887 -0.02443902770968508 -0.15985768331192723 -0.13598638526475829 -0.0836987561851257 -0.17120155148332827 0.012820753571713042 -0.18500359985824577 0.33152919290067867 -0.1036188537291002 0.44525394117795114 0.20618907693425859 0.16644541527930426 0.08714711441749542 </vector>

<vector>0.16077557539723733 0.1095156462397735 0.12438286462612014 0.11655930417191748 -0.34813836841514095 -0.057981261715176355 -0.4971924604345988 0.06460646028332771 -0.42263995057939313 -0.1623894890336857 -0.02467454793813525 -0.16142008216232598 0.05598714556331154 -0.18267125151911254 0.33044874488855347 -0.030830753356393115 0.4366426876792925 0.2368886671337143 0.18440830921275328 0.06772275995796062 </vector>

<vector>0.01981473558862308 0.13544030982839367 -0.06674715585977656 0.16592655219665298 -0.3241356872263724 0.13207010774324235 -0.48742602613164226 -0.0286025804702394 -0.22183949519644225 -0.1097262356454012 -0.1803192834763619 -0.13094886409193607 0.1615214834493024 -0.2417060411714546 0.4701072132668567 -0.06961735112950476 0.4206669688754865 0.12811136012720667 0.20835724671032643 0.019052742613040304 </vector>

</taxapheno>

<treelengthvec>0.0 0.0 0.0 0.0 0.0 0.0 0.0 0.0 0.0 0.0 0.0 0.0 0.0 0.0 0.0 0.0 0.0 0.0 0.0 0.0 </treelengthvec>

</phylogeny_content>

<morphojset>

<morphojset_contents visiblename="BranchDiffs, unweighted: Mandibular_articulation" uniqueid="44" alignmentmethod="0" dimension="2" hasprocrustesfit="false" hasrawdata="false" linkedoutlinenumber="0" linkedwireframenumber="0" nincl="72" nlmk="10" nobs="72" objsymmetry="false" procsumsquare="0.0" procrustesfitfromparentset="false">

<datavector>

<datamatrix visiblename="Centroid size" matrixtype="1" iscoordinates="false" issymmetrycomponent="false" isasymmetrycomponent="false">

<data>

<vector>-39.00076171910405 -0.04791234955832202 </vector>

<vector>-0.5983606258382679 0.001012992432929849 </vector>

<vector>-9.709366788803095 -0.010564750708405235 </vector>

<vector>44.495820798307705 0.05190305793490513 </vector>

<vector>-7.624511907331225 -0.008989376380515779 </vector>

<vector>40.75596596571859 0.05370466968006582 </vector>

<vector>2.7563829879807145 0.004351149701114387 </vector>

<vector>20.565822968988186 0.023772811526030502 </vector>

<vector>-34.01794880158934 -0.04015139630652875 </vector>

<vector>12.007264955695177 0.014252082215898909 </vector>

<vector>-23.58071335920397 -0.027049631948296238 </vector>

<vector>9.836685685943507 0.014166670192863329 </vector>

<vector>62.864543909066015 0.0711398804254415 </vector>

<vector>13.800259568217939 0.018825794726564204 </vector>

<vector>45.60801663704615 0.055504664891042665 </vector>

<vector>12.974221585297641 0.015761419084550532 </vector>

<vector>40.82516453157939 0.04597099573171892 </vector>

<vector>63.064139799507416 0.08336967283803443 </vector>

<vector>-5.003069444879088 -0.0040431583010729355 </vector>

<vector>-39.49218063312321 -0.0483118389902506 </vector>

<vector>10.43618388553898 0.012929640581814006 </vector>

<vector>-47.481287252410425 -0.05797533363895635 </vector>

<vector>9.265333310724714 0.011033017174759685 </vector>

<vector>-27.713761206075446 -0.03434335511165365 </vector>

<vector>-8.957550386519415 -0.010601599182096244 </vector>

<vector>-1.9749352550591084 -0.0018498433868252562 </vector>

<vector>32.93906785641866 0.04229347164835762 </vector>

<vector>10.187200102931797 0.012698733759425096 </vector>

<vector>-22.323571188188225 -0.02392323134401053 </vector>

<vector>46.769981636038096 0.05753384108200876 </vector>

<vector>-132.0575162847191 -0.18306000758135887 </vector>

<vector>12.481789255663102 0.014853770462039506 </vector>

<vector>-30.10707065396798 -0.03470375835977624 </vector>

<vector>-40.21627727905707 -0.04579084374474718 </vector>

<vector>-20.83067684268576 -0.023686955764665285 </vector>

<vector>26.13503084688591 0.030782337289983808 </vector>

<vector>-27.079316658958987 -0.03290324307201864 </vector>

<vector>28.891413834862192 0.035133486991093754 </vector>

<vector>42.69167340308013 0.05395928171765796 </vector>

<vector>20.368102214890087 0.03003605037364565 </vector>

<vector>-17.877075520133644 -0.02017668491879121 </vector>

<vector>22.878890445584148 0.03352798476127283 </vector>

<vector>43.24699266047571 0.06356403513491937 </vector>

<vector>-88.81052362424589 -0.11949597244644128 </vector>

<vector>-25.74638382473961 -0.03612629960840863 </vector>

<vector>7.192684031678368 0.006167172039947211 </vector>

<vector>2.1896145867979158 0.002124013738873387 </vector>

<vector>-16.892116556029578 -0.02020450931259621 </vector>

<vector>-3.9178949707331867 -0.0044430902280465645 </vector>

<vector>6.518288914804316 0.008486550353765665 </vector>

<vector>8.707903501601777 0.010610564092638164 </vector>

<vector>-25.310045299988474 -0.029540832213892365 </vector>

<vector>7.277801002911019 0.009222002091751946 </vector>

<vector>-31.722960716194393 -0.03869034746657096 </vector>

<vector>-32.32132134203391 -0.037677355033642 </vector>

<vector>12.174499456272656 0.01422570290126135 </vector>

<vector>-13.135545843716272 -0.015315129312630127 </vector>

<vector>-17.62528139830613 -0.019849987897738508 </vector>

<vector>2.940541570681262 0.003922823628291994 </vector>

<vector>-30.540043631490903 -0.03425170647307141 </vector>

<vector>10.285120900088032 0.011719289258646626 </vector>

<vector>13.225662470769294 0.01564211288693773 </vector>

<vector>0.09011662705313483 3.269835743076044E-4 </vector>

<vector>-16.58206229385121 -0.0195909755626138 </vector>

<vector>-4.574797338156941 -0.005338893346715778 </vector>

<vector>4.690535972567432 0.005694123828044795 </vector>

<vector>2.715600717508323 0.0038442804412186504 </vector>

<vector>-20.865112641696214 -0.023205351507078475 </vector>

<vector>-20.774996014642966 -0.02287836793277087 </vector>

<vector>42.08954789442271 0.04826151249266886 </vector>

<vector>1.8732706153650724 0.0024706687479225664 </vector>

<vector>47.48128725241088 0.057975333638964344 </vector>

</data>

<namesvector>Centroid Size Log Centroid Size</namesvector>

</datamatrix>

<datamatrix visiblename="Procrustes coordinates" matrixtype="2" iscoordinates="false" issymmetrycomponent="false" isasymmetrycomponent="false">

<data>

<vector>0.028483720025291315 -0.011713567090645916 -0.01349395961514474 -0.008053918496707752 -0.007330407718203202 -0.00306819789000376 -0.03815732744667877 -0.014098498785247965 0.017828802503354918 0.01700501356792862 0.005160684107749711 0.027038970278855393 0.011572782569674363 0.0014885160468049996 0.016587265909408122 0.017366495691027373 -0.025851319296249453 -0.03209439892366965 0.005199758960798134 0.006129585601658619 </vector>

<vector>-0.03407217806155506 -0.004154147339754166 -0.013783647204989313 -0.0038161194613121063 0.057920993499325 0.05222407995626219 -0.013190467403700668 -0.027227806184311365 0.04193863245763613 0.007969837490045997 -0.07615537724697255 -0.02558860963509088 0.026705857331797044 -0.018500010677390377 -0.021243089220031097 -0.011803396736773497 0.0048347370752239716 0.017542670147808276 0.027044538773266735 0.013353502440515835 </vector>

<vector>-0.006217017110591971 0.02340806350925359 5.521583623937787E-4 0.011046246835884288 0.013517635518889792 0.028837981706285543 0.0333996394358656 -0.029890538462135907 -0.022270660995839214 0.041653578386426124 0.022175282485419986 -0.015391212235886353 -0.005989407351508987 -0.02932513799306552 -0.006519020305661427 -0.04158454850720368 0.013450649352392596 0.0455502135574673 -0.04209925939136015 -0.03430464679702565 </vector>
[truncated: 153,336 more chars]
